# Tandem Quadruplication of *HMA4* in the Zinc (Zn) and Cadmium (Cd) Hyperaccumulator *Noccaea caerulescens*

**DOI:** 10.1371/journal.pone.0017814

**Published:** 2011-03-10

**Authors:** Seosamh Ó Lochlainn, Helen C. Bowen, Rupert G. Fray, John P. Hammond, Graham J. King, Philip J. White, Neil S. Graham, Martin R. Broadley

**Affiliations:** 1 Plant and Crop Sciences Division, University of Nottingham, Sutton Bonington, Leicestershire, United Kingdom; 2 Warwick HRI, University of Warwick, Wellesbourne, Warwick, United Kingdom; 3 Rothamsted Research, Harpenden, United Kingdom; 4 Southern Cross Plant Science, Southern Cross University, Lismore, Australia; 5 Scottish Crop Research Institute, Invergowrie, Dundee, United Kingdom; United States Department of Agriculture, Agricultural Research Service, United States of America

## Abstract

Zinc (Zn) and cadmium (Cd) hyperaccumulation may have evolved twice in the Brassicaceae, in *Arabidopsis halleri* and in the *Noccaea* genus. Tandem gene duplication and deregulated expression of the Zn transporter, *HMA4*, has previously been linked to Zn/Cd hyperaccumulation in *A. halleri*. Here, we tested the hypothesis that tandem duplication and deregulation of *HMA4* expression also occurs in *Noccaea*.

A *Noccaea caerulescens* genomic library was generated, containing 36,864 fosmid pCC1FOS™ clones with insert sizes ∼20–40 kbp, and screened with a PCR-generated *HMA4* genomic probe. Gene copy number within the genome was estimated through DNA fingerprinting and pooled fosmid pyrosequencing. Gene copy numbers within individual clones was determined by PCR analyses with novel locus specific primers. Entire fosmids were then sequenced individually and reads equivalent to 20-fold coverage were assembled to generate complete whole contigs.

Four tandem *HMA4* repeats were identified in a contiguous sequence of 101,480 bp based on sequence overlap identities. These were flanked by regions syntenous with up and downstream regions of *AtHMA4* in *Arabidopsis thaliana.* Promoter-reporter *β-glucuronidase* (*GUS*) fusion analysis of a *NcHMA4* in *A. thaliana* revealed deregulated expression in roots and shoots, analogous to *AhHMA4* promoters, but distinct from *AtHMA4* expression which localised to the root vascular tissue.

This remarkable consistency in tandem duplication and deregulated expression of metal transport genes between *N. caerulescens* and *A. halleri*, which last shared a common ancestor >40 mya, provides intriguing evidence that parallel evolutionary pathways may underlie Zn/Cd hyperaccumulation in Brassicaceae.

## Introduction

Transition metals, including Cu, Mn and Zn, have essential functions in plant growth and development [1]. However, when present at high concentrations, these metals, along with non-essential metals including Cd and Pb, become phytotoxic and must be prevented from interfering with cellular processes through compartmentalisation and exclusion [1]-[5]. Numerous transmembrane proteins catalyse metal efflux from plant cells. These include P_1B_-ATPases, of which one group transports Cu/Ag and another transports Zn/Cd/Co/Pb [2]. The most widely studied P_1B_-ATPase *in planta* is the plasma membrane protein HMA4 [6], which has been shown to transport Zn and Cd in yeast [2], [7] as well as confer Zn, Cd and Co tolerance in *Arabidopsis thaliana* [2], [8]. HMA4 is thought to be involved in Zn homeostasis and Cd detoxification, *via* metal translocation from the root to the shoot [2], [7]–[11]. At a subcellular level, the expression of *HMA4* has been shown to localise in the plasma membranes of *Arabidopsis thaliana* mesophyll protoplasts [8]. At the tissue level, it has been localised to the pericycle cell layer of the root vasculature [12]. In *hma4* knockout mutants, increased pericycle Zn accumulation, decreased Zn transport to the xylem parenchyma, and reduced shoot Zn accumulation have been observed [12]. In *A. thaliana* shoots, *HMA4* expression has been localised in the phloem tissue, at the base of developing siliques, and in developing anthers, especially tapetum cells, to supply Zn to male reproductive tissue [9].

A small number of plant species have evolved that can tolerate and accumulate high concentrations of some metals in their aerial tissues under natural conditions, including Zn and Cd [13], [14]. It is thought that 10-20 species of angiosperms are Zn hyperaccumulators (>∼0.3% Zn DW), with two of these also able to accumulate Cd to similarly high levels. In the Brassicaceae, the accumulation of high levels of Zn in shoot tissues occurs within *Noccaea* and its sister clade *Raparia* [13], [15], [16], but not in *Thlaspiceras* which contains Zn hypertolerant species (e.g. *Thlaspiceras oxyceras* (Boiss.) F.K. Mey; [17]), and not in the non-Zn-hypertolerant *Microthlaspi* and *Neurotropis* clades, which are more distantly related. Within *Noccaea*, Cd hyperaccumulation occurs in a subset of *N. caerulescens* populations. *Arabidopsis halleri* is the only known Brassicaceae Zn/Cd hyperaccumulator occurring outside of the *Noccaea* genus [13], [14]. Thus, Zn/Cd hyperaccumulation may have arisen through two evolutionary events within the Brassicaceae.

In *Arabidopsis halleri*, QTL involved with Zn and Cd tolerance co-localize with *HMA4* [18]. High expression of *HMA4* in the first back-cross (BC_1_) between *A. halleri,* and the non-hyperaccumulator, *A. lyrata* ssp. *petraea,* co-segregated with the *A. halleri HMA4* allele and with Cd tolerance [18]. Using RNA interference (RNAi), it was demonstrated that Zn and Cd hypertolerance were associated with *HMA4* expression in *A. halleri* [19]. These plants were sensitive to increased exogenous Zn and Cd treatments, translocated less Zn from the root to the shoot, and were phenotypically more similar to *A. thaliana* [19]. Conversely, expression of *AhHMA4* cDNA under its endogenous promoter in *A. thaliana* resulted in increased Zn concentrations in xylem parenchyma cells, resembling Zn distribution in *A. halleri* roots [19]. Subsequent sequencing and functional analyses of *AhHMA4* revealed that enhanced *HMA4* expression was the result of both tandem gene triplication and altered *cis* regulation [19].

For *N. caerulescens*, expression of a *NcHMA4* cDNA in yeast (*Saccharomyces cerevisae*) associated with enhanced Zn tolerance and increased Zn transport out of cells which supported a role for Zn efflux across plasma membranes *in planta* [20]. In general, P_1B_-type ATPases are more highly expressed in the shoots of *N. caerulescens* than non-hyperaccumulating *Thlaspi arvense* [21] and *Arabidopsis thaliana* [22], [23]. Further studies characterising *N. caerulescens HMA4* transcripts found increased expression as exogenous Zn was applied at levels which were either deficient or toxic to non-hyperaccumulating species [20], [21]. Despite circumstantial evidence for similar roles in Zn hyperaccumulation, genomic sequence data has not been published for *HMA4* in *Noccaea caerulescens.* The aim of this study was to test the hypothesis that tandem duplication and deregulation of *HMA4* expression, which occurs in *A. halleri* [19], also occurs in *N. caerulescens*.

## Results and Discussion

To test for tandem duplications of the *HMA4* locus in *N. caerulescens* required *de novo* sequence. To achieve this goal, the creation of a single copy genomic fosmid library coupled with high-throughput pyrosequencing were selected as appropriate strategies. Fosmid libraries yield large insert sizes, have high stability and reduced susceptibility to aberrent recombination, thereby ensuring maximum genomic sequence representation [24], [25]. By randomly shearing DNA fragments, these libraries also retain a wider selection of sequences than those based on traditional restriction digestion [26]. Sequences were generated *via* Next Generation Genome Sequencer (NextGen GS) FLX 454 technology as it offered the greatest read length (350–450 bp) of current pyrosequencing technologies, and is routinely employed for *de novo* sequencing [27]–[29].

### Construction and characterisation of a Noccaea caerulescens fosmid library

The genomic fosmid library was constructed for the self-compatible Zn and Cd hyperaccumulator *Noccaea caerulescens* (J.&C. Presl) F.K. Mey., from a first generation accession from a geographically isolated population in Saint Laurent Le Minier, southern France (supplied by Guy Delmot, Saint Laurent le Minier, France, 43°55′48″ N, 3°40′12″ E) [30]. Such populations are self-compatible and highly inbred [31]–[34], and demonstrate low levels of heterozygosity and high inbreeding coefficients [34]–[37]. The creation of a laboratory inbred line was not pursued, since this could result in an accumulation of mutations [38] leading to increased genetic load [39] and reduced fitness, as well as gene copy number variation [40]–[42] and perturbed sequencing results.

To further prevent potential allelic perturbations in sequencing results, the library was constructed using leaf genomic DNA from a single plant (250 Mb), and cloned into 36,864 *Escherichia coli* EPI300^TM^-T1^R^ host cells containing highly stable, randomly sheered, ∼40 kb genomic inserts, representing ∼5.9 fold genomic coverage, while 454 sequencing reads returned >20 fold coverage. Such sequencing strategies compare favourably with those adopted by [19] to robustly identify tandem triplication of *HMA4* in the self-incompatible *Arabidopsis halleri.*

**Figure 1:**
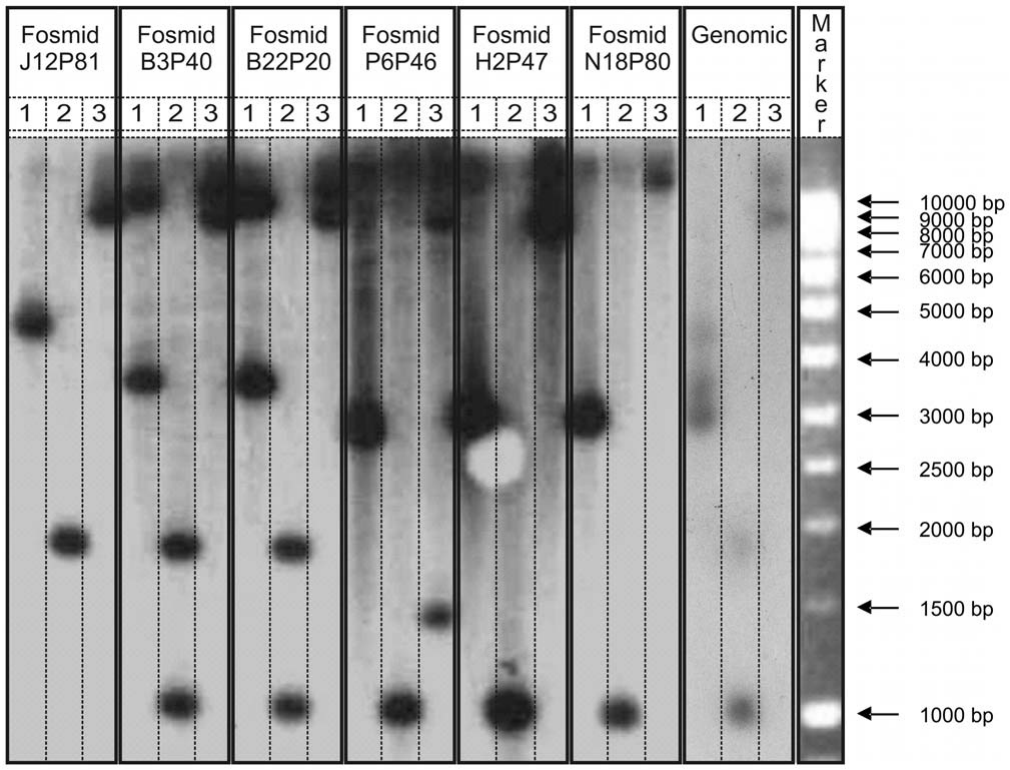
Montage of two gel blot autoradiographs of *NcHMA4* tandem repeats from *N. caerulescens* genomic DNA and genomic library fosmid insert DNA. All DNA was digested with *EcoR*I, *Hind*III or *Bam*HI corresponding to lanes **1**, **2** or **3** respectively, resolved on two 0.9% (w/v) agarose gels, blotted, and hybridized with the radiolabeled *NcHMA4* library probe (represented by darkened regions). Fosmids labelled with ‘+’ contain tandem repeats of a *NcHMA4* insert. The DNA marker was a 1 kb DNA ladder (Hyperladder I, Bioline). Montage was prepared using CorelDraw Graphics Suite X3.

**Figure 2:**
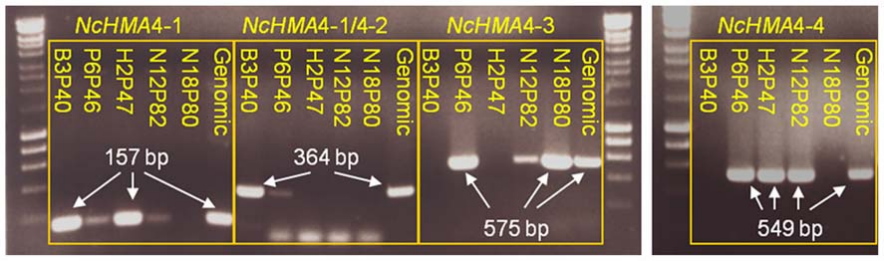
Agarose gel electrophoresis of PCR products from fosmid clones containing *N. caerulescens HMA4* sequences and *Noccaea* genomic DNA. Primers were specific for *NcHMA4*-1, *NcHMA4*-1 and 4-2, *NcHMA4*-3 and *NcHMA4*-4. Lanes were labelled according to fosmid clones or ‘Genomic’ *Noccaea caerulescens* DNA. The molecular ladder was a 1 kb DNA ladder (Hyperladder I, Bioline). Gel contained 1% (w/v) agarose.

**Figure 3:**
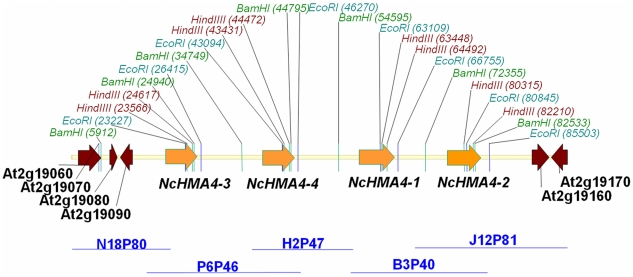
Genomic organisation of *HMA4* in *N. caerulescens*. Five overlapping genomic fosmids (horizontal blue lines) represented a 101,480 bp single locus of *N. caerulescens*. Genes and their transcriptional direction are represented by arrows and given *A. thaliana* Genome Identifier appellations (brown arrows, genes syntenic with *A. thaliana HMA4* flanking regions; orange, quadruplicated *NcHMA4* genes). Restriction endonuclease site locations used for fosmid fingerprinting are indicated in red; *Hind*III, green; *Bam*HI, and teal; *EcoR*I. Numbers in brackets refer to genomic locations in base pairs.

To elucidate the genomic sequence of *HMA4* in *N. caerulescens*, the library was probed with a radiolabelled *NcHMA4* specific sequence. Seven clones, N18P80, P6P46, N12P82, H2P47, B3P40, B22P20 and J12P81, were identified as containing *NcHMA4* sequences following PCR amplification using primers specific for the *NcHMA4* probe. Six of these fosmids demonstrated unique evidence of multiple copies of the *NcHMA4* locus following restriction digest fingerprinting (Figure 1). Initial pyrosequencing [27] of a pool containing all seven fosmids returned 3 Mbp of sequence at 5-fold coverage per fosmid. Sequences were assembled into contigs and aligned to syntenic regions in the *A. thaliana* genome to confirm the presence of multiple *NcHMA4* copies. Individual copies were assigned to unique clones through PCR analyses using locus specific primers (Figure 2). Fosmids were then sequenced individually to improve the specificity and efficiency of prior pooled sequence assemblies, and returned 2.4 Mbp at >20 fold coverage per fosmid. Two independent *HMA4* copies were identified in fosmids B3P40 (27,978 bp; *NcHMA4*-1 and *NcHMA4*-2) and P6P46 (31,521 bp; *NcHMA4*-3 and *NcHMA4*-4) (Figures S1 & S2, Data S1 & S2). Fosmid J12P81 (31,218 bp) contained *NcHMA4*-2 as well as two genes downstream to its 3′ end, whose sequences were homologous to the *A. thaliana* genes At2g19160 and At2g19170, and so demonstrated synteny with *Arabidopsis thaliana* (Figure S3, Data S3). Fosmid N18P80 (20,090 bp) contained 941 bp of the 5′ region of *NcHMA4*-3 in addition to four orthologues to At2g19060, At2g19070, At2g19080 and At2g19090, which were syntenic to this region in *A. thaliana* (Figure S4, Data S4). As indicated through locus specific PCR analysis (Figure 2), sequence data from fosmid H2P47 (20,258 bp) showed homology to *NcHMA4*-4 and its 5′ intergenic region, as well as the 5′ intergenic region of *NcHMA4*-1 (Figure S5, Data S5). Fosmid inserts, containing homologous sequences which demonstrated >99% sequence identity along 5′ and 3′ ends of between 425 and 14,866 bp, were assembled into unique contiguous sequences. Consequently, fosmid H2P47 assembled both fosmids P6P46 (containing *HMA4*-3 and *HMA4*-4) and B3P40 (containing *HMA4*-1 and *HMA4*-2) into a unique locus (Data S7,S8), flanked to its 5′ by N18P80, and to its 3′ by J12P81 (Figure 3). In support of this *HMA4* quadruplication, a genomic Southern illustrated hybridisation intensities for *Hind*III fragments, which were indicative of a 3:1 (1040–1050 bp fragment (representing *HMA4*-1, *HMA4*-3 and *HMA4*-4): 1.9 kb fragment (representing *HMA4*-2)) genomic ratio (Figure 1).

All five overlapping *N. caerulescens* fosmids spanned a single 101,480 bp locus in *N. caerulescens* and contained four *HMA4* tandem repeats (corresponding to At2g19110 in *A. thaliana*), compared to syntenic regions in *A. thaliana* and *A. halleri*, containing one and three copies respectively (Figure 3, Data S6). Sequences flanking *NcHMA4* tandem repeats remained essentially syntenic with *A. thaliana*.

### Analysis of NcHMA4 sequences

**Figure 4:**
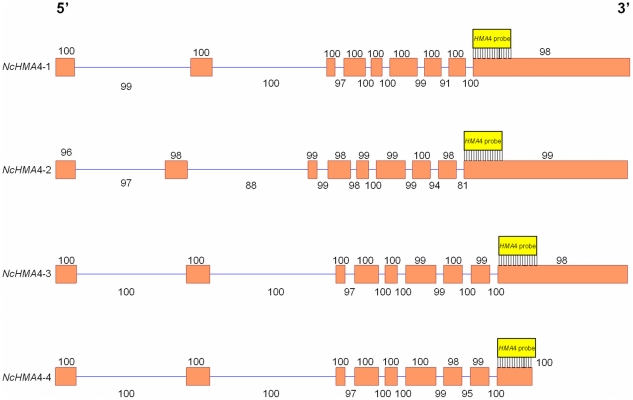
Genomic illustration of all four *NcHMA4* tandem repeats. Exons are represented by orange squares flanked by introns (blue lines). The *NcHMA4* library probe (yellow box) is illustrated at its site of hybridisation for each copy. Numbers above exons and below introns represent percentage sequence identities for each copy to a consensus *NcHMA4* sequence using Dot Matrix (Vector NTI 11).

Within the deduced coding sequences, all four *NcHMA4* gene copies share between 87 and 99% nucleotide sequence identity, whilst introns demonstrated between 81 and 100% identity to consensus *NcHMA4* sequences (Figures 4 & S6). The deduced coding sequences showed lower sequence identities with those of *AtHMA4* (between 76–78%) and of all three *AhHMA4* copies (between 62–66%), which may indicate that quadruplication was a relatively recent evolutionary event within *N. caerulescens* (Figure S6). *NcHMA4*-4 contained a truncation in exon 9 after amino acid (aa) 684 of the deduced protein sequence (Figure 4) and could indicate a functional but less efficient *in planta* Zn transporter, as recently reported for an *AtHMA4* which contained a comparable truncation after aa 713 [43]. At the deduced amino acid level, *NcHMA4* share between 92 and 98% identity, but only between 72 and 83% identity with *AtHMA4* and between 74 and 84% identity with the three *AhHMA4* (Figure S7).

Within the first 2000 bp upstream of the translational start codon, *NcHMA4* sequences shared 59 and 98% identity, but between 44–49% and 41–51% identity with *A. thaliana* and *A. halleri* promoter sequences respectively (Figure S8). *AhHMA4* regions shared greater identity, 53–88% with *AtHMA4*, as previously reported [19]. Significant sequence divergence from *A. thaliana* and *A. halleri* in the 5′-flanking regions of *NcHMA4* genes indicates *cis* gene regulation may differ between species. In *A. halleri*, high *HMA4* expression was regulated in *cis* and amplified by a triplication in gene copy number [19]. Increased expression of *AhHMA4* correlated with enhanced Zn flux from the root symplasm into the xylem parenchyma as well as up-regulation of Zn deficiency response genes in roots supporting its role in Zn hyperaccumulation.

### Expression profile of NcHMA4

To investigate the expression profile of *NcHMA4*, T_2_ *A. thaliana* plants (n = 30), transformed with *HMA4* promoters from *A. thaliana* (*AtHMA4*p::*GUS*, negative control), *A. halleri* (*AhHMA4*-3p::*GUS*, positive control) and *N. caerulescens* (*NcHMA4*-2p::*GUS*) fused to the *β-glucuronidase* (*GUS*) reporter gene, were analysed for *GUS* activity under identical nutrient replete conditions *in vitro,* 21 days after sowing (DAS).

**Figure 5:**
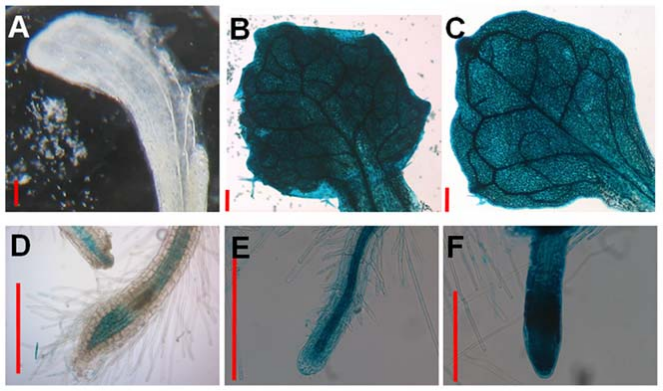
The spatial expression of *β-glucuronidase* (*GUS*) fused to *HMA4* promoter regions in *Arabidopsis thaliana*. The activity of *GUS* in whole leaves A–C and roots D–F from 21 day old *in vitro* cultured *A. thaliana* T_2_ transformants bearing pGWB3 constructs containing the *GUS* marker gene under the control of promoter sequences from **A, D**; *AtHMA4*, **B, E**; *NcHMA4*-2 and **C, F**; *AhHMA4*-3. Red bars represent 2 mm.

Lines bearing the *AtHMA4*p::*GUS* construct showed expression in root and stem tissue, although no staining was observed in leaf tissues (Figure 5). For both *NcHMA4*-2p::*GUS* and *AhHMA4*-3p::*GUS* constructs, transformed lines showed expression in most plant tissue including roots, shoots and stems (Figure 5). The *GUS* gene appeared to be similarly and more intensely expressed throughout plants when driven by either the *NcHMA4*-2 or the *AhHMA4*-3 promoters (Figure 5).

### Conclusion

The aim of this study was to test the hypothesis that tandem duplication and deregulation of *HMA4* expression, which occurs in *A. halleri*, occurs in *N. caerulescens*. A fosmid library comprising 36,864∼40 kb inserts was developed, representing a potentially valuable resource for future map-based cloning and genome sequencing in *N. caerulescens*. Following *de novo* sequencing, there was compelling evidence of tandem quadruplication for *HMA4* in *N. caerulescens*. Whilst it is hypothetically feasible that allelic artefacts can occur, even in highly inbred populations, here, the sequencing of multiple fosmids (including long-reads of intergenic regions/non-coding repeats which are overlapping between fosmids) provides very strong support for tandem repeats of *NcHMA4*. This observation is strikingly consistent with a tandem *HMA4* triplication in the *A. halleri* genome [19]. *Noccaea caerulescens* and *A. halleri* last shared a common ancestor >40 mya [44], and the current study provides intriguing new evidence that parallel evolutionary pathways may underlie two occurrences of Zn/Cd hyperaccumulation in the Brassicaceae. Further detailed sequencing is now required in a wider number of species.

An initial functional analysis was undertaken of *HMA4* using promoters from both *N. caerulescens* and *A. halleri* expressed in *A. thaliana*. Again, results were remarkably consistent between species, and the regulation of *HMA4* from both hyperaccumulator species appears to be distinct from endogenous *AtHMA4* regulation. *GUS* expression was driven more highly throughout the plant by *NcHMA4*-2p and *AhHMA4*-3p than by *AtHMA4*p, consistent with high levels of *HMA*4 transcripts in shoots and roots of *N. caerulescens* [20]-[22] and *A. halleri* [19], [45]. Throughout leaf tissue, promoters from both hyperaccumulator species drove enhanced *GUS* activity compared to *AtHMA4*p, which is consistent with a possible role in increasing Zn accumulation in leaf epidermal cells in *N. caerulescens* [46], [47] and mesophyll cells in *A. halleri* [48]. Moreover, expression of all three *AhHMA4* promoters in the xylem parenchyma and the cambium of leaves in both *A. thaliana* and *A. halleri* were hypothesised to be consistent with putative roles in Zn exclusion from particular cell types and metal distribution within the leaf blade [19].

In contrast to the relatively high, shared HMA4 sequence identities between all three genomes, *N. caerulescens HMA4* promoters (*NcHMA4*p) exhibited lower identities with those from both *A. thaliana* and *A. halleri*. We conclude that novel *cis* regulatory elements in *N. caerulescens* contribute to increased *NcHMA4* gene expression. Further elucidating these *cis* regulatory regions in hyperaccumulators could enable the manipulation of *HMA4* expression that may be exploited for use within crop systems to enhance Zn leaf accumulation for biofortification or phytoremediation strategies.

## Materials and Methods

### Library Construction

DNA from a *Noccaea caerulescens* (J.&C. Presl) F.K.Mey (∼250 Mb genome, 2n = 2x = 14) from a population originating from Saint Laurent Le Minier, southern France [30] was used to construct a genomic fosmid library by Warwick Plant Genomic Libraries Ltd. (Warwick HRI, Warwick, UK).

### Preparation of Noccaea DNA and bacterial cells for filter arraying

DNA (2.5 µg), extracted from leaf tissue of a single *N. caerulescens* plant *via* the phenol – chloroform procedure [49], was randomly sheared to 40 kb fragments and end repaired to blunt, 5′-phosphorylated ends. Fragments were size resolved and purified from a low melting point (LMP) agarose gel (without exposure to UV irradiation), before ligating to 8 kb Cloning-Ready CopyControl pCC1FOS vectors and phage packaging (Epicentre Biotechnologies, Madison, W.I., USA). EPI300^TM^-T1^R^ plating strains were streaked on solid Luria-Bertani (LB) plates and grown for 12 h at 37°C. A starter culture (5 ml LB broth) was inoculated with a single colony and incubated on a shaker at 225 rpm. for a further 12 h at 30°C. 50 ml of LB broth +10 mM MgSO_4_+0.2% (w/v) maltose (20% filter sterilised stock) was inoculated with 1 ml of starter culture and shaken at 37°C for 2–3 h until an optical density at 600 nm (OD_600_) of 0.8–1.0 was reached. Bacteria was pelleted (500× g for 10 mins.), gently resuspended in 25 ml of 10 mM MgSO_4_, before being diluted to an OD_600_ of 0.5 with sterile 10 mM MgSO_4_. A 25 µl aliquot of this solution was mixed in a 2 ml microcentrifuge tube with 25 µl of the fosmid packaging reaction (diluted in phage dilution buffer according to library titre), and incubated for 30 mins at room temperature (RT). LB (200 µl) was added to each sample and incubated for 1 h at 37°C, shaking the tube gently once every 15 minutes. CopyControl fosmid clones were selected by pelleting samples (1 min. at 10,000 rpm.) and resuspending in fresh LB medium before spreading on LB agar plates supplemented with 12.5 µg ml^−1^ chloramphenicol and incubating at 37°C for 12 h. Colonies were then picked into 384 well plates using a Q-Pix II bench top colony picker (Genetix Ltd., New Milton, Hampshire, UK). The filters were constructed using a MicroGrid II high-throughput automated microarrayer, (BioRobotics Ltd., Cambridge, UK).

### Probing the N. caerulescens genomic library

*Noccaea caerulescens* library DNA fragments were cloned into 36,864 *E-coli* EPI300^TM^-T1^R^ host cells and stored in 96×384-well microtiter plates which were arrayed evenly onto two nitrocellulose filters (48 plates per filter). Each well contained duplicated DNA fragments, whose arrangement indicated the plate of origin for DNA that hybridised to the *HMA4* probe. Filters contained approximately 5% ribosomal and 15% chloroplast contamination.

### Synthesis and radiolabelling of DNA probes

Oligonucleotide were designed to amplify 421 bp fragment in the 3′ region of the publicly available *N. caerulescens* ecotype Prayon *HMA4* cDNA sequence, GenBank accession ID AY486001.1, (http://www.ncbi.nlm.nih.gov/) using forward: 5′-GCTAGGGAATGCTTTGGATG-3′, and reverse: 5′-CTTCTCTCGCAGAAGCAACA-3′, primer sequences (MWG Biotech, Ebersberg, Germany).

DNA probes (50 ng) for library hybridisations were labelled with dCTP α-^32^P (0.4 MBq µl^−1^), by random priming using Ready-To-Go DNA Labelling Beads (-dCTP) kit (GE Healthcare, Buckinghamshire, UK) as described by the manufacturer. The labelled probe, dissolved in 50 µl of TE buffer, was separated from unincorporated nucleotides by passing through an Illustra Nick column (GE Healthcare, Buckinghamshire, UK) and heat denatured as described by the manufacturer.

### Radiolabelling and hybridisation of the HMA4 library probe

Each pair of library filters were submerged for 4 h at 55°C in 250 ml prehybridisation solution, then incubated (55°C) overnight with the radiolabelled probe, before reducing radioactivity to 15–30 counts per minute through repeated washing in solutions of 2 X SSC +0.1% SDS [49]. Hybridised filters were sealed in plastic and exposed to autoradigraphy film (Kodak X-Omat AR Film XAR-5, Sigma-Aldrich GmbH, Steinheim, Germany) at −80°C for 4–5 d. Positive hybridisations were localised and corresponding fosmids, grown and their plasmid extracted.

### Identification and ‘fingerprinting’ fosmids of interest

Fosmids containing genes of interest were confirmed initially by colony PCR using probe specific primers. Selected fosmids were then ‘fingerprinted’ through individual restriction digestion with *EcoR*I, *Hind*III and *Bam*HI (4 h at 37°C) (Promega, Southampton, UK) before running 10 µl of each digest in a 1% (w/v) agarose/TAE electrophoresis gel for 12 hrs at 0.5 V cm^−1^. Gels were blotted onto a pre-cut nylon membrane (12 h) and hybridised with the *HMA4* library probe [49]. Genomic DNA extracted from *N. caerulescens* Saint Laurent Le Minier was used as a positive control to compare all observed hybridisation patterns.

### Sequencing N. caerulescens fosmid clones of interest

Pooled fosmid pyrosequencing and shotgun library preparation using a 454 Genome Sequencer FLX (454 GS-FLX) Next Generation (NextGen) platform with standard sequencing chemistry (∼250 bp read lengths; Roche Diagnostics GmbH) was carried out by Cogenics Genome Express (Cambridge, UK) while individual fosmid shotgun libraries and GS-FLX Titanium sequencing chemistry (350–450 bp read lengths, 20-fold coverage) with gap filling by Sanger dideoxy sequencing was performed by Eurofins Genetic Services Limited (82152 Martinsried, Germany). For all, sequencing and assembly of the shotgun data was performed using a standard whole genome sequencing assembly with the 454/Roche Newbler assembler V 1.1.02.15. [28], [29]. Fosmids were extracted from bacterial suspension following the Maxiprep plasmid isolation protocol [49].

### Contig alignments of N. caerulescens fosmid sequences

Fosmid pCC1FOS™ vector sequences were isolated from *Noccaea caerulescens* inserts *in silico, via* the NCBI database Basic Local Alignment Search Tool (BLASTn) algorithm, against all available nucleotide sequences at default parameters (http://blast.ncbi.nlm.nih.gov/Blast.cgi?CMD=Web&PAGE_TYPE=BlastHome). Inserts were aligned to *A. thaliana* orthologous regions and assembled into one large contiguous sequence *via* AlignX and ContigExpress software using default gap settings (Vector NTI 11; Invitrogen, Paisley, UK). Overlapping insert regions were identified between fosmid end-sequences which aligned to identical *A. thaliana* regions and shared >99% sequence identity. Consensus sequences were assigned to assemblies of repetitive regions and poly-A and poly-T stretches that showed variation between homologous fosmid sections. All protein and nucleotide sequence comparisons and percentage identities were calculated using Dot Matrix at a stringency of 30% and window of 5 (Vector NTI 11).

### Creating promoter::GUS fusion constructs

Primers specific for the promoter regions of *Arabidopsis thaliana* (L.) Heynh. Colombia (Col-0) (*AtHMA4*), *A. halleri* (L.) O'Kane & Al-Shehbaz *ssp. halleri* (*AhHMA4*-3), and *N. caerulescens* (J.&C. Presl) F.K. Mey. Saint Laurent Le Minier (*NcHMA4*-2) were designed using Primer 3 Version 0.4.0 (http://frodo.wi.mit.edu/primer3). Promoter fragments were PCR amplified from plant DNA with Phusion® proofreading polymerase (Finnzymes, Finland) and ligated into the pCR8®/GW/TOPO® entry vector using the TA cloning system (Invitrogen, Paisley, UK.). Cloned promoter sequences were fused with *β-glucuronidase* (*GUS*) in pGWB3 Gateway-compatible destination vectors [50] *via* LR-mediated Gateway cloning technology as described by the manufacturer (Gateway® LR Clonase®; Invitrogen, Paisley, UK).

### Bacterial transformations

Presence and orientation of promoters were confirmed in all constructs through Sanger sequencing. Plasmid extractions, antibiotic selection and transformations for chemical- (*E. coli* DH5α) and electro- (*Agrobacterium tumefaciens* GV3101 [51]) competent bacterial cells were performed as described [49].

### Analysis of GUS expression in T_2_ transgenic Arabidopsis thaliana

Histochemical detection of *GUS* activity [52] was performed on T_2_ segregating transformed *Arabidopsis thaliana* Col-0 plants [53], selected on agar-based medium (10 g l^−1^ sucrose, 8 g l^−1^ agar and 2.1 g l^−1^ Murashige and Skoog (MS) basal medium (M5524, Sigma-Aldrich, Poole, UK)), supplemented with 50 µg ml^−1^ kanamycin sulphate. Seven DAS healthy, green, actively growing plants were transferred under axenic conditions to translucent polycarbonate growth boxes containing 75 ml of un-supplemented agar-based media and cultured for a further 14 days (21 DAS) at 20±2°C, under 16 h photoperiod, at 50–80 µmol photons m^−2^ s^−1^ light intensity from 58 W white halophosphate fluorescent tubes (Cooper Lighting and Security, Doncaster, UK). A randomised block design comprising three replicates was employed, with three independent transformed lines for each of the three promoter constructs and one wild type (WT) line allocated at random within each replicate (n = 30). For each replicate, all samples from each line, including WT control, were placed into individual sterile glass universals (3 plants per bottle) containing 10 ml of *GUS* assay solution [52] (5 mg of X-Gluc (5-bromo-4-chloro-3-indolyl-β-D-glucuronic acid; Melford Laboratories Ltd, Ipswich, UK) dissolved in 100 µl of dimethyl formamide (DMF), phosphate buffer (0.2 M NaH_2_PO_4_ plus 0.2 M Na_2_HPO_4_, pH 7.0), 0.5 M Na_2_EDTA, 10 mM K_3_Fe(CN)_6_, 10 mM K_4_Fe(CN)_6_.3H_2_O and 0.1% (v/v) Triton-X-100 (Sigma-Aldrich Co., Steinheim, Germany), and incubated in the dark at 37°C for 16 h.

Chlorophyll was removed from each sample to aid later imaging of *GUS* staining from the histochemical treatment. Samples were suspended in acidified methanol (2 ml conc. HCl +10 ml methanol +38 ml H_2_O) for 15 min at 50°C with intermittent gentle shaking, before decanting and re-suspending in a neutralisation solution (7% NaOH in 60% ethanol) for 15 min at RT. Solutions were discarded and retained samples were rehydrated using a series of decreasing concentrations of ethanol (from 40, 20 and 10% v/v). Once fully rehydrated in milli-Q H_2_O, samples were mounted in 50% glycerol (v/v) and viewed under a stereo microscope for traces of indigo staining to indicate *GUS* activity.

### Primers employed

Sequences of primers employed to isolate *HMA4* promoter sequences from *A. thaliana*, *A. halleri* and *N. caerulescens* were from 5′ to 3′:

***NcHMA4*** **-2 promoter_Fwd** CTCCTTCTGTAACGCCATTTCTGTA

***NcHMA4*** **-2 promoter_Rev** CTCCTTCTGTAACGCCATTTCTGTA

***AtHMA4*** **promoter_Fwd** ACTTACCGATCGGGTATGCCATG

***AtHMA4*** **promoter_Rev** TTTCTCTTCTTCTTTGTTTTGTAACGCC

***AhHMA4*** **-3 promoter_Fwd** GTGTTTGCTGGTGCTACTGTCTGA

***AhHMA4*** **-3 promoter_Rev** TTTCTCTTCTTCTTTGTTTTGTGACGCC

## Supporting Information

Figure S1 **Consensus of the genomic illustration of the fosmid B3P40.** Yellow bar represents the entire 27978 bp genomic insert. Green arrows illustrate both tandem repeats of *NcHMA4*-1 and *NcHMA4*-2 and their transcriptional direction. Blue script and lines highlight sites in the fosmid which were 100% specific for that primer. Image created through Vector NTI 11 (Invitrogen, Paisley, UK).
(TIF)

Figure S2 **Consensus of the genomic illustration of the fosmid P6P46.** Yellow bar represents the entire 31521 bp genomic insert. Green arrows illustrate both tandem repeats of *NcHMA4*-3 and *NcHMA4*-4 and their transcriptional direction. Blue script and lines highlight sites in the fosmid which were 100% specific for that primer. Image created through Vector NTI 11 (Invitrogen, Paisley, UK).
(TIF)

Figure S3 **Consensus of the genomic illustration of the fosmid J12P81.** Yellow bar represents the entire 31218 bp genomic insert. Green arrow illustrates a single copy of *NcHMA4*-2 its transcriptional direction. Brown arrows illustrate flanking genes At2g19160 and At2g19170 and their transcriptional directions. Flanking genes are labelled according to their *A. thaliana* orthologues. Blue script and lines highlight sites in the fosmid which were 100% specific for that primer. Image created through Vector NTI 11 (Invitrogen, Paisley, UK).
(TIF)

Figure S4 **Consensus of the genomic illustration of the fosmid N18P80.** Yellow bar represents the entire 20090 bp genomic insert. Green box illustrates a single copy of the 5′ end of *NcHMA4*-3. Brown arrows illustrate flanking genes At2g19060, At2g19070, At2g19080 and At2g19090 and their transcriptional directions. Flanking genes are labelled according to their *A. thaliana* orthologues. Blue script and lines highlight sites in the fosmid which were 100% specific for that primer. Image created through Vector NTI 11 (Invitrogen, Paisley, UK).
(TIF)

Figure S5 **Consensus of the genomic illustration of the fosmid H2P47.** Yellow bar represents the entire 20258 bp genomic insert. Green arrow illustrates a single copy of *NcHMA4*-4 and its transcriptional direction. Blue script and lines highlight sites in the fosmid which were 100% specific for that primer. Image created through Vector NTI 11 (Invitrogen, Paisley, UK).
(TIF)

Figure S6 ***HMA4*** **coding sequence identities.** A) Cladogram and B) Dot Matrix comparisons of coding sequences of *HMA4* orthologues from Ah: *Arabidopsis halleri*, At: *Arabidopsis thaliana* and Nc: *Noccaea caerulescens.* Tandem repeats are highlighted by “-”. “Pra” and “Her” refer to publicly available sequence data from *N. caerulescens* ecotypes Prayon and Hérault. The cladogram was created for nucleotide sequences by the DNA Sequence Parsimony Method (DNApars), and was run at default settings in Phylip version 3.68. The Dot Matrix program was run at default settings and supplied by Vector NTI 11. Numbers represent percentage sequence identities.
(TIF)

Figure S7 **HMA4 Protein sequence identities.** A) Cladogram and B) Dot Matrix comparison of protein sequences of *HMA4* orthologues from Ah: *Arabidopsis halleri*, At: *Arabidopsis thaliana* and Nc: *Noccaea caerulescens.* Tandem repeats are highlighted by “-”. “Pra” and “Her” refer to publicly available sequence data from *N. caerulescens* ecotypes Prayon and Hérault. The cladogram was created for amino acid sequences through Protpars, Protein Sequence Parsimony Method and was run at default settings and supplied by Phylip version 3.68. The Dot Matrix program was run at default settings and supplied by Vector NTI 11.
(TIF)

Figure S8 ***HMA4*** **promoter region sequence identities.** A) Cladogram and B) Dot Matrix comparisons of sequences 2000 bp upstream from the transcriptional start site of *HMA4* orthologues from Ah: *Arabidopsis halleri*, At: *Arabidopsis thaliana* and Nc: *Noccaea caerulescens.* Tandem repeats are differentiated by “-”. The cladogram was created for nucleotide sequences by the DNA Sequence Parsimony Method (DNApars), and was run at default settings in Phylip version 3.68. The Dot Matrix program was run at default settings and supplied by Vector NTI 11. Numbers represent percentage sequence identities.
(TIF)

Data S1 **Fosmid B3P40 insert sequence.**
(DOC)

Data S2 **Fosmid P6P46 insert sequence.**
(DOC)

Data S3 **Fosmid J12P81 insert sequence.**
(DOC)

Data S4 **Fosmid N18P80 insert sequence.**
(DOC)

Data S5 **Fosmid H2P47 insert sequence.**
(DOC)

Data S6 **Consensus sequence of the entire** ***NcHMA4*** **single genomic locus.**
(DOC)

Data S7 **Sequence alignment of overlapping regions of fosmids P6P46 and H2P47.**
(DOC)

Data S8 **Sequence alignment of overlapping regions of fosmids H2P47 and B3P40.**
(DOC)
