# Supplementary figures and images for: Tandem Quadruplication of HMA4 in the Zinc (Zn) and Cadmium (Cd) Hyperaccumulator Noccaea caerulescens
Source: PLoS One. 2011 Mar 10;6(3):e17814. doi: 10.1371/journal.pone.0017814 (PMC3053397; doi:10.1371/journal.pone.0017814)

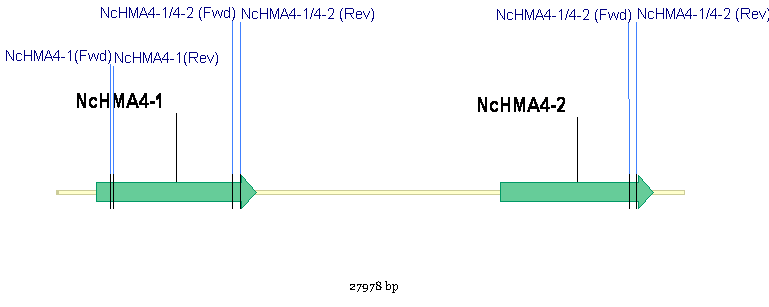

Supplement: Figure S1 — Consensus of the genomic illustration of the fosmid B3P40. Yellow bar represents the entire 27978 bp genomic insert. Green arrows illustrate both tandem repeats of NcHMA4-1 and NcHMA4-2 and their transcriptional direction. Blue script and lines highlight sites in the fosmid which were 100% specific for that primer. Image created through Vector NTI 11 (Invitrogen, Paisley, UK). (TIF) [file pone.0017814.s001.tif]

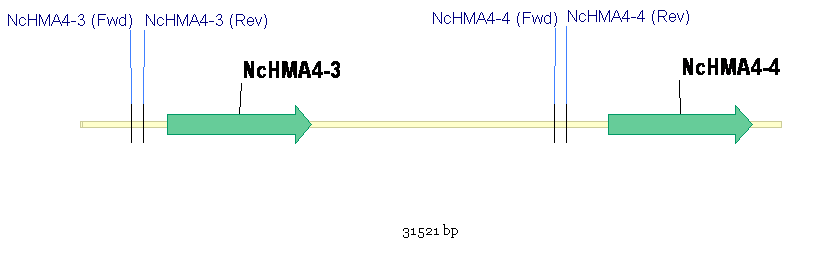

Supplement: Figure S2 — Consensus of the genomic illustration of the fosmid P6P46. Yellow bar represents the entire 31521 bp genomic insert. Green arrows illustrate both tandem repeats of NcHMA4-3 and NcHMA4-4 and their transcriptional direction. Blue script and lines highlight sites in the fosmid which were 100% specific for that primer. Image created through Vector NTI 11 (Invitrogen, Paisley, UK). (TIF) [file pone.0017814.s002.tif]

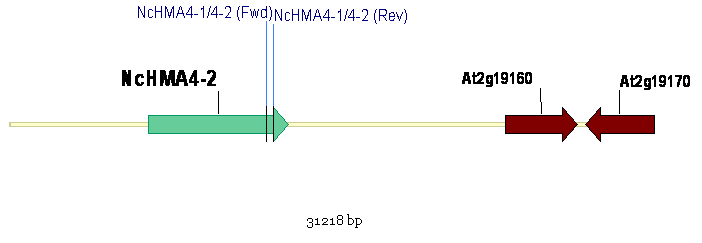

Supplement: Figure S3 — Consensus of the genomic illustration of the fosmid J12P81. Yellow bar represents the entire 31218 bp genomic insert. Green arrow illustrates a single copy of NcHMA4-2 its transcriptional direction. Brown arrows illustrate flanking genes At2g19160 and At2g19170 and their transcriptional directions. Flanking genes are labelled according to their A. thaliana orthologues. Blue script and lines highlight sites in the fosmid which were 100% specific for that primer. Image created through Vector NTI 11 (Invitrogen, Paisley, UK). (TIF) [file pone.0017814.s003.tif]

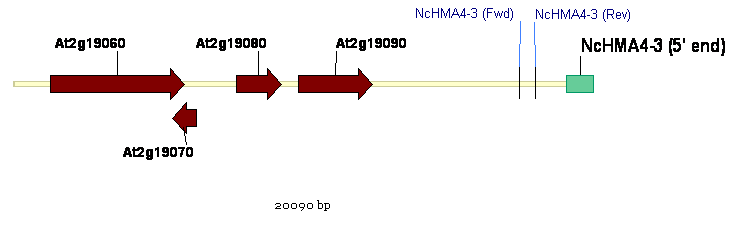

Supplement: Figure S4 — Consensus of the genomic illustration of the fosmid N18P80. Yellow bar represents the entire 20090 bp genomic insert. Green box illustrates a single copy of the 5′ end of NcHMA4-3. Brown arrows illustrate flanking genes At2g19060, At2g19070, At2g19080 and At2g19090 and their transcriptional directions. Flanking genes are labelled according to their A. thaliana orthologues. Blue script and lines highlight sites in the fosmid which were 100% specific for that primer. Image created through Vector NTI 11 (Invitrogen, Paisley, UK). (TIF) [file pone.0017814.s004.tif]

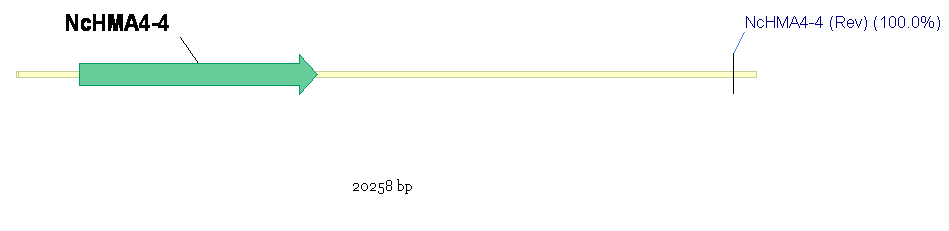

Supplement: Figure S5 — Consensus of the genomic illustration of the fosmid H2P47. Yellow bar represents the entire 20258 bp genomic insert. Green arrow illustrates a single copy of NcHMA4-4 and its transcriptional direction. Blue script and lines highlight sites in the fosmid which were 100% specific for that primer. Image created through Vector NTI 11 (Invitrogen, Paisley, UK). (TIF) [file pone.0017814.s005.tif]

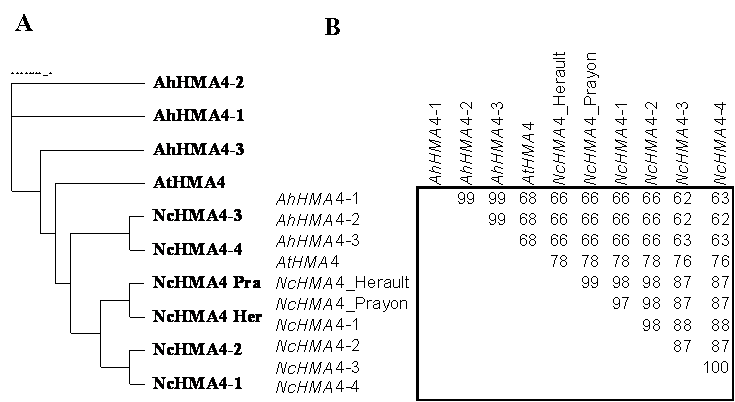

Supplement: Figure S6 — HMA4 coding sequence identities. A) Cladogram and B) Dot Matrix comparisons of coding sequences of HMA4 orthologues from Ah: Arabidopsis halleri, At: Arabidopsis thaliana and Nc: Noccaea caerulescens. Tandem repeats are highlighted by “-”. “Pra” and “Her” refer to publicly available sequence data from N. caerulescens ecotypes Prayon and Hérault. The cladogram was created for nucleotide sequences by the DNA Sequence Parsimony Method (DNApars), and was run at default settings in Phylip version 3.68. The Dot Matrix program was run at default settings and supplied by Vector NTI 11. Numbers represent percentage sequence identities. (TIF) [file pone.0017814.s006.tif]

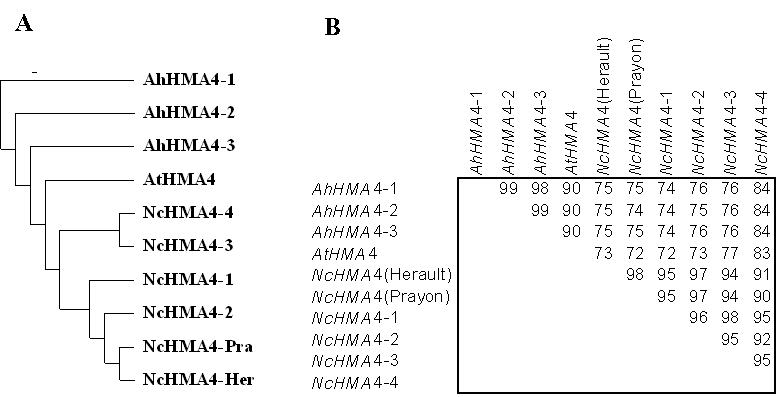

Supplement: Figure S7 — HMA4 Protein sequence identities. A) Cladogram and B) Dot Matrix comparison of protein sequences of HMA4 orthologues from Ah: Arabidopsis halleri, At: Arabidopsis thaliana and Nc: Noccaea caerulescens. Tandem repeats are highlighted by “-”. “Pra” and “Her” refer to publicly available sequence data from N. caerulescens ecotypes Prayon and Hérault. The cladogram was created for amino acid sequences through Protpars, Protein Sequence Parsimony Method and was run at default settings and supplied by Phylip version 3.68. The Dot Matrix program was run at default settings and supplied by Vector NTI 11. (TIF) [file pone.0017814.s007.tif]

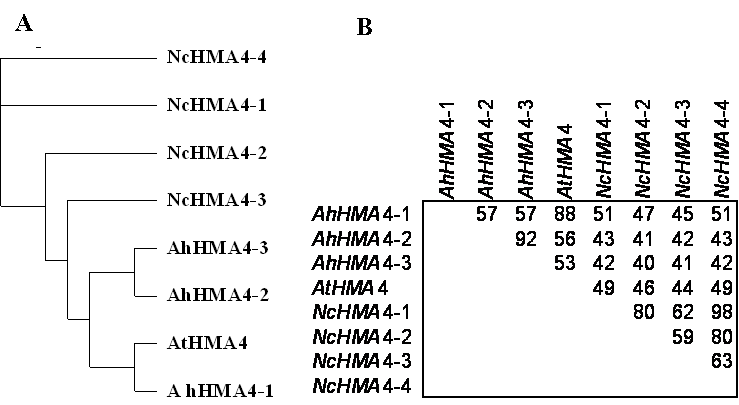

Supplement: Figure S8 — HMA4 promoter region sequence identities. A) Cladogram and B) Dot Matrix comparisons of sequences 2000 bp upstream from the transcriptional start site of HMA4 orthologues from Ah: Arabidopsis halleri, At: Arabidopsis thaliana and Nc: Noccaea caerulescens. Tandem repeats are differentiated by “-”. The cladogram was created for nucleotide sequences by the DNA Sequence Parsimony Method (DNApars), and was run at default settings in Phylip version 3.68. The Dot Matrix program was run at default settings and supplied by Vector NTI 11. Numbers represent percentage sequence identities. (TIF) [file pone.0017814.s008.tif]
