# Supplementary material for: Tandem Quadruplication of HMA4 in the Zinc (Zn) and Cadmium (Cd) Hyperaccumulator Noccaea caerulescens
Source: PLoS One. 2011 Mar 10;6(3):e17814. doi: 10.1371/journal.pone.0017814 (PMC3053397; doi:10.1371/journal.pone.0017814)
Supplement: Data S1 — Fosmid B3P40 insert sequence. (DOC) [file pone.0017814.s009.doc]

**Data S1 Fosmid B3P40 insert sequence.**

>Fosmid B3P40 27978bp

TTTTAGAATATATCACGTAGATCGTGGACAATAGGTGTGTGAACATATATATATATATATATTCAGACAT

ATAACAATAAAATATGTACACAAGCTTCGATTTTCACCTCTGATACAACAATTCATCCACATCTTTATTA

TGTCCAAACAAAAGTAATCCACGGCAATAAATCTACATAATTTGGCTCTCTTTGATTCTTCCACTCTTAC

TTTCATCTTTTTATTTCCGTAATCACAAGCAACAATGTTTCATTTTCACTTCTCGTTATCATCATATATT

ACAATTTTTTACCCATGTTAACCCTATCAAAACACCAATTAGATACATGAAAACAATTCAATCCCACACC

AAACCAGAAATCGTTTTAATTTATAGCTTCAGTTTCAGAAATTAATATACGCCAAAAACGTTGGTTACTG

TGAAACACACACCAAACCAAAAATCACCATCACAGAAATAGCCAAATTTATCCACAATTGTATAAGAAAA

AAAAAAAAAAAAGTCTTTGCGTAAGGAGAGTTATAAAAGTAAATTTCTCGTACACAAGTGTCACCAAGTG

AACATACATTAGTCAATGTTAGTATTTTAATATTCTCTTTTAGCTATATATAAGGGACCCATCTTTGTTG

AAAGAAGATGAAGTTCACAAAAAAACTTTTGCCTTCTCTCTCTATCGAATACTGTTATCCCACTTTCCTT

CCCTTTTCCTTCCCTCTCTCTTTTAATATCTCACCTTTATATATAATATTTTATAACATTAATAATTTTT

TTAATAAATCCTAATAGGGGTATGATATATAATTATATATGGTCAAAGAAGGTGGAAGAAGCGTGAGGAT

TACTGTGGAGGAAAAAGAGACATTGGAGAAAGCAACGGTCAACATTACTGTCAGATGTCGAAGGAGAGAA

AGTGAGAGAGTGTGAGACTCTGAGAGAGAGAGAAGTCAAGAAGGAGAAGAAGACAAAAGCTAATTTAAAG

CTACGAATAATTTAAAGCTACGAAGACGAGACGGGACATATATTCACCCTCGCTTTTCACATATATTTTC

GGTATTGCCACTCTCAAATTTTATTTTTTCCCTTTTTTCTTGTCTTTTTTGACCCGGCCCTGCTTATTTG

GCTATATAAGCAACTACCTTATCTAGATATCTTCACCTCGCAATCTTCCTCTCTACGTTCCAAAACCTCT

CTCACTCTCTGTCTTCACCTTTGTGGTAATACTTTAATCTCTGATCGAACCGCACCAAACAAGTCCGGTC

TTTCTTCTCGGCCTCGTCTTTTCTCCGGTATTCTTTCTCTTCTTAATTCACATAGATTTCATAACAAGTG

ATTTTTTCGTAATAATTAAAATCCGATCAAATTCACGATAGTGATATGATATATGCATATATGCATCCAA

CACGTTATATGCATCCCAGCATAAAAGTTTTGCTTTCTTATTTTTTTTTCCCTTAAAAGATTTGGAAAAT

TAGCCATTAATCCCATAATAATCTCTTTTTGCGATGTGATTTGTTTTTTTCTGTTTTAGATTTCCGTTTC

ACAGATTCGCCATTAATCCCATAATAATCTCGATTTGTTTTTTATTTTTAGATTTCCGTTTCACAGATTC

GCCATTAATCCCATAATATTCTCTTTTTATAATGCGATTTGTTTTTTCTTTTTAGATTTCCGTTTCACAG

ATTCGTTAATCATAAAAAACTTTGATACAGAAATGGCGTTACAGAAGGAGGACAAGAACAAAGAAGAAAA

TAAAATGACAAAGAAGAAGTGGCAGAAGAGTTACTTCGACGTTTTAGGAATCTGTTGTACATCGGAGATT

CCTCTGATCGAGAATATTCTCAAGTCTCTCGACGGCATTAAGGACTATACCATCATCGTTCCGTCGAGAA

CCGTGATCGTTGTCCACGACAGTCTCCTCATCTCCCCGTTCCAAATTGGTAAAGCATTAGCTAATTACTT

TCTTCGAATTTTTATTTTTACCTAATAAAAATAATTGAATCAAAAACCATAAAGTAATCTCACTTAACAC

GTAAACAATCACTTTACTTTTCTTCTCTTTCTGTTTTCTTCAAAATTAATTAATGGTTTCGCGTCCTCGT

TTGATACGCAAAGCCTCAAATTAATTTTTTTTTGGGAACTAAAATTACTCTATCTATCAGATTTACCATA

AAAGCTTACTTTGACTTTACAAAACATTTATTAGCAAAATTCGTTTATCACCAACCTATTCAAGATTTAA

GGGAAAATAGTTATCCTCAAAACTAGGGAATTCAGATTTTTGAAGTTTTTAACGATTCTACTGAAAAACA

AAAGCCCTATTATTTGGGTTTCTTCTCGAGAAAAAATAGAATATTAACCAAGTCGCCTTGGCCTAGTGGT

AATGGACTCACGGCTGTGAGCACCGCCCCCGGGTTCGAGTCGCCTTGGCCACCTAGCCGCGCCTTTAACA

GGGATAACGCGTAACCGGATGCAAGGCCTCGTGGGATTAGTCTTTAACCGGGATGCCCACGGTTATCAAA

AAAAAAAAAAAAAAAAAAAAAAAATAGAATATTGTTGTTATGGATTTTTTTTCATTTTTATTAAAATTAA

AAGAAAATTCAAAAGTTATTTATAAATCAAGTTTTTTAAAGCTATTTTGATGGATTGTTTTAGGAAAATT

GATCTAACCAACAATTGTAATTTTTTTTTTTTTTGTGTGTGATAAAGTCTACTTTTTCAACATTAAAAAC

TAGAAATTGAAATTTACGGCTTCTTTATACAATTTTGCTCGAGCCAGCATCTTTGTGTATAAAACTTTGC

ATAACTCATACATACCACATGTGACATGTCACGTGTGTACTGTGTAGCATAAACAATATCTAACTGAGTA

TTCCAAAAACATTTGCAAAAGAAAAGTGTTCAAGAAAGCCTGTTGAGTTATTTACCAGATCTTTTTTATA

ATTTTGCTAGAGCCAGCTTTTTTGTGTATAAAACTTTGCATAACTCACACATACCACATGTGACATGTCA

CGTGTGAACTGTGTAGCATAAACATAATATCTAACTGAGTATTCCAAAAACATTTGTAAAAGAAAAGTGT

TCAAAAAAGCCTGTCGAGTTGTTTACCAGATCTTTTTATCAAAATATTTTATTGGTAGTGGATCATACTC

GTTACTTAACTATATATTTATTTTTTATTTGACTGAAAACCCATTCCAGTAGTACTTTTTTTCCACTCAA

GAAAAGTATGAATTTGATGTTAAAAAAAAAAAGTATTAATTTTTAAAACAAAATTTCTTACATATTGGTT

GTTTAATCATTAACTTCCAAACAAAATTGCGGTGCAGCTAAGGCACTGAACCAAGCGAGGTTAGAAGCAA

ACGTGAAAGTAGACGGAGAAACCAGCTTCAAGAATAAATTGCCAAGCCCTTTCGCGGTGTTTTCCGGCAT

ATTCCTCCTCCTCTCCTTCTTAAAATTTGTATACCCACCTCTTCGATGGCTAGCTGTCGTGGGCGTCGCT

ACTGGTATTTATCCGATTCTTGCAAAATCCGTCGCTTCTATAAGAAGGCTTAGGGTCGACATCAACATCC

TAGTCATTATCACAGGTAATACCCACTTTTCACTTTTTATTTAATATTATTATTTTTATCCACATCACTC

ATATTGCGTGTAACTACTGTATAATGATTTGTTAGTTTACTATGTAGTATTAGTTGAGAAAGAAAATTGT

GGTTATAGTAAAACTATTCAGGCCCTATTAATAGACCTATAATGTTCTTGGAAACTTGCGAGTCTTTTAC

GCTGAATTTACCCCTTTATATGGTACTTCAGATTAGCTTACCTATATACTACTGCTTTCCTGCAACACCT

ACCACTCCACGAAACCTTTTAGAAAGTTATCCTTTACTTTTTTCTTAATATTTTTTTAAAGTATTACATA

TGGGAAAAATATCAAAACACATATTTATTAATTAATAGATGCGCAATTATTACTTTATAGAAATTCAATT

CTAGGAATGTAGCAATTTGATATTTATGTTGTATATGTTAATTGTATATTTGAGTTATAAGTTGTGGAAC

TACATAAAACTACTTTATATTTTCTTTTTATGTAAAGTACATTTGAGTAATAGCCTAATAGGATATAGAA

AAATATCAAAATGTCAATGTTTTTAAAACCGGACCAGAAGGCGAACCGGATAATCATCCGGGTCATGGTT

CAATTTGGTTCGACCGGGTTGAATTCGGTTCATAATAATTTATGTTTATTTATTTTTAAATATAGAACTT

TTATTTTTCAAAGTTCCCAAGTGTAAACACATACATAGAATAATTATTGTGATTTTACATAATTCTCTTA

TGGAAATATAATAATTCTTTTTTAACATGTAGTTTAAAAAGATAAATCTTTTACGTACACACAACATAGA

TATATAGATTTTATATATAACTATCGAGGCAACTAGGAAAATGGAAGTTTCATGATCGAGAGTTGTGTGG

TTCTTTGGGAAAACTTAATTTTTTTGGTTATTTTATACGAAAGTAAAGGATTCGTTTGATTCTTGCTCAG

TTTATTATTATTATTTTTTTTAAAAAAGCTGCAGTTACGTCCCATAGAAGAAAAAAAGGTTAACTCGTAT

TTGATTGGCTTATCTTCTACGACTCAAAATGGGAAAAACTCAAAAAAGCAAAGCAAACTTTTTAGTTTTA

AGTTTTAACTCGTGAAAAGAAAATTAAAAAGAGCAACAAATAATTGAAAGAACAAAAGCATCAAAAGTAA

AGAAATTAATTCATAATTCATAGACTGATAACGGAGTTACTTTTAGTTGAAATTTCGGTTTAGGACACCA

AGCTTGTGAATCCATAATATAAAATATTTTTTTAAAATCTTGATCTTTTGTTCGTTTGTATGATGTAATA

GTCACTTCAACAAAACTATAACTCACTAATATTCCAATTTCATCAAACAGTGGCTGCAACACTTGCAATG

CAAGATTACATGGAGGCTGCAGCAGTTGTCTTCTTATTCACCATAGCTGACTGGCTGGAAACAAGAGCTA

GCTACAAGGTATGTTAACTAGTAATCATCATATATTGTGTTAATCAAACTACTATGGATTATCTGAAGTT

GAAATTGTAATGGATTATTGATTATGGCAATTGCAATCCCAGGCGAGCTCGGTGATGCAGTCTCTGATGA

GCTTAGCTCCACAAAAGGCAGTCATAGCAGAGACTGGAGAAGAAGTTGAAGTAGATGAGGTTGAGCTCAA

CACAATCATAGCAGTTAAAGCCGGTGAAACCATACCTATTGATGGAATTGTAGTCGATGGAAACTGTGAA

GTAGACGAGAAAACCTTAACTGGTGAAGCATTTCCTGTGCCTAAACAGAGAGATTCTACGGTTTGGGCTG

GAACTATTAATCTAAATGGTAATGTAACCCTCTTACACAAGCTTCAATCTTAGAAAAGTTTCAAGCTTTA

ACCTTTTTGTTTTGGCAGGTTATATAAGTGTGAACACAACTGCTTTAGCTAGTGATTGTGTGGTTGCAAA

GATGGCTAAGCTCGTAGAAGAAGCTCAGAGCAGTAAAACCAAATCTCAGAGACTAATAGACAAATATTCT

CAGTACTATACTCCAGGTTTGCAAAAAAACATAAACCATAACTTGTTTTCTTTATGTTCTTGATTCTTGT

AATTTGAGACCTCTCTGTTTTTTGTTTGTTTCAGCAATCATCATAATATCGGCTGGCTTTGCAATTGTCC

CGGCTATAATGAAAGTTCGCAACCTCAACCATTGGTTTCATTTAGCACTGGTTGTGTTAGTCAGTGCTTG

TCCCTGTGGTCTTATCCTCTCTACACCAGTAGCTACATTCTGTGCACTTACTAAAGCGGCAACTTCAGGG

CTTCTGATCAAAAGTGCTGATTATCTTGACACTCTTTCAAAGATCAAGATCGCTGCTTTTGACAAAACCG

GAACTATCACTAGAGGAGAGTTCATTGTCATAGAATTCAAGTCACTCTCTAGAGACATAAGCCTAAGCAG

CTTGCTTTACTGGTAATAAAAACAATATCTTGTTCTAACCAAAAACTAGTTTGATGGGATAACGTATGAA

TGACAATTTCTTGTTTGGTTCTCAGGGTATCAAGTGTTGAAAGCAAATCAAGTCATCCAATGGCAGCAAC

GATTGTGGACTATGCTAAATCTGTTTCTGTTGAGCCTAGGAGTGAAGAGGTTGAGGATTATCAGAACTTT

CCTGGTGAAGGAATCTATGGGAAGATTGATGGGAACAATGTTTACATTGGGAACAAAAGGATTGCTTCAC

GAGCTGGTTGTTCAACAGGTAAAGCTTCAAACTTTGGCCAAGAAAAAACTCAATGGAATGGTTTTGTTGA

GCCTTTGATCATTTTGAAACTGTTCTTTCTTGACAGTTCCAGAGATTGATGTTGATACCAAAAAAGGAAA

GACTGTCGGATACGTCTATGTAGGTGAAAGATTAGCTGGAGTTTTCAATCTTTCCGATGCTTGTAGATCC

GGAGTAGCTCAAGCAATGAAGGAACTCAAAGATCTTGGAATCAAAACCGCAATGCTAACAGGAGATAATA

AAGATTCAGCAATGCATGCTCAAGAACAGGTATGAGACTGAAAAAACCAAGAATTTTTCATTACTCTCCT

AACGTTAAGAGATTATATTAAAACTTTGACATGTTCTTATATGGAACAGCTAGGGAATGCTTTGGATGTT

GTTCATGGAGAGCTTCTTCCTGAAGACAAATCCAAAATCATACAAGAGTTTAAGAAAGAAGGACCAACTT

GTATGGTAGGAGATGGTGTGAATGATGCACCAGCTTTAGCTAATGCTGATATTGGTATCTCCATGGGGAT

TTCTGGCTCTGCGCTCGCGACGCAGTCTGGTCATATCATTCTCATGTCAAATGATATCAGAAGGATACCA

AAAGCGATAAAGCTAGCAAGAAGAGCTCAGCGGAAAGTTCTTGAAAACGTGTTCATCTCCATCACTTTGA

AAGTAGGGATACTGGTTTTAGCATTTGCTGGTCATCCTTTGATTTGGGCTGCGGTGCTTACTGATGTAGG

GACTTGCCTGATTGTGATTTTTAACAGTATGTTGCTTCTGCGAGAGAAGGATAAATCTAAGAACAAGAAG

TGTTACAGGGCTTCTACATCTGTGTTGAATGGTAAGAAACTTGAAGGCGATGATGAAGAAGGTCTTGACT

TAGAAGCAGGGTTGGTATCAAAGAGTCAATGCAACTCAGGATGTTGTGGTGATAAGAAAAGCCAAGAGAA

GGTGATGTTGATGAGACCAGCTAGTAAAACCAGTACTGACCATCTTCACTCTGGTTGTTGTGGTGAAAAG

AAGCAAGAGAGTGTAAAGCTTGTGAAAGATAGCTGTTGCGGTGAGAAAAGTAGGAAACAAGAGGGAGATA

TGGCTTCACTGAGCTCATGCAAGAAGTCTGACAATGACCTGAAAATGAAAGGTGGTTCAAGTTGTTGTGC

TAGTAAAAATGAGAAGCTGAAGGAAGTAGCAGTAGCAAAGACCTGCTGTGAAGACAAGGAGAAAGCAGAG

GGAAATGTTGAGATGCAGATTCTTGATTTGGAGAAAGGGTCGCAGAAAAAGGTTGGTGAAACCTGCAAAT

CAAGCTGTTGTGGAGATAAAGAGAAGGCTAAGGAAACACGTTTGTTGCTGGCTAGTGAAGATCCATCTTA

TCTGGAGAAGGAAGAAAGGCAAACTACTGAAGCTAACATTGTGACAGTGAAACAGAGCTGCCATGAGAAG

GCAAGTCTGGACATTGAAACTGGAGTTACTTGTGATCTCAAGTTGGTCTGCTGTGGAGACATAGAAGTGG

GAGAGCAATCTGATCTTGAGAAAGGCATGAAGTTAAAGGGTGAAGGACAATGCAAGTCTGACTGCTGCGG

TGATGAAATACCTCTAACTTCTGAGGAAGACAGTGTGGATTGCTCCTCCGGATGCTGCGGAAACAAGGAG

GAATTAACACAAATCTGTCATGAGAAGGCATGTCTGGACATTGTAAGTTGTGATTCCAAGTTGGTTTGTT

GTGGAGAAACAGAAGTGGAAGTGAGAGAGCAATGTGATCTCAAGAAGGGTCTGCAGATAAAGAATGAAGG

ACAATGCAAGTCTGTTTGTTGCGGTGATGAAAAGAAAACAGAGGAGATAACTGAAGAGACGGACAATCTG

AAAAGTGAAAGTGGTGATGATTGCAAATCTCTTTGTTGTGGAACTGGTTTGAAGCAAGAAGGGTCTTCTA

GTTTGGTCAATGTTGTGGTGGAGAGTGGTGAATCCGGGTCAAGCTGTTGCAGCAAGGAGGGAGAGATAGT

GAAAGTCTCTAGCCAAAGCTGTTGCACAAGTCCAAGTGATGTGGTGTTATCTGACTTTCAAGCTAAGAAA

CTAGAGATTTGTTGCAAAGTGAAGAAGACTCCAGAGGAGGTTTGTGGATCTAAATGTAAGGCAACAGAGA

AGCCTCACCACGTCGGTAAAAGCTGTTGCAGGAGTTATGCAAAAGAGTATTGCAGCCACAGGCATCACGA

CAACCATCATCACCACCATGTTGGGGCTGCTTGACGGAGATAGTGATTGATTACCTTTAAACTCTCGACC

CATCCATCTATTTGCATAACCTTTCCTTCTTCAACCAATGTCGCCCAGAAAAAAAATAAAAACTTATTTA

GTGTTTCCAGCAAAGGTGTGATTCGTAAAGACAATGCTAGTGATCGTTGTTAGTCTTTTATGTTTGCCAA

AACCCTAATGTATTTCTCCTTTTCTTGTTTTTATTCGCTTCTTGAAGATGCCCAGAAGAAGTTTGAACTT

TGATCCTAGAGTCTTAAAATCAAATAGAACAAGCAGTTGAAACATAACTTCAACTTAGGCTTGGAGTCTT

TTTGTATGCGGTGTACTACATAAGCTTTCTTGACTGACACGTTTCTTGTCAATTCTAGGGCATTACTATA

TTAGGACAGAGAAGGTGTTGCAGTTCGTGTCCTGGAGAGTTTAGGTGAAAAAAAATAAAGAGCAAAAACC

GACTGTATGAAAAAACACTCGCACATCCATGTAATCAAGAATCAGTAAAAATAAAAATTAATCAAAGGGT

GTCACAACTCACTATATAAACAAAGGTCGTCACTCTTGAAAACAATCTGAAATATTCCGCAATCGCTGAA

AGCAGAGCATTAGAGGCAAAACCCTAGCTATTCTTTTGTTCTCGTCTTTGTTTCTTATCTTTCATCTCGT

CAAGCTTGTCGATGAGGTGAAAATTTAAAATCTATTGTTCACTTCTTCAAGTTTAATAAGACATATATCC

TAAGTTCCACAAACTTTTGTATCTCAAGATAAAACTCGTGACACATCTGATTCAACACGAAACCTAATAT

TTTTAAAGATCTACTAGGTAGGATACTTGCGCTTCGCCGCGGAAGACTTTTTTTGTATCTTTCCCGTGTT

ATAAATGATTAAATTATACATTTTATATTTTGATATTTTAATCCAATTTTCCTTATATTTCCTCTGTTTC

ATATTAAAATGTCGTTTAAGATTTTTTCACACATATTAAGAAAATATTAAAATTTTCTGTTTTACTCATA

ATTAATCTTCTCAAGGAGAAATGTGTAAAATCTTAGACGAATTCTCGTAGAAGGAAAGGAAGAATCAAGA

TACCGATTAGAGTCCATCATCATAGAAGAAGATGAACAGTCATGAGTTTGATCTCCATCAATTTCAGAGA

AAACTGCTTCCTCTTTCTCCTCCATGTTGTTCTTCTTCTTCCTCCATTCTCTTCTGCTCCGACGCGGAAT

CGGTTTAAGACTTTTGAAGAAACACAGTTTCTAAACGCTCGATTTACGTTTTCCGGTTAAATGTTTGACT

AGTAAATGAAAACAGGATAAAATTAAAAACCGAAAACCAGGATAAATGTTAAATGTTTTCAGGATAAATG

TTTGACTAGTAAATCAATAAATGAAAACAGGATAAAATTAAACCGAAAAAATTGCTAACTAGTATTAGAT

GTTTGTTTTAAATAACGTGTCAGCTTCAAAAAGGAGATTACCGTGAGAAGCACATGAGATTTTTCTGTGT

TGATTAAGTACAATATCTTTACAAAGAAAAAAAAAAAATTCCTCCAATGACACATGTAAAAATTAACTCC

AAATAATGCAACTTCATTTTTACCCCAGAATTAATGGTTGATTCCACCAATTTCATTATAATAGCATTAC

CATTTAGGTCTCTGGTGATGTGTATGTTTGTACCACCATTCATTAGTTATATAACATATATAATGCTAAT

GTTGGGTGTTGATAGTATGTCCACTATATAACTTCTTCGTGTTTTTCACTGTGTACATATCATGATAAGA

AACTTGTCTCTAGACCTTGTCCCCATTTTAATATTATACACTTATTCAAAATCTTTATATATATAGATTT

TGAAAGATTTGCTTGGAATAGAAAATATCGCTATGCTTAAGTATCAACTAGACAACTTGTAACAGTTGAG

AATCGTAAACTATATGTTGGCATAGAAAATGGCGTTTTCTATGTGAGTTGGCTGGTTGATTTATATATTT

ACAATCTCTAGTTCAGTATTCAGATGATGTCAAAAAAACGAAAATAAGGTTAAATTATGGTTTCACGGTA

GAAAAGAGTTATTGGGAGATGAATAAGTCAAAAGAATTGTTAAATTTATAATGAGTTTTTGTTAGATTTG

AAAAGAGTATGTAAATTTAGGAAATCAATCAAAATATTTTATTCTAAAGGATTTTGAAAATCTAAGGGTT

ATTGATAGTATTTTGAAAGTTTTGTTTCATGAGTTAATTTGTTTAAAATCCATCTCCCAATAACAAGGAT

TTTAATGGATTCATGAAATCATTAAAACACAAACTTTTTGAATAACAATGGATTCTGTATGGAATTTAAA

AATCATCAAACCAATAACAATGGATTTTAGTAAGATTATCAGAATTCATAAACCAATAACAACGGATTCT

TAAAATTTTAAAAATTCTTTGAAATCCATCTCCCAATAACCCCTAAATACCAAAGTGGCTATACGTCCAA

ACTCCGGAGTATATAACTTACGAAAATTTCAATATGAATTACAAAAACAGTTCTAAGAAATCTGAATACA

CGTCTTAAGCAGTATGTTAGAATTTTAGATGTACTTAGCCGACATTTTTTCAGTTTTCGATGTGAATTTA

GGACTGGTTATCGCTCATCAGGACTAGACATGACCAAAATCAAACCGTAACCGTATTTCGGACCGTAACC

GGACCGTTTTGTAACCGTAACCAGACCGTAACCATATTAAACAGTTAAAACCGTAACCGTTAACCGTTAA

AATATATTAGTTATGGTTACAAAAATTATTAACCGTAATCATTTTTTTAACCGTAACTATATTAAAACCG

TTGGTTAACCGCAAGGTTAAACCGTATAATTTTAACCGTAACCGTTTTAAAAGTAATTTAAATATAACAT

TAATTAGTTATATACAAATAATTTGACATAGAGAAGTTATGTTGTACAATCAACTTATCAAATCCAAACC

TAAATTATTTAGAAGAATATATAACTATAAATTTAATGTGAATGTGCAAAAATCAACTGATATTTTTCTA

ACAAATAAAACCTTTTTTTTGCCGTAACTAATTTTTAATAATAATAATAATAATTTTATAATAAATTTAG

GTTAAAAAAAAACTAGATCGTGCTAGATACAATCATACAACTAAATAAAATTATAATTATATAAATTACA

ATCTCATCATCCAACTATATATCATTATCTAGTATTCTAATTTATAAATATGTATGTATAATTATATGAT

ATTTATATATCTGAAATAATTATATTATACGTACATGAAATATATTTCTTTTCATTTTATATAAAAATAT

TTAATTACAAATCATTAATTAAATATAACCGTGTTTAACCATAACCGTGTTAACCGTAACCGTTCTAACC

GTAACCGTTTTAACCGTATATTAAATGGTTAAGGTTAAGGTTAAAAAAAAATTATAACCGTAACCGGAGG

TTAAATAACCTTAACCGTGATAACCGTAACCGTGGTCATGCCTAATCAGGACAAAGGTGGATCTCACAGG

TTACTACTTTGCATCAATTCTATCATATAACTCAAAGGTCAGAATAGGTTTGGGCATTTTTACCCGATCC

GAAATACTGAACCGAACTGAAGCGGGACACAAATATTCGAATGGGTCATAAATTCTTATACCTAAAAGAA

TAGGACTTGAACCAAAACTAAACCGATAACCGAAAGAGTACCTAAAATATTCAAAATATAATTATATACC

CAAAAATATTAGTTATATTATATTTAGACTTAAAATAATTAAAATATGTAAAATTACAATTCTAAACTTA

ATATAATAGTTAAATTTAGAAAAAATAACCAAAATATTCAATAAATCCAAAAATCTTTAGTTATATTTGT

TAATATTTAATATTTTGTTGTTAAAACAACATTTCATTTAGAATTAAAGTTTATCCAATTTTTTATTTTT

ATTTTATTTTTTATAATTAAAGTTTGGATACACCGAACCGATCCGAATCCGGGCAGAACCGAATCGGACC

CGACCCAAAAATAAAAAAATATTTTAATGGTTCTAAAATTTCTAGAACGAAAGAATTAAAAAAAACTGAC

CCGAATCCGATCCGAAAAACCGAATGCCCAGGCCTAGGTCAGAATATATCATGCCCTCGATCGTAGAACG

ATATGCTTTTCTTTGATAAAGATACGATTATGATAATATTTAACGAATTAAGTATACTATTAAGCTTGAC

GCCTTGACCACCTCACTGATAATTTTGTTGCATTGCGCATTTGCACTTTCATAACCATTTTTACAACTTT

TCTCAAAATATTGGATATCGATAAATAAATTAAAGGTATACATTAGAAATATTCTAAGTCGTGTTTATGT

TTATTTGAATGCATATCGCGATAACATATCCAACAATATTTTTCTCAAGATTCGTGTATTAGTTTATACA

ATAATTTTTTTAAATAAATTAGACCGATCAGGAGGCCTCAGCCACACCTAGCTCGGCTTCTAGGAATCCA

TTGGTTATAGTCTTATAGAATTAACAATTTCTCATTGTAAAATTATAATCTGGTTTATTGAGATATGTAA

AGGTTAATGGTCAGGAGGCATTAATAATTGTCTACTCACATTATTTAGAAGATTCAACGACTCCAAACTA

TTCTTGATAGTACAGTTGTTAAATAATTGGAGTACATGTTGGTCTTTGGTACGACTCTTGCTTGCATTGA

AATCGGTCATAGCCATAGATTAACGAGTCATAAATGCGAGGGTGACATTTTTCCTTTAAGCCGCCAAACA

TTCACTTTTTATTACAGCCAATTAAACTGAACGGTTCTGGTGGTTAAGGTGAAAGTGTTATAATTTCAGT

TCAGTTTTACAATAACGCAATTACCGAAATACTTGATTTTGAGATATTGACATGGATTTATATTATTAAT

TTAAAGAAAGACAAAATTTAGATGGGACACTTCACCCCTGCTTAAGCTTCACTACGACCACTAAAATCTT

ATAACGAAGTTTTAGAGATTTACTACTTGCTTTATAAATATGGATTTCGAATCACATTTAGACAATCACT

TAGATTGGTAATTTTTAACTGAACGGTTTGGTATCAGTACATTTTCAAAATTATTAATCAGAAATGCCTA

TGTTCAAGATTTCGCTAATCGCTAACCAGGCGGTTGGTCACCGATTAGCGATTTTTCAAAAATCGGTGAT

AAATCGAGGATTAATCGGGGTAGAATTTTTACTATATTTTAATATATTTTAAAAATTATATATATAAAAC

ATTAATACCAAAATCATAAACTCATACAAAAGATTATATTAAGCATTTTTATCAATTCACATATAATAAC

AAAGGTAAAAATAGTCTTGATCATATAGAGAACATACAAAAAAATAACTAAAGGATGGTAATTTAATAAT

TTTTCATCTTTATATTCCTCAATGATCAAATTGTCCATAAATGTGGACTAAAAATGGTAATTTCCTAATT

GTTCATCTTATTTTGGGTCCGTTTCATTTTTTTTTAACACATGGCAAAGCAAAATAAAGTAAATGGGCTT

CAATTTTTTAATGGCCCCAAAAAATTTCTGATTAATCGGTCAATTTTTTGCAATTAATTGGTCAAACCAG

TCAAACCACGGTTGACCGGTTTGTGATAACGATTAGGGGAAATCGACTCGGTCAGCACCCGATTAGCGAT

TAAACGGCCGATTAATCGTTAAATCGGCCGGTTTTTTGAACAGAGAGAAATGCTAATGTTAAACGAAAAT

ATTCATAGTGATGGTTGACTGACGAGCAGAGGTGGGCTCCAGCATAAAATAGCTGTGCATTTTAGCTTTT

GGGAAGAGAATGCCGTTCCTTACGGTACCGGTCTCAAATTTTCCAAATTTTGTGAAAATGAGAAGTAGCA

ATTTCCTGTTTGGGCTTATTTCCTTTATTTTAGCTAGTAAGGAAAGGGTACATCATAGGTATGGGAAATA

AAATCCAAATTACCCGTTTAAAAATTATAAATAAAAATAAAAATTATTAATATTAATATTTTTAAAAAAA

ATTTTGAATATTTCTCATATTTATCAAATTTCCCATATTTTTCAATAATTTCTCAAAATCTTTTCAATTT

TTCCGATTAAGTAAGATTTTGATATTTGAGTATGTAATTTTTAGTCCCAAACTTCTCATTTCCTTCATTT

TCCCCTTATTTTCCATGCCATTATCATAAAAGAATACTTCAGATAATATATTTTGATTCCCGAATTTTTC

ATTGTCGTCCGATTCCCGTCAATACCTGTCACAAAACCCAAAATGTACAGGTTATGAACAAATGTACTTT

CTAATTCTACCTAATCTAGTGAATATGTCAAAATTGGAAAAATGAATATAATAAACTATATGCCAAAAAA

GGTTCATAATTTTTTTTATTAGCATAAACATAATTAGGATCTTAAAATTTTTTTATCTTTAGTAAATTAG

CTCTTCTGGTTAAAGATATCAAAAGGAGTTGTAAATATATGATTATTTTTAGAAATGTGCTATTTTGTAT

GTTTAGAAATGGTTTGTACTCTGAACATGCATACTTTCGTAAAAAGTAACATAATAACACATAATAAACA

GTAAAAAGTAACTTAAACTATTTTGGACTATACGAGGTGGATCCATAACATACATCGCACCTCTAGATTT

CTACATCTCCCTTAACTTCTTACTCATCACCGTGATCGACACAGTTCGACATAACACTTTGACAATTATA

CATTTTTTCCACAAACCGTTCATGGCCACCAACACTTCCTCCCCAATCGTTATTACTGGTTCTTCATCCT

CACCATTCGGAAATTCCAAAGGAAGTCTCGCTTCTACAGAGGCCTCATCCACCACCGTCTCCGGTACCGG

TCTCCCACCCATACTACTCCCCGTCACCCTCTGCGCCCATGAGCCTGTTAAATCTGGTGGTCGCCCCTTC

TCCCCAACGTCCTCCATGTGGGCATCGATGACCCTGCCAATCTCGACCCCCGTTTTCTCAATCGCCTCAC

TCGCCGCACTCATTAGGTGTTTTTTTAAGAATTTTACTAGTGTGTGCCACTTAGCAATAAACATACTACT

AGACTTATTTAAGTAGTAATGTCACAATTCTAGGCAAGATTTATACTTTTGTTTGAAATAGCTTATTTAT

CTTTAAACATGATTTCGTAGATTGGTAGACCTCGGTACGGAATTTGGCATTGATAAATTGATTGGGGATG

ACTCTAGTAGTCTCGGTAAACATCTAAAGCTTTCCATTAGTTGCTAAAATGTGGGTAATATGTACCAGTA

TCACATAATATATAATTTTTCACGTCTCGGACGAGGATTGTTTTCAATAGGGGTCAAAAATAGGTGAAGG

GTCAAAGCGGGGAATCGAACTTGTGGGTCAGAGGTTTCAATTAGCATATTTTACCAATTTTCCAAGTGAA

TCTTATGGCATTTTAGCTACAATTTCTGGTTTTATAAAATGAATAGGGTGTCACTTAACATCGTATTCTT

CTAAGTCGACGCCACTGTCTCGGACGCGGTACGGTTTGAATTAAACAAGGTTAATTTTTAAGATAAGTTA

CAAAGAAAAGATCAAACATAATTAAAAAATTTGGGAAGCCAACAGATTAAAGATAATTAATGCTATTCCA

ATGTTGAGTCGCAAATTTAAGTTCTAATTAAGGAGAGAATTCACTTTTTATAGAACTGCCGCAATTTTTT

TTTTATCTTTCTACCTGATTATTCAAGCACCGAGCATAAGTTATGATCTTGTGCAAACATGTTACTAATT

TAATAATATGTATTAAAGATATAGATACATCTTAGAAGAAAAGCTAAGAGAGTAGACGACAAGTGCATTG

CGTAAAAAAAATGTGTAGTTTAATTAGAATTTTAGAAATAAACTAAGAAAATTGTACTAAAAACCAAATA

AAGAAAGCGATTAGATGAGGAATCACACATGGATTCCATTTTGTGACATTACACTATTGGTGTTTTCCAC

TAACATTTTATTATTTTAGTAACTTTGACTTCGTATCTCTCACTCACGAGATTAAATCCCTCTTTGATCA

AATTTTCTGCTCAATTCTTTCTTTAGAGAACTAGCAAGAATCATGATTATAATAATTCCAATTCTTAGTA

TGCAATATTGCGAGGATCATGTGTCTAAACTAGCGACGTATCGGACAAGTTTTATCCTCGCCCCATATTC

AAACTGATAATGTTTTATAATCTCACTTTTCTTTTGTAACCATTTTATATAAAGTGTTAATAGATATATA

CCATATTTTTATCCCAAAAACTTAGAATATGTAGTTGTTTTGATAAAACTCTAATTGATCATCTACTCCA

TAAAAAGCTAATTTCGAAATTTATAAAACAAAGTCACATGCACAAACAACTTATCTTGTGATTAAGGATG

TTTTTACTTATGACTCAGCTAGGTTCAAATCTCAAAAACATAGCCAATTCAAATTTTATGAAGTTCCGTA

CTACTGTAAAATGAGCCATCAATCGTTTTTAAAAAGGAGTTAGATTAGACTAGTCTATAATCCATTATAG

TGAAAACTGCTACACAAAATATCATACTTTTATATAGTGCTAATGTAATCGATTTTAAAATAAACTTACA

GTTTTATATTCTTGGAAATTACTGAAAACAATAAGAAATTACATCTTGATAGGAACTAGGTTGAAAATTC

GGAAGGAATATAGGAATTCGAAACAAAGATTAAAATATCCTACGAAATTAACATAGTAAAAAAAAAACTA

AACCAAATAAAAAAAGTTTAACGTAAAAAGAAAAGTTTTAATTCAAAGAATCCAAACTAACCACAACTTT

TGAAACATAATCCATAAATGTTAGGTTTAGAACGGCTTTTACAGATTTAGTAACTATTCTCAAATCATTT

AGTACGTCTTTCTTAAACTTCTAAGAACCAAATTTGTGGTATATATTTGATATATTTGAGTTATCTTTTT

CGTTCGACTTTGATGTAATTGAATTTTTTGGGGGATTTCGGGAAAGCAAAATATACATTTCGGTACTGTT

AGAACGAGAAAATTACATCAATAAGGACTTTTTGACTTATCTTATTACAGCAAGAAGGACAGTGTAACTA

TGGTGCACTTTGCCTTAACAAAAATACATTGTTGCTCTTTTTATTTGTTTTACCTTTACACAATTTAACT

TTATATTTTACCAGTTACACAATTTATCTCTCTCCACGATAAATCCTTCTCTCTCTTCTTTTCTTTCTCC

ACTTTATTCATCTCCACTTTCCTTATCTCTTTGCTTCCTACAAATTCTCTGTCTTTCTCATTTTCCACTA

GGTTCTTCTTCCACCAATAAAGATTGGGACAAGGGTTGCGTAAAGACATTATTATGCCTTGAATAAGTGT

TTGTGTATGCATGCCCTTTTTACAAAAAAAAAAAAAAGAATAAGTGTTTGTGAACAAAATATCTTGGATA

GAATTTTAGAATATGATTGACAAAAAAAAAAAAGAATTTTAGAATATATCACGTAGATCGTGGACAATAT

GCCATGGACAATATGGGTGTGTGAACATATATATATTCAGACATATAACAATAAAATATGTACACAAGCT

TCGATTTTCACCTCTGATACACAATTCATCCACATCTTTATTATGTCCAAACAAAAGTAATCCACGGCAA

TTAATCTACATAATTTGGCTTTCTCTTTGATTCTTCCACTCTTACTTTCATCTTTTTATTTCCGTAATCA

CAAGCAACAATGTTTCATTTTCACTTCTCGTTATCATCATATATTACAATTTTCTACCCATGTTAACCCT

ATTAAAACACCAATTGGATACATGAAAACAATTCAATCCCACACCAAACCAGAAATCGTTTTAATTTCTA

GCTTCAGTTTCAGAAACCAATTTACGCCAAAAACGTTGTTTACTGTGAAACACACACCAAACCAAAAATC

ACCATCACAAAAATAGCCAAATTCATCCATAATTGTATAAGTAACCGTACAAACGTATATAACAAAGCGT

ACACATGTTTTCAATTGTCCAAAAACACATTTTACTACAATTAAACGAAGCTATCAGATCCATGTCCTCT

CCCAGAAAAGAAAATAAACACTCGTCAAGATAACCATATTATTTATGAATAAGTAAAATTCATGCGATGC

AATTTCCTCCAGCCGTATCAGTCTCTACGTGGTGCGGTCGCCGGAGTTAAATTAAAAGACACCAACCAAA

ATAATCTGATTTTTCACCCCTTTTTCATTAAAGGTAAAATGGAACTCTCAACACACTATAAGCAAAAAAG

AAAAAAAGTCCACCACTTTCACAATTTCCCTCTTATAAAAGTCACTCTTAGTGTAAATGACAAATGACTC

CTGTTAGAACTACCTAAAAGCAGGTTATGGAATTCGTTGGTCAAATGAATTAAAAAAAAAAAAACAAATT

ATAAATTGTTATGAAAGAAAATATCTCGTACACAAGTGTCACCAAGTATAAGGGACCCATCTTTGTTGAA

AGAAGATGAAGTTAACAAAAAAACTTTTGCCTTCTCTCTCTCTCCATCGAATACTACTATTATCCCACTT

TCCTTCCCTCTCTCTTTCGAAAATGTTAAAGAACAAAAACCAATCATCTAACATTCTGACTTATCTACAG

GGGCGGCTTATTCGAGTGGGGTCAATAGATGCTCTGCACTAGGTGACGTAGAAAAACAAAATTTTAGTAG

AAAAAAAGTTCACTTAAGCCGGCTATATAAAGCAACTACCATTTCTAGATATCTTCACCTCACAATCTTC

CTCTCTACGTTCTAAAACCTCTCTCACTCTCAGTCTTCACCTTTGTGGTAATACTTTAATCTGATCGAAC

CGCACCAAACAAGTCCGGTCTTTCTTCTCGGCCTCGTCTTTTCTCCGGTATTCTTTCTCTTCTTAATTCA

CATAGATTTCATAACAAGTGATTTCTTCGTAATAATTAATATCCGATCAAATTCACGATAGTGATATCTC

CAACACGTTATATGCATGATGCATCCCAGCATAAAAGTTTTGCTTTCTTAATTTTTTTTCCCTTAAAAGA

TTGGAAATGGCTGCCATTAATCCCATAATAATCTCTTTTTGCGATGTGATTTATTTTTTTCTTTTTAGAT

TTCCGTTTCACAGATTCGTTAATCATAAAAAACTTTGATACAGAAATGGCGTTACAGAAGGAGATCAAGA

ACAAAGAAGAAGATAAAAAGACAAAGAAGAAGTGGCAGAAGAGTTACTTCGACGTTTTGGGAATCTGTTG

TACATCGGAGATTCCTGTGATCGAGAATATTCTCAAGTCTCTCGACGGCGTTAAGGAATATACCGTCATC

GTTCCGTCGAGAACCGTGATCGTTGTCCACGACAGTCTCCTCATCTCCCCGTTCCAAATTGGTAAGCATT

AGCTAATCACTTTATTCGAATTTTATATCATTTTTATTTTTACTTAATAAAAATAATAGAATAAAAAAGC

ATAAAGTAATCTCACTTAACACGTAAACAATCACTTTACTTTTCTTCTCTTTCTGTTTTCTTTAAAATTA

ATTAATGGTTTCGCGTCCTCGTTTGATACGCAAAGCCTCAAACGTTACTTTTTGGGAACTAAAATTACTC

TATCTATCAGATTTACCATAAAAGCTTACTTTGACTTTACAAAACATTTATTAGCAAAATTCGTTTATCA

CCAACCTATTCAAGATTTAAGGGAAAATAGTTATCCTCAAAACTAGGGAATTCAGATTTTTGAAGTTTTT

ACCGATTCTAATGAAAAACAAAGCCCTATTATTTGGGTTTCTTCTCGAGATAAAATAGAATATTGTTGTT

ATGGATTCTTTTTTTCATTTTTATTAAAATTAAAAGAAAATCTAAAAGTTATTTATAAATCAAGTTTTTT

AAAGCTATGTTGGTGGATTGTTTTAGGAAATTTGATCTAACCAACAATTGTAATTTTTTTTTTTGTGAGT

GTGATAAAGTCTACTTTTCAACATTAAAAACTAGAAAATTGAAATTTACGGCTTCTTTATACAATTTTGC

TAGAGCCAGCATTTTTGTGTATAAAACTTTGCATGACTCATACATACCACATGTGACATGTCACGTGTGA

ACTGTGTAGCATAAACATAATATCTAACTGAGTATTCCAAAAACATTTGTAGAAGAAAGTGTTCAAAAAA

GCCTGTCGAGTTATTTACCAGATCTTTTTATCAAAATATTTTATTGGTAGTGGATCATACTCGTTACTTA

ACTATATTTTTATTTTTTATTTGACAGAAAACCTACTCCAGTAGTATTTTTTTTCCACTCAAGAAAAGTA

TTAATTTGATGTTAAAAAAAAAGTATTAATTTTTAAAACAAATTTTCTTACATATTGGTTGTTTAATCAT

TAACTTCCGAACAAACAAAATTGTGGTGCAGCCAAGGCACTGAACCAAGCGAGGTTAGAAGCAAACGTGA

AAGTAAACGGAGAAACCAGCTTCAAGAATAAATGGCCAAGCCCTTTCGCGGTGGTTTCCGGCATATTCCT

CCTCCTCTCCTTCTTAAAATTTGTATACCCACCTCTTCGATGGCTAGCTGTCGTGGGCGTCGCTGCTGGT

ATTTATCCGATTCTTGCAAAATCCGTCGCTTCTATAAGAAGGCTTAGGGTCGACATCAACATCCTAATCA

TTATCACAGGTAATACCACTTTTCACTTTTTATTTAATATTATTATTTTTATCCACATCACTCATATTGA

CGTGTAACTACTGTATAATGATTTGTTAGTTTATGGTTCTCCGTAATCAATATACTATGTAGTATTAGTT

GAAAAATAAATTAGAAAGAAAATTGTGGTTATAGTACAACTATTCAGGCCCTATTAACTAATGTTCTTGG

AAACTTGCGAGTCTTTTACTCTGAATTTAGCAACACCTAGCACTCCACAAAACCTTTTAGAAAAGTTTTC

CTTTACTTTTTTCTTAATATTTTTTAAAAGTATTACATATGGGAAAAATATCAAAACACATATTTATTAA

TTAATAGATGCGCAATTATTACTTTATAGAAATTCAATATTAGGAATGTAGCAATTTGATATTTCTGTTG

TATATGTTAATTGTATATTTGACTTATAAGTTGTGGAACTACATAAAACTACTTTATATTTTCGTTTTAT

GTAAAGTACATTTGAGTAATAGCCTAATAGGATATAGAAAAATATCAAAATGTTCTTTGGATAGGTAAAG

TAAACATGTGATTAAACTCGGAAAGTTGGTATGGAAGATAATGCAAAATATAAAGGTGTATTCAAAATAT

GGAAAAAAAGATACGCCTTTCTCTTTTCTCCTTGCAAAAATCTACATTTTCGTTCATTTATTTGATGATA

CAGTAACGAAGGTGTAAAACATTACTTTTAGATGTGACATACATAGAGTAATTATTTTCATTTTACATAA

TTCTTTTATCGAAAATTACAATATTTCTTTTTAAAAATGTAGTATAAAAAGAGAAATCTTTTACGTACAC

ACAACGTAGATTTATAGATTTTATATAAAACGATCGAGGCAACTAGGAAAATAGAAATTTCATGATCGAT

CGAGAGTTGTGTGGTTCTTTGGGAAAACTTAATTATTTTTTGGTTATTTTATACGAAAGTAAAGGATTCG

TTTGATTCTTGCTCAGTTTTTTTATATTTTTTATAAAAGCTGCAGTTACGTCCCATAGAAGGAAAAAAGG

TTAAGTGGTTTTTGATTGGCTTATCTTCTACGACTCAAAATGGGAAAAAAGCAAACTTTTTAGTTTTAAG

TTTTAACTCGTGAAAAGAAAATTAAAAAGAGCAACAAATAATTGAAAGAACAAAATCATCAAAAGTAAAG

AAATTAATTCATAATTCATTGACTGATAACGGAATTACTTTTAGTTGAAATTTCGGTTTAGGACACCCTG

TTAACAAAGAAAAATAATAGAGACACCAAGCTTGTGAATCCATAATATAAAATATTTTTTGTAATATAAA

ATATAGTCACTTCAACAAAACTATAACTCACTAATATTCCAATTTCATCAAACAGTGGCTGCAACACTTG

CAATGCAAGATTACATGGAGGCTGCAGCAGTTGTCTTCTTATTCACCATCGCTGACTGGCTGGAAACAAG

AGCTAGCTACAAGGTATGTTAACTAGTAATGATCATATATTGTGTTAATCAAACAGCTATGGATTATCTT

GCTTTATTTATAGATGATCTGAAGTTGAAATTGTAATGGATTATTGATTATGGCAATTGCAATCTCAGGC

CAACTCGGTGATGCAGTCTCTGATGAGCTTAGCTCCACAAAAGGCAGTCATAGCAGAGACTGGAGAAGAA

GTTGAAGTAGATGAGGTTCAGCTCAACACAATCATAGCAGTTAAAGCCGGTGAAACCATACCTATTGATG

GAATTGTAGTCGATGGAAACTGTGAAGTAGACGAGAAAACCTTAACCGGTGAAGCATTTCCTGTGCCTAA

ACAGAGAGATTCTACGGTTTTGGCTGGAACTATTAATCTAAATGGTAATGTAACCCTCTTACACAAGCTT

CAATCTTAGAAAAGTTTCAAGCTTTAACCTTTTTGTTTTCGCAGGTTATATAAGTGTGAACACAACTGCT

TTAGCTAGTGATTGTGTGGTTGCAAAGATGGCTAAGCTCGTAGAAGAAGCTCAGAGCAGTAAAACCAAAT

CTCAGAGACTAATAGACAAATGTTCTCAGTACTATACTCCAGGTTTGCAAAAAAACATAAACCATAACTT

GTTTTCTTTATGTTCTTGATTCTTGTAATTTGAGACCTCTCTGTTTTTTGTTTGTTTCAGCAATCATCAT

AATATCGGCTGGCTTTGCGATTGTCCCGGCTATAATGAAAGTTCGCAACCTCAACCATTGGTTTCATTTA

GCACTGGTTGTGTTAGTCAGTGCTTGTCCCTGTGGTCTTATCCTCTCTACACCAGTAGCTACATTCTGTG

CACTTACTAAAGCGGCAACTTCAGGGCTTCTGATCAAAAGTGCTGATTATCTTGACACTCTTTCAAAGAT

CAAGATCGCTGCTTTTGACAAAACCGGAACTATCACTAGAGGAGAGTTCATTGTCATAGAATTCAAGTCA

CTCTCTAGAGACATAAGCCTACGCAGCTTGCTTTACTGGTAATAAAAACAATATCTTGTTCTAACCAAAA

ACTAGTTTGATGAGATAACTTATGAATGACAATTTCTTGTTTGGTTCTCAGGGTATCAAGTGTTGAAAGC

AAATCAAGTCATCCAATGGCAGCAACGATTGTGGACTATGCTAAATCTGTTTCTGTTGAGCCTAGGAGTG

AAGAGGTTGAGGATTATCAAAACTTTCCAGGTGAAGGAATCTATGGGAAGATTGATGGGAACAATGTTTA

CATTGGGAACAAAAGGATTGCTTCACGAGCTGGTTGTTCAACAGGTAAATCTTGGACTTTGGTAAAATCA

AACTCAATGGAATGTTTTTGAGGTTTTGTTGAGTCTTTGATCATTTTGAAACTGTTCTTTCTTGACAGTT

CCAGAGATTGATGTTGATACCAAAAAAGGAAAGACTGTCGGATACGTCTATGTAGGTGAAAGATTAGCTG

GAGTTTTCAATCTTTCCGATGCTTGTAGATCCGGAGTAGCTCAAGCAATGAAGGAACTCAAAGATCTTGG

AATCAAAACCGCAATGCTAACAGGAGATAATCAAGATTCAGCAATGCAAGCTCAAGAACAGGTATGAGGA

CTAAAAAAATCCCAGACATTTCCATTATACTCTCTTAATTGTATCGATTATATATTAAAACCTTGTTTTA

TATGAAAACAGCTAGGGAATGCTTTGGATGTTGTTCATGGAGAGCTTCTTCCAGAAGACAAATCCAAAAT

CATACAAGAGTTTAAGAAAGAAGGACCAACTTGTATGGTAGGAGATGGTGTGAATGATGCACCAGCTTTA

GCTAATGCTGATATTGGTATCTCCATGGGGATTTCTGGCTCTGCGCTCGCGACGCAGACTGGTCATATCA

TTCTTATGTCTAATGATATCAGAAGGATACCACAAGCGATAAAGCTAGCAAGAAGAGCTCAGCGGAAAGT

TCTTCAAAACGTGATCATCTCCATCACTTTGAAAGTAGGGATACTGGTTTTAGCATTTGCTGGTCATCCT

TTGATTTGGGCTGCGGTGCTTACTGATGTAGGGACTTGCCTGATTGTGATTCTCAACAGTATGTTGCTTC

TGCGAGAGAAGGATAAATCTAAGATCAAGAAGTGTTACAGGAAGAAACTTGAAGGCGTCGATGACCAAGG

CCTTGACTTAGAAGCAGGGTTGTTATCAAAGAGTCAATGCAACTCAGGATGTTGTGGTGATAAGAAAAGC

CAAGAGAAGGTGATGTTGATGAGACCAGCTAGTAAAACCAGTTCTGACCATCTTCACTCTGGTTGTTGTG

GTGAAAAGAAGCAAGAGAGTGTAAAGCTTGTGAAAGATAGCTGTTGCGGTGAGAAAAGTAGGAAACCAGT

GGGAGACATGGCTTCACTGAGCTCATGCAAGAAGTCTAACAATGACCTGAAAATGAAAGGTGGTTCAAGT

TGTTGTGCTAGTAAAAATGAGAAGCTGAAGGAAGTAGTAGTAGCAAAGAGCTGCTGTGAAGAGAAGGAGA

AAGCAGAGGGAAATGTTGAGATGCAGATTCTAAATTTGGAGAAAGGGTCGCAGAAAAAGGTTGGTGAAAC

CTGCAAATCAAGCTGTTGTGGAGATAAAGAGAAGGCTAAGGAAACACGTTTGGTGCTTGCTAGTGAGGAT

CCATCTTATCTGGAGAAGGAAGAAAGGCAAACTACTGAAGCTAACATTGTGACAGTGAAACAGAGCTGCC

ATGAGAAGGCAAGTCTGGACATTGAAAATGGAGTTACTTGTGATCTCAAGTTGGTCTGCTGTGGAAACAT

AGAAGTGGGAGAGCAATCTGATCTTGAGAAAGGCATGAAGTTAAAGGGTGAAGGACAATGCAAGTCTGAC

TGCTGCGGTGATGAAATACCTCTAGCTTCTGAGGAAGACAGTGTGGATTGCTCCTCCGGATGCTGCGGAA

ACAAGGAGGAATTGACACAAATCTGTCATGAGAAGGCATGTCTGGACATTGTAAGTTGTGATTCCAAGTT

GGTTTGTTGTGGAGAAACAGAAGTGGAAGTGAGAGAGCAATGTGATCTCAAGAAGGGTCTGCAGATAAAG

AATGAAGGACAATGCGAGTCTGTTTGTTGTGGTGATGAAAAGAAAACAGAGGAGATAACTCTGGTTTCTG

ATGAAGAGACGGACAATCTGAAAAGTGAAAGTGGTGGCGATAGCAAAGCTCTTTGTTGTGGAACTGGTTT

GAAGCAAGAAGGGTCTTCTAGTTTGGTCAATGTGGTGGTGGAGAGCGGTGAATCCGGGTCAAGCTGTTGC

AGCAAGGAGGGAGAGATAGTGAAAGTCTCTAGCCAAAGCCGTTGCACAAGTCCAAGTGATGTGGTGTTAT

CTGACTTGCAAGCTAAGAAACTAGAGATTTGTTGCAAAGTGAAGAAGACTCTTGAGGAGGTTCGTGGATC

TAAATGTAAGGAAACAGAGAAGCCTCACCACGTTGGTAAAAGCTGTTGCAGGAGTTATGCAAAAGAGTAT

TGCAGCCACAGGCATCACCACCACCACCACCACCACCATGTCGGGGCTGCTTGACGACGGATTGATTAGC

TTTAAATTCTCGACGCATCCATCTATTTGCATAACCTTTCCGTCTTCAACCAATGTCGCCGAGAAAAAAT

AAAAACTTCTTTAGTGTTTCCAGCAAAGGTTAAAGGTTTATCAACTGTGTGAATCGTAAAGACAATGCTA

GTGATCGTTGTTAGTCTTTTATGTTTGCCAAAACCCTAATGTATATTTCTTCTTTTCTTGTTTTTATTCT

CTTCTTGAAGATGCCGAGAAGAAGTTTGAACTTCGATCCTAGAGTCTTAAAATCAAATAGAACAAGCAGT

TGAAACATAACTTCAACTAGGCCTGGGCATTCGGGTCTTCGGGTCGGTTCTTGTCGGGTCCGGTTCTTTC

GGGTTTAGAAATTTTTAGACCCATATAGGAACCGATAGGATTTCGGTTCGGTTCGGGTCGGTTTTAGGTC

GGGTCCGGGTCGGTTCGGTTTAGAATTTTCAAAACCTGAAAAATAACCGGTTTTTGGCGGGTCTAATTCG

GTTCGGTTCTTTTTCGGTTATTTCGTACTCATAATCTACTTTTTAACCGAAAATATTACCAAATAACCGA

AAATTTTGCAAAATAACTGGGAAAAAAAACAAAATATCCAAACAAAAGTCAGATTAACGCTCCATTCCAT

CTCATCTCCGTCATCTACGTAAACCTCACATGTCACACAAAACACACATACTTAGTCTATGACTATATGA

CTCAATGCATAATTAGTAAATCATAATTCACAATTTCACAATGTATCAACTCTATGACTATTCGATTCTT

TGTGTTTAGTGATTACCTTACTCTTAAGTTCCTTGAGACTTAAACTCTGGTTCAAACTCTAAACAAAACA

CAAAGAAAAAAACATATCATAAATTAGAAAGATGAGTATCGAATTTCATACCCGTGATTGATTTCGAGAC

GTCTCTGATGGTGGAGACGATTCACCTCGACGATTTCGGATTTCCAGTGTTTTTTTTTCTTGATTGAACT

GGGAATTTATTAAGTATTGAGGATTTGGGAGTCGGGAAAGTAGATCGGGGAGTGGGAGAGACGACATAAG

CTATTGGCGAAGTTGGCGACAAAAAAAATTCTATCGGGGAGTGGGAGAGGAGAAAAGCTAATGGGGTTTA

GTTTTGGGCTGGGTATATGTGGGCTTGGGAAAACTTAGGAAGGGTCTTAGGGTTTTTGGTTTTGGGCTAT

AGGTACCCTTATCGGATATCGGGTAATTACCCGGACCCGAACCGAAAACCGTGGGTCTGCGAAAAAAGGA

CCCAATAGGGTAAAATCCAATTACCCATATCCGATCCGAACCGATTTTTCGGGTCGGTTCCGGGTCGAGT

CCCTGGGTCCGGTTAAAAATGCCCAGGCCTAACTTCAACTTAGGCTTGGATTCTTTTCATTCGAGGAACT

CTACCTGTACATGTGTCATTTATCTTTTGGATTTGAATATTTTTGTTTTTACTCACTGATCGATGTTTAC

TTACGTTTATGCCTTTGATTATACTAGATTTAGACCCGCGCTACGCCGCGGTATTTTTTTCTTTTAATTT

GTTATATTTTTATTTAATTTTGGTCATCATTTTATTATTTAATATATGTGAAATAAAATAGTTTGGGCAT

GTAGCCTGTTTTAATTATCTCTGTGTGAGCTGTCTATTGACTGTGTGA
