# Supplementary material for: Tandem Quadruplication of HMA4 in the Zinc (Zn) and Cadmium (Cd) Hyperaccumulator Noccaea caerulescens
Source: PLoS One. 2011 Mar 10;6(3):e17814. doi: 10.1371/journal.pone.0017814 (PMC3053397; doi:10.1371/journal.pone.0017814)
Supplement: Data S2 — Fosmid P6P46 insert sequence. (DOC) [file pone.0017814.s010.doc]

**Data S2 Fosmid P6P46 insert sequence.**

>Fosmid P6P46 31521bp

ACCAATGTTGAGTTGCGAATTTAAGTTCTAATTAAGAAGAGAATTCACGTTTTATAGAACTGCCGCAATT

TTTTTTTATCTTTCTACCTGATTATTCAAGCACCGAGCATAAGTTATGATCTTGTGCAAACATGTTACTA

ATTTAATAAGATGTATCAAAAATATATAGATACATCTTAGAAGAAAAGCTAAGAGAGTAGACGACAATTA

ATTGGTGCGTTGCGTTAAAAGAATGTGCACATAGTTTAATTAAAATTTTAGAAATAAACTAAGAAAATTG

TACTAGAAACCAAATAAAGAAAGCAATTAGATGAGGAATCACACATGGATTCCATTTTGTGACATTACAC

TTTTGGTGTTTTCCTACTAACATTTTACTATTTTAGTAACTTTAACTTCGTGTCTCTCACTCACGAGATT

AAATCCCCTTTTGATCAAATTTTCTGCTCAATTCTTTCTTTAGATAACTAGCAAGAATCATGATTATAAT

AATTCCAATTCTTAGCATGCGATATTGCGAGGATCATGTGTCTAAACTAGCGATGTATCGGACAAGTATT

ATCCTCGCCCCATATTCAAACTGATAATGTTTACTTTATAATCTCACTCTTCTTTTGTAACCATTTTATA

TAAAGTTTTAATAGATATTTACCATATTTTTATCCCAAAAACTTAGAATATGTAGTTCTTTTGATAAAAC

TCTAATTGATCATCTACTCCATAAAAAGCTAATGTCGAAATTTATAAAACAAAGTCACATGCACAAACAA

CTGATCTTGTCAGTGAGAATGTTTTTACTTATGGTTCAAATCTCAAAAACATAGCCAATTCAAATTTTAT

GAAGTTCGGTAGTACTATAAAATGAGCAATCAATCGTTTATAAAAAGGAGCTAGATTAGACTAGTCTATA

ATCCATTATAGTGAAAACCGTTACCACAAAATATCATACTTTTATTTAGTGCTGATGTAATCGATTTTAA

AATAAACTTATAATTTTCTATTCTTGGAAATTAATCATATGAACTAGCCTGAAAATTCGGAAGGAATATA

GGAATTTAAATCAAAAAATAAAATATATGTGCAATCAATAGTTGAGAAATAAAAATGTACAATTTAATAA

AATTCAGGAATAAATTGAGTGTTTTCCCCTAAATAAACATGGTAAACAAAACAAAAAAATAATGTAAAAG

AAAAGGTTTTAATTCGAAAAGAATCCAAACTAACAACAACTTTAGAAAAGTTGCTTTTATGTTTCAAAAG

AATCCATAAATGTTAGGTTTCGAACGGCTTTTAAATATTTAATAACGATTCTCAAATCTTTTAGGGGTGG

GTGTATTAAAATTAGAATTTGGAGTTATTTGATTTTTAATGGGGTTTTAGATGATTTTAGAGAGAATTGT

GAATATACCCTAAAAAAAGGCTCATTTTGTCTATTTACTTCTTTTTTGACATTGGTAAATTTTTGCCAAA

ATTGTCGTGTCCTTCACTATAATTAAAAACTGATACTAAATTCTTTTACAAGTGAACAAAATTTCTAAAA

TTCATAAAAATGATCTAAAGACTGTTTAAAAAATTTGAACATTTTTAAAATTGTTTTTCATGTTTCAGAA

AATTATATGAAGTTTCATTCTACCAATCTCTAAACTCAAAGTAGAATACGTTTTCTACTAGTTACTCGGA

CGTCCAACGACTATATAATGCAGAATCTACAGCTATGTCATACTTCTACCACAATTAGAATTCGAAATCT

ACCAAATACTATAGGCTCTACCATTTGGTAGAATTTGCTCCCAATATATTCTACAGAATATACTATCTTC

TGCTAATCGTGGATTTCAAGTTCTACTCTAAACACTGACATCTACCTTTCGTAGTTTTCGTATCCTACGT

ACTACAGTTCATTCTACCTCCCGCAGAATTAGTGTTCTACTAAAAGCAAGTTTTCTACTATCCGTAGTAT

TCGTGTTTGTACAAGATGTTCTACTAAAATAAGTATCATTATACCTTTTGTATAAATCATCTTCTACCAA

AATCGAGAAAATATTTCAACTGAATATTTTTCAAATCTTTGTTTCTTTTTTTCCTCTAACTATCCGAAAA

TAAACCAAAATGGTTTAATTAATTTTTAATTTTTAATAATGAAAATAGTTTAATTAAAAATAAAACCTTA

TCTAGTTAATTTGAAGGACAAAAATGGATTATGTGATAAAAAAGGTGTAAGGGGACAAAGAGCAAATGTG

AAAGAGTAATGGGACAAGATAGGTTTCTTTTAGGGTATTTTGGCAATTTTCTCATGATTATGTGAATTAT

AGAAATTCATGTGATTTTGGTTAAATCATTCTAAAATCTTATCTAAAACCAAGTGATTTTGAGTCTTTTA

TTTTTAACAAGAAAATCTCACAAAATCACTCTAAAATCAAATCTAATTTTAAAAATCTACTTTTAAAAAT

ATTTCTAATAACAGTCGATTTGAAAGTGGATTTTAAAATCACAAATTCAATAACACTGAATTTTAATAGG

GTTTTTAGAATTCAAGTTTGAATAACACTAAATTTGTTATTTTAATATAAATCACCGTAAATCACTTCAA

ATCTCAATTTAAATACACCATTTCCTTTCTTAAACTTCTAAGAACCAAATTTGTGGTATATATTTGATAT

ATTTAATCTATTTTTTTCTTCCAACTTTGATCTAATTGAATTTTGTTGGTGATTTTGGAAAAACAAAATA

TAAATTTCGGCACTGTTATGGGTCAATCTCAAAAAAGGTTTTCCTACCAAACTCAAACTGGAATAAACAC

CGGTTCCCGGTTAGACCGGTCCGACCAGCCGGTCCTATCTGATTTTTTAATTATTGTTTTTAGCTATATA

TATATAAGGGACTCATTTTTGTTGAAAGAAGATAAAGTTAACAAAAAAACTTTTGCCTTCTCTTTCGATC

GAATACTATCTCCCACTTTCCTTCTCTCTCTTTTAATATCACACCTTTATATATAATATTTTATAACATA

AATAAAAATTTTAATAAAAAGCTGATAATTTCGAAAATGTTAAAGAACAACAACCAATCATCATCTATCA

TCTAACATTCTCACTTATCTGCAGAGGCGGCTTAGAGGTCAATAGGTGCTCTGCACTAGGGACCTAAGGA

AAAACAAAATTTTAGTATAGAAAAAAATATGGAGACAAAAATTAGTATAGAAAAAAATATAGAATCTTTG

ATTAAGATTATAGTTTTGATTTTGCAATTAAGACTTATAAAATCTTTGACCCGGCCCTGCTTATCCGGCT

ATATAAGCAACTACCATTTCTAGATATCTTCACCTCACAATCTTCCTCTCTTCGTTCCAAAACCTCTCTC

ACTCTCAGTCTTCACCTTTGTGGTAATACTTTAATCTGGTCGAACCGCACCAAACCGGTCCGGTCTTTCT

TCTCGGCCTCGTCTTTTCTCCGGTATTCTTTCTCTTCTTAATTCACATAGATTTCATAACAAGTGATTTC

TTCGTAAAAATTAAAATCCGATCAAATTCACGGTAGTGATATCTCCAACACGTTATATGCATCCCAGCAT

AAAAGTTTTTCTTTCTTATTTTTTTTCCCCTTAAAAGATTTGGNAAAATTAACCATTAATCCCATAATAA

TCTCTTTTTGCGATGTGATTTGTTTTTTTCTTTTTAGATTTCCGTTTCACAGATTCGCCATTAATCCCAT

AATAATCTCGGTTTGTTTTTTTATTTTTAGATTTCCGTTTCACAGATTCGCCATTAATCCCATAATATTC

TCTTTTTATAATGCGATTTGTTTTTTCTTTTTAGATTTCCGTTTCACAGATTCGTTAATCATAAAAAACT

TTGATACAGAAATGGCGTTACAGAAGGAGGACAAGAACAAAGAAGAAAATAAAATGACAAAGAAGAAGTG

GCAGAAGAGTTACTTCGACGTTTTAGGAATCTGTTGTACATCGGAGATTCCTCTGATCGAGAATATTCTC

AAGTCTCTCGACGGCATTAAGGACTATACCATCATCGTTCCGTCGAGAACCGTGATCGTTGTCCACGACA

GTCTCCTCATCTCCCCGTTCCAAATTGGTAAAGCATTAGCTAATCACTTTCTTCGAATTTTTATTTTTAC

CTAATAAAAATAATTGAATCAAAAACCATAAAGTAATCTCACTTAACACGTAAACAATCACTTTACTTTT

CTTCTCTTTCTGTTTTCTTCAAAATTAATTAATGGTTTCGCGTCCTCGTTTGATACGCAAAGCCTCAAAT

TAATTTTTTTTTGGGAACTAAAATTACTCTATCTATCAGATTTACCATAAAAGCTTACTTTGACTTTACA

AAACATTTATTAGCAAAATTCGTTTATCACCAACCTATTCAAGATTTAAGGGAAAATAGTTATCCTCAAA

ACTAGGGAATTCAGATTTTTGAAGTTTTTAACGATTCTACTGAAAAACAAAAGCCCTATTATTTGGGTTT

CTTCTCGAGAAAAAATAGAATATTGTTGTTATGGATTTTTTTTCATTTTTATTAAAATTAAAAGAAAATT

CAAAAGTTATTTATAAATCAAGTTTTTTAAAGCTATTTTGATGGATTGTTTTAGGAAAATTGATCTAACC

AACAATTGTAATTTTTTTTTTTTGTGTGTGTGATAAAGTCTACTTTTTCAACATTAAAAACTAGAAATTG

AAATTTACGGCTTCTTTATACAATTTTGCTCGAGCCAGCATCTTTGTGTATAAAACTTTGCATAACTCAT

ACATACCACATGTGACATGTCACGTGTGTACTGTGTAGCATAAACAATATCTAACTGAGTATTCCAAAAA

CATTTGCAAAAGAAAAGTGTTCAGAAAAGCCTGTTGAGTTATTTACCAGATCTTTTTATAATTTTGCTAG

AGCCAGCTTTTTGTGTATAAAACTTTGCATAACTCACACATACCACATGTGACATGTCACGTGTGAACTG

TGTAGCATAAACATAATATCTAACTGAGTATTCCAAAAACATTTGAAAAAGAAAAGTGGTCAAAAAAGCC

TGTCGAGTTGTTTACCAGATCTTTTTATCAAAATATTTTATTGGTAGTGGATCATACTCGTTACTTAACT

ATATATTTATTTTTTATTTGACTGAAAACCCATTCCAGTAGTACTTTTTTTCCACTCAAGAAAAGTATGA

ATTTGATGTTAAAAAAAAAAAGTATTAATTTTTAAAACAAAATTTCTTACATATTGGTTGTTTAATCATT

AACTTCCAAACAAAATTGCGGTGCAGCTAAGGCACTGAACCAAGCGAGGTTAGAAGCAAACGTGAAAGTA

GACGGAGAAACCAGCTTCAAGAATAAATTGCCAAGCCCTTTCGCGGTGTTTTCCGGCATATTCCTCCTCC

TCTCCTTCTTAAAATTTGTATACCCACCTCTTCGATGGCTAGCTGTCGTGGGCGTCGCTACTGGTATTTA

TCCGATTCTTGCAAAATCCGTCGCTTCTATAAGAAGGCTTAGGGTCGACATCAACATCCTAGTCATTATC

ACAGGTAATACCCACTTTTCACTTTTTATTTAATATTATTATTTTTATCCACATCACTCATATTGCGTGT

AACTACTGTATAATGATTTGTTAGTTTACTATGTAGTATTAGTTGAGAAAGAAAATTGTGGTTATAGTAA

AACTATTCAGGCCCTATTAATAGACCTATAATGTTCTTGGAAACTTGCGAGTCTTTTACGCTGAATTTAC

CCCTTTATATGGTACTTCAGATTAGCTTACCTATATACTACTGCTTTCCTGCAACACCTACCACTCCACG

AAACCTTTTAGAAAGTTATCCTTTACTTTTTTCTTAATATTTTTTTAAAGTATTACATATGGGAAAAATA

TCAAAACACATATTTATTAATTAATAGATGCGCAATTATTACTTTATAGAAATTCAATTCTAGGAATGTA

GCAATTTGATATTTATGTTGTATATGTTAATTGTATATTTGAGTTATAAGTTGTGGAACTACATAAAACT

ACTTTATATTTTCTTTTTATGTAAAGTACATTTGAGTAATAGCCTAATAGGATATAGAAAAATATCAAAA

TGTCAATGTTTTTAAAACCGGACCAGAAGGCGAACCGGATAATCATCCGGGTCATGGTTCAATTTGGTTC

GACCGGGTTGAATTCGGTTCATAATAATTTATGTTTATTTATTTTTAAATATAGAACTTTTATTTTTCAA

AGTTCCCAAGTGTAAACACATACATAGAATAATTATTGTGATTTTACATAATTCTCTTATGGAAATATAA

TAATTCTTTTTTAACATGTAGTTTAAAAAGATAAATCTTTTACGTACACACAACATAGATATATAGATTT

TATATATAACTATCGAGGCAACTAGGAAAATGGAAGTTTCATGATCGAGAGTTGTGTGGTTCTTTGGGAA

AACTTAATTTTTTTGGTTATTTTATACGAAAGTAAAGGATTCGTTTGATTCTTGCTCAGTTTATTATTAT

TATTTTTTAAAAAAAAGCTGCAGTTACGTCCCATAGAAGAAAAAAAGGTTAACTCGTATTTGATTGGCTT

ATCTTCTACGACTCAAAATGGGAAAAACTCAAAAAAGCAAAGCAAACTTTTTAGTTTTAAGTTTTAACTC

GTGAAAAGAAAATTAAAAAGAGCAACAAATAATTGAAAGAACAAAAGCATCAAAAGTAAAGAAATTAATT

CATAATTCATAGACTGATAACGGAGTTACTTTTAGTTGAAATTTCGGTTTAGGACACCAAGCTTGTGAAT

CCATAATATAAAATATTTTTTTAAAATCTTGATCTTTTGTTCGTTTGTATGATGTAATAGTCACTTCAAC

AAAACTATAACTCACTAATATTCCAATTTCATCAAACAGTGGCTGCAACACTTGCAATGCAAGATTACAT

GGAGGCTGCAGCAGTTGTCTTCTTATTCACCATAGCTGACTGGCTGGAAACAAGAGCTAGCTACAAGGTA

TGTTAACTAGTAATCATCATATATTGTGTTAATCAAACTACTATGGATTATCTGAAGTTGAAATTGTAAT

GGATTATTGATTATGGCAATTGCAATCCCAGGCGAGCTCGGTGATGCAGTCTCTGATGAGCTTAGCTCCA

CAAAAGGCAGTCATAGCAGAGACTGGAGAAGAAGTTGAAGTAGATGAGGTTGAGCTCAACACAATCATAG

CAGTTAAAGCCGGTGAAACCATACCTATTGATGGAATTGTAGTCGATGGAAACTGTGAAGTAGACGAGAA

AACCTTAACTGGTGAAGCATTTCCTGTGCCTAAACAGAGAGATTCTACGGTTTGGGCTGGAACTATTAAT

CTAAATGGTAATGTAACCCTCTTACACAAGCTTCAATCTTAGAAAAGTTTCAAGCTTTAACCTTTTTGTT

TTGGCAGGTTATATAAGTGTGAACACAACTGCTTTAGCTAGTGATTGTGTGGTTGCAAAGATGGCTAAGC

TCGTAGAAGAAGCTCAGAGCAGTAAAACCAAATCTCAGAGACTAATAGACAAATATTCTCAGTACTATAC

TCCAGGTTTGCAAAAAAACATAAACCATAACTTGTTTTCTTTATGTTCTTGATTCTTGTAATTTGAGACC

TCTCTGTTTTTTGTTTGTTTCAGCAATCATCATAATATCGGCTGGCTTTGCAATTGTCCCGGCTATAATG

AAAGTTCGCAACCTCAACCATTGGTTTCATTTAGCACTGGTTGTGTTAGTCAGTGCTTGTCCCTGTGGTC

TTATCCTCTCTACACCAGTAGCTACATTCTGTGCACTTACTAAAGCGGCAACTTCAGGGCTTCTGATCAA

AAGTGCTGATTATCTTGACACTCTTTCAAAGATCAAGATCGCTGCTTTTGACAAAACCGGAACTATCACT

AGAGGAGAGTTCATTGTCATAGAATTCAAGTCACTCTCTAGAGACATTAGCCTACGCAGCTTGCTTTACT

GGTAATAAAAACAATATCTTGTTCTAACCAAAAACTAGTTTGATGAGATAACTTATGAATGACAATTTCT

TGTTTGGTTCTCAGGGTATCAAGTGTTGAAAGCAAATCAAGTCATCCAATGGCAACAACGATCGTGGACT

ATGCTAAATCTGTTTCTGTTGAGCCTAGGAGTGAAGAGGTTGAGGATTATCATAACTTTCCAGGTGAAGG

AATCTATGGGAAGATTGATGGGAACAATGTTTACATTGGGAACAAAAGGATTGCTTCACGAGCTGGTTGT

TCAACAGGTAAAGCTTCAAACTTTGGTAAAATCAAACTCAATGGAATGTTTTTGAGGTTTTGTTGAGTCC

TTAATCATTTTGAAACTGTTCTTCCTTGACAGTTCCAGAGATTGATGTTGATACCAAAGAAGGAAAGACT

GTCGGATACGTCTATGTAGATGAAAGATTAGCTGGAGTTTTCAATCTTTCTGATGCTTGTAGATCCGGAG

TAGCTCAAGCAATGAAGGAACTCAAAGATCTTGGAATCAAAACCGCAATGCTAACAGGAGATAATAAAGA

TTCAGCAATGCATGCTCAAGAACAGGTATGAGACTGAAAAAACCAAGAATTTTTCATTACTCTCCTAACG

TTAAGAGATTATATTAAAACTTTGACATGTTCTTATATGGAACAGCTAGGGAATGCTTTGGATGTTGTTC

ATGGAGAGCTTCTTCCAGAAGACAAATCCAAAATCATACAAGAGTTTAAGAAAGAAGGACCAACTTGTAT

GGTAGGAGATGGTGTGAATGATGCACCAGCTTTAGCTAATGCTGATATTGGTATCTCCATGGGGATTTCT

GGCTCTGCGCTCGCGACGCAGTCTGGTCATATCATTCTCATGTCAAATGATATCAGAAGGATACCAAAAG

CGATAAAGCTAGCAAGAAGAGCTCAGCGGAAAGTTCTTGAAAACGTGTTCATCTCCATCACTTTGAAAGT

AGGGATACTGGTTTTAGCATTTGCTGGTCATCCTTTGATTTGGGCTGCGGTGCTTACTGATGTAGGGACT

TGCCTGATTGTGATTTTTAACAGTATGTTGCTTCTGCGAGAGAAGGATAAATCTAAGAACAAGAATTGTT

ACAGGGCTTCTACATCTGTGTTGAATGGTAAGAAACTTGAAGGCGGCGATGACCAAGGCCTTGACTTAGA

AGCAGGGTTGTTCTCAAAGAGTCAATGCAACTCAGGATGTTGTGGTGATAAGAAAAGCCAAGAGAAGGTG

ATGTTGATGAGACCAGCTAGTAAAACCAGTACTGACCATCTTCACTCTGGTTGTTGTGGTGAAAAGAATC

AAGAGAGTGTAAAGCTTGTGAAAGATAGCTGTTGCGGTGAGAAAAGTAAGAAACCAGAGGGAGATATGGC

TTCACTGAGCTCATGCAAGAACTCTAACAATGACCTGAAAATGAAAGGTGGTTCAAGTTGTTGTGCTAGT

AAAAATGAGAAGCTGAAGGAAGTAGTAGTAGCAAAGAGCTGCTGTGGAGAGAAGGAGAAAGCAGAGGGAA

ATGTTGAGATGCAGATTCTAAATTTGGAGAAAGGGTCGCAGAAAAAGGTTGGTGAAACCTGCAAATCAAG

CTGTTGTGGAGATAAAGAGAAGGCTAAGGAAACACGTTTGTTGCTTGCTAGTGAGGATCCATCTTATCTG

GAGAAGGAGAATCTGAAAAGTGAAAGTGGTGATGATTGCAAATCTCTTTGTTGTGGAACTGGTTTGAAGC

AAGAAGGGTCTTCTAGTTTGGTCAATGTTGTGGTGGAGAGTGGTGAATCCGGGTCAAGCTGTTGCAGCAA

GGAGGGAGAGATAGTGAAAGTCTCTAGCCAAAGCTGTTGCACAAGTCCAAGTGATGTGGTGTTATCTGAC

TTTCAAGCTAAGAAACTAGAGATTTGTTGCGAAGTGAAGAAGACTCCAGAGGAGGTTTGTGGATCTAAAT

GTAAGGAAACAGAGAAGCCTCACCACGTTGGTAAAAGCTGTTGCAGGAGTTATGCAAAAGAGTATTGCAG

CCACAGGCATCACGACAACCATCATCACCACCATGTTGGGGCTGCTTGACGGAGATAGTGATTGATTACC

TTTAAACTCTCGACCCATCCATCTATTTGCATAACCTTTCCTTCTTCAACCAATGTCGCCCAGAACAAAA

TAAAAACTTATTTAGTGTTTCCAGCAAAGGTGTGATTCGTAAAGACAATGCTGTTGATCGTTGTTTGTCT

TTTATGTTTGCCAAAACCATAATGTATTTCTCCTTTTCTTGTTTTTATTCTCTTCTTGAAGATGCCCAGA

AGAAGTTTGAACTTCGATCCTAGAGTCTTAAAATCAAATAGAACAAGCAGTTGAAACATAACTTAGCCTT

GGAGTCTTTTTGTATGCTGTGTACTACATAAGCTTTCTTGACTGACACGTTTCTTGTCAATTCTAGGGCA

TTACTTTATTAGGACAGAGAAGGTGTTGCAGTTCGTGTCCTGGAGAGTTTAGGTGAAAAAAAAATAAAGA

GCAAAAACTGACTGCTCGCACATCCATGTAATCAAGAATCAGTAAAAATAAAAATTAATCAAAGGGTGAC

ACAGCTCATGATCTTATATGAATCACCAACCATACTCTTCTCACTATATAAACAAATGTGTCATTTCTTG

AAAACAATCTGAAATATTCCGCAATCGCTGAAAGCATAGCATTAGAGGCAAAACCCTAGCTATTCTTTTG

TTCTCCGTCTTTATTTCTTATCTTTTATTTCGTCAAGCTTGTCGATGAGGTGAAAACCTTCTTAAAATAT

ATTATTCGCTTCTTCAAGTTTAATAAGACATATACCCTAAGTTCCACAAACTTTTGTATCTCGAGATAAA

ACTTGTGACACATCTGATTCAACACGAAACCTAATGTTTTTAAGATCTACTAGGTAGGATACTTGCGCTT

CGCCGCGGAAGACTTTTTTTGTATTTTGATATTTTATATTTTGATATTTTAATCCAGTTTTCCTTATATT

CCATCTGTTCCATATTAAAATGTCGTTTAAGATTTTTTCATACATATTAAGAAAATATTAAAATTTTTTA

TTTTACTCATTATTACTCAAAATTAATCTTCTCAAGGAGAAATGTGTAAAATCTTTGATGAATTCTCTTA

GAAGGAAAGGAAGAATCATGAGTTTGATCTCCATCAATTTCAGAGAAAACTGCTTCCTCTTCCTCCTCCA

GGTTGTTCTTCTTCTTCCTCCATTCTCTTATGCTCCGACGCGGAATCGGCTTAAGACTTTTAAAGAAACA

CAGTCTCTAAACGCTCGATTTACGTTTTCCGGTTAAATGAAGGAAATCAATAAATGAAAACCGGATAAAA

TTAAACCAAAAAAATTGCTAACTGATATTATTTATAATATTTATATTAGATGTTTGTTTTAAATAACGTG

AAGTAGATTATCTTGAGAAGCACATGAGATTTTTCTGTGCTGATTAACTACAATATCTTTACAAAAAAAA

ACAACAGCAATTCCTCGAATGACACACGTAAAAATTAACTCCAAATAATGTAACTTCATTTTTACCCCAG

AATTAATGGTTGATTCCACCAATTTCATTATAATAGCATTACCATTTAGGTCTCTGGTGATGTTTATGTT

TGTATCACCATTCATTAGTTATATAAAATATATAATGCTAATGTTGGGTGTTGATAGTATGTCCACTATA

TAACTTCTTCGTGTTTCTCACTGTGTACATATCATGATAAGAAACTTGTCTCTAGACCTTGTCCCCATTT

TAATATTATACACTTATTCAAAATCTTTATATATATATATATATATATATATATGGTTTATTTATATACT

ATTTCAAAAAAACAATTGTTACAGTTAAATGTTTTAATTTTTAACATGTTGGTTCCAGGAATTCAAAGTG

ACTAATTTGATGATCTATATATGTTGCTTGTAATTTGCGGTTTCAAGTTTTCAACAATGTCTGAAGTGAG

ACAGAAGTGAGACAAAAGACTTTGAAAGATTTGATTGGAATATAAAATCTCGCTATGCTTAAGTATCAAC

TAGACAACTTATAACAGTTGAGACAATAATAGTAAACTATATGTTGGCATAGAAAATGGCGTTTTCTATA

TGAATTGGCTGGTTGATTTATATATTTACAATCTCTAGTTCAGTATTCAGATGTTGTCAAAAAATCGAAA

ATAAGGTTAAATTGTGGTTTCACGGTATAAAAGATTAAATACCAAAGTGGGTATACATCCAAACTCCGGA

ATATATAACTTACGAAAATTTCAATATGAATTACCAAAAACAGTTCTAAGAAATCTGAATACACGTCTTA

ACTCTTAAGTAGTATGTTAGAATTTTAGATGTACTTAGTCGACATTTTTTCAGTTTTCGATGTGAATTTA

GGACTGGTTATCACTCATTAGGACAAAAGTGGATCTCACAGGTTACTACTTTGCATCAATTCTATCATAT

AACTCAATGGTCATAATAGGCTTGGGCATTTTTACCCAGCTCGAAATACTAAACCGAATCTGACCCATAA

TAGATGGAATCGAACCGAACCGGAACACGAATATTCGAATGGGTCCTAAATTCCTATACCGGAAAGAATA

GGACTCGAACCAGAACTGAATCGAGAACCAAAAGAGTACCCAAAATATTCAAAATATAATTATATACCAA

AAAATATTAGTTATATTTAGACTTAAAATAACTAAAATATGTAAAATTACAATTCTAAACTTAATATACT

ACTTAAATTTAGAAAAAATAACCAAAATATTCAACAAATCCAAAACCGACCTGAACCCGGACAGAACCGA

ATCGGATCTGACCCGAAAATAAAAAATATTTGAATGGTTTTAAAATTTCTAGAACGAAAAAAACTAAACA

AAAAACAACCCAAACCCGATCCGAAAAACAGAATGCCCGGGCCTTGGTCAAAAACAGATCGTAGAACGAT

ATGCTTTTCTTTGATAAAGATACGATTATGATAATATTTAACGAATTAAGTATACTATTAAGCTTGACGC

CTTGACCACCTCACTGATAATTTTGTTGCATTGCGCATTTGCACTTTCATAACCATTTTTACAACTTTTC

TCAAAATATTAGATATCGATAAATAAATTAAAGGTATACATTAGTAATTTTCTAAGTCGTATTTATGTTT

ATTTGAATGCATCGCGATAACATATCCAACAATATTTTTCTCGAGATTCGTGTATTAGTTTATACAATAT

TTTTTTAAAAAAATTAGACCGATCAAGAGGCCTCAGCCACACCTAGCTAGGCTTCTAGGAATCCATTGGT

TATAGTCATATAGAATTAACAATTTCTCAATTGTAAAATTATAATCTTGTTTATTCAGATACGTAAAGGT

TAATGAGTCATGTGACATTAATAATTGTCTACTCACATTATTTAGAAGATTCAACGACTCCAAACTATTC

TTGATAGTACAGTTGTTAAATAATTGGAGTACATGTTGGGTCTTTGGTACGACTCTTGCTTGCATTGAAA

TCGATCATAACCATAGATTAACGATTCATAAATGCGAGGGTGACATTTTTCCTTTAAGCCGCCAAACATT

CACTTTTTATTACAATAAATTAAACTGAACGGTTTAGGTTGTTAAGGTGAAAGTGTTATAATTTCAGTTC

AGTTTTACAATAATGCAATTACCGAAATACTTGATTTTGAGATATTGACATGGATTTATACTATTAATTT

AAAGAAAGACAAAATTTAGATGAGGCACTTCACCCCTGCTTAGGCTTCACTACGACCACTAAAATCTTAT

AACGAAGTTTTAGAGATTTACTCCTTGCTTTATAAATATGGATTTCGAATCACATTTAGACAATCACTTA

GATTGGTAATTTTTAACTGAACGGTTTGGTATCAATACATTTTCAAAATTATTAATCAGAAATACTAGTG

TTAAACGAAAATATTCATATTGATGGTTGATTGAGGAGGAGAGGTGGGCTCCAGCATAAAATAGCTGTGC

ATTTTAGGATGGGAATCCCGTTTTTTTCTGTACCAACTCAAATTTTCCAAACTTTTTGAAAATGAGAAGT

AGCAATTTTCTGTTGGATTTATTTATTTTATTTTAATTGGTAAGGAAAAAGTACATGATAATGAGAAAGA

AAGAAATACCTGAATTATTCGTTTAAAAATTAAAAATAAAAACAAAATTATTCATATTTTCCTTAAATTT

TTCGAATATTTTATGTATTTACCCAAAATTTATCGTATTTTTCCATAATTTTTCAAAATCTCATCAATTT

TTCCGATTAATTAAGACTTTGGTCTTTGGATGTCTAATTTTTAGTCACAAACTTCTTATTTCCTTCTTTT

TCCCGTTATTTTCCGTCCCATTATCATAAAAGAATACTTCGGATAATATATTTTGATTCCCGAATTTTTC

ATTATCGTTCAGTTCCCGTCAATACCGGTCACAAAACCCAAAATGCAGATGGTTATGAACAAGTGTACTT

TCTAATTCTACCTATCCACTTGCGCCAAATCTAGTGAATATGTCAAAATTGGCAAAATGAATATAATAAA

CTATATGCCAAAAAAGGTTCATAATTTTTTTTATCAGCATATACTGGTTATCTTTGAATCGCATATCAAT

AAGAAGCATCTCGTTTATCTAATTACCAACGCTAATGAGAGACGACCATTCCCTCATGTTCTCTCATTCA

CCCGCAAAATAGTATTGAGTTCTCCGCCTATGATAATCAGTTTATTCACCAGCCGCAACTCATCATTTAG

TTGATGCGTACGCGACGATGATATGTAACACCTCCGCATCCTTTTTAATTCTCGCATGAATAAACTGGTC

CGTCGAATTAAGGACCTCTACATCCCCTATCCCATGTCTTCAAAATAACCACAATCCACCGCTCTGACTC

ACTGCATCCACTCTGAAAGAACTTTCAAACCCCAAGCCTTGACAAATGCTTCCTGCTCTCTCTGCACCAA

AATGTGTTTCAAAAATAGCCAAAACATCCGTAAGCCACTTCTTCACCATATACCGAATCAATCTTCTGAA

ATAGGGTTTATTCGCCTCCCGACAGTTCCATAAAATACAATTCATCATAATTTCATAGCTTAACCATAGA

AATATCGGGGCAACTTGTTATGCTCCAATCTCCTCCATCTGCTTCTCCGACGATGCGATGTGCGTAACCA

ACGTCCCAATCGAACTACGTATCCAATAAAACCTCGATGTCTGTTTCACCTAGATTTTGAGCCAAGATTC

CATTTTCCCGTACTTCACTTCCCTCCGTCACCACAAAACCCCCAGCCCGACCAAGGTCCCCCTGTTCAAC

TCTGAGGCGTTTACCATTTGCAGATAGAGCAGTCTTCCCCTTGGTTGGGCCAAAAACAAATCCGCTAGTG

GGCCTACTAGTTAAATTTTGTTTGGGCCGAGTAAGTTCCAAACTCTTCCCATTCCTGAACTTTTTTCCTT

TATGCGCAGCTCTAGTGTAATTTGGGCCTTGTCCCGATCCAGACCCAAAAACAATCCCCTTCCCGTGTTC

CTTACTCTTTCGGTGCTTAGTTCCTAAGACCGGATAGACATTTTCCTTATCTCTGTTGCAAACATTAATG

TTCATTGATTCCACTATCTTGATTGCGCCCGTACTGCCCTCTCGTCCATTAACTGTCGAGTCCCTATTCT

CTTCGGTTTCCATATTCTCATCGAGGTTTCCACACTTATTCCTCAACACAATATTCAATGAAACTCTTTC

CTCTCTTGAGACTCGCGTCTCCTCTTCCATCCCTTCTTGACCAACCACCTCCGTTGGCCGTCCCCTACCT

CGTCGAACCTGTGTGAACTCCTCCTCAACCGAAACAGTTTCCTTCCCATTATCCCTATCCTGCTGGACTT

GCATGTATGGACCAAGTGTCCATAAATTCCAAACTGAGAACAAATGTTGGTAAGACCTTCATAGGACAAG

AAATATCTCCCTCCGTTAATCAACACCGTGCCCTTCAATGGTTTCTTTAGATTAACCTCAACGGATACCC

TTGCAAACTGTGCTATTTCAAAGTTTAAAGTTGTGACGTCAACCTTCACCGGTTTCCCAAGACCCTTTGC

AATCCCCTTTATGATAGCCTTATGATAAAAGTTCACCGGAATGTTCGATAACCTAACCCAAACCGGCGTT

GTCTCAATCTTAGGATCAAACTGCGGAGACCACGCTTGCACCATAAGATAACTCCCAAACGCTTTATATG

GACCTCCCGTCAATGCCGCCATGTACTCCTCTTCATTCTCAAAATGGATCATAAAGAATTGACGAGGTAG

ATCCATGACATACATCGCAATTCTAGGTTTCCACATCTCCCTTAACTTCTTATTCATCGCCGTGATTGAC

ACAGTTCGACCTAACACTTTGACAATCATACATTTTTTCCACAAATCGTTCATGGCCGCCAACACTTCCT

CCCTAATCGTTATTACTGGTTCTCCATCCTCACCATTCGGAAATTCCAAACGAAGTCTTGCTTCTACAAA

GGCCTCATCCACCACCGTCTCTGGTACCGGTCTCCCACCCATACTACTCCCCGTCATCCTCTGCGCCTAT

GAGCTTGTTAAATCTGGTGGTCGCCCCTTCTCCCCAACGTCATCCATGTGGGCATTGATGACCCTGCCAC

TCTCGACCCCTGTTTCCTCAATCGCCTCACTCACCGCACTCATTAGGGTTGTTTTTATAAGTTTACTAGT

ATGTGCCACTTAGCAATAGACATACTACTAGACTTATTTAAGTAGTAATGTCTCAATTCTAGGCAAGATT

TATACTTTTGTTTAAAACAGATTATTTATGCTTAAACATGATTTCGTAGATTGGTAGACCTCGGTAGGGA

ATTTGGCATTGATGAATTGATTGGGGATGACTCTAGTAGTCTCGGTAAACATCTAGAGCTTTCCATTAGT

TGCTAACATGTGGGTAATATGTACCAGTATCACATATTATATGATTTGTCACGTCTCGGACCATGATTGT

TTTCAACAGGGGTCCAAAATAGGTCAAGGGTCAAAGCGGGGAATCGAACTTGGGTCAGAGGTTTCAATTA

GCATATTTTACCAATTTTCCTAGTGAGTTTTGCTACAATTTCTGTTTTTATAAAATGAATAGGGTGTCAC

TTAACATCGTATTTTTCTAAGTCGACACCACTGTCTCGGACGCGGTACGGTTTGAATTAAACAAGTAAGA

TTCATTTTTAAGATAAGTTACAAAGAAAATATCAAACATAATTAAAAATTTTGGGAAGCCAACAGATTAA

AGATAATTAATGCTATTCCAATGTTGAGTCGCAAATTTAAGTTCTAATTAAGGAGAGAATTCACTTTTTA

TAGAACTGCCGCAAATTTTTTTTTATCTTTCTACCTGATTATTCAAGCACCGAGCATAAGTTATGATCTT

GTGCAAACATGTTACTAATTTAATAATATGTATTAAAGATATAGATACATCTTAGAAGAAAAGCTAAGAG

ACTAGACGACAAGTGCATTGCGTTAAAAAAATGTGTAGTTTAATTTGAATTTTAGAAATAAACTAAGAAA

ATTGTACTAAAAACCAAATAAAGAAAGCAATTAGATGAGGAATCACACATGGATTCCATTTTGTGAATCC

ATTTTGTGACATTACACTATTGGTGTTTTCCACTAACATTTTACTATTTTAGTAACTTTGACTTCGTATC

TCTCACTCACGAGATTAAATCCCTCTTTGATCAAATTTTCTGCTCAATTATTTCTTTAGATAACTAGCAA

GAATCTATATTATTAAAAGAGAAACAAAACCTGAAAGTTATAAGGATAACACTATATCCAAACAAGAAAA

AAACAAAAAAAAAAAACTGACATAAACCGGGTTGAATCCTGTTAAAAGACCAAACAACCCATTATCTATA

CTATTAAAAGAGAAACAAAATCTGAAATTTACAAATTTATCACTATAACCAAACAAGAAAAAACTCAGTC

ACAACCAAAAATACCCGACTAGATCCGACCCGGCAACAATGAGAAGACAAAAAGCCCAAAGCAGTAACAA

ATAAATATTTTTCGGATACAATTTTAAGAGGGGTTTTCTGGTCCGAAAATCTTCAAGAAATATAGTTGTT

TGGCAACGGAGATACTCGATCCGACCCGAACGCGGCGTAGAAACACGCGGATCTAACAAGAGGAGAGGTG

ACTTAAATTGAACCGACTAAATAAACCGAATATTGTTCCAAAAACAAAAGAACCGGACGCTTACCAAAGC

CCATAAATAAGTCCAATTAACCGGATAGCTCCTAAGAATATGCAAAAAATTATCAAGAAAATTAGTTCTT

TTTCGATATTTGAAACATAAATGTCATATATTTGAAACTTAAATACGATATCACAGCGTAAGAAAGATTT

TGCGTACCCTGATTTAGCATAATAAATACTTCAGCTTCGACGAATGAAAAATCTGCGTCATTTTCCATAA

ATATCGTTAATTAACTGTGCCAATCAAATACAAAAACAGAAATGTCATAAACATCAATAACAACTCGAAT

TTCTTTCCACGCAAAATCTAAGAAGTTTTCCACAAATTTCGCTAATTAACTAAAACGAATATACTTAATA

CCTAATCTAAAGCATGTACTAAGCACACAAAAGTGTCGACATTCATGATAGTATTAAAACTGAATTTGAA

AATCAGTTACCAAAAAGAAAAAATCAAAGATTCTTGTGAAAATATCCCTACCAAAAATATACCGTGTGAT

AATTAATATTTTTAAATCATATTGATTAAAAACCTAATGACCCATCCTATGAAACTATAAATACATCGCT

CACAGGGTAATTGTTACATACCTTGAAATTGCTAATGATTTTTTCCTTCTGTCTAATTTCATGTTTACTA

ATCACGATTTGTTTTATATGTAGGGGAATTCATGAATGGAAAAGCAACCAAAAGCTCTTTCTTAAATCAC

CAAGAAACTATGATACGATCAAAGGTTAATCCCCATCGATCGGGTCAAGAAAATAGATAGTTGCCCCTTA

TTGTTTCACATACCTTTATGATGGCAAGCCTTTCAAAATAGGTTGGGGAATCCAAGTTAAGCTTCTTTTG

GAAGCAATACACGTCCGCATCTGGACAAATGGAGCTAATCCTAGCAAAAGAAAATGTAAGTCAACGTAAT

ATAATAGACTATTAGAGATTTTGTACACGTTTTCGATTCTGAAATCTTAATCTCGATATGTCTTGCAAGG

TATGAAAACACAGGCTTATGAATGTCACCATACCTTTAATTCGATTACGCGTGATTTTCATCGAAGTTTA

CAGAACCAACTGATCCGACGGATTCACCAAATCGATTTCATATCAGAAGACTAGATATCAATTTACGATC

TTCGAGTCTACGCTCACGGTTGTGCGACTTTTCGTTTTTCGCAAGATGGTAAACAAAAAGAGTGGATCCG

ACAAAGGCCCACTTTTTAATGGATAAAAACGACGAGAAATGGGCTTATCCGTTTTAGACTCATACCCGAA

AAGAATTAGTTCGGCCCAAATTTTGACCACTTAATTTATCACTTTTAATCCTACACTAAAATTAACACTA

AAATTAACACTGAATAATTGTATAATTCATCTCATATCATATAATATATTTTTTACCTAATGTATTTTAA

CGTATCAAAAATTACAAAATCTCGTGCTTCTCAACAACATACATTTCTTCGTTATATATCAGAAATATAT

TATTCTTTACATAATTACAATATAAATACTTTAGGGGGGTTTATTGGTAGATGAATTTGTAAGAATTCTT

AAAATTTTCAGAAATCTTTGTTATTGATTTGTGAATTCTAACAATCTTATTAAAATCTGTTGTTATTGGT

TTGGTGATTTATAAAGTCAATACAAAATCAGTTGTTATTCAAAAAGTTTGTGTTTTAATGATTTCATGAA

TCCATTAAAATCCTTGTTATTGGGACATGGATTTTAAACATTTTAACTCATAGAACAAGATTTCCAAAAT

ACTAGCTATAACCCTTAGATTTTCAAAATTCATTATAACAAAATATTTTGATTGATTTTATGAATATACA

ATCTCTCTCCAAATCTAACATAAACTCTTCATAAATTTAACAAATCTCTTAACTTTCAAAATTTATCAAC

TCTATAGAAATTCATCTCCCAATAACCCCCCTTAGTTTTCTTATAAAAAATTACAACATCATGTGTCTAG

TCATGATTATAATAATTCCAATTCTTAGTATGCAATATTGCGAGGATCATGTGTCTAAACTAGCGACGTA

TCGGACAAGTTTTATCCTCGCCCCATATTCAAATTGATAATGTTTTATAATCTCACTTTTCTTTTGTAAC

CATTTTATATAAAGTGTTAATAGATATATACCATATTTTTATCCCAAAAACTTAAGAGTATGTAGTTGTT

TTGATAAAACTCTAATTGATCATCTACTCCATAAAAAGCTAATTTCGAAATTTATAAAACAAAGTCACAT

GCACAAACAACTTATCTTGTGATTAAGGATGTTTTTACTTATGACTCAGCTAGGTTCAAATCTCAAAAAC

ATAGCCAATTCAAATTTTATGAAGTTCGGTACTACTGTAAATTGAGCCACCAATCGTTTTTAAAAAGGAG

TTCGATTAGACTAGTCTATAATCCATTATAGTGAAAATTGCTACACAAAATATCATACTTTTATATAGTG

CTAATGTAATCGATTTTAAAATAAACTTATAGTTTTATATTCTTGGAAATTACTGAAAACAAAAAGAAAC

TACATTTTGATAGGAACTAGCCTGAAAATTCGGAAGGAAAATAGGAATTCGAAACAAAGATTAAAATATC

CTACGAAATTAACATGGTAAAAAAAACTAAACCAAATAAAAAAAGTTTAACGTAAAAAGAAAAGTTTTAA

TTCAAAAGAATCCAGACTAACCACAACTTTTGAAGCATAATCCATAAATGTTAGGTTTAGAACGACTTTT

AAAGATTTAGTAACTATTTTCAAATCATTTAGTACGTCTTTCTTTTTTTTTTTGGCTCAAACATAACAGA

TTTCATTAGAAATGATTTCATTAGTACGTCTTTCTTAAACTTCTAAGAACCAAATTTGTGGTATATATTT

GATATATTTGAGTTATTTTTTCTTTTGACTTTTATGTAATTGAATTCTTTTGGGGATTTCGGAAAAGCAA

AATATACATTTCGGTACTGTTAGAAGGAGAAAATTACATCAATGAGGAGGACTTTGTAACTATGGTACAT

TTTGCCTTAACAAAAATACATTGTTGCCCTTTTATTTGTTTCACCTTTAGACAATTTAACTTTATATTTG

GGAAAATCGCATTTTAAGCCGATAAATGCTAACATTTTAAACTTTTAAAGTTTTTATTAGCACTTCAAAC

ACTCAAACTATTTTTTTCATACTTTAAACGAAAGTAACAAATTTTTACGCGCCGCCGAAAAAATTAATGA

AAAAAATTTAATATTCTAAATTCAAAGAATCAAAAATTAAACGAAAAAGGCTTAATAACTTGAACTCAAT

CATACAAATTTTATTTACCCTATTTCGACTTATAAACATTCAATTATCTCTTTGAAACTCTGCATTGTAT

AAAAAATTGTTAATTTTATGGTTTAAAATACGAGGATAAATAGTTTGAGTGTTGTCATTGGTCATTTTCA

TTGTTTTAAAATGCAATTTCTCCCTTTATATTTTACTATTTTTTCAATTTTTACTTTTTATCTCTCAACA

AAGAGATATCTTCTCTCCCTCACGATAAATCCTTCTCTCTCTTCTTTTCTTTCTCCACTTTATTCATCTC

CACTTTCCTTCTCTCTTTGCTTCCTACAAATTCTGTCTCTCATTTTCCACTAGGTTCTTCTTCCACCAAT

AAAGATTGGGACAAGGGTTGCGTAAAGACATTATTATGCCTTGAATAAGTGTTTGTGTATGCATGCCCTT

TTTCAAAAAAAAAAAAGAATAAGTGTTTGTGAACAAAATATCTTGGATAGAATTTTAGAATATATCACGT

AGATCGTGGACAATAGGTGTGTGAACATATATATATATATTCAGACATATAACAATAAAATATGTACACA

AGCTTCGATTTTCACCTCTGATACAACAATTCATCCACATCTTTATTATGTCCAAACAAAAGTAATCCAC

GGCAATAAATCTACATAATTTGGCTCTCTTTGATTCTTCCACTCTTACTTTCATCTTTTTATTTCCGTAA

TCACAAGCAACAATGTTTCATTTTCACTTCTCGTTATCATCATATATTACAATTTTTTACCCATGTTAAC

CCTATCAAAACACCAATTAGATACATGAAAACAATTCAATCCCACACCAAACCAGAAATCGTTTTAATTT

ATAGCTTCAGTTTCAGAAATTAATATACGCCAAAAACGTTGGTTACTGTGAAACACACACCAAACCAAAA

ATCACCATCACAGAAATAGCCAAATTTATCCACAATTGTATAAGAAAAAGTCTTTGCGTAAGGAGAGTTA

TAAAAGTAAATTTCTCGTACACAAGTGTCACCAAGTGAACATACATTAGTCAATGTTAGTATTTTAATAT

TCTCTTTAGCTATATATAAGGACCCATCTTTGTTGAAAGAAGATGAAGTTCACAAAAAAACTTTTGCCTT

CTCTCTCTATCGAATACTGTTATCCCACTTTCCTTCCCTTTTCCTTCCCTCTCTCTTTTAATATCTCACC

TTTATATATAATATTTTATAACATTAATAATTTTTTAATAAATCCTAATAGGGGTATGATATATAATTAT

ATATGGTCAAAGAAGGTGGAAGAAGCGTGAGGATTACTGTGGAGGAAAAAGAGACATTGGAGAAAGCAAC

GGTCAACATTACTGTCAGATGTCGAAGGAGAGAAAGTGAGAGAGTGTGAGACTCTGAGAGAGAGAGAAGT

CAAGAAGGAGAAGAAGACAAAAGCTAATTTAAAGCTACGAATAATTTAAAGCTACGAAGACGAGACGGGA

CATATATTCACCCTCGCTTTTCACATATATTTTCGGTATTGCCACTCTCAAATTTTATTTTTTCCCTTTT

TTCTTGTCTTTTTTGACCCGGCCCTGCTTATTTGGCTATATAAGCAACTACCTTATCTAGATATCTTCAC

CTCGCAATCTTCCTCTCTACGTTCCAAAACCTCTCTCACTCTCTGTCTTCACCTTTGTGGTAATACTTTA

ATCTCTGATCGAACCGCACCAAACCAGTCCGGTCTTTCTTCTCGGCCTCGTCTTTTCTCCGGTATTCTTT

CTCTTCTTAATTCACATAGATTTCATAACAAGTGATTTTTTCGTAATAATTAAAATCCGATCAAATTCAC

GATAGTGATATGATATATGCATATATGCATCCAACACGTTATATGCATCCCAGCATAACAGTTTTGCTTT

CTTATTTTTTTTCCCTTAAAAGATTTGGAAAATTAGCCATTAATCCCATAATAATCTCTTTTTGCGATGT

GATTTGTTTTTTTCTGTTTTAGATTTCCGTTTCACAGATTCGCCATTAATCCCATAATAATCTCGATTTG

TTTTTTATTTTTAGATTTCCGTTTCACAGATTCGCCATTAATCCCATAATATTCTCTTTTTATAATGCGA

TTTGTTTTTTTCTTTTTAGATTTCCGTTTCACAGATTCGTTAATCATAAAAAACTTTGATACAGAAATGG

CGTTACAGAAGGAGGACAAGAACAAAGAAGAAAATAAAATGACAAAGAAGAAGTGGCAGAAGAGTTACTT

CGACGTTTTAGGAATCTGTTGTACATCGGAGATTCCTCTGATCGAGAATATTCTCAAGTCTCTCGACGGC

ATTAAGGACTATACCATCATCGTTCCGTCGAGAACCGTGATCGTTGTCCACGACAGTCTCCTCATCTCCC

CGTTCCAAATTGGTAAAGCATTAGCTAATCACTTTCTTCGAATTTTTATTTTTACCTAATAAAAATAATT

GAATCAAAAACCATAAAGTAATCTCACTTAACACGTAAACAATCACTTTACTTTTCTTCTCTTTCTGTTT

TCTTCAAAATTAATTAATGGTTTCGCGTCCTCGTTTGATACGCAAAGCCTCAAATTAATTTTTTTTTGGG

AACTAAAATTACTCTATCTATCAGATTTACCATAAAAGCTTACTTTGACTTTACAAAACATTTATTAGCA

AAATTCGTTTATCACCAACCTATTCAAGATTTAAGGGAAAATAGTTATCCTCAAAACTAGGGAATTCAGA

TTTTTGAAGTTTTTAACGATTCTACTGAAAAACAAAAGCCCTATTATTTGGGTTTCTTCTCGAGAAAAAA

TAGAATATTGTTGTTATGGATTTTTTTTCATTTTTATTAAAATTAAAAGAAAATTCAAAAGTTATTTATA

AATCAAGTTTTTTAAAGCTATTTTGATGGATTGTTTTAGGAAAATTGATCTAACCAACAATTGTAATTTT

TTTTTTTTGTGTGTGTGATAAAGTCTACTTTTTCAACATTAAAAACTAGAAATTGAAATTTACGGCTTCT

TTATACAATTTTGCTCGAGCCAGCATCTTTGTGTATAAAACTTTGCATAACTCATACATACCACATGTGA

CATGTCACGTGTGTACTGTGTAGCATAAACAATATCTAACTGAGTATTCCAAAAACATTTGCAAAAGAAA

AGTGTTCAGAAAAGCCTGTTGAGTTATTTACCAGATCTTTTTATAATTTTGCTAGAGCCAGCTTTTTTGT

GTATAAAACTTTGCATAACTCACACATACCACATGTGACATGTCACGTGTGAACTGTGTAGCATAAACAT

AATATCTAACTGAGTATTCCAAAAACATTTGTAAAAGAAAAGTGTTCAAAAAAGCCTGTCGAGTTGTTTA

CCAGATCTTTTTATCAAAATATTTTATTGGTAGTGGATCATACTCGTTACTTAACTATATATTTATTTTT

TATTTGACTGAAAACCCATTCCAGTAGTACTTTTTTTCCACTCAAGAAAAGTATGAATTTGATGTTAAAA

AAAAAAAAGTATTAATTTTTAAAACAAAATTTCTTACATATTGGTTGTTTAATCATTAACTTCCAAACAA

AATTGCGGTGCAGCTAAGGCACTGAACCAAGCGAGGTTAGAAGCAAACGTGAAAGTAGACGGAGAAACCA

GCTTCAAGAATAAATTGCCAAGCCCTTTCGCGGTGTTTTCCGGCATATTCCTCCTCCTCTCCTTCTTAAA

ATTTGTATACCCACCTCTTCGATGGCTAGCTGTCGTGGGCGTCGCTACTGGTATTTATCCGATTCTTGCA

AAATCCGTCGCTTCTATAAGAAGGCTTAGGGTCGACATCAACATCCTAGTCATTATCACAGGTAATACCC

ACTTTTCACTTTTTATTTAATATTATTATTTTTATCCACATCACTCATATTGCGTGTAACTACTGTATAA

TGATTTGTTAGTTTACTATGTAGTATTAGTTGAGAAAGAAAATTGTGGTTATAGTAAAACTATTCAGGCC

CTATTAATAGACCTATAATGTTCTTGGAAACTTGCGAGTCTTTTACGCTGAATTTACCCCTTTATATGGT

ACTTCAGATTAGCTTACCTATATACTACTGCTTTCCTGCAACACCTACCACTCCACGAAACCTTTTAGAA

AGTTATCCTTTACTTTTTTCTTAATATTTTTTTAAAGTATTACATATGGGAAAAATATCAAAACACATAT

TTATTAATTAATAGATGCGCAATTATTACTTTATAGAAATTCAATTCTAGGAATGTAGCAATTTGATATT

TATGTTGTATATGTTAATTGTATATTTGAGTTATAAGTTGTGGAACTACATAAAACTACTTTATATTTTC

TTTTTATGTAAAGTACATTTGAGTAATAGCCTAATAGGATATAGAAAAATATCAAAATGTCAATGTTTTT

AAAACCGGACCAGAAGGCGAACCGGATAATCATCCGGGTCATGGTTCAATTTGGTTCGACCGGGTTGAAT

TCGGTTCATAATAATTTATGTTTATTTATTTTTAAATATAGAACTTTTATTTTTCAAAGTTCCCAAGTGT

AAACACATACATAGAATAATTATTGTGATTTTACATAATTCTCTTATGGAAATATAATAATTCTTTTTTA

ACATGTAGTTTAAAAAGATAAATCTTTTACGTACACACAACATAGATATATAGATTTTATATATAACTAT

CGAGGCAACTAGGAAAATGGAAGTTTCATGATCGAGAGTTGTGTGGTTCTTTGGGAAAACTTAATTTTTT

TGGTTATTTTATACGAAAGTAAAGGATTCGTTTGATTCTTGCTCAGTTTATTATTATTATTTTTTAAAAA

AAAGCTGCAGTTACGTCCCATAGAAGAAAAAAAGGTTAACTCGTATTTGATTGGCTTATCTTCTACGACT

CAAAATGGGAAAAACTCAAAAAAGCAAAGCAAACTTTTTAGTTTTAAGTTTTAACTCGTGAAAAGAAAAT

TAAAAAGAGCAACAAATAATTGAAAGAACAAAAGCATCAAAAGTAAAGAAATTAATTCATAATTCATAGA

CTGATAACGGAGTTACTTTTAGTTGAAATTTCGGTTTAGGACACCAAGCTTGTGAATCCATAATATAAAA

TATTTTTTTAAAATCTTGATCTTTTGTTCGTTTGTATGATGTAATAGTCACTTCAACAAAACTATAACTC

ACTAATATTCCAATTTCATCAAACAGTGGCTGCAACACTTGCAATGCAAGATTACATGGAGGCTGCAGCA

GTTGTCTTCTTATTCACCATAGCTGACTGGCTGGAAACAAGAGCTAGCTACAAGGTATGTTAACTAGTAA

TCATCATATATTGTGTTAATCAAACTACTATGGATTATCTGAAGTTGAAATTGTAATGGATTATTGATTA

TGGCAATTGCAATCCCAGGCGAGCTCGGTGATGCAGTCTCTGATGAGCTTAGCTCCACAAAAGGCAGTCA

TAGCAGAGACTGGAGAAGAAGTTGAAGTAGATGAGGTTGAGCTCAACACAATCATAGCAGTTAAAGCCGG

TGAAACCATACCTATTGATGGAATTGTAGTCGATGGAAACTGTGAAGTAGACGAGAAAACCTTAACTGGT

GAAGCATTTCCTGTGCCTAAACAGAGAGATTCTACGGTTTGGGCTGGAACTATTAATCTAAATGGTAATG

TAACCCTCTTACACAAGCTTCAATCTTAGAAAAGTTTCAAGCTTTAACCTTTTTGTTTTGGCAGGTTATA

TAAGTGTGAACACAACTGCTTTAGCTAGTGATTGTGTGGTTGCAAAGATGGCTAAGCTCGTAGAAGAAGC

TCAGAGCAGTAAAACCAAATCTCAGAGACTAATAGACAAATATTCTCAGTACTATACTCCAGGTTTGCAA

AAAAACATAAACCATAACTTGTTTTCTTTATGTTCTTGATTCTTGTAATTTGAGACCTCTCTGTTTTTTG

TTTGTTTCAGCAATCATCATAATATCGGCTGGCTTTGCAATTGTCCCGGCTATAATGAAAGTTCGCAACC

TCAACCATTGGTTTCATTTAGCACTGGTTGTGTTAGTCAGTGCTTGTCCCTGTGGTCTTATCCTCTCTAC

ACCAGTAGCTACATTCTGTGCACTTACTAAAGCGGCAACTTCAGGGCTTCTGATCAAAAGTGCTGATTAT

CTTGACACTCTTTCAAAGATCAAGATCGCTGCTTTTGACAAAACCGGAACTATCACTAGAGGAGAGTTCA

TTGTCATAGAATTCAAGTCACTCTCTAGAGACATAAGCCTAAGCAGCTTGCTTTACTGGTAATAAAACAA

TATCTTGTTCTAACCAAAAACTAGTTTGATGGGATAACGTATGAATGACAATTTCTTGTTTGGTTCTCAG

GTATCAAGTGTTGAAAGCAAATCAAGTCATCCAATGGCAGCAACGATTGTGGACTATGCTAAATCTGTTT

CTGTTGAGCCTAGGAGTGAAGAGGTTGAGGATTATCAGAACTTTCCTGGTGAAGGAATCTATGGGAAGAT

TGATGGGAACAATGTTTACATTGGGAACAAAAGGATTGCTTCACGAGCTGGTTGTTCAACAGGTAAAGCT

TCAAACTTTGGCCAAGAAAAAACTCAATGGAATGGTTTTGTTGAGTCCTTAATCATTTTGAAACTGTTCT

TCCTTGACAGTTCCAGAGATTGATGTTGATACCAAAGAAGGAAAGACTGTCGGATACGTCTATGTAGATG

AAAGATTAGCTGGAGTTTTCAATCTTTCTGATGCTTGTAGATCCGGAGTAGCTCAAGCAATGAAGGAACT

CAAAGATCTTGGAATCAAAACCGCAATGCTAACAGGAGATAATAAAGATTCAGCAATGCATGCTCAAGAA

CAGGTATGAGACTGAAAAAACCAAGAATTTTTCATTACTCTCCTAACGTTAAGAGATTATATTAAAACTT

TGACATGTTCTTATATGGAACAGCTAGGGAATGCTTTGGATGTTGTTCATGGAGAGCTTCTTCCAGAAGA

CAAATCCAAAATCATACAAGAGTTTAAGAAAGAAGGACCAACTTGTATGGTAGGAGATGGTGTGAATGAT

GCACCAGCTTTAGCTAATGCTGATATTGGTATCTCCATGGGATTTCTGGCTCTGCGCTCGCGACGCAGTC

TGGTCATATCATTCTCATGTCAAATGATATCAGAAGGATACCAAAGCGATAAAGCTAGCAAGAAGAGCTC

AGCGGAAAGTTCTTGAAAACGTGTTCATCTCCATCACTTTGAAAGTAGGGATACTGGTTTTAGCATTTGC

TGGTCATCCTTTGATTTGGGCTGCGGTGCTTACTGATGTAGGGACTTGCCTGATTGTGATTTTTAACAGT

ATGTTGCTTCTGCGAGAGAAGGATAAATCTAAGAACAAGAATTGTTACAGGGCTTCTACATCTGTGTTGA

ATGGTAAGAAACTTGAAGGCGATGATGAAGAAGGTCTTGACTTAGAAGCAGGGTTGGTATCAAAGAGTCA

ATGCAACTCAGGATGTTGTGGTGATAAGAAAAGCCAAGAGAAGGTGATGTTGATGAGACCAGCTAGTAAA

ACCAGTACTGACCATCTTCACTCTGGTTGTTGTGGTGAAAAGATCAAGAGAGTGTAAAGCTTGTGAAAGA

TAGCTGTTGCGGTGAGAAAAGTAAGAAACCAGAGGGAGATATGGCTTCACTGAGCTCATGCAAGAACTCT

AACAATGACCTGAAAATGAAAGGTGGTTCAAGTTGTTGTGCTAGTAAAAATGAGAAGCTGAAGGAAGTAG

TAGTAGCAAAGAGCTGCTGTGGAGAGAAGGAGAAAGCAGAGGGAAATGTTGAGATGCAGATTCTAAATTT

GGAGAAAGGGTCGCAGAAAAAGGTTGGTGAAACCTGCAAATCAAGCTGTTGTGGAGATAAAGAGAAGGCT

AAGGAAACACGTTTGTTGCTTGCTAGTGAGGATCCATCTTATCTGGAGAAGGAGAATCTGAAAAGTGAAA

GTGGTGATGATTGCAAATCTCTTTGTTGTGGAACTGGTTTGAAGCAAGAAGGGTCTTCTAGTTTGGTCAA

TGTTGTGGTGGAGAGTGGTGAATCCGGGTCAAGCTGTTGCAGCAAGGAGGGAGAGATAGTGAAAGTCTCT

AGCCAAAGCTGTTGCACAAGTCCAAGTGATGTGGTGTTATCTGACTTTCAAGCTAAGAAACTAGAGATTT

GTTGCGAAGTGAAGAAGACTCCAGAGGAGGTTTGTGGATCTAAATGTAAGGAAACAGAGAAGCCTCACCA

CGTTGGTAAAAGCTGTTGCAGGAGTTATGCAAAAGAGTATTGCAGCCACAGGCATCACGACAACCATCAT

CACCACCATGTTGGGGCTGCTTGACGGAGATAGTGATTGATTACCTTTAAACTCTCGACCCATCCATCTA

TTTGCATAACCTTTCCTTCTTCAACCAATGTCGCCCAGAACAAAATAAAAACTTATTTAGTGTTTCCAGC

AAAGGTGTGATTCGTAAAGACAATGCTGTTGATCGTTGTTTGTCTTTTATGTTTGCCAAAACCATAATGT

ATTTCTCCTTTTCTTGTTTTTATTCTCTTCTTGAAGATGCCCAGAAGAAGTTTGAACTTCGATCCTAGAG

TCTTAAAATCAAATAGAACAAGCAGTTGAAACATAACTTAGCCTTGGAGTCTTTTTGTATGCTGTGTACT

ACATAAGCTTTCTTGACTGACACGTTTCTTGTCAATTCTAGGGCATTACTTTATTAGGACAGAGAAGGTG

TTGCAGTTCGTGTCCTGGAGAGTTTAGGTGAAAAAAAAATAAAGAGCAAAAACTGACTGCTCGCACATCC

ATGTAATCAAGAATCAGTAAAAATAAAAATTAATCAAAGGGTGACACAGCTCATGATCTTATATGAATCA

CCAACCATACTCTTCTCACTATATAAACAAATGTGTCATTTCTTGAAAACAATCTGAAATATTCCGCAAT

CGCTGAAAGCATAGCATTAGAGGCAAAACCCTAGCTATTCTTTTGTTCTCCGTCTTTATTTCTTATCTTT

TATTTCGTCAAGCTTGTCGATGAGGTGAAAACCTTCTTAAAATATATTATTCGCTTCTTCAAGTTTAATA

AGACATATACCCTAAGTTCCACAAACTTTTGTATCTCGAGATAAAACTTGTGACACATCTGATTCAACAC

GAAACCTAATGTTTTTAAGATCTACTAGGTAGGATACTTGCGCTTCGCCGCGGAAGACTTTTTTTGTATT

TTGATATTTTATATTTTGATATTTTAATCCAGTTTTCCTTATATTCCATCTGTTCCATATTAAAATGTCG

TTTAAGATTTTTTCATACATATTAAGAAAATATTAAAATTTTTTATTTTACTCATTATTACTCAAAATTA

ATCTTCTCAAGGAGAAATGTGTAAAATCTTTGATGAATTCTCTTAGAAGGAAAGGAAGAATCATGAGTTT

GATCTCCATCAATTTCAGAGAAAACTGCTTCCTCTTCCTCCTCCAGGTTGTTCTTCTTCTTCCTCCATTC

TCTTATGCTCCGACGCGGAATCGGCTTAAGACTTTTAAAGAAACACAGTCTCTAAACGCTCGATTTACGT

TTTCCGGTTAAATGAAGGAAATCAATAAATGAAAACCGGATAAAATTAAACCAAAAAAATTGCTGACTGA

TATTATTTATAATATTTATATTAGATGTTTGTTTTAAATAACGTGAAGTAGATTATCTTGAGAAGCACAT

GAGATTTTTCTGTGCTGATTAACTACAATATCTTTACAAAAAAAAAACAACAGCAATTCCTCGAATGACA

CACGTAAAAATTAACTCCAAATAATGTAACTTCATTTTTACCCCAGAATTAATGGTTGATTCCACCAATT

TCATTATAATAGCATTACCATTTAGGTCTCTGGTGATGTTTATGTTTGTATCACCATTCATTAGTTATAT

AAAATATATAATGCTAATGTT
