# Supplementary material for: Tandem Quadruplication of HMA4 in the Zinc (Zn) and Cadmium (Cd) Hyperaccumulator Noccaea caerulescens
Source: PLoS One. 2011 Mar 10;6(3):e17814. doi: 10.1371/journal.pone.0017814 (PMC3053397; doi:10.1371/journal.pone.0017814)
Supplement: Data S3 — Fosmid J12P81 insert sequence. (DOC) [file pone.0017814.s011.doc]

**Data S3 Fosmid J12P81 insert sequence.**

>Fosmid J12P81 31218bp

AACAATTTCTCATTGTAAAATTATAATCTGGTTTATTGAGATATGTAAAGGTTAATGGTCAGGAGGCATT

AATAATTGTCTACTCACATTATTTAGAAGATTCAACGACTCCAAACTATTCTTGATAGTACAGTTGTTAA

ATAATTGGAGTACATGTTGGTCTTTGGTACGACTCTTGCTTGCATTGAAATCGGTCATAGCCATAGATTA

ACGAGTCATAAATGCGAGGGTGACATTTTTCCTTTAAGCCGCCAAACATTCACTTTTTATTACAGCCAAT

TAAACTGAACGGTTCTGGTGGTTAAGGTGAAAGTGTTATAATTTCAGTTCAGTTTTACAATAACGCAATT

ACCGAAATACTTGATTTTGAGATATTGACATGGATTTATATTATTAATTTAAAGAAAGACAAAATTTAGA

TGGGACACTTCACCCCTGCTTAAGCTTCACTACGACCACTAAAATCTTATAACGAAGTTTTAGAGATTTA

CTACTTGCTTTATAAATATGGATTTCGAATCACATTTAGACAATCACTTAGATTGGTAATTTTTAACTGA

ACGGTTTGGTATCAGTACATTTTCAAAATTATTAATCAGAAATGCCTATGTTCAAGATTTCGCTAATCGC

TAACCAGGCGGTTGGTCACCGATTAGCGATTTTTCAAAAATCGGTGATAAATCGAGGATTAATCGGGGTA

GAATTTTTACTATATTTTAATATATTTTAAAAATTATATATATAAAACATTAATACCAAAATCATAAACT

CATACAAAAGATTATATTAAGCATTTTTATCAATTCACATATAATAACAAAGGTAAAAATAGTCTTGATC

ATATAGAGAACATACAAAAAATAACTAAAGGATGGTAATTTAATAATTTTTCATCTTTATATTCCTCAAT

GATCAAATTGTCCATAAATGTGGACTAAAAATGGTAATTTCCTAATTGTTCATCTTATTTTGGGTCCGTT

TCATTTTTTTTTAACACATGGCAAAGCAAAATAAAGTAAATGGGCTTCAATTTTTTAATGGCCCCAAAAA

ATTTCTGATTAATCGGTCAATTTTTTGCAATTAATTGGTCAAACCAGTCAAACCACGGTTGACCGGTTTG

TGATAACGATTAGGGGAAATCGACTCGGTCAGCACCCGATTAGCGATTAAACGGCCGATTAATCGTTAAA

TCGGCCGGTTTTTTGAACAGAGAGAAATGCTAATGTTAAACGAAAATATTCATAGTGATGGTTGACTGAC

GAGCAGAGGTGGGCTCCAGCATAAAATAGCTGTGCATTTTAGCTTTTGGGAAGAGAATGCCGTTCCTTAC

GGTACCGGTCTCAAATTTTCCAAATTTTGTGAAAATGAGAAGTAGCAATTTCCTGTTTGGGCTTATTTCC

TTTATTTTAGCTAGTAAGGAAAGGGTACATCATAGGTATGGGAAATAAAATCCAAATTACCCGTTTAAAA

ATTATAAATAAAAATAAAATTATTAATATTAATATTTTTAAAAAAATTTTGAATATTTCTCATATTTATC

AAATTTCCCATATTTTTCAATAATTTCTCAAAATCTTTTCAATTTTTCCGATTAAGTAAGATTTTGATAT

TTGAGTATGTAATTTTTAGTCCCAAACTTCTCATTTCCTTCATTTTCCCCTTATTTTCCATGCCATTATC

ATAAAAGAATACTTCAGATAATATATTTTGATTCCCGAATTTTTCATTGTCGTCCGATTCCCGTCAATAC

CTGTCACAAAACCCAAAATGTACAGGTTATGAACAAATGTACTTTCTAATTCTACCTAATCTAGTGAATA

TGTCAAAATTGGAAAAATGAATATAATAAACTATATGCCAAAAAAGGTTCATAATTTTTTTATTAGCATA

AACATAATTAGGATCTTAAAATTTTTTATCTTTAGTAAATTAGCTCTTCTGGTTAAAGATATCAAAAGGA

GTTGTAAATATATGATTATTTTTAGAAATGTGCTATTTTGTATGTTTAGAAATGGTTTGTACTCTGAACA

TGCATACTTTCGTAAAAAGTAACATAATAACACATAATAAACAGTAAAAAGTAACTTAAACTATTTTGGA

CTATACGAGGTGGATCCATAACATACATCGCACCTCTAGATTTCTACATCTCCCTTAACTTCTTACTCAT

CACCGTGATCGACACAGTTCGACATAACACTTTGACAATTATACATTTTTTCCACAAACCGTTCATGGCC

ACCAACACTTCCTCCCCAATCGTTATTACTGGTTCTTCATCCTCACCATTCGGAAATTCCAAAGGAAGTC

TCGCTTCTACAGAGGCCTCATCCACCACCGTCTCCGGTACCGGTCTCCCACCCATACTACTCCCCGTCAC

CCTCTGCGCCCATGAGCCTGTTAAATCTGGTGGTCGCCCCTTCTCCCCAACGTCCTCCATGTGGGCATCG

ATGACCCTGCCAATCTCGACCCCCGTTTTCTCAATCGCCTCACTCGCCGCACTCATTAGGTGTTTTTTTA

AGAATTTTACTAGTGTGTGCCACTTAGCAATAAACATACTACTAGACTTATTTAAGTAGTAATGTCACAA

TTCTAGGCAAGATTTATACTTTTGTTTGAAATAGCTTATTTATCTTTAAACATGATTTCGTAGATTGGTA

GACCTCGGTACGGAATTTGGCATTGATAAATTGATTGGGGATGACTCTAGTAGTCTCGGTAAACATCTAA

AGCTTTCCATTAGTTGCTAAAATGTGGGTAATATGTACCAGTATCACATAATATATAATTTTTCACGTCT

CGGACGAGGATTGTTTTCAATAGGGGTCAAAAATAGGTGAAGGGTCAAAGCGGGGAATCGAACTTGTGGG

TCAGAGGTTTCAATTAGCATATTTTACCAATTTTCCAAGTGAATCTTATGGCATTTTAGCTACAATTTCT

GGTTTTATAAAATGAATAGGGTGTCACTTAACATCGTATTCTTCTAAGTCGACGCCACTGTCTCGGACGC

GGTACGGTTTGAATTAAACAAGGTTAATTTTTAAGATAAGTTACAAAGAAAAGATCAAACATAATTAAAA

AATTTGGGAAGCCAACAGATTAAAGATAATTAATGCTATTCCAATGTTGAGTCGCAAATTTAAGTTCTAA

TTAAGGAGAGAATTCACTTTTTATAGAACTGCCGCAATTTTTTTTATCTTTCTACCTGATTATTCAAGCA

CCGAGCATAAGTTATGATCTTGTGCAAACATGTTACTAATTTAATAATATGTATTAAAGATATAGATACA

TCTTAGAAGAAAAGCTAAGAGAGTAGACGACAAGTGCATTGCGTAAAAAAATGTGTAGTTTAATTAGAAT

TTTAGAAATAAACTAAGAAAATTGTACTAAAAACCAAATAAAGAAAGCGATTAGATGAGGAATCACACAT

GGATTCCATTTTGTGACATTACACTATTGGTGTTTTCCACTAACATTTTATTATTTTAGTAACTTTGACT

TCGTATCTCTCACTCACGAGATTAAATCCCTCTTTGATCAAATTTTCTGCTCAATTCTTTCTTTAGAGAA

CTAGCAAGAATCATGATTATAATAATTCCAATTCTTAGTATGCAATATTGCGAGGATCATGTGTCTAAAC

TAGCGACGTATCGGACAAGTTTTATCCTCGCCCCATATTCAAACTGATAATGTTTTATAATCTCACTTTT

CTTTTGTAACCATTTTATATAAAGTGTTAATAGATATATACCATATTTTATCCCAAAAACTTAGAATATG

TAGTTGTTTTGATAAAACTCTAATTGATCATCTACTCCATAAAAAGCTAATTTCGAAATTTATAAAACAA

AGTCACATGCACAAACAACTTATCTTGTGATTAAGGATGTTTTTACTTATGACTCAGCTAGGTTCAAATC

TCAAAAACATAGCCAATTCAAATTTTATGAAGTTCCGTACTACTGTAAAATGAGCCATCAATCGTTTTTA

AAAAGGAGTTAGATTAGACTAGTCTATAATCCATTATAGTGAAAACTGCTACACAAAATATCATACTTTT

ATATAGTGCTAATGTAATCGATTTTAAAATAAACTTACAGTTTTATATTCTTGGAAATTACTGAAAACAA

TAAGAAATTACATCTTGATAGGAACTAGGTTGAAAATTCGGAAGGAATATAGGAATTCGAAACAAAGATT

AAAATATCCTACGAAATTAACATAGTAAAAAAAAAACTAAACCAAATAAAAAAAGTTTAACGTAAAAAGA

AAAGTTTTAATTCAAAGAATCCAAACTAACCACAACTTTTGAAACATAATCCATAAATGTTAGGTTTAGA

ACGGCTTTTACAGATTTAGTAACTATTCTCAAATCATTTAGTACGTCTTTCTTAAACTTCTAAGAACCAA

ATTTGTGGTATATATTTGATATATTTGAGTTATCTTTTTCGTTCGACTTTGATGTAATTGAATTTTTTGG

GGGATTTCGGGAAAGCAAAATATACATTTCGGTACTGTTAGAACGAGAAAATTACATCAATAAGGACTTT

TTGACTTATCTTATTACAGCAAGAAGGACAGTGTAACTATGGTGCACTTTGCCTTAACAAAAATACATTG

TTGCTCTTTTTATTTGTTTTACCTTTACACAATTTAACTTTATATTTTACCAGTTACACAATTTATCTCT

CTCCACGATAAATCCTTCTCTCTCTTCTTTTCTTTCTCCACTTTATTCATCTCCACTTTCCTTATCTCTT

TGCTTCCTACAAATTCTCTGTCTTTCTCATTTTCCACTAGGTTCTTCTTCCACCAATAAAGATTGGGACA

AGGGTTGCGTAAAGACATTATTATGCCTTGAATAAGTGTTTGTGTATGCATGCCCTTTTTCAAAAAAAAA

AAAGAATAAGTGTTTGTGAACAAAATATCTTGGATAGAATTTTAGAATATGATTGACAAAAAAAAAAGAA

TTTTAGAATATATCACGTAGATCGTGGACAATATGCCATGGACAATATGGGTGTGTGAACATATATATAT

TCAGACATATAACAATAAAATATGTACACAAGCTTCGATTTTCACCTCTGATACACAATTCATCCACATC

TTTATTATGTCCAAACAAAAGTAATCCACGGCAATTAATCTACATAATTTGGCTTTCTCTTTGATTCTTC

CACTCTTACTTTCATCTTTTTATTTCCGTAATCACAAGCAACAATGTTTCATTTTCACTTCTCGTTATCA

TCATATATTACAATTTTCTACCCATGTTAACCCTATTAAAACACCAATTGGATACATGAAAACAATTCAA

TCCCACACCAAACCAGAAATCGTTTTAATTTCTAGCTTCAGTTTCAGAAACCAATTTACGCCAAAAACGT

TGTTTACTGTGAAACACACACCAAACCAAAAATCACCATCACAAAAATAGCCAAATTCATCCATAATTGT

ATAAGTAACCGTACAAACGTATATAACAAAGCGTACACATGTTTTCAATTGTCCAAAAACACATTTTACT

ACAATTAAACGAAGCTATCAGATCCATGTCCTCTCCCAGAAAAGAAAATAAACACTCGTCAAGATAACCA

TATTATTTATGAATAAGTAAAATTCATGCGATGCAATTTCCTCCAGCCGTATCAGTCTCTACGTGGTGCG

GTCGCCGGAGTTAAATTAAAAGACACCAACCAAAATAATCTGATTTTTCACCCCTTTTTCATTAAAGGTA

AAATGGAACTCTCAACACACTATAAGCAAAAAAGAAAAAAAGTCCACCACTTTCACAATTTCCCTCTTAT

AAAAGTCACTCTTAGTGTAAATGACAAATGACTCCTGTTAGAACTACCTAAAAGCAGGTTATGGAATTCG

TTGGTCAAATGAATTAAAAAAAAAAAACAAATTATAAATTGTTATGAAAGAAAATATCTCGTACACAAGT

GTCACCAAGTATAAGGGACCCATCTTTGTTGAAAGAAGATGAAGTTAACAAAAAAACTTTTGCCTTCTCT

CTCTCTCCATCGAATACTACTATTATCCCACTTTCCTTCCCTCTCTCTTTCGAAAATGTTAAAGAACAAA

AACCAATCATCTAACATTCTGACTTATCTACAGGGGCGGCTTATTCGAGTGGGGTCAATAGATGCTCTGC

ACTAGGTGACGTAGAAAAACAAAATTTTAGTAGAAAAAAAGTTCACTTAAGCCGGCTATATAAAGCAACT

ACCATTTCTAGATATCTTCACCTCACAATCTTCCTCTCTACGTTCTAAAACCTCTCTCACTCTCAGTCTT

CACCTTTGTGGTAATACTTTAATCTGATCGAACCGCACCAAACAAGTCCGGTCTTTCTTCTCGGCCTCGT

CTTTTCTCCGGTATTCTTTCTCTTCTTAATTCACATAGATTTCATAACAAGTGATTTCTTCGTAATAATT

AATATCCGATCAAATTCACGATAGTGATATCTCCAACACGTTATATGCATGATGCATCCCAGCATAAAAG

TTTTGCTTTCTTAATTTTTTTCCCTTAAAAGATTGGAAATGGCTGCCATTAATCCCATAATAATCTCTTT

TTGCGATGTGATTTATTTTTTTCTTTTTAGATTTCCGTTTCACAGATTCGTTAATCATAAAAAACTTTGA

TACAGAAATGGCGTTACAGAAGGAGATCAAGAACAAAGAAGAAGATAAAAAGACAAAGAAGAAGTGGCAG

AAGAGTTACTTCGACGTTTTGGGAATCTGTTGTACATCGGAGATTCCTGTGATCGAGAATATTCTCAAGT

CTCTCGACGGCGTTAAGGAATATACCGTCATCGTTCCGTCGAGAACCGTGATCGTTGTCCACGACAGTCT

CCTCATCTCCCCGTTCCAAATTGGTAAGCATTAGCTAATCACTTTATTCGAATTTTATATCATTTTTATT

TTTACTTAATAAAAATAATAGAATAAAAAGCATAAAGTAATCTCACTTAACACGTAAACAATCACTTTAC

TTTTCTTCTCTTTCTGTTTTCTTTAAAATTAATTAATGGTTTCGCGTCCTCGTTTGATACGCAAAGCCTC

AAACGTTACTTTTTGGGAACTAAAATTACTCTATCTATCAGATTTACCATAAAAGCTTACTTTGACTTTA

CAAAACATTTATTAGCAAAATTCGTTTATCACCAACCTATTCAAGATTTAAGGGAAAATAGTTATCCTCA

AAACTAGGGAATTCAGATTTTTGAAGTTTTTACCGATTCTAATGAAAAACAAAGCCCTATTATTTGGGTT

TCTTCTCGAGATAAAATAGAATATTGTTGTTATGGATTCTTTTTTTCATTTTTATTAAAATTAAAAGAAA

ATCTAAAAGTTATTTATAAATCAAGTTTTTTAAAGCTATGTTGGTGGATTGTTTTAGGAAATTTGATCTA

ACCAACAATTGTAATTTTTTTTTTGTGAGTGTGATAAAGTCTACTTTTCAACATTAAAAACTAGAAAATT

GAAATTTACGGCTTCTTTATACAATTTTGCTAGAGCCAGCATTTTTGTGTATAAAACTTTGCATGACTCA

TACATACCACATGTGACATGTCACGTGTGAACTGTGTAGCATAAACATAATATCTAACTGAGTATTCCAA

AAACATTTGTAGAAGAAAGTGTTCAAAAAAGCCTGTCGAGTTATTTACCAGATCTTTTTATCAAAATATT

TTATTGGTAGTGGATCATACTCGTTACTTAACTATATTTTTATTTTTATTTGACAGAAAACCTACTCCAG

TAGTATTTTTTTTCCACTCAAGAAAAGTATTAATTTGATGTTAAAAAAAAGTATTAATTTTTAAAACAAA

TTTTCTTACATATTGGTTGTTTAATCATTAACTTCCGAACAAACAAAATTGTGGTGCAGCCAAGGCACTG

AACCAAGCGAGGTTAGAAGCAAACGTGAAAGTAAACGGAGAAACCAGCTTCAAGAATAAATGGCCAAGCC

CTTTCGCGGTGGTTTCCGGCATATTCCTCCTCCTCTCCTTCTTAAAATTTGTATACCCACCTCTTCGATG

GCTAGCTGTCGTGGGCGTCGCTGCTGGTATTTATCCGATTCTTGCAAAATCCGTCGCTTCTATAAGAAGG

CTTAGGGTCGACATCAACATCCTAATCATTATCACAGGTAATACCACTTTTCACTTTTTATTTAATATTA

TTATTTTTATCCACATCACTCATATTGACGTGTAACTACTGTATAATGATTTGTTAGTTTATGGTTCTCC

GTAATCAATATACTATGTAGTATTAGTTGAAAAATAAATTAGAAAGAAAATTGTGGTTATAGTACAACTA

TTCAGGCCCTATTAACTAATGTTCTTGGAAACTTGCGAGTCTTTTACTCTGAATTTAGCAACACCTAGCA

CTCCACAAAACCTTTTAGAAAAGTTTTCCTTTACTTTTTCTTAATATTTTTTAAAAGTATTACATATGGG

AAAAATATCAAAACACATATTTATTAATTAATAGATGCGCAATTATTACTTTATAGAAATTCAATATTAG

GAATGTAGCAATTTGATATTTCTGTTGTATATGTTAATTGTATATTTGACTTATAAGTTGTGGAACTACA

TAAAACTACTTTATATTTTCGTTTTATGTAAAGTACATTTGAGTAATAGCCTAATAGGATATAGAAAAAT

ATCAAAATGTTCTTTGGATAGGTAAAGTAAACATGTGATTAAACTCGGAAAGTTGGTATGGAAGATAATG

CAAAATATAAAGGTGTATTCAAAATATGGAAAAAAGATACGCCTTTCTCTTTTCTCCTTGCAAAAATCTA

CATTTTCGTTCATTTATTTGATGATACAGTAACGAAGGTGTAAAACATTACTTTTAGATGTGACATACAT

AGAGTAATTATTTTCATTTTACATAATTCTTTTATCGAAAATTACAATATTTCTTTTTAAAAATGTAGTA

TAAAAAGAGAAATCTTTTACGTACACACAACGTAGATTTATAGATTTTATATAAAACGATCGAGGCAACT

AGGAAAATAGAAATTTCATGATCGATCGAGAGTTGTGTGGTTCTTTGGGAAAACTTAATTATTTTTTGGT

TATTTTATACGAAAGTAAAGGATTCGTTTGATTCTTGCTCAGTTTTTTTATATTTTTTATAAAAGCTGCA

GTTACGTCCCATAGAAGGAAAAAGGTTAAGTGGTTTTTGATTGGCTTATCTTCTACGACTCAAAATGGGA

AAAAAGCAAACTTTTTAGTTTTAAGTTTTAACTCGTGAAAAGAAAATTAAAAAGAGCAACAAATAATTGA

AAGAACAAAATCATCAAAAGTAAAGAAATTAATTCATAATTCATTGACTGATAACGGAATTACTTTTAGT

TGAAATTTCGGTTTAGGACACCCTGTTAACAAAGAAAAATAATAGAGACACCAAGCTTGTGAATCCATAA

TATAAAATATTTTTTGTAATATAAAATATAGTCACTTCAACAAAACTATAACTCACTAATATTCCAATTT

CATCAAACAGTGGCTGCAACACTTGCAATGCAAGATTACATGGAGGCTGCAGCAGTTGTCTTCTTATTCA

CCATCGCTGACTGGCTGGAAACAAGAGCTAGCTACAAGGTATGTTAACTAGTAATGATCATATATTGTGT

TAATCAAACAGCTATGGATTATCTTGCTTTATTTATAGATGATCTGAAGTTGAAATTGTAATGGATTATT

GATTATGGCAATTGCAATCTCAGGCCAACTCGGTGATGCAGTCTCTGATGAGCTTAGCTCCACAAAAGGC

AGTCATAGCAGAGACTGGAGAAGAAGTTGAAGTAGATGAGGTTCAGCTCAACACAATCATAGCAGTTAAA

GCCGGTGAAACCATACCTATTGATGGAATTGTAGTCGATGGAAACTGTGAAGTAGACGAGAAAACCTTAA

CCGGTGAAGCATTTCCTGTGCCTAAACAGAGAGATTCTACGGTTTTGGCTGGAACTATTAATCTAAATGG

TAATGTAACCCTCTTACACAAGCTTCAATCTTAGAAAAGTTTCAAGCTTTAACCTTTTTGTTTTCGCAGG

TTATATAAGTGTGAACACAACTGCTTTAGCTAGTGATTGTGTGGTTGCAAAGATGGCTAAGCTCGTAGAA

GAAGCTCAGAGCAGTAAAACCAAATCTCAGAGACTAATAGACAAATGTTCTCAGTACTATACTCCAGGTT

TGCAAAAAAACATAAACCATAACTTGTTTTCTTTATGTTCTTGATTCTTGTAATTTGAGACCTCTCTGTT

TTTTGTTTGTTTCAGCAATCATCATAATATCGGCTGGCTTTGCGATTGTCCCGGCTATAATGAAAGTTCG

CAACCTCAACCATTGGTTTCATTTAGCACTGGTTGTGTTAGTCAGTGCTTGTCCCTGTGGTCTTATCCTC

TCTACACCAGTAGCTACATTCTGTGCACTTACTAAAGCGGCAACTTCAGGGCTTCTGATCAAAAGTGCTG

ATTATCTTGACACTCTTTCAAAGATCAAGATCGCTGCTTTTGACAAAACCGGAACTATCACTAGAGGAGA

GTTCATTGTCATAGAATTCAAGTCACTCTCTAGAGACATAAGCCTACGCAGCTTGCTTTACTGGTAATAA

AAACAATATCTTGTTCTAACCAAAAACTAGTTTGATGAGATAACTTATGAATGACAATTTCTTGTTTGGT

TCTCAGGGTATCAAGTGTTGAAAGCAAATCAAGTCATCCAATGGCAGCAACGATTGTGGACTATGCTAAA

TCTGTTTCTGTTGAGCCTAGGAGTGAAGAGGTTGAGGATTATCAAAACTTTCCAGGTGAAGGAATCTATG

GGAAGATTGATGGGAACAATGTTTACATTGGGAACAAAAGGATTGCTTCACGAGCTGGTTGTTCAACAGG

TAAATCTTGGACTTTGGTAAAATCAAACTCAATGGAATGTTTTTGAGGTTTTGTTGAGTCTTTGATCATT

TTGAAACTGTTCTTTCTTGACAGTTCCAGAGATTGATGTTGATACCAAAAAAGGAAAGACTGTCGGATAC

GTCTATGTAGGTGAAAGATTAGCTGGAGTTTTCAATCTTTCCGATGCTTGTAGATCCGGAGTAGCTCAAG

CAATGAAGGAACTCAAAGATCTTGGAATCAAAACCGCAATGCTAACAGGAGATAATCAAGATTCAGCAAT

GCAAGCTCAAGAACAGGTATGAGGACTAAAAAAATCCCAGACATTTCCATTATACTCTCTTAATTGTATC

GATTATATATTAAAACCTTGTTTTTATATGAAAACAGCTAGGGAATGCTTTGGATGTTGTTCATGGAGAG

CTTCTTCCAGAAGACAAATCCAAAATCATACAAGAGTTTAAGAAAGAAGGACCAACTTGTATGGTAGGAG

ATGGTGTGAATGATGCACCAGCTTTAGCTAATGCTGATATTGGTATCTCCATGGGGATTTCTGGCTCTGC

GCTCGCGACGCAGACTGGTCATATCATTCTTATGTCTAATGATATCAGAAGGATACCACAAGCGATAAAG

CTAGCAAGAAGAGCTCAGCGGAAAGTTCTTCAAAACGTGATCATCTCCATCACTTTGAAAGTAGGGATAC

TGGTTTTAGCATTTGCTGGTCATCCTTTGATTTGGGCTGCGGTGCTTACTGATGTAGGGACTTGCCTGAT

TGTGATTCTCAACAGTATGTTGCTTCTGCGAGAGAAGGATAAATCTAAGATCAAGAAGTGTTACAGGAAG

AAACTTGAAGGCGTCGATGACCAAGGCCTTGACTTAGAAGCAGGGTTGTTATCAAAGAGTCAATGCAACT

CAGGATGTTGTGGTGATAAGAAAAGCCAAGAGAAGGTGATGTTGATGAGACCAGCTAGTAAAACCAGTTC

TGACCATCTTCACTCTGGTTGTTGTGGTGAAAAGAAGCAAGAGAGTGTAAAGCTTGTGAAAGATAGCTGT

TGCGGTGAGAAAAGTAGGAAACCAGTGGGAGACATGGCTTCACTGAGCTCATGCAAGAAGTCTAACAATG

ACCTGAAAATGAAAGGTGGTTCAAGTTGTTGTGCTAGTAAAAATGAGAAGCTGAAGGAAGTAGTAGTAGC

AAAGAGCTGCTGTGAAGAGAAGGAGAAAGCAGAGGGAAATGTTGAGATGCAGATTCTAAATTTGGAGAAA

GGGTCGCAGAAAAAGGTTGGTGAAACCTGCAAATCAAGCTGTTGTGGAGATAAAGAGAAGGCTAAGGAAA

CACGTTTGGTGCTTGCTAGTGAGGATCCATCTTATCTGGAGAAGGAAGAAAGGCAAACTACTGAAGCTAA

CATTGTGACAGTGAAACAGAGCTGCCATGAGAAGGCAAGTCTGGACATTGAAAATGGAGTTACTTGTGAT

CTCAAGTTGGTCTGCTGTGGAAACATAGAAGTGGGAGAGCAATCTGATCTTGAGAAAGGCATGAAGTTAA

AGGGTGAAGGACAATGCAAGTCTGACTGCTGCGGTGATGAAATACCTCTAGCTTCTGAGGAAGACAGTGT

GGATTGCTCCTCCGGATGCTGCGGAAACAAGGAGGAATTGACACAAATCTGTCATGAGAAGGCATGTCTG

GACATTGTAAGTTGTGATTCCAAGTTGGTTTGTTGTGGAGAAACAGAAGTGGAAGTGAGAGAGCAATGTG

ATCTCAAGAAGGGTCTGCAGATAAAGAATGAAGGACAATGCGAGTCTGTTTGTTGTGGTGATGAAAAGAA

AACAGAGGAGATAACTCTGGTTTCTGATGAAGAGACGGACAATCTGAAAAGTGAAAGTGGTGGCGATAGC

AAAGCTCTTTGTTGTGGAACTGGTTTGAAGCAAGAAGGGTCTTCTAGTTTGGTCAATGTGGTGGTGGAGA

GCGGTGAATCCGGGTCAAGCTGTTGCAGCAAGGAGGGAGAGATAGTGAAAGTCTCTAGCCAAAGCCGTTG

CACAAGTCCAAGTGATGTGGTGTTATCTGACTTGCAAGCTAAGAAACTAGAGATTTGTTGCAAAGTGAAG

AAGACTCTTGAGGAGGTTCGTGGATCTAAATGTAAGGAAACAGAGAAGCCTCACCACGTTGGTAAAAGCT

GTTGCAGGAGTTATGCAAAAGAGTATTGCAGCCACAGGCATCACCACCACCACCACCACCACCATGTCGG

GGCTGCTTGACGACGGATTGATTAGCTTTAAATTCTCGACGCATCCATCTATTTGCATAACCTTTCCGTC

TTCAACCAATGTCGCCGAGAAAAATAAAAACTTCTTTAGTGTTTCCAGCAAAGGTTAAAGGTTTATCAAC

TGTGTGAATCGTAAAGACAATGCTAGTGATCGTTGTTAGTCTTTTATGTTTGCCAAAACCCTAATGTATA

TTTCTTCTTTTCTTGTTTTTATTCTCTTCTTGAAGATGCCGAGAAGAAGTTTGAACTTCGATCCTAGAGT

CTTAAAATCAAATAGAACAAGCAGTTGAAACATAACTTCAACTAGGCCTGGGCATTCGGGTCTTCGGGTC

GGTTCTTGTCGGGTCCGGTTCTTTCGGGTTTAGAAATTTTTAGACCCATATAGGAACCGATAGGATTTCG

GTTCGGTTCGGGTCGGTTTTAGGTCGGGTCCGGGTCGGTTCGGTTTAGAATTTTCAAAACCTGAAAAATA

ACCGGTTTTTGGCGGGTCTAATTCGGTTCGGTTCTTTTTCGGTTATTTCGTACTCATAATCTACTTTTTA

ACCGAAAATATTACCAAATAACCGAAAATTTTGCAAAATAACTGGGAAAAAAAACAAAATATCCAAACAA

AAGTCAGATTAACGCTCCATTCCATCTCATCTCCGTCATCTACGTAAACCTCACATGTCACACAAAACAC

ACATACTTAGTCTATGACTATATGACTCAATGCATAATTAGTAAATCATAATTCACAATTTCACAATGTA

TCAACTCTATGACTATTCGATTCTTTGTGTTTAGTGATTACCTTACTCTTAAGTTCCTTGAGACTTAAAC

TCTGGTTCAAACTCTAAACAAAACACAAGAAAAAAACATATCATAAATTAGAAAGATGAGTATCGAATTT

CATACCCGTGATTGATTTCGAGACGTCTCTGATGGTGGAGACGATTCACCTCGACGATTTCGGATTTCCA

GTGTTTTTTTTCTTGATTGAACTGGGAATTTATTAAGTATTGAGGATTTGGGAGTCGGGAAAGTAGATCG

GGGAGTGGGAGAGACGACATAAGCTATTGGCGAAGTTGGCGACAAAAAAAATTCTATCGGGGAGTGGGAG

AGGAGAAAAGCTAATGGGGTTTAGTTTTGGGCTGGGTATATGTGGGCTTGGGAAAACTTAGGAAGGGTCT

TAGGGTTTTTGGTTTTGGGCTATAGGTACCCTTATCGGATATCGGGTAATTACCCGGACCCGAACCGAAA

ACCGTGGGTCTGCGAAAAAAGGACCCAATAGGGTAAAATCCAATTACCCATATCCGATCCGAACCGATTT

TTCGGGTCGGTTCCGGGTCGAGTCCCTGGGTCCGGTTAAAAATGCCCAGGCCTAACTTCAACTTAGGCTT

GGATTCTTTTCATTCGAGGAACTCTACCTGTACATGTGTCATTTATCTTTTGGATTTGAATATTTTTGTT

TTTACTCACTGATCGATGTTTACTTACGTTTATGCCTTTGATTATACTAGATTTAGACCCGCGCTACGCC

GCGGTATTTTTTTCTTTTAATTTGTTATATTTTTATTTAATTTTGGTCATCATTTTATTATTTAATATAT

GTGAAATAAAATAGTTTGGGCATGTAGCCTGTTTTAATTATCTCTGTGTGAGCTGTCTATTGACTGTGTG

AATCTTCTTTTTAGGGACGACGTACCGTCTAGAACATCTTCGAGAAGAGCTTGCTCCTCCTCCACCTCTT

CTTCTTCTTCATCATCATCTTTTTTGTTTCTTAATTTTTTGAGTGATTTTTCGACTTTGTTCTGTTTCTT

TGGTTGAACGATAATCGTTTGCTTCTTACTCTCTTTTGCATAAGTTGATGGAAATTTTCGGAGACTGGTT

AAATGGCGGCGCCGAAAATCAAAAACGAAGACTCGTCATCGAACGGGAATACTGCTTATGCTAAAATCAC

GCACAATTGAGATTTTACGAAAAGACTGAATGGGTCGGAAACAATAAATCAAATCCATTTTCTCCCGCAA

CTAATTCTCGTTTAAACTCGACACAGAAAAGAAGAAGAGATTGGCCTCTGAATTCGCGTCAAAATTATTT

ATGCGGATAAAATCAATTTTACCGGTTCGGTGGAATAAAAAAGAACAAATTAAACCAATTTCTATTTCAC

TTAAATCAAACCGGTTAAAAAGCTGATGTGTATTAATGAAATGCTGACTGATATTATTTGAAATGTTCCT

ATTGGCCAGACGTTTTTGCTGAGGTGTCAGCTCATACCTTACTCTTAAGTTCCTTGAGACTTAAACTCTG

GTTCAAACTCTAAACAAAACACAAAGAAAAAAACATATCATAAATTAGAAAGATGAGTATCGAATTTCAT

ACCCGTGATTGATTTCGAGACGTCTCTGATGGTGGAGACGATTCACCTCGACGATTTCGGATTTCCAGTG

TTTTTTTTTCTTGATTGAACTGGGAATTTATTAAGTATTGAGGATTTGGGAGTCGGGAAAGTAGATCGGG

GAGTGGGAGAGACGACATAAGCTATTGGCGAAGTTGGCGACAAAAAAAATTCTATCGGGGAGTGGGAGAG

GAGAAAAGCTAATGGGGTTTAGTTTTGGGCTGGGTATATGTGGGCTTGGGAAAACTTAGGAAGGGTCTTA

GGGTTTTTGGTTTTGGGCTATAGGTACCCTTATCGGATATCGGGTAATTACCCGGACCCGAACCGAAAAC

CGTGGGTCTGCGAAAAAAGGACCCAATAGGGTAAAATCCAATTACCCATATCCGATCCGAACCGATTTTT

CGGGTCGGTTCCGGGTCGAGTCCCTGGGTCCGGTTAAAAATGCCCAGGCCTAACTTCAACTTAGGCTTGG

ATTCTTTTCATTCGAGGAACTCTACCTGTACATGTGTCATTTATCTTTTGGATTTGAATATTTTTGTTTT

TACTCACTGATCGATGTTTACTTACGTTTATGCCTTTGATTATACTAGATTTAGACCCGCGCTACGCCGC

GGTATTTTTTTCTTTTAATTTGTTATATTTTTATTTAATTTTGGTCATCATTTTATTATTTAATATATGT

GAAATAAAATAGTTTGGGCATGTAGCCTGTTTTAATTATCTCTGTGTGAGCTGTCTATTGACTGTGTGAA

TCTTCTTTTTAGGGACGACGTACCGTCTAGAACATCTTCGAGAAGAGCTTGCTCCTCCTCCACCTCTTCT

TCTTCTTCATCATCATCTTTTTTGTTTCTTAATTTTTTGAGTGATTTTTCGACTTTGTTCTGTTTCTTTG

GTTGAACGATAATCGTTTGCTTCTTACTCTCTTTTGCATAAGTTGATGGAAATTTTCGGAGACTGGTTAA

ATGGCGGCGCCGAAAATCAAAAACGAAGACTCGTCATCGAACGGGAATACTGCTTATGCTAAAATCACGC

ACAATTGAGATTTTACGAAAAGACTGAATGGGTCGGAAACAATAAATCAAATCCATTTTCTCCCGCAACT

AATTCTCGTTTAAACTCGACACAGAAAAGAAGAAGAGATTGGCCTCTGAATTCGCGTCAAAATTATTTAT

GCGGATAAAATCAATTTTACCGGTTCGGTGGAATAAAAAAGAACAAATTAAACCAATTTCTATTTCACTT

AAATCAAACCGGTTAAAAAGCTGATGTGTATTAATGAAATGCTGACTGATATTATTTGAAATGTTCCTAT

TGGCCAGACGTTTTTGCTGAGGTGTCAGCTCATCCCTTATTGAAAAAGCTGATGTGTCATCACCTGGATT

GACAATGAGGGTTTTGTGTATTGAATTATGTTTTCATCGATCACATCCGCTATTAAGAGGCTTTGCATCT

TATTAATTTTCCACTGACCTAGTGACGACCGTCGTCAGTTCTCTTTGCTTTTGGCTTTTTGGTGGCGATT

GTCGCTACTCGCTTTCGAAGGTTGACTTGTTCGGCAGTAATTATTATTACAATTTTCTCCTAGGAGACAT

TGTCTCTGTTTACTAATGATCGTCAATTTTCTTTTTTTTTCCGTTTTTAGCAAGCTTTTCATTCTAAAGA

AGACTTGCTGTGTACGTAAGTGTAACGATGAACGTCACTTCTCGTTTTCGAAGGTAAGTGATATGGTCTG

TCACTTATCTTTTTCCCGAGGCTTTGGCTTAGTTTTTGGTGCGTGACCATCGTAGTTTTTACTTCTAGGT

GGTGCGTGACCGAACCTAACATAAACGTCCCATGTTCGACATTTTAATCTAGTACAACTTTTCTAATGAT

ATGTTCTCTTTTTAGTTTAGCGTCGATCGCGGACATTATTTTAGGCTTAGTTCTAAGCAACCATAGTCAT

TTTAGGGTTAGTGCCTTTAGTTTGACAATGACAGTCGTTGCAGTCATCTATACCAACGGCATCGGCCGTG

GTCAGAGCCAGTGGTCACTGCTTGTCACTCTTGTAAGAGGTGTTTCCTCTTATTTGATTGGCATTGACCA

TGGACGTTGCACGTTAATTAAGGCCTTAATTTCCTTTGATTTGTTTAGTAGTGGCCGTTTTCACACTCGT

TTTGAAGTCGTACCTCTTAATTGGCTAAATGTAACTGTTAACACAGATTTTTGTGGCAGTCACAACTTGG

TCTCTAGAGGTCATGCCTCTACTTTGTTTTGTGGTGATCCTTGTCCTTTCTGTTTTTTTTTTAATGCAAT

GCTCATAAATCAGTTTTGTTGTGAGTATAGTAGCTGTTTTATTTTTTTCCTGACCACTATGTATGTGACA

GTAGCCAAGTCAATTTTAAGAGCTATATTGTCTCTTAAATATGTTTTCGTTCACTTAAGACAATACTCCA

AGTCAATTTTTAAGCCTTTCTAGAAAACAAAGATGCCATTTCAAATCGATTTAGGCAAAATATATCATTA

GGAAGCAAAAAGAGGAGATTCAAAATCTAAAGAACATTATCACTACATCTTCTCAAGATGGTGCGCGTAT

GGAGTATCCGAGTGGCTTAGTGGACGATCTGAAGCAAAAACTAATTGATGTGGGTTCGCTAGTATTTTAG

CATAATTTCATTATTTCTCTGGTCCACCAAACTAAAGTAAAACTATCAGTATTAGGTCAATTTATCTTTT

ATTATAAATAATTTGTGTGGATGTGAAACATTCTGGGGGGGGGCATTATCAGGACCATGATTATGAAGAT

GAAAATGATGGATATCACGAAGCTGATGTAGAGAAAAGGGATGAGAAATATGATGAAGTCCCAAATTAGG

CTATATTAGTTGTAGGGTTTATGTAATAGACTAATAGTGACTCTAGTTGGTTCGAAGTGTCTTGAAAATG

GGAATTATAATTTAAAATATCTCCAATGTTTACATTCAATCAACGGTCCAAAATTCCTAATCATAAGTTC

AAAAGCTCACCTGAATTCTGGTTTAAAAGTCAAGAAAATAAGAAGCACCATCTACGTTAAGAGGAAAAGG

AGGAGCAAGAGAAAAAGGCAAGAAAATAAGAAGCACCATCTACGTACTACTGGTTTTGGTTCTATAGAGT

ATAGAATAGTCTTATCTCTTGTGCAAGCGTTTATCAAACAGCGATCACTTGAGGTACGCTACACAGAGTT

TGACCATTACAACAAATGAGAAAGAAGCTACAATTTGTAGACGTAATTTCTTCAAACAAGAATCAAAACG

TGGTATCATATAAACGTGTTATACTTAGAAATCCTTCAAACAAGAAATGCATTGTCTTAGACCAGACCTT

TCTTCTACTGTTCTACATGCCACAGAGAGTTCACAATAACTAAACCAAACATTTCTATATATTTGTTACC

ATTATAAAGGCTACACTGCAGACACACCTTAGAGTTCCGAGCTGCTGTAGGAGTAGAGCTTCTTCCAGGC

CAACCAAATAGATTTTAAAAACCCACATCCATTTGGATTAGTTAACCCATTCCGCAAAAAGATTTGTACG

AATCCTAATACCCTCTTAATCTTTTGTTATCTTCATATTTTAGCATAATCACAGCAAACAAGCTATCACT

ACATATGGATCTTCTTTCCTTTTGTTTGTTGTTTGTAAAAAAATGTAGGTCTCATAAAGAAAAGTTATGA

GAAAATGGATGTTTGCTTAAAAGACCGAGAAAAACATTGTGGAAACAAGGAAGTCGACATTTTTGTTAAT

TATCTTCTCAATCTTGTCAATAATTTTTTTCAACATTTCATTTTGTTAGCCACTTCATTTGATCGAGACC

CATGTCGTGAAAGTAGCAGCTTTGAAACAGTAGGAGTTTGTTTTTGCGATAGAGGATCCTGATGCTGGTA

AAATTGGGGGCTTTATGTTCAATTTCAATCCAGGACGCTTACAGCGTGATATTCCAACTGGGTCATACTG

CTTCACTGTTGACTGTTGAGTATGGATTTTGAATTTAGGTCTCGAAACTGTAGTAATATGTTTCTTTTTG

CGATAGAGGACGGATGATCTTTCTGTTGGTAAAGTTTGTGGATTTTAGGCTCAGTTTCAATCCAGGATGC

TTACAATATGATCTAATTGGTACTTACTGCTTCACCAATGAGTATAAATTTACTCAACAGATAGAGCTTG

AAACTGTCTGCGATTAAGTTTGTAAATAGTAAACACTCTCTGTAAATGTTTTGAGTTTATGAGTCTCACT

TTTAGATTTAGTTGTTGAAACCGTGTCGATATATATATGTAAAGATTGCAAAGGGCTAAATCCTTTGAAA

TTTATTTTCTTTGATTGATACAGGATGCTAGCAGTTTGATAAAACAATCTGATAAATTCTCTGAATACGG

CGAGATTCTTCTCATGAAAGTTTTTGGAGTTCTTGCTGTATGTTTTTGGAGGTCTAGTGCAATTCTCTGT

CAAGCAGTTTTAGTTTTTGGCCTCTTGATTTGGTTACATTACATATATCTTATGTTTGCTTTCTTCGCAA

ACACCGCAGGTTACATACATGTATTATACCGGAAGATTAGAAGTCTTCAACGAGAATTTTCCTGGTGTGA

GTCACTAAGAGCTTTTCTCGTTCCGTTAAAGTTCTTATCAGTTGAGATATTGATAGTAACATTTCCTATT

CTACAATCTTGCAGGCTGACACAAAGCTATCATATGCCTTACAACATTGCAACCCCAGGAGAGACCGGAA

TATAAGGTATGATTGTGGTTTACTCTCTCATTTACCTTACTCTTGCATTGCGTTGCTTAATGAGCTTATG

TTGGATCAGGATGATATTGAAGTATCTGATACCAGTAAAACTTTCGTTAGGAATCATACCAAAAGATGAG

CTCTTGCAAAATTACAGTCTTAATGAGGTAACAACTTTCCTTTTGCTCTATAATATTCTCTTCAATCTAC

GTCAAAACTTATTTCGCATCTGTGAGTGAGATTAGTATCCCTCTGAATCAAACTTCTCTATGAGTCCAGT

ACATAAAGATTGTGCAAGCTCTGAGAAAGGGTGATCTCAGACTTCTCCGCCATGCTCTTCAAGAACATGA

AGATCGGTATGTTTTACTTGTTAGTCTAGCGCAAGAGCATTGTAATTAGCGAGGATTGTAGCTGTGTTGA

ATGCTTTCGTCAGTCAAGTCTCTCTATGATGTGTAGTTTTTTGGTGAATTGAATAAAGGAAGTTTATCGA

TCTGGATTGAATCACAAAAGAGCTCCAAGTCTACCAGAGACTCATGAAGAAAATGTAAGAAGCTTTTATA

TAAGGGCACACAAGCCATTTATACATTGAACAGTCTCAGATTTACAGTACTGTTGCGTAACTCTTTTATC

AATGTTTCAGTTATACCATCCAGAAGCTGAGTGATCCAGCGAGAGCTCACCAACTAAAACTTGAAGTGAT

TGCCAAAGCACTTGGATGGCTAGAAATGGACATGCCATACCATTTGTCGCTGTTGATTTACAAAACAAAT

TTCTGTAAAACTCACAGTTCTTCTGATACTATTTTTCTTGCAGGTGGAATGTATAATGACGATCCTGATA

TACAAGAACCTTGTGAAAGGCTATTTAGCACACAAGAACAAAGTGGTTGTCCTAAGCAAGCAAAAGATCC

TTTCCCTAAGGAAGCCCGTTGGCTCATAGATGTTACCTTCACTTGTTAGCCTGCGAATTGCATCAAGAAA

CCATGTTTAAGCACATGCGTAATCTCAGTATTTGTTTTGACAACTTACTTTCCTCTGGTTACTCTAGTGC

ATGAGCTTAAGAAAGTTAATATCGATCTAATATCCTCTGATCAGGTTCTTGAGGTCGGTGTATTCGAAAA

GCTAGAGCTCAAAAAATTGAAAGCTAAATCTCAAACTATAAATTTAGATGCAAAATGAGTTCATAATAAA

GCATATGGTTCAAAATAAGTTCAGAAATAAAGCATATGGTTCAAAATGAGTTCAGAAATAAAGCTTAGGG

TTCAAACTGAAATTAAAAAGGACCAAATCGCAAAGAAAGAAACACATGAACTTCATAGGTTTTTCTTTCA

TTAGCCTGATTCTTTAGGTAACTCTAAAAGATGATGTTGCTTTTATAATAAAGCAACTAGATCTTGACCG

TGCTTGAGAAGCACGGGATGTTGGAATTTTTTTAAAGATTTAAGTTGTTTGAGTTACACATTTATTGAGT

GTTAATTTTAATTTAGGGTTAACTTAATTATTGAATCAGTAATGAAATAAAAAGTCATCATCTTAATTAA

TTGATAAATTTTTAGGCCACAATAATTCTTATCGGATATGGGTCTAGAACGGAGAAACCCATTAGCATGT

TTTCTACTTCAGATTTGCATTTGCCTTTTTTGCATCCATACCGTTGGTTTAACATCTTGGGCGAAAGGAA

ATGTCGCGCAACTTGGGGTCATGTGAGAAATCGAATCTCAAATCGATTTGATATATACTCTCCTGATATG

AAATGGATTTGCCGACATCAGCTGTTTGGGATTTGATATAATATTCACTAATTAATTAATTAAATAGATA

CTTTCATTTGGGATTTTTATTTGCCTATATCTCAGATTTAACTAATGGATACAAATTTTAAAGCTGAATA

GTTCAAAAATATTTTAGGATCTTCGTAAAACTAAACTTTAAAAAAATTCGAATATACGATGGAATCTGAA

AACATGAGAGCAACACCCTAGAAGTGGGATGAAGGTACTTTAATTGTGGCTAGGAGCAAGTATGTGTATG

TCAATGTGGGCCGTTGAGTTGACTTGACCTTGTATAGATCGTTGATTAGATAACTTATATACGTACGACT

TTTCGATTAACGGTTTGTATTAGGAAATAAGGATATGATTTTGATTTAATATTTGTGGAAGTTCGTCTAT

ACGAGAACATATAAGAATAATTTGGAAATTGACGCAGTCGTGGGAAGTATATGGGTGGCACGATCTTTCA

TACCCTTTAACCTTTACGTCTCTCTTGCTTTAAGTTATGTCGAGCTTTTTTTTTTTTTTTTTTCGAACTA

TTTTTCATATAAGAAATGAAATTGTAGGGCATATCGCAAGTTGGTCAAGGTGTTCTACGCAACTTTTGTT

TTTCCTCTCTCTATCACATAGTTTTAGTAAAAAAAATAGTAAACCAATATTATTATATTTTACAATCGAA

TAATATATCTATCATATATGGATGATCTGATTTATAAAACACAGAATTTTGACAAATGATATATATAAAA

CATAGTTGAATCTACGAACTGCAATAGTTTACTTTTCAAATCTGAATCTAGACCTAATCTTATGAATATA

TAATAACATCAATTTGTTCACATATAAACCAATTTATAACTACCAAGATGGCAAGATATATTCCCAATAG

TTTGCTATTCATATTTGAATGTAGACCTAATCTTCAAAAAAATAAAATAAAATTGTTTGGTCCAACAATC

TTCAAATAAGTTATTAATTATACGATAAAACGATTATACGAATTCACGAGTTATACCAAAGACAGAAAAA

TAAGTAGAGGTCAATGACCACTTGTTTGAGAAAATGGGATAATAAATAATTAGAGCCTGTAAACAACCCA

ACAAAAATTATAATATTACAATTTGTCTGGGATATGCTTTGGAACATAACTCGAATACAACGTAAAAAAA

TTATTTTCGGAGTGAAAAAACACATGTTAATTATTTTCTTACAGAGTATTGGATGATGTAGATCTACGTA

TGAATCTAAATTAAGTTTGATATCCTATTATAAAATACTATCGTGCATCTAGAGAAAAGATTTGTAAGCT

TACAAAACAGTTGTATGCGCCTGTAAGTGAAAGAACGTGCATTCCTTTGACTCGACACCAAATTGACCAA

CCAACCAACCAATCGACGTTGACTTCTCCAGAGATGAACCTAGAAACCGAACCAAACCAAATCGAGTCGA

ACTAACAAAAACACTTGTCATTTTATCAACGTAAGTTTGTTACGAATTTCATATCAACCCAAATGATATG

ATAATTGCACTTAGACCATAAGAGAACCAAATCCAAATTCATAAAACATGGATTTTACACAATTAATTTT

TCTCCAAAACCCAATTGAATCTGAACCGGATTCAGATTGAACCGAACAGAGTCACAATTCTTTTCCAAAG

CCAAATAAACTGGACGAACCGAACCAATAAACGTACAGTTTTCAAACCTACGCGGTATATAGCGCGTGGT

TACTCAATACAATTTCAAGTAGCCAATTATAAAATTACACGTCAGTGATTTAGAAAATTCTTAATTATTG

TAAAATAAAATAAAAAATTCTTATTAATCTCTCTCTACCTCAAAATTTTACTTGTTTATGTTTTTGAACC

GAAGAGACAGACTTCTTCTTCTCCGTCGTCGTCGTCTTCTTTCTTCTTCTTTTGTGTACAGACATACCTT

CATTTTATAGCGAGAACGACAAAGAGAAACATAGAGAGAGAGAGAGAGATCTTAAGAAGAAGAAGAAGAA

ACGAAATCGCGGACGAACAATAGAAGAAATTGAAATTACCCATAGATATCGATACGTATACTCCGGGAGA

TTTATACACGTTTCTTCGAGTGTGTAGCTCTCAGTTGTTCGTTCGCTGAGAGTTAAGGATTTTTCTCGGG

GTTTTGTTTTTCGTTAGATCTTTGGGGTGGAAACAAACAAGCTCGTGTAGACGGAAACTCTCGCCGTCTG

TGGGATCCGCCTCCGTTTTGAAAAGGTTCTATTTTTCCTCGGATTTTTTATTATTAGGCTGTTACAGTGT

TCAATTTTTCGATTCTGGATTTTGTGCGGACGATGAATTTAGCTTTAGAAGAGATCCGATTTGGTTTGCG

GGATGTGAAAAAAACTCTAATTGAATATTTCGATTTTCATTTGATCTCCTTCTATTCTTAGATTGAGAGA

AGCATTTGTTTGACTTAGATGTTTGCTTTGAAGCAATGCGCGTGAATGATTGTGTTGTGTTGTGCATTTT

GAATTGGCTATGAGAAGCTGTGACGATGCTTTGACTTTTCTTTGTTTGGACTTTTTTGCTGTGCTAATTT

GAAATCGTCAATTTATTTTCTTGTCCATGTTTGATTCTTCCATCTTACTGTTGTTATGCTTATAAGCTTG

CTGGTAGGAAACTTGAAAAATGGTGAAATCGTGTTGTTCTGTTCAAGAAACGAACTTTAGAGAAACTATA

TTTTTCAAGAATCAAGGTTGTTTAGTTGAGTTTCACTATTATGTTTCGATTAATTTGTATGGCTTTCTCT

TCTCCATTTCTGTGTTGTTTCCTTGTGTGTGTATGGATTTCTGATATTCAAATATGGTTTTTTTAATATG

TAACTGTTAGGTGCGAAGTGGAATTGTCTCGTTTGAGCTGAAGAAACTTGTTCCTACAAGTCTGAGGTGT

TGCAGTATGCAATAAAGGTTTTGTATGAGATGAAGGCAATTAAAGGGTGGCGTCTAGGCAGAACAAACTA

TATGCAGTCTTTGCCTGGGGCTCGCCACCGTTCTCTTACAAGGAAACCAGTATGGATCATCGCGGTGGTT

TCGCTGATAACAATGTTTGTGATCGGTGCTTGCATGTTCCCTCATCACAGCAAAGCGGCTTGTTATATGT

TTTCATCTAGAGGATGCAAGGGGATTACTGACTGGCTTCCACCCTCGCCGAGAGAGTTTTCGGATGACGA

GATTGCAGCTCGTGTAGTCATTAGGGAGATATTGAGCTCCCCTCGTGTTATTAAAAAGACTTCTAAAATT

GCATTCATGTTCTTAACTCCTGGTACATTGCCTTTTGAAAAGCTATGGGACCTCTTTTTCCAGGTAGATT

TCTTCTCTTCCCAACTTTATTTTCCCCGCCAAAATTGTGTCACAGGTTTCGTTTTGGTTAAGAAGATACT

GATGATAGGAAGTACAATGAAGTCTTTTTAGTTTAGGTTGGGTTTTTATGCTGTTTGGTACTTCTTGGGA

AATGTAGACTTTGTCTAGAAAATCGTTTAAGATCTTAGATGCAGCATATTCTCAGGCGGCTAAATACTTA

GATATGCCTTGTGAACACTTGCAATATGTGTAGTATCTCTGCGTTTGTTTGATTCTTTTAACTTATTTCT

TTCACTGATGGTTTGTTGTCCTTTTATCCCTCCTCACAGGGTCATGAGGGGAAGTTCTCTGTTTATATCC

ATGCATCGAAGGATACGCCAGTTCACACCAGTCGTTACTTTCTTAACCGTGAAATTCGAAGTGATGAGGT

CTGTATTTTTTCTGTGTCTAATATCTTTAGATATCATGTCATTTCTCTCTATGGTCATGACTCATGAGTA

TAGTTGATGGAATATCATGAATTTTTCTTGCTTCTGTAATAAACATGAATCATATTCGTTTTCTGAAGGT

GGTCTGGGGTAGGATATCAATGATTGACGCTGAGAGACGTTTACTGACCAGTGCTCTTAGAGATCCTGAA

AACCAGCAATTTGTTTTACTCTCTGATAGGTAATCTCCCAGAAACTTCATTTCTCCTCGCACTGATTATT

CTGAGGTTTCCTTAACCAATTTGAGTGTTTCTTGTTTTTTGCAGTTGTGTGCCACTGCGAAGTTTTGAAT

ACATGTACAACTATATGCTGTACAGCAATGTCAGCTATGTTGACTGGTAAAGTCATTTCTCCAAGTTGAT

GAGATTTCAGAATTGTAATTACACATATGTTGCCAGATTTCTTCCCGGATTCTCGATACTAATATATGGT

TTTCTCCTACTCTTCAGCTTTATCGATCCTGGTCCACATGGAACCGGCAGGCATATGGATCACATGTTGC

CGGAAATTCCAAAGGAAGATTTTCGAAAGGGTGCACAGGTAAAACCTATTTCACTGATTCAGTAATATGC

CTGCCTCATACTCTGAACATACATGCGAAATCACTAAAATGTCTTTATATCCGTTCTGTGTCAATTATCC

TTCTCTATATCCGCGCAGTGGTTCTCCATGAAGCGTCAGCATGCTGTAGTAACAATGGCAGACAGTCTTT

ACTACTCTAAATTCCGGGACTACTGTGGGGTGAGTATGACCTATACCCTCAAAAATAGATATTTCTTACT

TTCGTTCTTGATTTGATGGAGGTGTTATTATCTACTTTATATGGATATAATGTAACCACCCATTAATAGG

AAGCCTCTGTTTTCTCTTCAGCCAGGTATAGAGAGCAACAAGAACTGCATTGCGGATGAACACTACCTGC

CAACATTCTTCCATGTGAGAGAATCTGTTCTGACCCTTATAACTAAAAATTCATTTTTTGTACTTTCTTC

ATCCGCTAATATACTTTTGGTGTGATAAATCTCTCAGATGCTTGATCCTGGTGGCATTGCTAACTGGACT

GTGACATCTGTTGATTGGTCTGAGAAACAGTGGCATCCAAAGACATACATGCCCGAAGATGTCACTCTCG

AGTTACTCAAGAACCTCACGGTATTATCACCAAACCAAACTGTTATGGGCTCATAAATTTTCAACCTTTG

ACTATTCCCTTTCTAGAGATAATTAACTGATTGGTATACCTTTTGATCCATTTGCAGTCCATTGACGCAG

TCTCACGCGGAACAACTGAGGGAACGGTACACGCTTTAAAACCAATTCATCTCCCACTTGTAACCAGTTG

TGAAATTACCAAAGACTATAATTATCTTTTTTGTTTTGATGTCTGCAAACACAGGGTGAAGAAACATGGA

CACATTGCATGTGGAACGGAATCAAAAGACCCTGCTATCTCTTTGGAAGGAAATTCCACGCAGACACTCT

CGATAAACTCATCGAACTCTTTTCAAACTACACAAGCATCGCATAAACCTTCATGAGATGTTTTGACTGA

TCGATACGGAAGCAAAGGAGGAGGTATTCTTTTGACTCGGATTCTTTGACCAACGTCCCATTACATTTCC

AAGGAACCAAATTAAGAACTTGAGGAACTATCATGGAATGCCTAGAAGATGTTACAACTCGCTGTAAAGT

CCCTTTAAGAGCCGAGAAAATCGTTGTGATCTTGAGAAGCTTAGGAAGAAGTGTTTGGGATTTGTTTATC

ATTTGGTTCGGGATGTTGGTACATTTTTTTCACTCATCTGTTTTTACTACATTTCATTGTTATTATAAAA

AAAATTTCAGAGTGCTTTTTAAATCTCTCTTTTTGTTTGGAACTGGTAATGAATCGTTAAAAAAAAGACA

AAAGTTTATACAGTTTGAAGCTAAGCATTTTGATCATTTCTCACTGAAACTCCACTTTCTGGAGTTCTTT

TATCAGACTAATTATACAAACTGTTATTAAGCAATTTTCTCTTCGTTAAGACAGAGTTCTCGAGAACTAC

ATTCTTATTACTGCATATTACAAAAAAATAATATTACAACACCATTTGTTCATCGCCGATGTCCCAAAGC

CACCACCGGAATCCTCACCTTATGCCCTCGGCTACCTTTCAACTTCACCTCTCCAAAGCTGTATACTCCA

GAAACTGATCTCACGGTCAGTGTGACCGAGAAACTTCTAGCAGCGCCAGGTCTGAGAGTCATTGCCGGTG

GGTTTACTTCAATGGCGATAGATGGCTGCATCCTAGCTGTTATGGTGTATGTTTCTTCTACCTCTGCCAC

GTTTGTCACTCTCCTTGTTATGGTTTGAGTCCCCACAAGGTGAGAAATGGCGATTGATGGTGCATTGAAG

TTTGAAGGATGTTTCATGTTGAAGTTGCAGGGCGTGTTTGTGTAGTTTCTTATCTCGTGTGCATCGATCC

CCGGGGTTGTGCACAAGAATCCTAGATAGTCCTCATAACCTACACAGACAAAATCGACCAGTCTTATTGA

CCAGAGATCAAAAGATCTTTGATATATCATCATTGGGGGAAAACAGAGCAACAACTACCTGCATCAAAGA

TAAGACCAGGATCTAGAGCAGCACTTGGATTGACATGACCACTTCCGTAATCAAAAGGAGTGGCTTTAAC

AAGCGTTACAGTTTCTGTGTCTGAATATTGCTGTGCCTGAAGAGGCCTTCCTGCTCTATCTATGACCGTT

GAGGTTGTCATCAACGCTGATTTGATAGCAGCTGGACTCCATTGAGGATGCTTCTGCTTCACCAGCGCTG

CTATCCCAGCTATGTGTGGTGCAGCCATGCTGGTTCCTGATATCAGTGCAAATCCTTCACCTACTCAAAA

GAGCCCAAAGTAAGAAGAAACTTTGACTTCTGTTCAAAAGAATCTCCACATGTTAGCTTCAGTACTTTGA

GATTTGAATGCTTACCAACATAGTTAGGCTCGTCTGTTCCATTTGGACACCAAGCAGCCCATATTAGATA

ACCAGGTGCAAGAATATCCGGTTTAAGAAGATCAGCATCTTGAAAGCTGAAATCTTTGGTATTAGGTCCT

CTAGCTGAGAACAATGCCACTTGAGGTGCTGATTTGTGAAGTACAGGTTCTAAACCATCTCCGATGCTTC

CCTCGGCTTTAAAGCTTTTTACTCGTCCTGTCCAATCCCTTAATGTACTGACGTTATAGTAATCAATCAA

ATCCTAACGATGCCAAGAAAAAAACAGCATTTCAGATTGAGAAATTAGGCTGAGAGTAAGTGAAAACATT

GAAACTCCCATTACCATTGACTTCGACACATCAGTAATCAGGATTCCTGGAATGGCAGAAGGAACAGGAT

CGAATTTTGTTCCTGGAGAAACATTTTCAACGACAAGAACAAAACCAGCAGCTCCGAGATGCTTTGCGGT

TTCAACAACTTTCTTGATGGAAGCTGTACCAACTACAAAGTTGAAAGAATATCCACAGAGAAGAATCTTC

CCTTCAACCAGTTTCTTGTTCAGAACGTCTGGTCTCTGACAATCAGATGGATTATACTTGGAAACAGATG

AATCCAGTAGAACATCATTTGCAGACACCAAAGTGTACAACCGATGAGGCCGAGTTGATGCTGCAGAAAT

TACAGAACCATTCATCAAAAACATCAAATAGAAACTTGGTATAGAATATAGATCATGCCAAATGTAATAT

AACATTTTTGTGTGTTCTTACGTGATAATCCCATCCCAGCTAGCATTTTTCCGTTTCCTAGGGTCAGATG

GTTTTTGTATCTGCGGTCATCGATTGCAGCTGCGACAGTAGTTATCCAGGGGCTATACGAAACCAAAGTC

TTCGGAAATGGGCCTCCGTTTCCAGCAGCTTGAGCAACGAACACGCCAGCTTTAACGGCTCCAAGAAGTG

TGGCATCGAATGGATTCAAGAACGTTGTCTTGGTAGTTGTTGGAGGACTGTTTGGACCAACAGAGAGGCT

AAGAATATCAACTCCATCATGAACAGCCTGCTCATGAAAATTAAGGTAATATTCATGGAAAGTTAGCAGT

TAGAACAACAATACAACAAGCATATGCAGCTGGAGAAACTTGTGATCAGTTCTTAGTACTCTTACCTGAT

CAATCGCAGCAACTACATCAGCAACAAAGCCTCCAAAAAGCCGGTAAAGAGCCTTGTAAACAGCAATCCT

GAAAGAAACTTAGCCTTTTAGCTTACGAAGGAGAAGAAAACAAAACTGTCAACAAAACTGCCATTTTTCA

TTATCTACATACCTTGCCCGCGGAGCCATCCCACTTGCTTTTCCGAATTCATAACCGTGCATTCTCAACG

GAATACCGTTATTCCCAGCTGCAATGGCTGCTGTATGACTGAAGAATAAAAGAATCAGAATTGCTTAGTA

CAGTGAAATAAGAGCACAATGAAAGGGTCCTATATCACCTCCTAGATCCTGAGGACAATATTAAATTATA

CAGACAAACCTTCCATGTCCATCGCCATCCATTGGCGATGCATAGTCAATATTAGGGTTAAATGCCCCAG

CCGCTTTAGCAGCTTCAGCAAAATGTTGGGCTCCAACGATCTTCCTGTTGCAGAAGCTCTTCTTGGTGTG

AGGATCTTCTTCACATTTCCCTCTGTAATGAGGAAGAGGGCCATAAGGTAGTCTATGGTGAGAGGCAAAA

CTCGGGTGATGCGGATAGATCCCCGAGTCCACAAACCCGATAACAATGTCTTCTCCTGCTCTATCAAAAC

CACCACCAGTAGGCCAAACATCTGTTGGTAGTCCAAGAAACTCTGGTGTATGTGTGGTGAGTCTCCTCAC

TTTCCAATCTTTGTTAACAGATTTCACACCGGGGGCGCGACGTAGTGTCTCTGCCTACCAAATATAGTGG

ATTAGTGTAAGGGAACACGGATAAAGAAACTCGTAGAAAATGATCTTAAATCAGTTTTCCACCTGCTCAG

GAGAAACATGAGCTGCAAATCCATTTATAACGTGTTTATAGCTGTAAAGCTTTTTGTATGATCCTTCCTC

AAAGAGCATCCCAAGAATCATATCGTGCTTCCTCTCTAAGTGACGAGCATAAGATGTCACCAACTCACTG

GAAAAAAGTAACAGATTGTTGATCTAGCAAGCAATGTATAGACAGTTCTGAAGAGGAGAAACAATGTATC

TAAGTACCTTGATGTATCAATCTTCTCATCAGATTCAACAGCAGTTGCTTCAAAACCATTTTCTCCACCT

TTGTAACTTATGATTGGATCTCCTTCCATAGTCACAATGTAGACCTCTGCTATCACGGTAACAAGAAG
