# Supplementary material for: Tandem Quadruplication of HMA4 in the Zinc (Zn) and Cadmium (Cd) Hyperaccumulator Noccaea caerulescens
Source: PLoS One. 2011 Mar 10;6(3):e17814. doi: 10.1371/journal.pone.0017814 (PMC3053397; doi:10.1371/journal.pone.0017814)
Supplement: Data S4 — Fosmid N18P80 insert sequence. (DOC) [file pone.0017814.s012.doc]

**Data S4 Fosmid N18P80 insert sequence.**

>Fosmid N18P80 20090bp

CACGTTTTTGGTTTTAATGATTTTAATAAGAAAGAAAAAATGAGATCCAAATTTTGTATTGTTATTTCCT

ATCCACTGTTAAAAAGAAGGAGCAAAAAGAAGGAAATTACTTGATTTAATTTGTCTTCTTCAAACATTAA

TTGTGATGACATAAAGACGTGGCAGTGATTAAAGACTATGCGGTGACGATGACGGTTGGGTTTTGATATT

TTTTAAAACATGTCCAGTTTCATTTGTGTTGCTATATATGTATGAACTTGCCGGAGATTGTGTAAGATCG

ATGAGGTCAAGAAAAATCTGAAATATAGCTTACAAAATTATCTTTACTTATGAAAGTATTATTTTCATTT

AGTTAGAGAACTTAGGTTGATTATATATATCCTTATATAAGTTCAATAATAGTTCTTTCTAAACCCATAT

TCATACTTTTTTGGAATTGCAAATCTCATACGCTTGCAAACGAATTTGAACTCGATGCCGTGAAATAGAG

TCTACAAAATGACGAAAACAAAGAAACACAAAAACATAATTAGCGAGTTTAGATACTGTTAAAGCTAACC

TAATCTCGGCTACATTCATGCTCATCAATCCAAATATCCAATATGGGTTTTGATAAAGATAATTTCTCTG

ATCGTTTTTACACTTGTAAAGTTGTAAATGAATAAACTTTCTCTATTACCAAAACTATATATAGCAAGTA

ACAAATTCAACTTCTAACTTTCATTCTTCAGCTGAGATGACTATTTTGCCTTCACGTCTTAACCATTATC

TAGCGTTTGTGGTTGTTGCTGAGTCGCATCAGCTTCAACAGTTCATATAGAAAAGACAATGTAACCAAAT

CCCTAGTTTTATGGGATTCACGACGTATGTAAACTTTAAACAAATAAACAAACTACAGCTGATTGTAGAA

GTTATCAAAATAATTAACTAACATGCCCAAATGGGCCAATGCGTCGACTTTACACTCTAAATCATTTTGT

CTGTTAATAAGATGTGATCTACTTAATTTATACTATTAAATCAAATGAAACACCACGAAGAGAAATATAG

ATATCAGACTCAAAACTTGGAACAGTTATTAAAATATTCTTGATAAACACCATGAGGGTGCTCTATTCCT

GTCAAGATGACTTGTATTTTTCGTAATTATCTTTTCTGTTGAATTGGCTTGAAATAGAATTTTATTTCTG

TCTTGTCTTTCTCTATGTATGCTTATATATAGACTTTTTCTTCTTTTCTTCAACGTCGCCATTTGCAGAT

TCTCTTAATTGATCTCCAAAATATGGCCGAGGGAATGTTTAAGGCATTGTTGTGGGGTTTTGCGGCCACG

GTGTTTGCAATGGCCGAGGCAGCTCGTGGACAGCGAGTGCCTTGCTATTTCGTTTTCGGAGACTCAGTCT

TCGACAACGGTAACAACAATGCCTTGAACACCTCGGCCAAAGTTAACTATTCACCTTACGGTATTGATTT

TGCTAGAGGTCCTACCGGACGGTTCAGCAATGGTCGTAATATTCCCGACTTTATCGGTTAGTCCCAAGCT

TGGTTTCTCTGCTTGTCAATATATTTCCGATTGAATTGCTACTATAAACTACCAAAAAGCAACAATATTG

TTTTAAAACAAATAGTAGCATAAGATTTGATTTTCCTAACTTGAAGTTTAGAAATCACGTTTCTTTTATT

TTTGAATAAGACAATTAGAATTAATACTCTCTAATTGTTATATTCAAAATATTCTTTAGAATATTGGAGT

ACTAAGAAAAAATTAACGGAAACATTAGAAGTAATGTTGCAGAATAGTCGTCAAATGCAAATTAAGAGAC

TATTTTAGGACATACATAATTTTGTTTTTTTTAGTAACTTTTTATACGTATTATTAATCTATTCAAGACT

CTATTAAGGAAATAGAAAAACAAAACTTGTGAATGAAATATCGTGAATATCCTCATAACTAAATATACTA

GAAAATAACCTGCGCGTTGCGTGGGACTAAACATATATCAAGTTTTTGTTCATTGTTTTCAGATTTTATT

ATAAAAGTATTTTTAACACTAAATAATATAATATCCTAATTTGTTTAGATATTTCAAAAATTGGTGGATA

TACTATTCACGAAAAACATTTGGCACATGCTCGATATGTTAAATTCACATTTCATCTTCCTGTTTTTTTT

ACCAAAAATTTAGCATGTTTAACCTTTTTTGCTTTTCACTTTCTTTAGACTATAATAATTTATCTCGACC

GTAAAACTTTTAAATATTAAGATCACATTCTCACTTGGAATACGGTACAAAATAATAAATAACTCTTTAG

ATATCTAGTTTTTTTTTGTCGTAGACCACAATAACCGTTGATTATTATATTTTTTTTGGTATTTTAAAAA

CATACATATCATAATATATTAAAATTATCCAACTTTTCGTTCCAGTAAAATTTACATATAATAGCTCGAT

TTCTATCTATTATAAAAGTAAATAAATTGTAAACCTAATTTCTTATTAGTTTTCCACTATAATAAATTAT

ATTCTACATATTTAACATGCAACCACATTATAACATATTTAAAAATGATAAATCATTTATATAACTTTTT

GGTAAAATCTAACTATATATTATTTTGAGTGAAGTATTTCATTTCTTTCTCGCCAACTTATAATACAACT

TTGTTTGATTTTTAGTATCAAGTTATCATATATTTTAAAATATGTAATTACTTAATTTATATGTGTGTTT

TGTTTTAGTTTTTCATGTTTTAAGTATGAGTATATATGAAGAAATTATTAAATATAATTTAATAATTTTT

TATTGATGTGGACACTCTAGATGGAGAGAAAACTCCCTAATTATATATAAGATATGTATATGTTTTCTTG

TGGTACATGAGTTTGAATGGTTTATTTTTCAGCTGAATTATTAAGATTCAGTGATTACATTCCACCGTTC

ACCGGAGCATCGCCCGACAAAGCTCACACCGGAATAAACTACGCTTCTGGTGGCGGCGGAATTCGCAAAG

ACACTAGCCAACATTTGGTAAGAACGGGACAAAAGTAAGAGAAAGAGAGATTAACTTAAATCTATCTTCA

TTCTTTTTACTCGGGTAATATATTGCACTAGTTTTATAGGGTTGACTAAATTTTCTAAACGTACTATACT

GTTTTAGTAATGGTTTAATTGGTTCTTAAAACGTGCTGTGATCATTGTTTCTCATTTATTAGCTATAATG

TTTTGTTAATATAAAACTAAATACTATAATACAAATCTATGAAACATAAGGAAGAAAACATTTATATATC

ATACAAATCTATTAAGTGAATGAATATAACATTAATTGACGAGTTTTTTCTCCAGGAAAGATAACTAATC

ATGTGATTGTCTGATTGATTATCTCAGAGAGGCCTTAATTCATTGGATTTCAATTCAAAATTCTTAGGCT

CTAAATAGACAGCATGCATGAATCACAAGGAATACAAATTAAAGTATTATATAAAAGGTTTAGGTGACTA

AAATGAAACTTAACTAGTTTTCTTTCAAGGGAACTAATCATTTGAGTTTAATTGATTTACTTCTTCTTCT

TTGACAAGGAACAAAAACGACTCGTTTATTGAGTACTGCTTCCATATATATAAAAGACTAAAACTAACTA

CTTATGATATTTCTGATGATAGGGTGATAGAATCAATTTTCAACAACAAATATACAATCACTGGACGATG

ATTACGACCGCGAATGTGCCACCGGAGAAGCTGAACAAATGTCTATACACAATCAATATTGGAAGCAATG

ATTATCTCAACAACTATTTCATGCCAGCTCCCTACATTACCAATGGCAAGTACACTTACGATCAATATGC

TGCTTCCCTCATTAGTGGTTATCGCACTTATTTGAAGGTAAAACAAGGTTCTTAATAGATGGATTATAGA

TATCTCAGTATGATTATTTATTACGGCAACATTTTCAAAATATTAATTTATAATTTATAAGACATGTACA

TATGAAAAATAAACCGAAGAAAACTATAGAAAAATGATGCAGTGGATTGGATCTATCTCACTAAAATTGT

TTCGCAATCATGTATCTTTAAACATATAGTAATTTGAAGATACATATACATATATTTGTAGTCATTGTAC

GCCCTAGGAGCAAGGAAGGTGGCAGTGTTTGGGGTCAGTAAGCTCGGGTGCACGCCGCGGATGATCGCTT

CCCACGGTGGTGGAAAAGGCTGTGCCGCTGAAGTGAACAAAGCGGTTGAACCTTTCAACAAGAAGCTCAA

AGCTCTTGTCTGTGACTTCAACAGAAACTCCGATGCTAAGTTCACCTTTGTAGATCTCTTCTCTGGTCAA

AGTCCTCTTGCGTATGCTGCCTTAGGTACGTAAACTATATATGTTGTACTTTTAGAAGGAAAAAAAAAAG

AGAAAAAAGGAGTTTTGCAAAGATTCATACTTTTAGAAAAACTTCATAAGGTTTTACAAAGATTCGTACT

TTTTAGAAAAACTTCCAAAGGTTTTTTTCACAGATATATATATAGGTTAAGAAATATAAAACAATATGTT

TTGTGAATTGTTATCATTGGTTATTTACAAAAAATAATATTCATTAGAAACAAAACATGTGAACACACAC

ACTTTCATATATATAGTAGATTTGAATAAACCTGTTTACTTTTATATAGGTTAAGGTTTTCGTTTTGCTT

AAATTTCAAATCTACTTTTAGTCACAAAACTGGATAGGATTTTATGATCTTCGTAATATAATTTCAATAA

ATTTCCTTATCACAAATGCTATTTTCGATGCTGTTCTTATAGATTTAATCATATACATATTTCATGTATT

TTTTGTAGGATTTACGGTAATGGACAAGAGTTGTTGTACGGTAGAAGCAGGGGAAGAACTATGTGCGGCG

AATAAACCGGTTTGTGCGTTTCGAAGACGATATGTGTACTGGGACAATGTCCACAGCACTGAGGCGGCTA

ATATGCTTGTGGCTAAGGCTGCATTTGCCGGAGTCCTTACTTTTCCTTACAGTATTGCTTTGTTAGCAAA

GTTATAGGACAAAAAGATATTCACATATTTCTTTTTAAAATATATATGTATGATAACCTTATTCTTTAAT

TCAACTATACAAAGCAGCTAGTATGCAAACATACATGTTGCCTTGTAGGTATCATATATGATCTATTGAT

ATAATTTTTTTCCAAATGCATTGCTAAATTAGTTTAGTATTGGTATAGCTAAGTCAGCTTCATACCCAGA

TCATATATACCAGCTTTTTCTATGGACAAGCTCATCTCTAAATTTTATGCCAATTTTTTGTTATAATTAA

TAAGCACAACAATACAATGTTTACATGTATTGGGTAAATTTATTGTTACAATTCAAGAACCACAACAGTA

GTAGAATACAATCCCTACTCGAATCCTTATTCAAATGAAAATGCATAAATATATGAAGACTTGTGTGTGT

GTGTGTGTATAAACCTAGATATCTTCATAAAAATGTTTCTTGAAGGCCTCCATGTGAGCCACTTGTAGAC

ACGTGGCGAGAATCAGCGAACCGTCTTCATTTTGATCCCGCAATAGCAGAGAGTCGCCGTCTAAGTCATG

CGTGCCTGGTCCCATGTAGACCTCCTTGCCCCATCCGAAATCAATGCCGTACATCGGCAGAGTTAACCAA

CTCACCAGTCCAAGATTAGGGTTTCCATAGAATGGACCTTCCGTGCTTCCCAAGGCATGTATATCTTGAA

ACTTCTTCAGATCCTCTTGGATCTTTAGATATTCTATCCCAACCATCACGTATTCGTTTGTCACATTCTT

TATGGCTTTACTGATTTTTCCCGCCGCGAAACCCAACTCATTTGATATCAATTCACCTATATGCGTAACA

AAATTAACGAAATATAGCTTTTGCATTATAGGATCCCTATATCCAAAACTAACCTACTCTTAAGAAGAAA

AAAAAAGTAACGTACCCTACCTTAAATCCCAAGAAACAGGAAATCACAAATACAATAGCTGAAAATTAGA

CAAACGAACCTGAGGTGCTTGCTGCGACCACATCAAGCGTGGCATTGCCGAAGTAGCCACGTGGCAGAGG

TGGCTGCATCCGATTGCGAATATCTACAGAGATTCCCACAGACGCGGGTTGCTCCGGCGAGTGCCCACGT

GCTTTACACGCGCACCTCCATACGTGTCCCGAGACCGTCTCGTACCTTGTAAACCCTCTCGCTGGATCAG

CGTATTTACTTGTATTTACTCTACTTCTAAGCTTCTCAACCTGAGATTTGCTCAGTTTTAGCATCGCCAC

AACCGTTTTCTTCTTTCTTTCTTCTCCATTGTCCGTTTTTCCGATCAGAAATGGAGGCTGTTCAAACTCT

TCGTGGTCGAAGTTAGGTGGTGACTCAAACGGCGGAAGCGGTTCATCAGCCCAGAGGACTCTCCGGTCAA

GAAATGGAACGGTTTCTAGCGGTTCACCGCGCGCGATTCTTCCCCATTCCCTCATGAAGTGAAGCGCACT

TTGGCCATCGACCACCGCGTGTGAAACGTTGACGCTGAGGCTCAGTCCGCCGCATTTGAATTTGGTGACC

TGAGCTAGAAAAAGAGGAATCGTTTCGATAGGGTTTTTGTAGTTTACTTGCGGCATAAGTTTCTCATATT

CCGGCGTCGGGCAGAAATCGTTGAAATCGGAAAGCTCGGCCTCGGATTCCGCTTCGATGAACGTCACTCC

CGCGGCGTTACAAATGAGCTCTAACCGACCCCGTGGAAGCCACCGGAGACGTCCTGCCATAGGGTAGAAA

TAGAAAAGCGCACGGCTCAATGAATTTTTTAGGGTTTCGACTACATTGCCTTGGAATGATTCAGATGGCT

TGTCGTAGAAGTAAACGGTGGGAACGTGAGTTATTGTACCAACTTGATCCCTTTCGGCTAATGGATACCG

ACCGGTCCATGTTGGTTCAGCCGGTACTATGGTGTAAGTATTTCTCAAGACGATGGGAGCCATTTCATAA

ACCGTTTTCTTTTCTTTTCTCTTTTCGGAATTCGTTGCAGAGTATTGTTTATATAATATCATTGAAATCC

ATGGGTTTATATCATAAGTTTATAGTACTTAATTAAAATGTCTTTCTCGTGGTTTAGTTTGTTTCCTTGT

AAACAGCTTCTTTAGTGGAAGTGTGATAATAAGGTTTCTTTTTTTGGTTATATGATCATGCTTCTTGTCC

TTTTTTTTAGTTTCCATTCGCAAAATGATGAACATTCCCATTTAAAAAAAATATAAACTTGTCTACTTTA

ACCAATTTGTCACGAGTATTTCACATTTACAATTAAGAATCTTATAATATTAATACCCAATCTAGATATA

TAGGTAATGTATTAAAATTTTATAATTGGTAAAAGTATTCAACTTTCACAGGTTTAAAAAAATAAATAGT

CTGAAATTTCAAAATTTCAGGTTACAAGGAAACAAACACTCGAGTCATCGTGTTGAAAAAGGTAGTCCAT

GGGCTATACATAAAGCCCAAAGTGGATGGACTGTTAATAAACCCTAGCTTAAACCCGACTGACTAATGTT

GACCCGGACTCGAACTGGATCTTCTCCTCTTCTCGCCAAGTTCAATTTGACGATCAAAACAAGTACACCA

CTATTCCCCCACGAACCAGAGCTCGAAGACGACAATGGAAGGCGTCGAAGAGACGGCGAATTTCACTTTG

GTGGCGAGGAAGCCTTGCTTCGGTCTCCCAACAGCTTGCCCTAGTTGCCTTCCCGCTTACATATACCTGA

AACTAGCCCAGCTTCCTTTCGAACTTGCCTTCAATTCGATCTTCCCTGATTCAGGTTCGATGTGTTTTTG

GGGTGCTTGGTTGTTAGAATAAGTTTTTTCGTTGCGTGATTGAAGTGTAATGCTTGAATTGAGATTCGAA

TCTTATACAAGATAAGTTTCAGCTCTGGATTCGAATTCGTAGCATTAAGCTCTTGCTCGTCTATTAATCA

GAGTCTGAACAGGGCTTTAGGCTATAGTAAATTGCTGTTTGAGGTTAAAAGGATTCGTTAAGAGAGAAGA

AGACATTGGTGAATTTTGTTCTCGCTTTGGTTGAATTGTTTTTCTAAATTAGAAGAGGTAATAAGTTTGT

ATATTAACAGAAATTTTGTGTACGCATTGTTCAGCTTAAGATTGTTCATTGATTTACAAATTTGAGCTTT

GGATACATTCTCATTTGTGTATTTGTGGTTTTTATGCAGATGAACTTCCGTACTTCGAAACCGGTACATA

TGTTGCATACAACAATGAAGATGGAGGAGTGATTGAAAAACTGAAGAAGGATGGTATTGTTGATCTGGAC

TCTCAGCTCCAGTCTCTTCCCGATTATCTATCGTTGAAGGCTCTTATCGTTTCTTGGCTGGAAGAAGCGC

TTACTTACGAGCTATGGGTTGGGACCGAGGGAATATCTGCGTCGAAAATCTACTACGCAGATCTTCCATG

GGTGATCAGCAAGGTCCTGTTTTATAAGCAGACGTACATGGCCAAGAACCGTTTAGGGATCACCAAAGAA

AACGCAGAGGAAAGAGAGAAACAGGTAGCTTCTATTTTTCGAGCTTTGTTGTTCGTATGACAGTAAGTTT

CGTTTGCTGGTAAAGAACACATGTGGTTCATATTTGTTACTGAAGCTTAAGGTGTTAAAATGAACAGATT

TACAAGAGGGCTAGTGATGCATATGAAGCTTTGTCGACTAGGTTAGGCGAGCAGAAGTTTCTCTTTGAAG

ACAGGTACTGCAAGCCACATTGACCAAACTTGATTCTTTCTCTTATACCTTTTTATGATAAGATTGATTG

ACTATGCTCTTCGCGCAGGCCATCGAGTTTGGATGCTATCTTTCTCTCGCACATGCTTTTTATAATCCAA

GTTTTACCGGTAAGCAGCTTTACAGAATTCAACTAGTATACTTTTCGCTTATTGGTTGCCATCAGGTAGA

ATCTATGCTTTCCTTGAAACAGTGTAGACTAAGTTTATTTCCGTTATGGTTGCATGATCAGGAAACATCA

GTGCTTCGGTGCAAACTTCTGGAACATAGTAATCTTGTCAGATATGCTGAGAAACTGAAGTCAGAGTTCC

TCGAAGCCTCTTCTTCATCTCCTTCGCCTCCGCTTCAATCATTCCCTTCCCCGTTTTCAAGAAAGGGTAA

GAAGACACTTCCAAACGTTACATTTCTCATGTCATGTACTCTTTTCAAATCTCATGAATTTCATCGGTTT

GTTTAGGTTCGAAGCCAAAGAGCAAACCAAAGACTGAAAAGACCGAAGAGGAGAAAAAATTCAAGAAAAG

AGCAAGATTCTTTCTAGCTGCTCAGTTCCTAGCCGTCCTTATTTACTTATCCGTCATGGGAGGAGGTAGT

AGTTCCGATGAACTGGAGTATGAAGATGAAGATTACTAAAAAAACTGAAGCTTCTCTTAACTCTCAGGCA

AACTCTTTGACCTCTTCTTCCTTAGACTAAGAAAAAACAGAAACCAATAATTTATGTGCTTTGTTTGTTG

AAACTCTTGAGTTAGAGAGAGGATACAAATCCATGTTATATTGATTTTTCGTTCTGAATGAGAGATTCTA

TTGGCTTTTTATACAAATTTCATTGGTTTTAAGGCGATGAGGCTCGCAAGTTGTCACATCAAGAGAACCT

GTTAACCGGATAAATTACCGAACTAAACTCTATTTGTCTTACTAATACAAATCTCATTCTTCTAAAAACT

ATCTTCTGATAGCAAAAGGTGATGCCCAAAAACCTACCTAAAGCTAAACTCTTCTAAAACCCAAATAATA

ATCAAATAACAATTAGGAATTCAAATTTACATCCGGAGGTTGCTCCGGCGAGGCGTCCTCCGGTTGGTTA

ACAAGCTTTGAGTGTTCATCGGCAGAGTTGATAGCAAACTCAGCCAGTGAACTCACTGCAACAGACATTC

CATGGCAGAGAACTTTAACAGCAACCTCTGCAAGTTTCTCAGCAGTCATGACAACATCTTCTTCTACACT

ATCAGCCACCACAAGGTCCATGCTTTCTGACCCGGAATATCTTCTTGACCCGCTTCCGCTCCAGTTTGGA

TCTTCCCTAACCTGTCTTGCATAGATTGAACCCATCCCGGAGGCAAAAAAGTCGAGTTTGTCAAGAACCG

GCTTCTCGTTCAAGCTCTTAAGTAACCTTGACCATTGGATGCAGACTCTATATATCGGATGAGGACATGA

GGATAATCTAACTTTCTCAGGGTCAGGATCACATCTGAAACATCTGAGAAGCCAACCGGATAATGCTGTC

ATGTAGGATCTTTGAGATGTGATCCAGAACTCGAAACAAGCTCTCCAGTTTCGAAGCTGAGCTTCAAGAT

TCAATGCAGATTGAGCTAATCTTTGTGAGTTGATGGCCTCTGGCATGATTGGAGGTTGTCTCTTCTTGTG

CCGCTTCGAAACTGGTGTCCCTGCAAGTAGCATTTTAGCTTCGTCTAGTGTTCGTTTCTGTATCTGATGA

CTCTCTGCCATCACTTGCCACATCTTTGTTAACCTGTAATGATCATATGATTACCATTTGAGGAGGGTTT

AAGAGAAGAAACCAAAGAGGAAGAAGAAGAAGTTGTTGGTATTACCCTTGAACAAGCTCGAGAAGCTGAG

GCAACAGTTCTTGATCGCGAAGAGTCTCGATCCTTTTGGAGATAGATTCAATCGAGTGTATAGAGACCTT

AATCTGAGTATCTAGATCTCTGAGTGTAGCCCTTGTTTTATCAACCGAAAACGGATCATCTCCTTTAACA

TCTTGATTCCTCAGCTGCATACATTTCTTCTCATACGCCCTTCGAACACGTTCTCCAGACTGTAAACAAC

ACCAATAAAAAGGCCATTCTGTTCATAAACAACACAGATTTTGAATCTTTGTTACTCAAAACTTTATCTC

TTTGTTACTTACTTTAACTTCATCGTAGAGTTTCTTCTCCCAGGCGAAAAGCCTGTCCAATGTTGTTTGG

TGGCTACCTGAAATCATGCAAGATTCATCTGAAACATCGCTTCTGCTCTCAGATCCACTCTCCTTCGAAC

CACCACCAGAAGAAGTGATCAAGAATCTTGCAGATGAAGATCTTGACGAACCCGAACGGAACAAAGCTAC

TGGATTCAGCATTTTCATAGCTGCAAAAGATAAAACTTCCTTCAACATTCAGTGAGTGAAAGACTGCAAA

AAAAAATTCAAGAACTTACCACTATGATCATTAGAGGATGGTGCATACTGAGCTCTACTAGCTTCTAGCA

ACCCCGAGACTTCTTTAGCCGCGTCGCAAATAGTTGTGAACTGATCTTCAAGATCTTTGATCACCTCTGC

CATGCTCGTTGGTCTTCTGTTCACATAAACAGTGAAACCTGGAGGCGTCTCTCTTTTGGCAACATCAACA

ACATTCTTCATCTCTTGACCACTCTTCTTCACAACTCCAACAACATTCCCTCTAGGCACCTCAACACTTC

TCTGTTCTTGTTCTTGCGTCCCAACGCATTTCTCATCTCCTCCTTCGTTCTCACTTTCACAACCACTATC

GGTAAACTCTCCATCCTCATCCTCATCATCCTCGTCCTCATCATCATCATCATTCTCATCAACATCTTCA

ACTTTCACTTCTTCCTTGCAACAACTCTTATCGACTTTACCTCTACTCTCTTCCGCTGCTTTCGGATGAT

TATGATGCTGAAACCTCACAGGTAGAGGTTGTTCAATAGGCTCATCTTCTTCCAAATCTGGAATCCCTTC

TTCCTCACGAACACGTCTCAATCCTCTAATCTCATCATCCATTATACCACCACCACTCCGATTATCAACA

CTTCCTCTATCATAACTATTATCATATCCATAATAATCCAACGAAGAGAAGGGATTCCAGAAGAAGTCCC

ACTGCGAATTCTGCGGCGAAGGAGGAGGAATGTTATGACTACTGCTGCTTAATCTCTGCTCAGGAGAAGA

GCTCAACGGATTCCAAAACGAAGACGAAGAAGCTGCAGAAGTGTTCATGTTCATGTTCATGTTCATTCCA

AAGAAACCATCTCCTTCTCCTCCGTATTGTTGACGAATCGGAGGTGAAAATGACTCAACACGAAACGTTT

CTGCAGATCTCTGTTCGACGCGAACCGGTCTGCTTCTGTTCGCCATTAAGTAACTCGCTCTCACCTTCGA

TCTCGGCTTATCTTGAATCATTTTAGCTGGATGTGGAGGCATCGAAGAAGGCGAAATCGTTATGAAATCG

CCGCCGTTGCTCCGGCTGCTGCTGTTGCGGCGGCGGCTCGGTGGTCTTCTCTTAACCGGAGTGACGAATG

AGTCGTGATGACTCGGAACAAACTCGTGAGGCTTGTAGTTGTCTCCTTGGACAAAGTCATGGAGAGCATC

AGAGACTTTTCGAAGTGATTGGATATAAGCAATGTGACCAGAAGCAAACTTGGTTCTGTGTTCTATCGCT

TGTTTGATGAATCGTTTCCTGTCTTTACAGATCTGAACAGCTTCTTCGTCGTCTAGCTTTGAATGAGAAC

ATCCCATTTCTCAGCTCTTCTTCTTCTTCTTCTTTTTATTAAGCTTCAAATCCACAGATCAAGCACTTCA

TATCAGTGGATAAATCGACATAGCTATTTTCTTTTAACTCACATTAAATGAGTAAAAGACATTGTTTGTC

TCGATGTCTGAAGAAGAAGAAGATTCTTTGCTTAATTTACAAGACAAAAAGCTCTTCACACAGTCTTTCT

TTCAACTCTTAAACCCTCTTCAAGAAAATCTAAAACCCAGTGCACAGATTTTTGCTTATTTCCTTTAAAG

AGACTGAAAACCGACAGAGACCCGGATGAGAAATAGTAGAAAACAAAGAAAATCTAATCTTGGTTTTGTT

CTAGTGGAGTGCCCAAAAAAAAAGAAGCAATGAAACAAATCTGGTAAAGAAGACTTAGATTAGAGAAGAA

AAAAAAAAAAAAAAAAACTAATAGGGTTATGATATGGTCAAAGAAGCGTGAGGATTACTGTGGAGGAAAG

AAGAGACGTTGGAGAAAGCAACGGTCAAAATTACTGTCGGATGTCGAAGGAGAGAGAGTGAGAGACTCTG

AGAGAGAGAGAGAGAGAGAGAGAAGTCAAGAAGGAGAAGAAGACAGAAGCAAAAAAACATAAAAACAAAG

GCAAACCAACCAATAACAAATTTTCTAAACAAATACATAAAGGAAATGGAAAATGGAAAATGGAGGTGGG

AGGAGAGGAGAGCTAAGCTAATTTAAAGCTACGAATAATTTTATAATATGGCAAAGACGAGACGGGACAC

GGAATACGACTACTTGTTTTGTTTATTCACCCTCGCTTTTCACATATATTTTCGGTATTGCCACTCTCAA

ATTTTATTTTTTTCCTTTTTTCTTGTCTTTTTCTTGTACTATGCTGATTTTTTTATTTCATTCTTGCACG

TTGTTTCATGGCATTATACAATATAACACTTTATATAAAACGTCATAGATCAAAATTTTGTACTCATAAT

AAATTTATAGACCTACCAACAAGTACAAACTTTATGTAATTGTGTTTTGTGTTTTCCTATTTGGGTAATT

ATAATACAAAGCATGGGCATTTATGTAATTTGGTTAGCATCTGCAGTGGGAAAATACGTTGGAAGAATTT

AGGAAAAGCGATAAAAAGGAAGGACCGGCCTGTAAAGTAAAAGGCAGGATCTGGACCCACACTTCTAAAA

GATATCATGAAGTGCCTCGATGCCTGAGATTTCTCTTCATGTCCCTCACTGTTTCACTTAATCACGAAAA

CACCAACCTCTTTTATTGGGCTCACGTTTACATTAATATCTGACCGAATGGACCTTATTATCATGAATTG

GATTATAGAAATAACCCATAGCATAGTTTGTTTTTGCGTAAACCTGAACTTAATCCAGGCCCAATCTATT

TTATTGGGTCTAGAGTGTCCACTGACCAAAATTTTCAGACTCGTATTTTTCCACCGAAAGAGTATGTCGT

ATAGACTCTGTTAAACACTTAAAATCAAGAAAAAATTGGTGAAGTAGGCACATTTTAAGTTTCTAATTGG

GAAAAGAGGCAACACCAAGTTTTTATTAGCAAAAGAGGCAACTTTCTCGTGGGCCCCGCCATGTGTGGTT

GGTTTGGGTTCACTTTTTGGGACTTAGGATTTATTTTTTGTTCATGGAAAAGTGTTTTTATTGTGGTGGG

ACCCACCGATTTGCCTCTTTTGCTAATAAAAACTTGGTGTTGCCTCTTTTACAAATTAGAAACTTGAAAA

CATCCTCTTTTGCTAATTCACCCTAAAATCAAGGTCTCTCCAACACTTAAAGCCTACTCTAAGCTTTATG

ATACTTCCTTTAAGTTTTCATAAATTTTAGATTCTCCATGCTCTTAGCCACAACTTTTTTAAGATCTATT

AGATTTTTTTTTGGTTAAAAGGCCTAAAAAAAACAAAATTTGGGGGTGGGAGGGAAGGCGAGGAATCGGT

TAACCGCAGACACTAAACCTAGTTTCAACAGAACCACCTACGAACCAACCCAACCCAATGGTCTACTTTG

GAGATGAGTGTCTTATGGAATCATCGATAGAGTATCTTTATTAAAAAATCACCAATGACATACGTAAAAA

TTATCTCCGAATAATGTAACTTCATTTTTTCCCTGAATTAGTGCTTGAATCCAGCAATTTCATTATAATG

GCATTACCATTTAGGTCTCTGGTGATGTTTATGTTTGTATCACCATTCATTAGTTATATAATAACATATA

TAATGCTAATGTTGGGTGTTGATAGTATGTCCACTATATAACTTCTTCGTGTTTCTCACTGTGTACATAT

CATGATAAGAAACTTGTATCTATCTTTGCTCAAAAAATAAATAAAAAATAAAAACTTAATCTAGACCTTG

TCCCCATTTTAATATTATACACTTATTCAAAATCTTTATATATGTGTAGATATATATATATATATATATA

TATGGTTTATTTATATACTATTTCAAACAAAAAAATTGTTACAATTAAATGTTTTAACTTTTAACATGTT

GGTGCCAGGAATTCAAAGTCATTATTTTGATGATCTATATATGTTTCTTGTAATATGCGGTTTCAACAAT

GTTGGAAGTGAGACGGAAGACTTTAAAAGATTTGTTTGGAATAAAAATCTCGCCATAATTAGAAAAATCT

TATCGTATGGTTTGAATTAAACAACTAAGATTTATTTTCAAGATAAGTTACAAACATAATTAAAAAAATT

GGGAAGCTAATATATCGAAGATAATTAATGCTATTTCAATGTTGAGTTGCGAATTTAAGTTCTAATTAAG

AAGAGAATTCACGTTTTATAGAACTGCCGCAATTTTTTTTTATCTTTCTACCTGATTATTCAAGCACCGA

GCATAAGTTATGATCTTGTGCAAACATGTTACTAATTTAATAAGATGTATCAAAAATATATAGATACATC

TTAGAAGAAAAGCTAAGAGAGTAGACGACAATTAATTGGTGCGTTGCGTTAAAAGAATGTGCACATAGTT

TAATTAAAATTTTAGAAATAAACTAAGAAAATTGTACTAGAAACCAAATAAAGAAAGCAATTAGATGAGG

AATCACACATGGATTCCATTTTGTGACATTACACTTTTGGTGTTTTCCTACTAACATTTTACTATTTTAG

TAACTTTAACTTCGTGTCTCTCACTCACGAGATTAAATCCCCTTTTGATCAAATTTTCTGCTCAATTCTT

TCTTTAGATAACTAGCAAGAATCATGATTATAATAATTCCAATTCTTAGCATGCGATATTGCGAGGATCA

TGTGTCTAAACTAGCGATGTATCGGACAAGTATTATCCTCGCCCCATATTCAAACTGATAATGTTTACTT

TATAATCTCACTCTTCTTTTGTAACCATTTTATATAAAGTTTTAATAGATATTTACCATATTTTTATCCC

AAAAACTTAGAATATGTAGTTCTTTTGATAAAACTCTAATTGATCATCTACTCCATAAAAAGCTAATGTC

GAAATTTATAAAACAAAGTCACATGCACAAACAACTGATCTTGTCAGTGAGAATGTTTTTACTTATGGTT

CAAATCTCAAAAACATAGCCAATTCAAATTTTATGAAGTTCGGTAGTACTATAAAATGAGCAATCAATCG

TTTATAAAAAGGAGCTAGATTAGACTAGTCTATAATCCATTATAGTGAAAACCGTTACCACAAAATATCA

TACTTTTATTTAGTGCTGATGTAATCGATTTTAAAATAAACTTATAATTTTCTATTCTTGGAAATTAATC

ATATGAACTAGCCTGAAAATTCGGAAGGAATATAGGAATTTAAATCAAAAAATAAAATATATGTGCAATC

AATAGTTGAGAAATAAAAATGTACAATTTAATAAAATTCAGGAATAAATTGAGTGTTTTCCCCTAAATAA

ACATGGTAAACAAAACAAAAAAATAATGTAAAAGAAAAGGTTTTAATTCGAAAAGAATCCAAACTAACAA

CAACTTTAGAAAAGTTGCTTTTATGTTTCAAAAGAATCCATAAATGTTAGGTTTCGAACGGCTTTTAAAT

ATTTAATAACGATTCTCAAATCTTTTAGGGGTGGGTGTATTAAAATTAGAATTTGGAGTTATTTGATTTT

TAATGGGGTTTTAGATGATTTTAGAGAGAATTGTGAATATACCCTAAAAAAAGGCTCATTTTGTCTATTT

ACTTCTTTTTTGACATTGGTAAATTTTTGCCAAAATTGTCGTGTCCTTCACTATAATTAAAAACTGATAC

TAAATTCTTTTACAAGTGAACAAAATTTCTAAAATTCATAAAAATGATCTAAAGACTGTTTAAAAAATTT

GAACATTTTTAAAATTGTTTTTCATGTTTCAGAAAATTATATGAAGTTTCATTCTACCAATCTCTAAACT

CAAAGTAGAATACGTTTTCTACTAGTTACTCGGACGTCCAACGACTATATAATGCAGAATCTACAGCTAT

GTCATACTTCTACCACAATTAGAATTCGAAATCTACCAAATACTATAGGCTCTACCATTTGGTAGAATTT

GCTCCCAATATATTCTACAGAATATACTATCTTCTGCTAATCGTGGATTTCAAGTTCTACTCTAAACACT

GACATCTACCTTTCGTAGTTTTCGTATCCTACGTACTACAGTTCATTCTACCTCCCGCAGAATTAGTGTT

CTACTAAAAGCAAGTTTTCTACTATCCGTAGTATTCGTGTTTGTACAAGATGTTCTACTAAAATAAGTAT

CATTATACCTTTTGTATAAATCATCTTCTACCAAAATCGAGAAAATATTTCAACTGAATATTTTTCAAAT

CTTTGTTTCTTTTTTTCCTCTAACTATCCGAAAATAAACCAAAATGGTTTAATTAATTTTTAATTTTTAA

TAATGAAAATAGTTTAATTAAAAATAAAACCTTATCTAGTTAATTTGAAGGACAAAAATGGATTATGTGA

TAAAAAAGGTGTAAGGGGACAAAGAGCAAATGTGAAAGAGTAATGGGACAAGATAGGTTTCTTTTAGGGT

ATTTTGGCAATTTTCTCATGATTATGTGAATTATAGAAATTCATGTGATTTTGGTTAAATCATTCTAAAA

TCTTATCTAAAACCAAGTGATTTTGAGTCTTTTATTTTTAACAAGAAAATCTCACAAAATCACTCTAAAA

TCAAATCTAATTTTAAAAATCTACTTTTAAAAATATTTCTAATAACAGTCGATTTGAAAGTGGATTTTAA

AATCACAAATTCAATAACACTGAATTTTAATAGGGTTTTTAGAATTCAAGTTTGAATAACACTAAATTTG

TTATTTTAATATAAATCACCGTAAATCACTTCAAATCTCAATTTAAATACACCATTTCCTTTCTTAAACT

TCTAAGAACCAAATTTGTGGTATATATTTGATATATTTAATCTATTTTTTTCTTCCAACTTTGATCTAAT

TGAATTTTGTTGGTGATTTTGGAAAAACAAAATATAAATTTCGGCACTGTTATGGGTCAATCTCAAAAAA

GGTTTTCCTACCAAACTCAAACTGGAATAAACACCGGTTCCCGGTTAGACCGGTCCGACCAGCCGGTCCT

ATCTGATTTTTTAATTATTGTTTTTAGCTATATATATATAAGGGACTCATTTTTGTTGAAAGAAGATAAA

GTTAACAAAAAAACTTTTGCCTTCTCTTTCGATCGAATACTATCTCCCACTTTCCTTCTCTCTCTTTTAA

TATCACACCTTTATATATAATATTTTATAACATAAATAAAAATTTTAATAAAAAGCTGATAATTTCGAAA

ATGTTAAAGAACAACAACCAATCATCATCTATCATCTAACATTCTCACTTATCTGCAGAGGCGGCTTAGA

GGTCAATAGGTGCTCTGCACTAGGGACCTAAGGAAAAACAAAATTTTAGTATAGAAAAAAATATGGAGAC

AAAAATTAGTATAGAAAAAAATATAGAATCTTTGATTAAGATTATAGTTTTGATTTTGCAATTAAGACTT

ATAAAATCTTTGACCCGGCCCTGCTTATCCGGCTATATAAGCAACTACCATTTCTAGATATCTTCACCTC

ACAATCTTCCTCTCTTCGTTCCAAAACCTCTCTCACTCTCAGTCTTCACCTTTGTGGTAATACTTTAATC

TGGTCGAACCGCACCAAACCGGTCCGGTCTTTCTTCTCGGCCTCGTCTTTTCTCCGGTATTCTTTCTCTT

CTTAATTCACATAGATTTCATAACAAGTGATTTCTTCGTAAAAATTAAAATCCGATCAAATTCACGGTAG

TGATATCTCCAACACGTTATATGCATCCCAGCATAAAAGTTTTTCTTTCTTATTTTTTTTTCCCCTTAAA

AGATTTGGAAAATTAACCATTAATCCCATAATAATCTCTTTTTGCGATGTGATTTGTTTTTTTCTTTTTA

GATTTCCGTTTCACAGATTCGCCATTAATCCCATAATAATCTCGGTTTGTTTTTTTATTTTTAGATTTCC

GTTTCACAGATTCGCCATTAATCCCATAATATTCTCTTTTTATAATGCGATTTGTTTTTTTCTTTTTAGA

TTTCCGTTTCACAGATTCGTTAATCATAAAAAACTTTGATACAGAAATGGCGTTACAGAAGGAGGACAAG

AACAAAGAAGAAAATAAAATGACAAAGAAGAAGTGGCAGAAGAGTTACTTCGACGTTTTAGGAATCTGTT

GTACATCGGAGATTCCTCTGATCGAGAATATTCTCAAGTCTCTCGACGGCATTAAGGACTATACCATCAT

CGTTCCGTCGAGAACCGTGATCGTTGTCCACGACAGTCTCCTCATCTCCCCGTTCCAAATTGGTAAAGCA

TTAGCTAATCACTTTCTTCGAATTTTTATTTTTACCTAATAAAAATAATTGAATCAAAAACCATAAAGTA

ATCTCACTTAACACGTAAACAATCACTTTACTTTTCTTCTCTTTCTGTTTTCTTCAAAATTAATTAATGG

TTTCGCGTCCTCGTTTGATACGCAAAGCCTCAAATTAATTTTTTTTTGGGAACTAAAATTACTCTATCTA

TCAGATTTACCATAAAAGCTTACTTTGACTTTACAAAACATTTATTAGCAAAATTCGTTTATCACCAACC

TATTCAAGATTTAAGGGAAAATAGTTATCCTCAAAACTAGGGAATTCAGATTTTTGAAGTTTTTAACGAT

TCTACTGAAAAACAAAAGCCCTATTATTTGGGTTTCTTCTCGAGAAAAAATAGAATATTGTTGTTATGGA

TTTTTTTTCATTTTTATTAAAATTAAAAGAAAATTCAAAAGTTATTTATAAATCAAGTTTTTTAAAGCTA

TTTTGATGGATTGTTTTAGGAAAATTGATCTAACCAACAATTGTAATTTTTTTTTTTTTGTGTGTGTGAT

AAAGTCTACTTTTTCAACATTAAAAACTAGAAATTGAAATTTACGGCTTCTTTATACAATTTTGCTCGAG

CCAGCATCTTTGTGTATAAAACTTTGCATAACTCATACATACCACATGTGACATGTCACGTGTGTACTGT
