# Supplementary material for: Tandem Quadruplication of HMA4 in the Zinc (Zn) and Cadmium (Cd) Hyperaccumulator Noccaea caerulescens
Source: PLoS One. 2011 Mar 10;6(3):e17814. doi: 10.1371/journal.pone.0017814 (PMC3053397; doi:10.1371/journal.pone.0017814)
Supplement: Data S5 — Fosmid H2P47 insert sequence. (DOC) [file pone.0017814.s013.doc]

**Data S5 Fosmid H2P47 insert sequence.**

>Fosmid H2P47 20258bp

ATAGGTGTGTGAACATATATATATATATATATTCAGACATATAACATTAAAATATGTTACACAAGCTTCG

ATTTTCACCTCTGATACAACAATTCATCCACATCTTTATTATGTCCAAACAAAAGTAATCCACGGCAATA

AATCTACATAATTTGGCTCTCTTTGATTCTTCCACTCTTACTTTCATCTTTTTATTTCCGTAATCACAAG

CAACAATGTTTCATTTTCACTTCTCGTTATCATCATATATTACAATTTTTTACCCATGTTAACCCTATCA

AAACACCAATTAGATACATGAAAACAATTCAATCCCACACCAAACCAGAAATCGTTTTAATTTATAGCTT

CAGTTTCAGAAATTAATATACGCCAAAAACGTTGGTTACTGTGAAACACACACCAAACCAAAAATCACCA

TCACAGAAATAGCCAAATTTATCCACAATTGTATAAGAAAAAAAAAAAAAAAGTACTTTGCGTAAGGAGA

GTTATAAAAGTAAATTTCTCGTACACAAGTGTCACCAAGTGAACATACATTAGTCAATGTTAGTATTTTA

ATATTCTCTTTTAGCTATATATAAGGGACCCATCTTTGTTGAAAGAAGATGAAGTTCACAAAAAAAACTT

TTGCCTTCTCTCTCTATCGAATACTGTTATCCCACTTTCCTTCCCTTTTCCTTCCCTCTCTCTTTTAATA

TCTTCACCTTTATATATAATATTTTTATAACATTAATAATTTTTTTTAATAAATCCTAATAGGGGTATGA

TATATAATTATATATGGTCAAAGAAGGTGGAAGAAGCGTGAGGATTACTGTGGAGGAAAAAAGAGACATT

TGGAGAAAGCAACGGTCAACATTACTGTCAGATGTCGAAGGAGAGAAAGTGAGAGAGTGTGAGACTCTGA

GAGAGAGAGAAGTCAAGAAGGAGAAGAAGACAAAAGCTAATTTAAAGCTACGAATAATTTAAAGCTACGA

AGACGAGACGGGACATATATTCACCCTCGCTTTTCACATATATTTTCGGTATTGCCACTCTCAAATTTTA

TTTTTTCCCTTTTTTCTTGTCTTTTTTGTACCCGGCCCTGCTTATTTGGCTATATAAGCAACTACCTTAT

CTAGATATCTTCACCTCGCAATCTTCCTCTCTACGTTCCAAAACCTCTCTCACTCTCTGTCTTCACCTTT

GTGGTAATACTTTAATCTCTGATCGAACCGCACCAAACCAGTCCGGTCTTTCTTCTCGGCCTCGTCTTTT

CTCCGGTATTCTTTCTCTTCTTAATTCACATAGATTTCATAACAAGTGATTTTTTCGTAATAATTAAAAT

CCGATCAAATTCACGATAGTGATATGATATATGCATATATGCATCCAACACGTTATATGCATCCCAGCAT

AACAGTTTTGCTTTTCTTTATTTTTTTTTCCCTTTAAAAGATTTGGAAAATTAGCCATTAATCCCATAAT

AATCTCTTTTTGCGATGTGTATTTTGTTTTTTTTCTTGTTTTAGTATTTCCGTTTCACAGATTCGCCATT

AATCCCATAATAATCTCGATTTGTTTTTTTTATTTTTTAGTATTTCCGTTTCACAGATTCGCCATTAATC

CCATAATATTCTCTTTTTATAATGCGATTTGTTTTTTTCTTTTTAGATTTCCGTTTCACAGATTCGTTAA

TCATAAAAAACTTTGATACAGAAATGGCGTTACAGAAGGAGGACAAGAACAAAGAAGAAAATAAAATGAC

AAAGAAGAAGTGGCAGAAGAGTTACTTCGACGTTTTTAGGAATCTGTTGTACATCGGAGATTCCTCTGAT

CGAGAATATTCTCAAGTCTCTCGACGGCATTAAGGACTATACCATCATCGTTCCGTCGAGAACCGTGATC

GTTGTCCACGACAGTCTCCTCATCTCCCCGTTCCAAATTGGTAAAGCATTAGCTAATCACTTTCTTCGAA

TTTTTATTTTTACCTAATAAAAATAATTGAATCAAAAACCATAAAGTAATCTCACTTAACACGTAAACAA

TCACTTTACTTTTCTTCTCTTTCTGTTTTCTTCAAAATTAATTAATGGTTTCGCGTCCTCGTTTGATACG

CAAAGCCTCAAATTAATTTTTTTTTGGGAACTAAAATTACTCTATCTATCAGATTTACCATAAAAGCTTA

CTTTGACTTTACAAAACATTTATTAGCAAAATTCGTTTATCACCAACCTATTCAAGATTTAAGGGAAAAT

AGTTATCCTCAAAACTAGGGAATTCAGATTTTTGAAGTTTTTAACGATTCTACTGAAAAACAAAAGCCCT

ATTATTTGGGTTTCTTCTCGAGAAAAAATAGAATATTGTTGTTATGGATTTTTTTTCATTTTTATTAAAA

TTAAAAGAAAATTCAAAAGTTATTTATAAATCAAGTTTTTTAAAGCTATTTTGATGGATTGTTTTAGGAA

AATTGATCTAACCAACAATTGTAATTTTTTTTTTTTGTGTGTGTGATAAAGTCTACTTTTTTCAACATTA

AAAAACTAGAAATTGAAATTTACGGCTTCTTTATACAATTTTGCTCGAGCCAGCATCTTTGTGTATAAAA

CTTTGCATAACTCATACATACCACATGTGACATGTCACGTGTGTACTGTGTAGCATAAACAATATCTAAC

TGAGTATTCCAAAAACATTTGCAAAAGAAAAGTGTTCAGAAAAGCCTGTTGAGTTATTTACCAGATCTTT

TTATAATTTTGCTAGAGCCAGCTTTTTTGTGTATAAAACTTTGCATAACTCACACATACCACATGTGACA

TGTCACGTGTGAACTGTGTAGCATAAACATAATATCTAACTGAGTATTCCAAAAACATTTGTAAAAGAAA

AGTGTTCAAAAAAGCCTGTCGAGTTGTTTACCAGATCTTTTTATACAAAATTATTTTATTGGTAGTGGAT

CATACTCGTTACTTAACTATATATTTTATTTTTTATTTGACTACGAAAACCCATTCCAGTAGTTCTTTTT

TTCCACTCAAAGAAAAGTATAGTAATTTGATAGTTAAAAAAAAAAAGTATTAATTTTTAAAACAAAATTT

CTTACATATTGGTTGTTTAATCATTAACTTCCAAACAAAATTGCGGTGCAGCTAAGGCACTGAACCAAGC

GAGGTTAGAAGCAAACGTGAAAGTAGACGGAGAAACCAGCTTCAAGAATAAATTGCCAAGCCCTTTCGCG

GTGTTTTCCGGCATATTCCTCCTCCTCTCCTTCTTAAAATTTGTATACCCACCTCTTCGATGGCTAGCTG

TCGTGGGCGTCGCTACTGGTATTTATCCGATTCTTGCAAAATCCGTCGCTTCTATAAGAAGGCTTAGGGT

CGACATCAACATCCTAGTCATTATCACAGGTAATACCCACTTTTCACTTTTTATTTAATATTATTATTTT

TATCCACATCACTCATATTGCGTGTAACTACTGTATAATGATTTGTTAGTTTACTATGTAGTATTAGTTG

AGAAAGAAAAATTGTGGTTATAGTAAAACTATTCAGGCCCTATTAATAGACCTATAATGTTCTTGGAAAC

TTGCGAGTCTTTTACGCTGAATTTACCCCTTTATATGGTACTTCAGATTAGCTTACCTATATACTACTGC

TTTCCTGCAACACCTACCACTCCACGAAACCTTTTAGAAAGTTATCCTTTACTTTTTTCTTAATATTTTT

TTAAAGTATTACATATGGGAAAAATATCAAAACACATATTTATTAATTAATAGATGCGCAATTATTACTT

TATAGAAATTCAATTCTAGGAATGTAGCAATTTGATATTTCTGTTGTATATGTTAATTGTATATTTGAGT

TATAAGTTGTGGAACTACATAAAACTACTTTATATTTTCTTTTTATGTAAAGTACATTTGAGTAATAGCC

TAATAGGATATAGAAAAATATCAAAATGTCAATGTTTTTAAAACCGGACCAGAAGGCGAACCGGATAATC

ATCCGGGTCATGGTTCAATTTGGTTCGACCGGGTTGAATTCGGTTCATAATAATTTATGTTTATTTATTT

TTAAATATAGAACTTTTATTTTTCAAAGTTCCCAAGTGTAAACACATACATAGAATAATTATTGTGATTT

TACATAATTCTCTTATGGAAATATAATAATTCTTTTTTAACATGTAGTTTAAAAAGATAAATCTTTTACG

TACACACAACATAGATATATAGATTTTATATATAACTATCGAGGCAACTAGGAAAATGGAAGTTTCATGA

TCGAGAGTTGTGTGGTTCTTTGGGAAAACTTAATTTTTTTGGTTATTTTATACGAAAGTAAAGGATTCGT

TTGATTCTTGCTCAGTTTATTATTATTATTTTTTTTAAAAAAAGTACTGCAGTTACGTCCCATAGAAGAA

AAAAAGGTTAACTCGTATTTGATTGGCTTATCTTCTACGACTCAAAATGGGAAAAACTACAAAAAAGCAA

AGCAAACTTTTTAGTTTTAAGTTTTAACTCGTGAAAAGAAAATTAAAAAGAGCAACAAATAATTGAAAGA

ACAAAAGCATCAAAAGTAAAGAAATTAATTCATAATTCATAGACTGATAACGGAGTTACTTTTAGTTGAA

ATTTCGGTTTAGGACACCAAGCTTGTGAATCCATAATATAAAATATTTTTTTAAAATCTTGATCTTTTGT

TCGTTTGTATGATGTAATAGTCACTTCAACAAAACTATAACTCACTAATATTCCAATTTCATCAAACAGT

GGCTGCAACACTTGCAATGCAAGATTACATGGAGGCTGCAGCAGTTGTCTTCTTATTCACCATAGCTGAC

TGGCTGGAAACAAGAGCTAGCTACAAGGTATGTTAACTAGTAATCATCATATATTGTGTTAATCAAACTA

CTATGGATTATCTGAAGTTGAAATTGTAATGGATTATTGATTATGGCAATTGCAATCCCAGGCGAGCTCG

GTGATGCAGTCTCTGATGAGCTTAGCTCCACAAAAGGCAGTCATAGCAGAGACTGGAGAAGAAGTTGAAG

TAGATGAGGTTGAGCTCAACACAATCATAGCAGTTAAAGCCGGTGAAACCATACCTATTGATGGAATTGT

AGTCGATGGAAACTGTGAAGTAGACGAGAAAACCTTAACTGGTGAAGCATTTCCTGTGCCTAAACAGAGA

GATTCTACGGTTTGGGCTGGAACTATTAATCTAAATGGTAATGTAACCCTCTTACACAAGCTTCAATCTT

AGAAAAGTTTCAAGCTTTAACCTTTTTGTTTTGGCAGGTTATATAAGTGTGAACACAACTGCTTTAGCTA

GTGATTGTGTGGTTGCAAAGATGGCTAAGCTCGTAGAAGAAGCTCAGAGCAGTAAAACCAAATCTCAGAG

ACTAATAGACAAATATTCTCAGTACTATACTCCAGGTTTGCAAAAAAACATAAACCATAACTTGTTTTCT

TTATGTTCTTGATTCTTGTAATTTGAGACCTCTCTGTTTTTTGTTTGTTTCAGCAATCATCATAATATCG

GCTGGCTTTGCAATTGTCCCGGCTATAATGAAAGTTCGCAACCTCAACCATTGGTTTCATTTAGCACTGG

TTGTGTTAGTCAGTGCTTGTCCCTGTGGTCTTATCCTCTCTACACCAGTAGCTACATTCTGTGCACTTAC

TAAAGCGGCAACTTCAGGGCTTCTGATCAAAAGTGCTGATTATCTTGACACTCTTTCAAAGATCAAGATC

GCTGCTTTTGACAAAACCGGAACTATCACTAGAGGAGAGTTCATTGTCATAGAATTCAAGTCACTCTCTA

GAGACATAAGCCTAAGCAGCTTGCTTTACTGGTAATAAAAACAATATCTTGTTCTAACCAAAAACTAGTT

TGATGGGATAACGTATGAATGACAATTTCTTGTTTGGTTCTCAGGGTATCAAGTGTTGAAAGCAAATCAA

GTCATCCAATGGCAGCAACGATTGTGGACTATGCTAAATCTGTTTCTGTTGAGCCTAGGAGTGAAGAGGT

TGAGGATTATCAGAACTTTCCTGGTGAAGGAATCTATGGGAAGATTGATGGGAACAATGTTTACATTGGG

AACAAAAGGATTGCTTCACGAGCTGGTTGTTCAACAGGTAAAGCTTCAAACTTTGGCCAAGAAAAAACTC

AATGGAATGGTTTTGTTGAGCCTTTGATCATTTTGAAACTGTTCTTTCTTGACAGTTCCAGAGATTGATG

TTGATACCAAAAAAGGAAAGACTGTCGGATACGTCTATGTAGGTGAAAGATTAGCTGGAGTTTTCAATCT

TTCCGATGCTTGTAGATCCGGAGTAGCTCAAGCAATGAAGGAACTCAAAGATCTTGGAATCAAAACCGCA

ATGCTAACAGGAGATAATAAAGATTCAGCAATGCATGCTCAAGAACAGGTATGAGACTGAAAAAACCAAG

AATTTTTCATTACTCTCCTAACGTTAAGAGATTATATTAAAACTTTGACATGTTCTTATATGGAACAGCT

AGGGAATGCTTTGGATGTTGTTCATGGAGAGCTTCTTCCTGAAGACAAATCCAAAATCATACAAGAGTTT

AAGAAAGAAGGACCAACTTGTATGGTAGGAGATGGTGTGAATGATGCACCAGCTTTAGCTAATGCTGATA

TTGGTATCTCCATGGGGATTTCTGGCTCTGCGCTCGCGACGCAGTCTGGTCATATCATTCTCATGTCAAA

TGATATCAGAAGGATACCAAAAGCGATAAAGCTAGCAAGAAGAGCTCAGCGGAAAGTTCTTGAAAACGTG

TTCATCTCCATCACTTTGAAAGTAGGGATACTGGTTTTAGCATTTGCTGGTCATCCTTTGATTTGGGCTG

CGGTGCTTACTGATGTAGGGACTTGCCTGATTGTGATTTTTAACAGTATGTTGCTTCTGCGAGAGAAGGA

TAAATCTAAGAACAAGAATTGTTACAGGGCTTCTACATCTGTGTTGAATGGTAAGAAACTTGAAGGCGGC

GATGACCAAGGCCTTGACTTAGAAGCAGGGTTGTTCTCAAAGAGTCAATGCAACTCAGGATGTTGTGGTG

ATAAGAAAAGCCAAGAGAAGGTGATGTTGATGAGACCAGCTAGTAAAACCAGTACTGACCATCTTCACTC

TGGTTGTTGTGGTGAAAAGAATCAAGAGAGTGTAAAGCTTGTGAAAGATAGCTGTTGCGGTGAGAAAAGT

AAGAAACCAGAGGGAGATATGGCTTCACTGAGCTCATGCAAGAACTCTAACAATGACCTGAAAATGAAAG

GTGGTTCAAGTTGTTGTGCTAGTAAAAATGAGAAGCTGAAGGAAGTAGTAGTAGCAAAGAGCTGCTGTGG

AGAGAAGGAGAAAGCAGAGGGAAATGTTGAGATGCAGATTCTAAATTTGGAGAAAGGGTCGCAGAAAAAG

GTTGGTGAAACCTGCAAATCAAGCTGTTGTGGAGATAAAGAGAAGGCTAAGGAAACACGTTTGTTGCTTG

CTAGTGAGGATCCATCTTATCTGGAGAAGGAGAATCTGAAAAGTGAAAGTGGTGATGATTGCAAATCTCT

TTGTTGTGGAACTGGTTTGAAGCAAGAAGGGTCTTCTAGTTTGGTCAATGTTGTGGTGGAGAGTGGTGAA

TCCGGGTCAAGCTGTTGCAGCAAGGAGGGAGAGATAGTGAAAGTCTCTAGCCAAAGCTGTTGCACAAGTC

CAAGTGATGTGGTGTTATCTGACTTTCAAGCTAAGAAACTAGAGATTTGTTGCGAAGTGAAGAAGACTCC

AGAGGAGGTTTGTGGATCTAAATGTAAGGAAACAGAGAAGCCTCACCACGTTGGTAAAAGCTGTTGCAGG

AGTTATGCAAAAGAGTATTGCAGCCACAGGCATCACGACAACCATCATCACCACCATGTTGGGGCTGCTT

GACGGAGATAGTGATTGATTACCTTTAAACTCTCGACCCATCCATCTATTTGCATAACCTTTCCTTCTTC

AACCAATGTCGCCCAGAACAAAATAAAAACTTATTTAGTGTTTCCAGCAAAGGTGTGATTCGTAAAGACA

ATGCTGTTGATCGTTGTTTGTCTTTTATGTTTGCCAAAACCATAATGTATTTCTCCTTTTCTTGTTTTTA

TTCTCTTCTTGAAGATGCCCAGAAGAAGTTTGAACTTCGATCCTAGAGTCTTAAAATCAAATAGAACAAG

CAGTTGAAACATAACTTAGCCTTGGAGTCTTTTTGTATGCTGTGTACTACATAAGCTTTCTTGACTGACA

CGTTTCTTGTCAATTCTAGGGCATTACTTTATTAGGACAGAGAAGGTGTTGCAGTTCGTGTCCTGGAGAG

TTTAGGTGAAAAAAAAAATAAAGAGCAAAAACTGACTGCTCGCACATCCATGTAATCAAGAATCAGTAAA

AATAAAAATTAATCAAAGGGTGACACAGCTCATGATCTTATATGAATCACCAACCATACTCTTCTCACTA

TATAAACAAATGTGTCATTTCTTGAAAACAATCTGAAATATTCCGCAATCGCTGAAAGCATAGCATTAGA

GGCAAAACCCTAGCTATTCTTTTGTTCTCCGTCTTTATTTCTTATCTTTTATTTCGTCAAGCTTGTCGAT

GAGGTGAAAACCTTCTTAAAATATATTATTCGCTTCTTCAAGTTTAATAAGACATATACCCTAAGTTCCA

CAAACTTTTGTATCTCGAGATAAAACTTGTGACACATCTGATTCAACACGAAACCTAATGTTTTTAAGAT

CTACTAGGTAGGATACTTGCGCTTCGCCGCGGAAGACTTTTTTTGTATTTTGATATTTTATATTTTGATA

TTTTAATCCAGTTTTCCTTATATTCCATCTGTTCCATATTAAAATGTCGTTTAAGATTTTTTCATACATA

TTAAGAAAATATTAAAATTTTTTATTTTACTCATTATTACTCAAAATTAATCTTCTCAAGGAGAAATGTG

TAAAATCTTTGATGAATTCTCTTAGAAGGAAAGGAAGAATCATGAGTTTGATCTCCATCAATTTCAGAGA

AAACTGCTTCCTCTTCCTCCTCCAGGTTGTTCTTCTTCTTCCTCCATTCTCTTATGCTCCGACGCGGAAT

CGGCTTAAGACTTTTAAAGAAACACAGTCTCTAAACGCTCGATTTACGTTTTCCGGTTAAATGAAGGAAA

TCAATAAATGAAAACCGGATAAAATTAAACCAAAAAAATTGCTGACTGATATTATTTATAATATTTATAT

TAGATGTTTGTTTTAAATAACGTGAAGTAGATTATCTTGAGAAGCACATGAGATTTTTCTGTGCTGATTA

ACTACAATATCTTTACAAAAAAAAAACAACAGCAATTCCTCGAATGACACACGTAAAAATTAACTCCAAA

TAATGTAACTTCATTTTTACCCCAGAATTAATGGTTGATTCCACCAATTTCATTATAATAGCATTACCAT

TTAGGTCTCTGGTGATGTTTATGTTTGTATCACCATTCATTAGTTATATAAAATATATAATGCTAATGTT

GGGTGTTGATAGTATGTCCACTATATAACTTCTTCGTGTTTCTCACTGTGTACATATCATGATAAGAAAC

TTGTCTCTAGACCTTGTCCCCATTTTAATATTATACACTTATTCAAAATCTTTATATATATATATATATA

TGGTTTATTTATATACTATTTCAAAAAAACAATTGTTACAGTTAAATGTTTTAATTTTTAACATGTTGGT

TCCAGGAATTCAAAGTGACTAATTTGATGATCTATATATGTTGCTTGTAATTTGCGGTTTCAAGTTTTCA

ACAATGTCTGAAGTGAGACAGAAGTGAGACAAAAGACTTTGAAAGATTTGATTGGAATATAAAATCTCGC

TATGCTTAAGTATCAACTAGACAACTTATAACAGTTGAGACAATAATAGTAAACTATATGTTGGCATAGA

AAATGGCGTTTTCTATATGAATTGGCTGGTTGATTTATATATTTACAATCTCTAGTTCAGTATTCAGATG

TTGTCAAAAAATCGAAAATAAGGTTAAATTGTGGTTTCACGGTATAAAAGATTAAATACCAAAGTGGGTA

TACATCCAAACTCCGGAATATATAACTTACGAAAATTTCAATATGAATTACCAAAAACAGTTCTAAGAAA

TCTGAATACACGTCTTAACTCTTAAGTAGTATGTTAGAATTTTAGATGTACTTAGTCGACATTTTTTCAG

TTTTCGATGTGAATTTAGGACTGGTTATCACTCATTAGGACAAAAGTGGATCTCACAGGTTACTACTTTG

CATCAATTCTATCATATAACTCAATGGTCATAATAGGCTTGGGCATTTTTACCCAGCTCGAAATACTAAA

CCGAATCTGACCCATAATAGATGGATCGAACCGAACCGGAACACGAATATTCGAATGGGTCCTAAATTCC

TATACCGGAAAGAATAGGACTCGAACCAGAACTGAATGAGAACCAAAAGAGTACCCAAAATATTCAAAAT

ATAATTATATACCAAAAAATATTAGTTATATTTAGACTTAAAATAACTAAAATATGTAAAATTACAATTC

TAAACTTAATATACTACTTAAATTTAGAAAAAATAACCAAAATATTCAACAAATCCAAAACCGACCTGAA

CCCGGACAGAACCGAATCGGATCTGACCCGAAAATAAAAAATATTTGAATGGTTTTAAAATTTCTAGAAC

GAAAAAAACTAAACAAAAAACAACCCAAACCCGATCCGAAAAACAGAATGCCCGGGCCTTGGTCAAAAAC

AATCGTAGAACGATATGCTTTTCTTTGATAAAGATACGATTATGATAATATTTAACGAATTAAGTATACT

ATTAAGCTTGACGCCTTGACCACCTCACTGATAATTTTGTTGCATTGCGCATTTGCACTTTCATAACCAT

TTTTACAACTTTTCTCAAAATATTAGATATCGATAAATAAATTAAAGGTATACATTAGTAATTTTCTAAG

TCGTATTTATGTTTATTTGAATGCATCGCGATAACATATCCAACAATATTTTTCTCGAGATTCGTGTATT

AGTTTATACAATATTTTTTTAAAAAAATTAGACCGATCAAGAGGCCTCAGCCACACCTAGCTAGGCTTCT

AGGAATCCATTGGTTATAGTCATATAGAATTAACAATTTCTCAATTGTAAAATTATAATCTTGTTTATTC

AGATACGTAAAGGTTAATGAGTCATGTGACATTAATAATTGTCTACTCACATTATTTAGAAGATTCAACG

ACTCCAAACTATTCTTGATAGTACAGTTGTTAAATAATTGGAGTACATGTTGGGTCTTTGGTACGACTCT

TGCTTGCATTGAAATCGATCATAACCATAGATTAACGATTCATAAATGCGAGGGTGACATTTTTCCTTTA

AGCCGCCAAACATTCACTTTTTATTACAATAAATTAAACTGAACGGTTTAGGTTGTTAAGGTGAAAGTGT

TATAATTTCAGTTCAGTTTTACAATAATGCAATTACCGAAATACTTGATTTTGAGATATTGACATGGATT

TATACTATTAATTTAAAGAAAGACAAAATTTAGATGAGGCACTTCACCCCTGCTTAGGCTTCACTACGAC

CACTAAAATCTTATAACGAAGTTTTAGAGATTTACTCCTTGCTTTATAAATATGGATTTCGAATCACATT

TAGACAATCACTTAGATTGGTAATTTTTAACTGAACGGTTTGGTATCAATACATTTTCAAAATTATTAAT

CAGAAATACTAGTGTTAAACGAAAATATTCATATTGATGGTTGATTGAGGAGGAGAGGTGGGCTCCAGCA

TAAAATAGCTGTGCATTTTAGGATGGGAATCCCGTTTTTTTCTGTACCAACTCAAATTTTCCAAACTTTT

TGAAAATGAGAAGTAGCAATTTTCTGTTGGATTTATTTATTTTATTTTAATTGGTAAGGAAAAAGTACAT

GATAATGAGAAAGAAAGAAATACCTGAATTATTCGTTTAAAAATTAAATAAAACAAAATTATTCATATTT

TCCTTAAATTTTTCGAATATTTTATGTATTTACCCAAAATTTATCGTATTTTTCCCATAATTTTTTCAAA

ATCTCATCAATTTTTCCGATTAATTAAGACTTTGGTCTTTGGTATGTCTAATTTTTAGTTCACAAACTTC

TTATTTCCTTCTTTTCCCGTTATTTTCCGTTCCCATTATCATAAAAGAATACTTCGGATAATATATTTTG

ATTCCCGAATTTTTCATTATCGTTCAGTTCCCGTCAATACCGGTCACAAAACCCAAAATGCAGATGGTTA

TGAACAAGTGTACTTTCTAATTCTACCTATCCACTTGCGCCAAATCTAGTGAATATGTCAAAATTGGCAA

AATGAATATAATAAACTATATGCCAAAAAAGGTTCATAATTTTTTTTTATCAGCATATACTGGTTCTCTT

TGAATCGCATATCAATAAGAAGCATCTCGTTTATCTAATTACCAACGCTAATGAGAGACGACCATTCCCT

CATGTTCTCTCATTCACCCGCAAAATAGTATTGAGTTCTCCGCCTATGATAATCAGTTTATTCACCAGCC

GCAACTCATCATTTAGTTGATGCGTACGCGACGATGATATGTAACACCTCCGCATCCTTTTTAATTCTCG

CATGAATAAACTGGTCCGTCGAATTAAGGACCTCTACATCCCCTATCCCATGTCTTCAAAATAACCACAA

TCCACCGCTCTGACTCACTGCATCCACTCTGAAAGAACTTTCAAACCCCAAGCCTTGACAAATGCTTCCT

GCTCTCTCTGCACCAAAATGTGTTTCAAAAATAGCCAAAACATCCGTAAGCCACTTCTTCACCATATACC

GAATCAATCTTCTGAAATAGGGTTTATTCGCCTCCCGAAGTTCCATAAAATACAATTCATCATAATTTCA

TAGCTTAACCATAGAAATATCGGGGCAACTTGTTATGCTCCAATCTCCTCCATCTGCTTCTCCGACGATG

CGATGTGCGTAACCAACGTCCCAATCGAACTACGTATCCAATAAAACCTCGATGTCTGTTTCACCTAGAT

TTTGAGCCAAGATTCCATTTTCCCGTACTTCACTTCCCTCCGTCACCACAAAACCCCCAGCCCGACCAAG

GTCCCCCTGTTCAACTCTGAGGCGTTTACCATTTGCAGATAGAGCAGTCTTCCCCTTGGTTGGGCCAAAA

ACAAATCCGCTAGTGGGCCTACTAGTTAAATTTTGTTTGGGCCGAGTAAGTTCCAAACTCTTCCCATTCC

TGAACTTTTTTCCTTTATGCGCAGCTCTAGTGTAATTTGGGCCTTGTCCCGATCCAGACCCAAAAACAAT

CCCCTTCCCGTGTTCCTTACTCTTTCGGTGCTTAGTTCCTAAGACCGGATAGACATTTTCCTTATCTCCG

TTGCAAACATTAATGTTCATTGATTCCACTATCTTGATTGCGCCCGTACTGCCCTCTCGTCCATTAACTG

TCGAGTCCCTATTCTCTTCGGTTTCCATATTCTCATCGAGGTTTCCACACTTATTCCTCAACACAATATT

CAATGAAACTCTTTCCTCTCTTGAGACTCGCGTCTCCTCTTCCATCCCTTCTTGACCAACCACCTCCGTT

GGCCGTCCCCTACCTCGTCGAACCTGTGTGAACTCCTCCTCAACCGAAACAGTTTCCTTCCCATTATCCC

TATCCTGCTGGACTTGCATGTATGGACCAAGTGTCCATAAATTCCAAACTGAGAACAAATGTTGGTAAGA

CCTTCATAGGACAAGAAATATCTCCCTCCGTTAATCAACACCGTCCCTTCAATGGTTTCTTTAGATTAAC

CTCAACGGATACCCTTGCAAACTGTGCTATTTCAAAGTTTAAAGTTGTGACGTCAACCTTCACCGGTTTC

CCAAGACCCTTTGCAATCCCCTTTATGATAGCCTTATGATAAAAGTTCACCGGAATGTTCGATAACCTAA

CCCAAACCGGCGTTGTCTCAATCTTAGGATCAAACTGCGGAGACCACGCTTGCACCATAAGATAACTCCC

AAACGCTTTATATGGACCTCCCGTCAATGCCGCCATGTACTCCTCTTCATTCTCAAAATGGATCATAAAG

AATTGACGAGGTAGATCCATGACATACATCGCAATTCTAGGTTTCCACATCTCCCTTAACTTCTTATTCA

TCGCCGTGATTGACACAGTTCGACCTAACACTTTGACAATCATACATTTTTTCCACAAATCGTTCATGGC

CGCCAACACTTCCTCCCTAATCGTTATTACTGGTTCTCCATCCTCACCATTCGGAAATTCCAAACGAAGT

CTTGCTTCTACAAAGGCTCATCCACCACCGTCTCTGGTACCGGTCTCCCACCCATACTACTCCCCGTCAT

CCTCTGCGCCTATGAGCTTGTTAAATCTGGTGGTCGCCCCTTCTCCCCAACGTCATCCATGTGGGCATTG

ATGACCCTGCCACTCTCGACCCCTGTTTCCTCAATCGCCTCACTCACCGCACTCATTAGGGTTGTTTTTA

TAAGTTTACTAGTATGTGCCACTTAGCAATAGACATACTACTAGACTTATTTAAGTAGTAATGTCTCAAT

TCTAGGCAAGATTTATACTTTTGTTTAAAACAGATTATTTATGCTTAAACATGATTTCGTAGATTGGTAG

ACCTCGGTAGGGAATTTTGGCATTGATGAATTGATTGGGGATGACTCTAGTAGTCTCGGTAAACATCTAG

AGCTTTCCATTAGTTGCTAACATGTGGGTAATATGTACCAGTATCACATACTATATGATTTGTCACGTCT

CGGACCATGATTGTTTTCAACAGGGGTCCAAAATAGGTCAAGGGTCAAAGCGGGGAATCGAACTTGGGTC

AGAGGTTTCAATTAGCATATTTTACCAATTTTCCTAGTGAGTTTTGCTACAATTTCTGTTTTTATAAAAT

GAATAGGGTGTCACTTAACATCGTATTTTTCTAAGTCACACCACTGTCTCGGACGCGGTACGGTTTGAAT

TAAACAAGTAAGATTCATTTTTAAGATAAGTTACAAAGAAAATATCAAACATAATTAAAAATTTTGGGAA

GCCAACAGATTAAAGATAATTAATGCTATTCCAATGTTGAGTCGCAAATTTAAGTTCTAATTAAGGAGAG

AATTCACTTTTTATAGAACTGCCGCAAATTTTTTTTTATCTTTCTACCTGATTATTCAAGCACCGAGCAT

AAGTTATGATCTTGTGCAAACATGTTACTAATTTAATAATATGTATTAAAGATATAGATACATCTTAGAA

GAAAAGCTAAGAGACTAGACGACAAGTGCATTGCGTTAAAAAAATGTGTAGTTTAATTTGAATTTTAGAA

ATAAACTAAGAAAATTGTACTAAAAACCAAATAAAGAAAGCAATTAGATGAGGAATCACACATGGATTCC

ATTTTGTGAATCCATTTTGTGACATTACACTATTGGTGTTTTCCACTAACATTTTACTATTTTAGTAACT

TTGACTTCGTATCTCTCACTCACGAGATTAAATCCCTCTTTGATCAAATTTTCTGCTCAATTATTTCTTT

AGATAACTAGCAAGAATCTATATTATTAAAAGAGAAACAAAACCTGAAAGTTATAAGGATAACACTATAT

CCAAACAAGAAAAAAAAAAAAAAAAAAAACTGACATAAACCGGGTTGAATCCTGTTAAAAGACCAAACAA

CCCATTATCTATACTATTAAAAGAGAAACAAAATCTGAAATTTACAAGTTTATCACTATAATCAAACAAG

AAAAAACTCAGTCACAACCAAAAATACCCGACTAGATCCGACCCGGCAACAATGAGAAGACAAAAAGCCC

AAAGCAGTAACAAATAAATATTTTTCGGATACAATTTTAAGAGGGGTTTTCTGGTCCGAAAATCTTCAAG

AAATATAGTTGTTTGGCAACGGAGATACTCGATCCGACCCGAACGCGGCGTAGAAACACGCGGATCTAAC

AAGAGGAGAGGTGACTTAAATTGAACCGACTAAATAAACCGAATATTGTTCCAAAAACAAAAGAACCGGA

CGCTTACCAAAGCCCATAAATAAGTCCAATTAACCGGATAGCTCCTAAGAATATGCAAAAAATTATCAAG

AAAATTAGTTCTTTTTTCGATATTTGAAACATAAATGTCATATATTTGAAACTTAAATACGATATCACAG

CGTAAGAAAGATTTTGCGTACCCTGATTTAGCATAATAAATACTTCAGCTTCGACGAATGAAAAATCTGC

GTCATTTTCCATAAATATCGTTAATTAACTGTGCCAATCAAATACAAAAACAGAAATGTCATAAACATCA

ATAACAACTCGAATTTCTTTCCACGCAAAATCTAAGAAGTTTTCCACAAATTTCGCTAATTAACTAAAAC

GAATATACTTAATACCTAATCTAAAGCATGTACTAAGCACACAAAAGTGTCGACATTCATGATAGTATTA

AAACTGAATTTGAAAATCAGTTACCAAAAAGAAAAAATCAAAGATTCTTGTGAAAATATCCCTACCAAAA

ATATACCGTGTGATAATTAATATTTTTAAATCATATTGATTAAAAACCTAATGACCCATCCTATGAAACT

ATAAATACATCGCTCACAGGGTAATTGTTACATACCTTGAAATTGCTAATGATTTTTTCCTTCTGTCTAA

TTTCATGTTTACTAATCACGATTTGTTTTATATGTAGGGGAATTCATGAATGGAAAAGCAACCAAAAGCT

CTTTCTTAAATCACCAAGAAACTATGATACGATCAAAGGTTAATCCCCATCGATCGGGTCAAGAAAATAG

ATAGTTGCCCCTTATTGTTTCACATACCTTTATGATGGCAAGCCTTTCAAAATAGGTTGGGGAATCCAAG

TTAAGCTTCTTTTGGAAGCAATACACGTCCGCATCTGGACAAATGGAGCTAATCCTAGCAAAAGAAAATG

TAAGTCAACGTAATATAATAGACTATTAGAGATTTTGTACACGTTTTCGATTCTGAAATCTTAATCTCGA

TATGTCTTGCAAGGTATGAAAACACAGGCTTATGAATGTCACCATACCTTTAATTCGATTACGCGTGATT

TTCATCGAAGTTTACAGAACCAACTGATCCGACGGATTCACCAAATCGATTTCATATCAGAAGACTAGAT

ATCAATTTACGATCTTCGAGTCTACGCTCACGGTTGTGCGACTTTTCGTTTTTCGCAAGGATGGTAAACA

AAAAGAGTGGATCCGACAAAAGGCCCACTTTTTAATGGATAAAAACGACGAGAAAATGGGCTTATCCGTT

TTTAGACTCATACCCGAAAAGAATTTAGTTCGGCCCAAATTTTGACCACTTAATTTATCACTTTTAATCC

TACACTAAAATTAACACTAAAATTAACACTGAATAATTGTATAATTCATCTCATATCATATAATATATTT

TTTACCTAATGTATTTTAACGTATCAAAAATTACAAAATCTCGTGCTTCTCAACAACATACATTTCTTCG

TTATATATCAGAAATATATTATTCTTTACATAATTACAATATAAATACTTTAGGGGGGTTTATTGGTAGA

TGAATTTGTAAGAATTCTTAAAATTTTCAGAAATCTTTGTTATTGATTTGTGAATTCTAACAATCTTATT

AAAATCTGTTGTTATTGGTTTGGTGATTTATAAAGTCAATACAAAATCAGTTGTTATTCAAAAAGTTTGT

GTTTTAATGATTTCATGAATCCATTAAAATCCTTGTTATTGGGACATGGATTTTAAACATTTTAACTCAT

AGAACAAGATTTCCAAAATACTAGCTATAACCCTTAGATTTTCAAAATTCATTATAACAAAATATTTTGA

TTGATTTTATGAATATACAATCTCTCTCCAAATCTAACATAAACTCTTCATAAATTTAACAAATCTCTTA

ACTTTCAAAATTTATCAACTCTATAGAAATTCATCTCCCAATAACCCCCCTTAGTTTTCTTATAAAAAAT

TACAACATCAGGTCTAGTCATGATTATAATAATTCCAATTCTTAGTATGCAATATTGCGAGGATCATGTG

TCTAAACTAGCGACGTATCGGACAAGTTTTATCCTCGCCCCATATTCAAATTGATAATGTTTTATAATCT

CACTTTTCTTTTGTAACCATTTTATATAAAGTGTTAATAGATATATACCATATTTTTATCCCAAAAACTT

AAGAGTATGTAGTTGTTTTGATAAAACTCTAATTGATCATCTACTCCATAAAAAGCTAATTTCGAAATTT

ATAAAACAAAGTCACATGCACAAACAACTTATCTTGTGATTAAGGATGTTTTTACTTATGACTCAGCTAG

GTTCAAATCTCAAAAACATAGCCAATTCAAATTTTATGAAGTTCGGTACTACTGTAAATTGAGCCACCAA

TCGTTTTTAAAAAGGAGTTCGATTAGACTAGTCTATAATCCATTATAGTGAAAATTGCTACACAAAATAT

CATACTTTTATATAGTGCTAATGTAATCGATTTTAAAATAAACTTATAGTTTTATATTCTTGGAAATTAC

TGAAAACAAAAAGAAACTACAATTTTGATAGGAACTAGCCTGAAAATTCGGAAGGTAAAATAGGTAATTC

GAAACAAAGATTAAAATATACCTACGAAATTAACATGGTAAAAAAAACTAAACCAAATAAAAAAAGTTTA

ACGTAAAAAGAAAAGTTTTAATTACAAAAGAATCCAGACTAACCACAACTTTTGTAAGCATAATCCATTA

ATGTTAGGTTTAGAACGACTTTTAAAGATTTAGTAACTATTTTACAAATCATTTAGTTACGTACTTTCTT

TAAACTTCTAAGAACCAAATTTGTGGTATATATTTGATAATATTTAGAGTTATTTTTTCTTTTAGACTTT

TATGTAATTGAATTCTTTTGGGGATTTCGGAAAAGCAAAATATACATTTCGGTACTGTTAGAAGGAGAAA

ATTACATCAATGAGGAGGACTTTGTAACTATGGTACATTTTGCCTTAATAAAAATATATTGTTGCCCTTT

TATTTGTTTCACCTTTAGACAATTTAACTTTATATTTGGGAAAATCGCATTTTAAGCCGATAAATGCTAA

CATTTTAAACTTTTAAAGTTTTTATTAGCACTTCAAACACTCAAACTATTTTTTTCATACTTTAAACGAA

AGTAACAAATTTTTACGCGCCGCCGAAAAAATTAATGAAAAAAATTTAATATTCTAAATTCAAAGAATCA

AAAATTAAACGAAAAAGGCTTAATAACTTGAACTCAATCATACAAATTTTATTTACCCTATTTCGACTTA

TAAACATTCAATTATCTCTTTGAAACTCTGCATTGTATAAAAAAATTGTTAATTTTATGGTTTAAAATAC

GAGGATAAATAGTTTGAGTGTTGTCAGTGGTCATTTTCATTGTTTTAAAATGCAATTTCTCCCTTTATAT

TTTACTATTTTTTCAATTTTTACTTTTTATCTCTCAACAAAGAGATATCTTCTCTCCCTCACGATAAATC

CTTCTCTCTCTTCTTTTCTTTCTCCACTTTATTCATCTCCACTTTCCTTCTCTCTTTGCTTCCTACAAAT

TCTGTCTCTCATTTTCCACTAGGTTCTTCTTCCACCAATAAAGATTGGGACAAGGGTTGCGTAAAGACAT

TATTATGCCTTGAATAAGTGTTTGTGTATGCATGCCCTTTTTACAAAAAAAAAAAAAGAATAAGTGTTTG

TGAACAAAATATCTTGGATAGAATTTTAGAATATATCACGTAGATCGTGGACAATAGGTGTGTGAACATA

TATATATATATTCAGACATATAACAATAAAATATGTACACAAGCTTCGATTTTCACCTCTGATACAACAA

TTCATCCACATCTTTATTATGTCCAAACAAAAGTAATCCACGGCAATAAATCTACATAATTTGGCTCTCT

TTGATTCTTCCACTCTTACTTTCATCTTTTTATTTCCGTAATCACAAGCAACAATGTTTCATTTTCACTT

CTCGTTATCATCATATATTACAATTTTTTACCCATGTTAACCCTATCAAAACACCAATTAGATACATGAA

AACAATTCAATCCCACACCAAACCAGAAATCGTTTTAATTTATAGCTTCAGTTTCAGAAATTAATATACG

CCAAAAAACGTTGGTTACTGTGAAACAC
