# Supplementary material for: Tandem Quadruplication of HMA4 in the Zinc (Zn) and Cadmium (Cd) Hyperaccumulator Noccaea caerulescens
Source: PLoS One. 2011 Mar 10;6(3):e17814. doi: 10.1371/journal.pone.0017814 (PMC3053397; doi:10.1371/journal.pone.0017814)
Supplement: Data S6 — Consensus sequence of the entire NcHMA4 single genomic locus. (DOC) [file pone.0017814.s014.doc]

**Data S6 Consensus sequence of the entire *NcHMA*4 single genomic locus.**

>Fosmid N18P80 + P6P46 + H2P47 + B3B40 + J12P81 101480bp

CACGTTTTTGGTTTTAATGATTTTAATAAGAAAGAAAAAATGAGATCCAAATTTTGTATTGTTATTTCCT

ATCCACTGTTAAAAAGAAGGAGCAAAAAGAAGGAAATTACTTGATTTAATTTGTCTTCTTCAAACATTAA

TTGTGATGACATAAAGACGTGGCAGTGATTAAAGACTATGCGGTGACGATGACGGTTGGGTTTTGATATT

TTTTAAAACATGTCCAGTTTCATTTGTGTTGCTATATATGTATGAACTTGCCGGAGATTGTGTAAGATCG

ATGAGGTCAAGAAAAATCTGAAATATAGCTTACAAAATTATCTTTACTTATGAAAGTATTATTTTCATTT

AGTTAGAGAACTTAGGTTGATTATATATATCCTTATATAAGTTCAATAATAGTTCTTTCTAAACCCATAT

TCATACTTTTTTGGAATTGCAAATCTCATACGCTTGCAAACGAATTTGAACTCGATGCCGTGAAATAGAG

TCTACAAAATGACGAAAACAAAGAAACACAAAAACATAATTAGCGAGTTTAGATACTGTTAAAGCTAACC

TAATCTCGGCTACATTCATGCTCATCAATCCAAATATCCAATATGGGTTTTGATAAAGATAATTTCTCTG

ATCGTTTTTACACTTGTAAAGTTGTAAATGAATAAACTTTCTCTATTACCAAAACTATATATAGCAAGTA

ACAAATTCAACTTCTAACTTTCATTCTTCAGCTGAGATGACTATTTTGCCTTCACGTCTTAACCATTATC

TAGCGTTTGTGGTTGTTGCTGAGTCGCATCAGCTTCAACAGTTCATATAGAAAAGACAATGTAACCAAAT

CCCTAGTTTTATGGGATTCACGACGTATGTAAACTTTAAACAAATAAACAAACTACAGCTGATTGTAGAA

GTTATCAAAATAATTAACTAACATGCCCAAATGGGCCAATGCGTCGACTTTACACTCTAAATCATTTTGT

CTGTTAATAAGATGTGATCTACTTAATTTATACTATTAAATCAAATGAAACACCACGAAGAGAAATATAG

ATATCAGACTCAAAACTTGGAACAGTTATTAAAATATTCTTGATAAACACCATGAGGGTGCTCTATTCCT

GTCAAGATGACTTGTATTTTTCGTAATTATCTTTTCTGTTGAATTGGCTTGAAATAGAATTTTATTTCTG

TCTTGTCTTTCTCTATGTATGCTTATATATAGACTTTTTCTTCTTTTCTTCAACGTCGCCATTTGCAGAT

TCTCTTAATTGATCTCCAAAATATGGCCGAGGGAATGTTTAAGGCATTGTTGTGGGGTTTTGCGGCCACG

GTGTTTGCAATGGCCGAGGCAGCTCGTGGACAGCGAGTGCCTTGCTATTTCGTTTTCGGAGACTCAGTCT

TCGACAACGGTAACAACAATGCCTTGAACACCTCGGCCAAAGTTAACTATTCACCTTACGGTATTGATTT

TGCTAGAGGTCCTACCGGACGGTTCAGCAATGGTCGTAATATTCCCGACTTTATCGGTTAGTCCCAAGCT

TGGTTTCTCTGCTTGTCAATATATTTCCGATTGAATTGCTACTATAAACTACCAAAAAGCAACAATATTG

TTTTAAAACAAATAGTAGCATAAGATTTGATTTTCCTAACTTGAAGTTTAGAAATCACGTTTCTTTTATT

TTTGAATAAGACAATTAGAATTAATACTCTCTAATTGTTATATTCAAAATATTCTTTAGAATATTGGAGT

ACTAAGAAAAAATTAACGGAAACATTAGAAGTAATGTTGCAGAATAGTCGTCAAATGCAAATTAAGAGAC

TATTTTAGGACATACATAATTTTGTTTTTTTTAGTAACTTTTTATACGTATTATTAATCTATTCAAGACT

CTATTAAGGAAATAGAAAAACAAAACTTGTGAATGAAATATCGTGAATATCCTCATAACTAAATATACTA

GAAAATAACCTGCGCGTTGCGTGGGACTAAACATATATCAAGTTTTTGTTCATTGTTTTCAGATTTTATT

ATAAAAGTATTTTTAACACTAAATAATATAATATCCTAATTTGTTTAGATATTTCAAAAATTGGTGGATA

TACTATTCACGAAAAACATTTGGCACATGCTCGATATGTTAAATTCACATTTCATCTTCCTGTTTTTTTT

ACCAAAAATTTAGCATGTTTAACCTTTTTTGCTTTTCACTTTCTTTAGACTATAATAATTTATCTCGACC

GTAAAACTTTTAAATATTAAGATCACATTCTCACTTGGAATACGGTACAAAATAATAAATAACTCTTTAG

ATATCTAGTTTTTTTTTGTCGTAGACCACAATAACCGTTGATTATTATATTTTTTTTGGTATTTTAAAAA

CATACATATCATAATATATTAAAATTATCCAACTTTTCGTTCCAGTAAAATTTACATATAATAGCTCGAT

TTCTATCTATTATAAAAGTAAATAAATTGTAAACCTAATTTCTTATTAGTTTTCCACTATAATAAATTAT

ATTCTACATATTTAACATGCAACCACATTATAACATATTTAAAAATGATAAATCATTTATATAACTTTTT

GGTAAAATCTAACTATATATTATTTTGAGTGAAGTATTTCATTTCTTTCTCGCCAACTTATAATACAACT

TTGTTTGATTTTTAGTATCAAGTTATCATATATTTTAAAATATGTAATTACTTAATTTATATGTGTGTTT

TGTTTTAGTTTTTCATGTTTTAAGTATGAGTATATATGAAGAAATTATTAAATATAATTTAATAATTTTT

TATTGATGTGGACACTCTAGATGGAGAGAAAACTCCCTAATTATATATAAGATATGTATATGTTTTCTTG

TGGTACATGAGTTTGAATGGTTTATTTTTCAGCTGAATTATTAAGATTCAGTGATTACATTCCACCGTTC

ACCGGAGCATCGCCCGACAAAGCTCACACCGGAATAAACTACGCTTCTGGTGGCGGCGGAATTCGCAAAG

ACACTAGCCAACATTTGGTAAGAACGGGACAAAAGTAAGAGAAAGAGAGATTAACTTAAATCTATCTTCA

TTCTTTTTACTCGGGTAATATATTGCACTAGTTTTATAGGGTTGACTAAATTTTCTAAACGTACTATACT

GTTTTAGTAATGGTTTAATTGGTTCTTAAAACGTGCTGTGATCATTGTTTCTCATTTATTAGCTATAATG

TTTTGTTAATATAAAACTAAATACTATAATACAAATCTATGAAACATAAGGAAGAAAACATTTATATATC

ATACAAATCTATTAAGTGAATGAATATAACATTAATTGACGAGTTTTTTCTCCAGGAAAGATAACTAATC

ATGTGATTGTCTGATTGATTATCTCAGAGAGGCCTTAATTCATTGGATTTCAATTCAAAATTCTTAGGCT

CTAAATAGACAGCATGCATGAATCACAAGGAATACAAATTAAAGTATTATATAAAAGGTTTAGGTGACTA

AAATGAAACTTAACTAGTTTTCTTTCAAGGGAACTAATCATTTGAGTTTAATTGATTTACTTCTTCTTCT

TTGACAAGGAACAAAAACGACTCGTTTATTGAGTACTGCTTCCATATATATAAAAGACTAAAACTAACTA

CTTATGATATTTCTGATGATAGGGTGATAGAATCAATTTTCAACAACAAATATACAATCACTGGACGATG

ATTACGACCGCGAATGTGCCACCGGAGAAGCTGAACAAATGTCTATACACAATCAATATTGGAAGCAATG

ATTATCTCAACAACTATTTCATGCCAGCTCCCTACATTACCAATGGCAAGTACACTTACGATCAATATGC

TGCTTCCCTCATTAGTGGTTATCGCACTTATTTGAAGGTAAAACAAGGTTCTTAATAGATGGATTATAGA

TATCTCAGTATGATTATTTATTACGGCAACATTTTCAAAATATTAATTTATAATTTATAAGACATGTACA

TATGAAAAATAAACCGAAGAAAACTATAGAAAAATGATGCAGTGGATTGGATCTATCTCACTAAAATTGT

TTCGCAATCATGTATCTTTAAACATATAGTAATTTGAAGATACATATACATATATTTGTAGTCATTGTAC

GCCCTAGGAGCAAGGAAGGTGGCAGTGTTTGGGGTCAGTAAGCTCGGGTGCACGCCGCGGATGATCGCTT

CCCACGGTGGTGGAAAAGGCTGTGCCGCTGAAGTGAACAAAGCGGTTGAACCTTTCAACAAGAAGCTCAA

AGCTCTTGTCTGTGACTTCAACAGAAACTCCGATGCTAAGTTCACCTTTGTAGATCTCTTCTCTGGTCAA

AGTCCTCTTGCGTATGCTGCCTTAGGTACGTAAACTATATATGTTGTACTTTTAGAAGGAAAAAAAAAAG

AGAAAAAAGGAGTTTTGCAAAGATTCATACTTTTAGAAAAACTTCATAAGGTTTTACAAAGATTCGTACT

TTTTAGAAAAACTTCCAAAGGTTTTTTTCACAGATATATATATAGGTTAAGAAATATAAAACAATATGTT

TTGTGAATTGTTATCATTGGTTATTTACAAAAAATAATATTCATTAGAAACAAAACATGTGAACACACAC

ACTTTCATATATATAGTAGATTTGAATAAACCTGTTTACTTTTATATAGGTTAAGGTTTTCGTTTTGCTT

AAATTTCAAATCTACTTTTAGTCACAAAACTGGATAGGATTTTATGATCTTCGTAATATAATTTCAATAA

ATTTCCTTATCACAAATGCTATTTTCGATGCTGTTCTTATAGATTTAATCATATACATATTTCATGTATT

TTTTGTAGGATTTACGGTAATGGACAAGAGTTGTTGTACGGTAGAAGCAGGGGAAGAACTATGTGCGGCG

AATAAACCGGTTTGTGCGTTTCGAAGACGATATGTGTACTGGGACAATGTCCACAGCACTGAGGCGGCTA

ATATGCTTGTGGCTAAGGCTGCATTTGCCGGAGTCCTTACTTTTCCTTACAGTATTGCTTTGTTAGCAAA

GTTATAGGACAAAAAGATATTCACATATTTCTTTTTAAAATATATATGTATGATAACCTTATTCTTTAAT

TCAACTATACAAAGCAGCTAGTATGCAAACATACATGTTGCCTTGTAGGTATCATATATGATCTATTGAT

ATAATTTTTTTCCAAATGCATTGCTAAATTAGTTTAGTATTGGTATAGCTAAGTCAGCTTCATACCCAGA

TCATATATACCAGCTTTTTCTATGGACAAGCTCATCTCTAAATTTTATGCCAATTTTTTGTTATAATTAA

TAAGCACAACAATACAATGTTTACATGTATTGGGTAAATTTATTGTTACAATTCAAGAACCACAACAGTA

GTAGAATACAATCCCTACTCGAATCCTTATTCAAATGAAAATGCATAAATATATGAAGACTTGTGTGTGT

GTGTGTGTATAAACCTAGATATCTTCATAAAAATGTTTCTTGAAGGCCTCCATGTGAGCCACTTGTAGAC

ACGTGGCGAGAATCAGCGAACCGTCTTCATTTTGATCCCGCAATAGCAGAGAGTCGCCGTCTAAGTCATG

CGTGCCTGGTCCCATGTAGACCTCCTTGCCCCATCCGAAATCAATGCCGTACATCGGCAGAGTTAACCAA

CTCACCAGTCCAAGATTAGGGTTTCCATAGAATGGACCTTCCGTGCTTCCCAAGGCATGTATATCTTGAA

ACTTCTTCAGATCCTCTTGGATCTTTAGATATTCTATCCCAACCATCACGTATTCGTTTGTCACATTCTT

TATGGCTTTACTGATTTTTCCCGCCGCGAAACCCAACTCATTTGATATCAATTCACCTATATGCGTAACA

AAATTAACGAAATATAGCTTTTGCATTATAGGATCCCTATATCCAAAACTAACCTACTCTTAAGAAGAAA

AAAAAAGTAACGTACCCTACCTTAAATCCCAAGAAACAGGAAATCACAAATACAATAGCTGAAAATTAGA

CAAACGAACCTGAGGTGCTTGCTGCGACCACATCAAGCGTGGCATTGCCGAAGTAGCCACGTGGCAGAGG

TGGCTGCATCCGATTGCGAATATCTACAGAGATTCCCACAGACGCGGGTTGCTCCGGCGAGTGCCCACGT

GCTTTACACGCGCACCTCCATACGTGTCCCGAGACCGTCTCGTACCTTGTAAACCCTCTCGCTGGATCAG

CGTATTTACTTGTATTTACTCTACTTCTAAGCTTCTCAACCTGAGATTTGCTCAGTTTTAGCATCGCCAC

AACCGTTTTCTTCTTTCTTTCTTCTCCATTGTCCGTTTTTCCGATCAGAAATGGAGGCTGTTCAAACTCT

TCGTGGTCGAAGTTAGGTGGTGACTCAAACGGCGGAAGCGGTTCATCAGCCCAGAGGACTCTCCGGTCAA

GAAATGGAACGGTTTCTAGCGGTTCACCGCGCGCGATTCTTCCCCATTCCCTCATGAAGTGAAGCGCACT

TTGGCCATCGACCACCGCGTGTGAAACGTTGACGCTGAGGCTCAGTCCGCCGCATTTGAATTTGGTGACC

TGAGCTAGAAAAAGAGGAATCGTTTCGATAGGGTTTTTGTAGTTTACTTGCGGCATAAGTTTCTCATATT

CCGGCGTCGGGCAGAAATCGTTGAAATCGGAAAGCTCGGCCTCGGATTCCGCTTCGATGAACGTCACTCC

CGCGGCGTTACAAATGAGCTCTAACCGACCCCGTGGAAGCCACCGGAGACGTCCTGCCATAGGGTAGAAA

TAGAAAAGCGCACGGCTCAATGAATTTTTTAGGGTTTCGACTACATTGCCTTGGAATGATTCAGATGGCT

TGTCGTAGAAGTAAACGGTGGGAACGTGAGTTATTGTACCAACTTGATCCCTTTCGGCTAATGGATACCG

ACCGGTCCATGTTGGTTCAGCCGGTACTATGGTGTAAGTATTTCTCAAGACGATGGGAGCCATTTCATAA

ACCGTTTTCTTTTCTTTTCTCTTTTCGGAATTCGTTGCAGAGTATTGTTTATATAATATCATTGAAATCC

ATGGGTTTATATCATAAGTTTATAGTACTTAATTAAAATGTCTTTCTCGTGGTTTAGTTTGTTTCCTTGT

AAACAGCTTCTTTAGTGGAAGTGTGATAATAAGGTTTCTTTTTTTGGTTATATGATCATGCTTCTTGTCC

TTTTTTTTAGTTTCCATTCGCAAAATGATGAACATTCCCATTTAAAAAAAATATAAACTTGTCTACTTTA

ACCAATTTGTCACGAGTATTTCACATTTACAATTAAGAATCTTATAATATTAATACCCAATCTAGATATA

TAGGTAATGTATTAAAATTTTATAATTGGTAAAAGTATTCAACTTTCACAGGTTTAAAAAAATAAATAGT

CTGAAATTTCAAAATTTCAGGTTACAAGGAAACAAACACTCGAGTCATCGTGTTGAAAAAGGTAGTCCAT

GGGCTATACATAAAGCCCAAAGTGGATGGACTGTTAATAAACCCTAGCTTAAACCCGACTGACTAATGTT

GACCCGGACTCGAACTGGATCTTCTCCTCTTCTCGCCAAGTTCAATTTGACGATCAAAACAAGTACACCA

CTATTCCCCCACGAACCAGAGCTCGAAGACGACAATGGAAGGCGTCGAAGAGACGGCGAATTTCACTTTG

GTGGCGAGGAAGCCTTGCTTCGGTCTCCCAACAGCTTGCCCTAGTTGCCTTCCCGCTTACATATACCTGA

AACTAGCCCAGCTTCCTTTCGAACTTGCCTTCAATTCGATCTTCCCTGATTCAGGTTCGATGTGTTTTTG

GGGTGCTTGGTTGTTAGAATAAGTTTTTTCGTTGCGTGATTGAAGTGTAATGCTTGAATTGAGATTCGAA

TCTTATACAAGATAAGTTTCAGCTCTGGATTCGAATTCGTAGCATTAAGCTCTTGCTCGTCTATTAATCA

GAGTCTGAACAGGGCTTTAGGCTATAGTAAATTGCTGTTTGAGGTTAAAAGGATTCGTTAAGAGAGAAGA

AGACATTGGTGAATTTTGTTCTCGCTTTGGTTGAATTGTTTTTCTAAATTAGAAGAGGTAATAAGTTTGT

ATATTAACAGAAATTTTGTGTACGCATTGTTCAGCTTAAGATTGTTCATTGATTTACAAATTTGAGCTTT

GGATACATTCTCATTTGTGTATTTGTGGTTTTTATGCAGATGAACTTCCGTACTTCGAAACCGGTACATA

TGTTGCATACAACAATGAAGATGGAGGAGTGATTGAAAAACTGAAGAAGGATGGTATTGTTGATCTGGAC

TCTCAGCTCCAGTCTCTTCCCGATTATCTATCGTTGAAGGCTCTTATCGTTTCTTGGCTGGAAGAAGCGC

TTACTTACGAGCTATGGGTTGGGACCGAGGGAATATCTGCGTCGAAAATCTACTACGCAGATCTTCCATG

GGTGATCAGCAAGGTCCTGTTTTATAAGCAGACGTACATGGCCAAGAACCGTTTAGGGATCACCAAAGAA

AACGCAGAGGAAAGAGAGAAACAGGTAGCTTCTATTTTTCGAGCTTTGTTGTTCGTATGACAGTAAGTTT

CGTTTGCTGGTAAAGAACACATGTGGTTCATATTTGTTACTGAAGCTTAAGGTGTTAAAATGAACAGATT

TACAAGAGGGCTAGTGATGCATATGAAGCTTTGTCGACTAGGTTAGGCGAGCAGAAGTTTCTCTTTGAAG

ACAGGTACTGCAAGCCACATTGACCAAACTTGATTCTTTCTCTTATACCTTTTTATGATAAGATTGATTG

ACTATGCTCTTCGCGCAGGCCATCGAGTTTGGATGCTATCTTTCTCTCGCACATGCTTTTTATAATCCAA

GTTTTACCGGTAAGCAGCTTTACAGAATTCAACTAGTATACTTTTCGCTTATTGGTTGCCATCAGGTAGA

ATCTATGCTTTCCTTGAAACAGTGTAGACTAAGTTTATTTCCGTTATGGTTGCATGATCAGGAAACATCA

GTGCTTCGGTGCAAACTTCTGGAACATAGTAATCTTGTCAGATATGCTGAGAAACTGAAGTCAGAGTTCC

TCGAAGCCTCTTCTTCATCTCCTTCGCCTCCGCTTCAATCATTCCCTTCCCCGTTTTCAAGAAAGGGTAA

GAAGACACTTCCAAACGTTACATTTCTCATGTCATGTACTCTTTTCAAATCTCATGAATTTCATCGGTTT

GTTTAGGTTCGAAGCCAAAGAGCAAACCAAAGACTGAAAAGACCGAAGAGGAGAAAAAATTCAAGAAAAG

AGCAAGATTCTTTCTAGCTGCTCAGTTCCTAGCCGTCCTTATTTACTTATCCGTCATGGGAGGAGGTAGT

AGTTCCGATGAACTGGAGTATGAAGATGAAGATTACTAAAAAAACTGAAGCTTCTCTTAACTCTCAGGCA

AACTCTTTGACCTCTTCTTCCTTAGACTAAGAAAAAACAGAAACCAATAATTTATGTGCTTTGTTTGTTG

AAACTCTTGAGTTAGAGAGAGGATACAAATCCATGTTATATTGATTTTTCGTTCTGAATGAGAGATTCTA

TTGGCTTTTTATACAAATTTCATTGGTTTTAAGGCGATGAGGCTCGCAAGTTGTCACATCAAGAGAACCT

GTTAACCGGATAAATTACCGAACTAAACTCTATTTGTCTTACTAATACAAATCTCATTCTTCTAAAAACT

ATCTTCTGATAGCAAAAGGTGATGCCCAAAAACCTACCTAAAGCTAAACTCTTCTAAAACCCAAATAATA

ATCAAATAACAATTAGGAATTCAAATTTACATCCGGAGGTTGCTCCGGCGAGGCGTCCTCCGGTTGGTTA

ACAAGCTTTGAGTGTTCATCGGCAGAGTTGATAGCAAACTCAGCCAGTGAACTCACTGCAACAGACATTC

CATGGCAGAGAACTTTAACAGCAACCTCTGCAAGTTTCTCAGCAGTCATGACAACATCTTCTTCTACACT

ATCAGCCACCACAAGGTCCATGCTTTCTGACCCGGAATATCTTCTTGACCCGCTTCCGCTCCAGTTTGGA

TCTTCCCTAACCTGTCTTGCATAGATTGAACCCATCCCGGAGGCAAAAAAGTCGAGTTTGTCAAGAACCG

GCTTCTCGTTCAAGCTCTTAAGTAACCTTGACCATTGGATGCAGACTCTATATATCGGATGAGGACATGA

GGATAATCTAACTTTCTCAGGGTCAGGATCACATCTGAAACATCTGAGAAGCCAACCGGATAATGCTGTC

ATGTAGGATCTTTGAGATGTGATCCAGAACTCGAAACAAGCTCTCCAGTTTCGAAGCTGAGCTTCAAGAT

TCAATGCAGATTGAGCTAATCTTTGTGAGTTGATGGCCTCTGGCATGATTGGAGGTTGTCTCTTCTTGTG

CCGCTTCGAAACTGGTGTCCCTGCAAGTAGCATTTTAGCTTCGTCTAGTGTTCGTTTCTGTATCTGATGA

CTCTCTGCCATCACTTGCCACATCTTTGTTAACCTGTAATGATCATATGATTACCATTTGAGGAGGGTTT

AAGAGAAGAAACCAAAGAGGAAGAAGAAGAAGTTGTTGGTATTACCCTTGAACAAGCTCGAGAAGCTGAG

GCAACAGTTCTTGATCGCGAAGAGTCTCGATCCTTTTGGAGATAGATTCAATCGAGTGTATAGAGACCTT

AATCTGAGTATCTAGATCTCTGAGTGTAGCCCTTGTTTTATCAACCGAAAACGGATCATCTCCTTTAACA

TCTTGATTCCTCAGCTGCATACATTTCTTCTCATACGCCCTTCGAACACGTTCTCCAGACTGTAAACAAC

ACCAATAAAAAGGCCATTCTGTTCATAAACAACACAGATTTTGAATCTTTGTTACTCAAAACTTTATCTC

TTTGTTACTTACTTTAACTTCATCGTAGAGTTTCTTCTCCCAGGCGAAAAGCCTGTCCAATGTTGTTTGG

TGGCTACCTGAAATCATGCAAGATTCATCTGAAACATCGCTTCTGCTCTCAGATCCACTCTCCTTCGAAC

CACCACCAGAAGAAGTGATCAAGAATCTTGCAGATGAAGATCTTGACGAACCCGAACGGAACAAAGCTAC

TGGATTCAGCATTTTCATAGCTGCAAAAGATAAAACTTCCTTCAACATTCAGTGAGTGAAAGACTGCAAA

AAAAAATTCAAGAACTTACCACTATGATCATTAGAGGATGGTGCATACTGAGCTCTACTAGCTTCTAGCA

ACCCCGAGACTTCTTTAGCCGCGTCGCAAATAGTTGTGAACTGATCTTCAAGATCTTTGATCACCTCTGC

CATGCTCGTTGGTCTTCTGTTCACATAAACAGTGAAACCTGGAGGCGTCTCTCTTTTGGCAACATCAACA

ACATTCTTCATCTCTTGACCACTCTTCTTCACAACTCCAACAACATTCCCTCTAGGCACCTCAACACTTC

TCTGTTCTTGTTCTTGCGTCCCAACGCATTTCTCATCTCCTCCTTCGTTCTCACTTTCACAACCACTATC

GGTAAACTCTCCATCCTCATCCTCATCATCCTCGTCCTCATCATCATCATCATTCTCATCAACATCTTCA

ACTTTCACTTCTTCCTTGCAACAACTCTTATCGACTTTACCTCTACTCTCTTCCGCTGCTTTCGGATGAT

TATGATGCTGAAACCTCACAGGTAGAGGTTGTTCAATAGGCTCATCTTCTTCCAAATCTGGAATCCCTTC

TTCCTCACGAACACGTCTCAATCCTCTAATCTCATCATCCATTATACCACCACCACTCCGATTATCAACA

CTTCCTCTATCATAACTATTATCATATCCATAATAATCCAACGAAGAGAAGGGATTCCAGAAGAAGTCCC

ACTGCGAATTCTGCGGCGAAGGAGGAGGAATGTTATGACTACTGCTGCTTAATCTCTGCTCAGGAGAAGA

GCTCAACGGATTCCAAAACGAAGACGAAGAAGCTGCAGAAGTGTTCATGTTCATGTTCATGTTCATTCCA

AAGAAACCATCTCCTTCTCCTCCGTATTGTTGACGAATCGGAGGTGAAAATGACTCAACACGAAACGTTT

CTGCAGATCTCTGTTCGACGCGAACCGGTCTGCTTCTGTTCGCCATTAAGTAACTCGCTCTCACCTTCGA

TCTCGGCTTATCTTGAATCATTTTAGCTGGATGTGGAGGCATCGAAGAAGGCGAAATCGTTATGAAATCG

CCGCCGTTGCTCCGGCTGCTGCTGTTGCGGCGGCGGCTCGGTGGTCTTCTCTTAACCGGAGTGACGAATG

AGTCGTGATGACTCGGAACAAACTCGTGAGGCTTGTAGTTGTCTCCTTGGACAAAGTCATGGAGAGCATC

AGAGACTTTTCGAAGTGATTGGATATAAGCAATGTGACCAGAAGCAAACTTGGTTCTGTGTTCTATCGCT

TGTTTGATGAATCGTTTCCTGTCTTTACAGATCTGAACAGCTTCTTCGTCGTCTAGCTTTGAATGAGAAC

ATCCCATTTCTCAGCTCTTCTTCTTCTTCTTCTTTTTATTAAGCTTCAAATCCACAGATCAAGCACTTCA

TATCAGTGGATAAATCGACATAGCTATTTTCTTTTAACTCACATTAAATGAGTAAAAGACATTGTTTGTC

TCGATGTCTGAAGAAGAAGAAGATTCTTTGCTTAATTTACAAGACAAAAAGCTCTTCACACAGTCTTTCT

TTCAACTCTTAAACCCTCTTCAAGAAAATCTAAAACCCAGTGCACAGATTTTTGCTTATTTCCTTTAAAG

AGACTGAAAACCGACAGAGACCCGGATGAGAAATAGTAGAAAACAAAGAAAATCTAATCTTGGTTTTGTT

CTAGTGGAGTGCCCAAAAAAAAAGAAGCAATGAAACAAATCTGGTAAAGAAGACTTAGATTAGAGAAGAA

AAAAAAAAAAAAAAAAACTAATAGGGTTATGATATGGTCAAAGAAGCGTGAGGATTACTGTGGAGGAAAG

AAGAGACGTTGGAGAAAGCAACGGTCAAAATTACTGTCGGATGTCGAAGGAGAGAGAGTGAGAGACTCTG

AGAGAGAGAGAGAGAGAGAGAGAAGTCAAGAAGGAGAAGAAGACAGAAGCAAAAAAACATAAAAACAAAG

GCAAACCAACCAATAACAAATTTTCTAAACAAATACATAAAGGAAATGGAAAATGGAAAATGGAGGTGGG

AGGAGAGGAGAGCTAAGCTAATTTAAAGCTACGAATAATTTTATAATATGGCAAAGACGAGACGGGACAC

GGAATACGACTACTTGTTTTGTTTATTCACCCTCGCTTTTCACATATATTTTCGGTATTGCCACTCTCAA

ATTTTATTTTTTTCCTTTTTTCTTGTCTTTTTCTTGTACTATGCTGATTTTTTTATTTCATTCTTGCACG

TTGTTTCATGGCATTATACAATATAACACTTTATATAAAACGTCATAGATCAAAATTTTGTACTCATAAT

AAATTTATAGACCTACCAACAAGTACAAACTTTATGTAATTGTGTTTTGTGTTTTCCTATTTGGGTAATT

ATAATACAAAGCATGGGCATTTATGTAATTTGGTTAGCATCTGCAGTGGGAAAATACGTTGGAAGAATTT

AGGAAAAGCGATAAAAAGGAAGGACCGGCCTGTAAAGTAAAAGGCAGGATCTGGACCCACACTTCTAAAA

GATATCATGAAGTGCCTCGATGCCTGAGATTTCTCTTCATGTCCCTCACTGTTTCACTTAATCACGAAAA

CACCAACCTCTTTTATTGGGCTCACGTTTACATTAATATCTGACCGAATGGACCTTATTATCATGAATTG

GATTATAGAAATAACCCATAGCATAGTTTGTTTTTGCGTAAACCTGAACTTAATCCAGGCCCAATCTATT

TTATTGGGTCTAGAGTGTCCACTGACCAAAATTTTCAGACTCGTATTTTTCCACCGAAAGAGTATGTCGT

ATAGACTCTGTTAAACACTTAAAATCAAGAAAAAATTGGTGAAGTAGGCACATTTTAAGTTTCTAATTGG

GAAAAGAGGCAACACCAAGTTTTTATTAGCAAAAGAGGCAACTTTCTCGTGGGCCCCGCCATGTGTGGTT

GGTTTGGGTTCACTTTTTGGGACTTAGGATTTATTTTTTGTTCATGGAAAAGTGTTTTTATTGTGGTGGG

ACCCACCGATTTGCCTCTTTTGCTAATAAAAACTTGGTGTTGCCTCTTTTACAAATTAGAAACTTGAAAA

CATCCTCTTTTGCTAATTCACCCTAAAATCAAGGTCTCTCCAACACTTAAAGCCTACTCTAAGCTTTATG

ATACTTCCTTTAAGTTTTCATAAATTTTAGATTCTCCATGCTCTTAGCCACAACTTTTTTAAGATCTATT

AGATTTTTTTTTGGTTAAAAGGCCTAAAAAAAACAAAATTTGGGGGTGGGAGGGAAGGCGAGGAATCGGT

TAACCGCAGACACTAAACCTAGTTTCAACAGAACCACCTACGAACCAACCCAACCCAATGGTCTACTTTG

GAGATGAGTGTCTTATGGAATCATCGATAGAGTATCTTTATTAAAAAATCACCAATGACATACGTAAAAA

TTATCTCCGAATAATGTAACTTCATTTTTTCCCTGAATTAGTGCTTGAATCCAGCAATTTCATTATAATG

GCATTACCATTTAGGTCTCTGGTGATGTTTATGTTTGTATCACCATTCATTAGTTATATAATAACATATA

TAATGCTAATGTTGGGTGTTGATAGTATGTCCACTATATAACTTCTTCGTGTTTCTCACTGTGTACATAT

CATGATAAGAAACTTGTATCTATCTTTGCTCAAAAAATAAATAAAAAATAAAAACTTAATCTAGACCTTG

TCCCCATTTTAATATTATACACTTATTCAAAATCTTTATATATGTGTAGATATATATATATATATATATA

TATGGTTTATTTATATACTATTTCAAACAAAAAAATTGTTACAATTAAATGTTTTAACTTTTAACATGTT

GGTGCCAGGAATTCAAAGTCATTATTTTGATGATCTATATATGTTTCTTGTAATATGCGGTTTCAACAAT

GTTGGAAGTGAGACGGAAGACTTTAAAAGATTTGTTTGGAATAAAAATCTCGCCATAATTAGAAAAATCT

TATCGTATGGTTTGAATTAAACAACTAAGATTTATTTTCAAGATAAGTTACAAACATAATTAAAAAAATT

GGGAAGCTAATATATCGAAGATAATTAATGCTATTTCAATGTTGAGTTGCGAATTTAAGTTCTAATTAAG

AAGAGAATTCACGTTTTATAGAACTGCCGCAATTTTTTTTTATCTTTCTACCTGATTATTCAAGCACCGA

GCATAAGTTATGATCTTGTGCAAACATGTTACTAATTTAATAAGATGTATCAAAAATATATAGATACATC

TTAGAAGAAAAGCTAAGAGAGTAGACGACAATTAATTGGTGCGTTGCGTTAAAAGAATGTGCACATAGTT

TAATTAAAATTTTAGAAATAAACTAAGAAAATTGTACTAGAAACCAAATAAAGAAAGCAATTAGATGAGG

AATCACACATGGATTCCATTTTGTGACATTACACTTTTGGTGTTTTCCTACTAACATTTTACTATTTTAG

TAACTTTAACTTCGTGTCTCTCACTCACGAGATTAAATCCCCTTTTGATCAAATTTTCTGCTCAATTCTT

TCTTTAGATAACTAGCAAGAATCATGATTATAATAATTCCAATTCTTAGCATGCGATATTGCGAGGATCA

TGTGTCTAAACTAGCGATGTATCGGACAAGTATTATCCTCGCCCCATATTCAAACTGATAATGTTTACTT

TATAATCTCACTCTTCTTTTGTAACCATTTTATATAAAGTTTTAATAGATATTTACCATATTTTTATCCC

AAAAACTTAGAATATGTAGTTCTTTTGATAAAACTCTAATTGATCATCTACTCCATAAAAAGCTAATGTC

GAAATTTATAAAACAAAGTCACATGCACAAACAACTGATCTTGTCAGTGAGAATGTTTTTACTTATGGTT

CAAATCTCAAAAACATAGCCAATTCAAATTTTATGAAGTTCGGTAGTACTATAAAATGAGCAATCAATCG

TTTATAAAAAGGAGCTAGATTAGACTAGTCTATAATCCATTATAGTGAAAACCGTTACCACAAAATATCA

TACTTTTATTTAGTGCTGATGTAATCGATTTTAAAATAAACTTATAATTTTCTATTCTTGGAAATTAATC

ATATGAACTAGCCTGAAAATTCGGAAGGAATATAGGAATTTAAATCAAAAAATAAAATATATGTGCAATC

AATAGTTGAGAAATAAAAATGTACAATTTAATAAAATTCAGGAATAAATTGAGTGTTTTCCCCTAAATAA

ACATGGTAAACAAAACAAAAAAATAATGTAAAAGAAAAGGTTTTAATTCGAAAAGAATCCAAACTAACAA

CAACTTTAGAAAAGTTGCTTTTATGTTTCAAAAGAATCCATAAATGTTAGGTTTCGAACGGCTTTTAAAT

ATTTAATAACGATTCTCAAATCTTTTAGGGGTGGGTGTATTAAAATTAGAATTTGGAGTTATTTGATTTT

TAATGGGGTTTTAGATGATTTTAGAGAGAATTGTGAATATACCCTAAAAAAAGGCTCATTTTGTCTATTT

ACTTCTTTTTTGACATTGGTAAATTTTTGCCAAAATTGTCGTGTCCTTCACTATAATTAAAAACTGATAC

TAAATTCTTTTACAAGTGAACAAAATTTCTAAAATTCATAAAAATGATCTAAAGACTGTTTAAAAAATTT

GAACATTTTTAAAATTGTTTTTCATGTTTCAGAAAATTATATGAAGTTTCATTCTACCAATCTCTAAACT

CAAAGTAGAATACGTTTTCTACTAGTTACTCGGACGTCCAACGACTATATAATGCAGAATCTACAGCTAT

GTCATACTTCTACCACAATTAGAATTCGAAATCTACCAAATACTATAGGCTCTACCATTTGGTAGAATTT

GCTCCCAATATATTCTACAGAATATACTATCTTCTGCTAATCGTGGATTTCAAGTTCTACTCTAAACACT

GACATCTACCTTTCGTAGTTTTCGTATCCTACGTACTACAGTTCATTCTACCTCCCGCAGAATTAGTGTT

CTACTAAAAGCAAGTTTTCTACTATCCGTAGTATTCGTGTTTGTACAAGATGTTCTACTAAAATAAGTAT

CATTATACCTTTTGTATAAATCATCTTCTACCAAAATCGAGAAAATATTTCAACTGAATATTTTTCAAAT

CTTTGTTTCTTTTTTTCCTCTAACTATCCGAAAATAAACCAAAATGGTTTAATTAATTTTTAATTTTTAA

TAATGAAAATAGTTTAATTAAAAATAAAACCTTATCTAGTTAATTTGAAGGACAAAAATGGATTATGTGA

TAAAAAAGGTGTAAGGGGACAAAGAGCAAATGTGAAAGAGTAATGGGACAAGATAGGTTTCTTTTAGGGT

ATTTTGGCAATTTTCTCATGATTATGTGAATTATAGAAATTCATGTGATTTTGGTTAAATCATTCTAAAA

TCTTATCTAAAACCAAGTGATTTTGAGTCTTTTATTTTTAACAAGAAAATCTCACAAAATCACTCTAAAA

TCAAATCTAATTTTAAAAATCTACTTTTAAAAATATTTCTAATAACAGTCGATTTGAAAGTGGATTTTAA

AATCACAAATTCAATAACACTGAATTTTAATAGGGTTTTTAGAATTCAAGTTTGAATAACACTAAATTTG

TTATTTTAATATAAATCACCGTAAATCACTTCAAATCTCAATTTAAATACACCATTTCCTTTCTTAAACT

TCTAAGAACCAAATTTGTGGTATATATTTGATATATTTAATCTATTTTTTTCTTCCAACTTTGATCTAAT

TGAATTTTGTTGGTGATTTTGGAAAAACAAAATATAAATTTCGGCACTGTTATGGGTCAATCTCAAAAAA

GGTTTTCCTACCAAACTCAAACTGGAATAAACACCGGTTCCCGGTTAGACCGGTCCGACCAGCCGGTCCT

ATCTGATTTTTTAATTATTGTTTTTAGCTATATATATATAAGGGACTCATTTTTGTTGAAAGAAGATAAA

GTTAACAAAAAAACTTTTGCCTTCTCTTTCGATCGAATACTATCTCCCACTTTCCTTCTCTCTCTTTTAA

TATCACACCTTTATATATAATATTTTATAACATAAATAAAAATTTTAATAAAAAGCTGATAATTTCGAAA

ATGTTAAAGAACAACAACCAATCATCATCTATCATCTAACATTCTCACTTATCTGCAGAGGCGGCTTAGA

GGTCAATAGGTGCTCTGCACTAGGGACCTAAGGAAAAACAAAATTTTAGTATAGAAAAAAATATGGAGAC

AAAAATTAGTATAGAAAAAAATATAGAATCTTTGATTAAGATTATAGTTTTGATTTTGCAATTAAGACTT

ATAAAATCTTTGACCCGGCCCTGCTTATCCGGCTATATAAGCAACTACCATTTCTAGATATCTTCACCTC

ACAATCTTCCTCTCTTCGTTCCAAAACCTCTCTCACTCTCAGTCTTCACCTTTGTGGTAATACTTTAATC

TGGTCGAACCGCACCAAACCGGTCCGGTCTTTCTTCTCGGCCTCGTCTTTTCTCCGGTATTCTTTCTCTT

CTTAATTCACATAGATTTCATAACAAGTGATTTCTTCGTAAAAATTAAAATCCGATCAAATTCACGGTAG

TGATATCTCCAACACGTTATATGCATCCCAGCATAAAAGTTTTTCTTTCTTATTTTTTTTCCCCTTAAAA

GATTTGGNAAAATTAACCATTAATCCCATAATAATCTCTTTTTGCGATGTGATTTGTTTTTTTCTTTTTA

GATTTCCGTTTCACAGATTCGCCATTAATCCCATAATAATCTCGGTTTGTTTTTTTATTTTTAGATTTCC

GTTTCACAGATTCGCCATTAATCCCATAATATTCTCTTTTTATAATGCGATTTGTTTTTTCTTTTTAGAT

TTCCGTTTCACAGATTCGTTAATCATAAAAAACTTTGATACAGAAATGGCGTTACAGAAGGAGGACAAGA

ACAAAGAAGAAAATAAAATGACAAAGAAGAAGTGGCAGAAGAGTTACTTCGACGTTTTAGGAATCTGTTG

TACATCGGAGATTCCTCTGATCGAGAATATTCTCAAGTCTCTCGACGGCATTAAGGACTATACCATCATC

GTTCCGTCGAGAACCGTGATCGTTGTCCACGACAGTCTCCTCATCTCCCCGTTCCAAATTGGTAAAGCAT

TAGCTAATCACTTTCTTCGAATTTTTATTTTTACCTAATAAAAATAATTGAATCAAAAACCATAAAGTAA

TCTCACTTAACACGTAAACAATCACTTTACTTTTCTTCTCTTTCTGTTTTCTTCAAAATTAATTAATGGT

TTCGCGTCCTCGTTTGATACGCAAAGCCTCAAATTAATTTTTTTTTGGGAACTAAAATTACTCTATCTAT

CAGATTTACCATAAAAGCTTACTTTGACTTTACAAAACATTTATTAGCAAAATTCGTTTATCACCAACCT

ATTCAAGATTTAAGGGAAAATAGTTATCCTCAAAACTAGGGAATTCAGATTTTTGAAGTTTTTAACGATT

CTACTGAAAAACAAAAGCCCTATTATTTGGGTTTCTTCTCGAGAAAAAATAGAATATTGTTGTTATGGAT

TTTTTTTCATTTTTATTAAAATTAAAAGAAAATTCAAAAGTTATTTATAAATCAAGTTTTTTAAAGCTAT

TTTGATGGATTGTTTTAGGAAAATTGATCTAACCAACAATTGTAATTTTTTTTTTTTGTGTGTGTGATAA

AGTCTACTTTTTCAACATTAAAAACTAGAAATTGAAATTTACGGCTTCTTTATACAATTTTGCTCGAGCC

AGCATCTTTGTGTATAAAACTTTGCATAACTCATACATACCACATGTGACATGTCACGTGTGTACTGTGT

AGCATAAACAATATCTAACTGAGTATTCCAAAAACATTTGCAAAAGAAAAGTGTTCAGAAAAGCCTGTTG

AGTTATTTACCAGATCTTTTTATAATTTTGCTAGAGCCAGCTTTTTGTGTATAAAACTTTGCATAACTCA

CACATACCACATGTGACATGTCACGTGTGAACTGTGTAGCATAAACATAATATCTAACTGAGTATTCCAA

AAACATTTGAAAAAGAAAAGTGGTCAAAAAAGCCTGTCGAGTTGTTTACCAGATCTTTTTATCAAAATAT

TTTATTGGTAGTGGATCATACTCGTTACTTAACTATATATTTATTTTTTATTTGACTGAAAACCCATTCC

AGTAGTACTTTTTTTCCACTCAAGAAAAGTATGAATTTGATGTTAAAAAAAAAAAGTATTAATTTTTAAA

ACAAAATTTCTTACATATTGGTTGTTTAATCATTAACTTCCAAACAAAATTGCGGTGCAGCTAAGGCACT

GAACCAAGCGAGGTTAGAAGCAAACGTGAAAGTAGACGGAGAAACCAGCTTCAAGAATAAATTGCCAAGC

CCTTTCGCGGTGTTTTCCGGCATATTCCTCCTCCTCTCCTTCTTAAAATTTGTATACCCACCTCTTCGAT

GGCTAGCTGTCGTGGGCGTCGCTACTGGTATTTATCCGATTCTTGCAAAATCCGTCGCTTCTATAAGAAG

GCTTAGGGTCGACATCAACATCCTAGTCATTATCACAGGTAATACCCACTTTTCACTTTTTATTTAATAT

TATTATTTTTATCCACATCACTCATATTGCGTGTAACTACTGTATAATGATTTGTTAGTTTACTATGTAG

TATTAGTTGAGAAAGAAAATTGTGGTTATAGTAAAACTATTCAGGCCCTATTAATAGACCTATAATGTTC

TTGGAAACTTGCGAGTCTTTTACGCTGAATTTACCCCTTTATATGGTACTTCAGATTAGCTTACCTATAT

ACTACTGCTTTCCTGCAACACCTACCACTCCACGAAACCTTTTAGAAAGTTATCCTTTACTTTTTTCTTA

ATATTTTTTTAAAGTATTACATATGGGAAAAATATCAAAACACATATTTATTAATTAATAGATGCGCAAT

TATTACTTTATAGAAATTCAATTCTAGGAATGTAGCAATTTGATATTTATGTTGTATATGTTAATTGTAT

ATTTGAGTTATAAGTTGTGGAACTACATAAAACTACTTTATATTTTCTTTTTATGTAAAGTACATTTGAG

TAATAGCCTAATAGGATATAGAAAAATATCAAAATGTCAATGTTTTTAAAACCGGACCAGAAGGCGAACC

GGATAATCATCCGGGTCATGGTTCAATTTGGTTCGACCGGGTTGAATTCGGTTCATAATAATTTATGTTT

ATTTATTTTTAAATATAGAACTTTTATTTTTCAAAGTTCCCAAGTGTAAACACATACATAGAATAATTAT

TGTGATTTTACATAATTCTCTTATGGAAATATAATAATTCTTTTTTAACATGTAGTTTAAAAAGATAAAT

CTTTTACGTACACACAACATAGATATATAGATTTTATATATAACTATCGAGGCAACTAGGAAAATGGAAG

TTTCATGATCGAGAGTTGTGTGGTTCTTTGGGAAAACTTAATTTTTTTGGTTATTTTATACGAAAGTAAA

GGATTCGTTTGATTCTTGCTCAGTTTATTATTATTATTTTTTAAAAAAAAGCTGCAGTTACGTCCCATAG

AAGAAAAAAAGGTTAACTCGTATTTGATTGGCTTATCTTCTACGACTCAAAATGGGAAAAACTCAAAAAA

GCAAAGCAAACTTTTTAGTTTTAAGTTTTAACTCGTGAAAAGAAAATTAAAAAGAGCAACAAATAATTGA

AAGAACAAAAGCATCAAAAGTAAAGAAATTAATTCATAATTCATAGACTGATAACGGAGTTACTTTTAGT

TGAAATTTCGGTTTAGGACACCAAGCTTGTGAATCCATAATATAAAATATTTTTTTAAAATCTTGATCTT

TTGTTCGTTTGTATGATGTAATAGTCACTTCAACAAAACTATAACTCACTAATATTCCAATTTCATCAAA

CAGTGGCTGCAACACTTGCAATGCAAGATTACATGGAGGCTGCAGCAGTTGTCTTCTTATTCACCATAGC

TGACTGGCTGGAAACAAGAGCTAGCTACAAGGTATGTTAACTAGTAATCATCATATATTGTGTTAATCAA

ACTACTATGGATTATCTGAAGTTGAAATTGTAATGGATTATTGATTATGGCAATTGCAATCCCAGGCGAG

CTCGGTGATGCAGTCTCTGATGAGCTTAGCTCCACAAAAGGCAGTCATAGCAGAGACTGGAGAAGAAGTT

GAAGTAGATGAGGTTGAGCTCAACACAATCATAGCAGTTAAAGCCGGTGAAACCATACCTATTGATGGAA

TTGTAGTCGATGGAAACTGTGAAGTAGACGAGAAAACCTTAACTGGTGAAGCATTTCCTGTGCCTAAACA

GAGAGATTCTACGGTTTGGGCTGGAACTATTAATCTAAATGGTAATGTAACCCTCTTACACAAGCTTCAA

TCTTAGAAAAGTTTCAAGCTTTAACCTTTTTGTTTTGGCAGGTTATATAAGTGTGAACACAACTGCTTTA

GCTAGTGATTGTGTGGTTGCAAAGATGGCTAAGCTCGTAGAAGAAGCTCAGAGCAGTAAAACCAAATCTC

AGAGACTAATAGACAAATATTCTCAGTACTATACTCCAGGTTTGCAAAAAAACATAAACCATAACTTGTT

TTCTTTATGTTCTTGATTCTTGTAATTTGAGACCTCTCTGTTTTTTGTTTGTTTCAGCAATCATCATAAT

ATCGGCTGGCTTTGCAATTGTCCCGGCTATAATGAAAGTTCGCAACCTCAACCATTGGTTTCATTTAGCA

CTGGTTGTGTTAGTCAGTGCTTGTCCCTGTGGTCTTATCCTCTCTACACCAGTAGCTACATTCTGTGCAC

TTACTAAAGCGGCAACTTCAGGGCTTCTGATCAAAAGTGCTGATTATCTTGACACTCTTTCAAAGATCAA

GATCGCTGCTTTTGACAAAACCGGAACTATCACTAGAGGAGAGTTCATTGTCATAGAATTCAAGTCACTC

TCTAGAGACATTAGCCTACGCAGCTTGCTTTACTGGTAATAAAAACAATATCTTGTTCTAACCAAAAACT

AGTTTGATGAGATAACTTATGAATGACAATTTCTTGTTTGGTTCTCAGGGTATCAAGTGTTGAAAGCAAA

TCAAGTCATCCAATGGCAACAACGATCGTGGACTATGCTAAATCTGTTTCTGTTGAGCCTAGGAGTGAAG

AGGTTGAGGATTATCATAACTTTCCAGGTGAAGGAATCTATGGGAAGATTGATGGGAACAATGTTTACAT

TGGGAACAAAAGGATTGCTTCACGAGCTGGTTGTTCAACAGGTAAAGCTTCAAACTTTGGTAAAATCAAA

CTCAATGGAATGTTTTTGAGGTTTTGTTGAGTCCTTAATCATTTTGAAACTGTTCTTCCTTGACAGTTCC

AGAGATTGATGTTGATACCAAAGAAGGAAAGACTGTCGGATACGTCTATGTAGATGAAAGATTAGCTGGA

GTTTTCAATCTTTCTGATGCTTGTAGATCCGGAGTAGCTCAAGCAATGAAGGAACTCAAAGATCTTGGAA

TCAAAACCGCAATGCTAACAGGAGATAATAAAGATTCAGCAATGCATGCTCAAGAACAGGTATGAGACTG

AAAAAACCAAGAATTTTTCATTACTCTCCTAACGTTAAGAGATTATATTAAAACTTTGACATGTTCTTAT

ATGGAACAGCTAGGGAATGCTTTGGATGTTGTTCATGGAGAGCTTCTTCCAGAAGACAAATCCAAAATCA

TACAAGAGTTTAAGAAAGAAGGACCAACTTGTATGGTAGGAGATGGTGTGAATGATGCACCAGCTTTAGC

TAATGCTGATATTGGTATCTCCATGGGGATTTCTGGCTCTGCGCTCGCGACGCAGTCTGGTCATATCATT

CTCATGTCAAATGATATCAGAAGGATACCAAAAGCGATAAAGCTAGCAAGAAGAGCTCAGCGGAAAGTTC

TTGAAAACGTGTTCATCTCCATCACTTTGAAAGTAGGGATACTGGTTTTAGCATTTGCTGGTCATCCTTT

GATTTGGGCTGCGGTGCTTACTGATGTAGGGACTTGCCTGATTGTGATTTTTAACAGTATGTTGCTTCTG

CGAGAGAAGGATAAATCTAAGAACAAGAATTGTTACAGGGCTTCTACATCTGTGTTGAATGGTAAGAAAC

TTGAAGGCGGCGATGACCAAGGCCTTGACTTAGAAGCAGGGTTGTTCTCAAAGAGTCAATGCAACTCAGG

ATGTTGTGGTGATAAGAAAAGCCAAGAGAAGGTGATGTTGATGAGACCAGCTAGTAAAACCAGTACTGAC

CATCTTCACTCTGGTTGTTGTGGTGAAAAGAATCAAGAGAGTGTAAAGCTTGTGAAAGATAGCTGTTGCG

GTGAGAAAAGTAAGAAACCAGAGGGAGATATGGCTTCACTGAGCTCATGCAAGAACTCTAACAATGACCT

GAAAATGAAAGGTGGTTCAAGTTGTTGTGCTAGTAAAAATGAGAAGCTGAAGGAAGTAGTAGTAGCAAAG

AGCTGCTGTGGAGAGAAGGAGAAAGCAGAGGGAAATGTTGAGATGCAGATTCTAAATTTGGAGAAAGGGT

CGCAGAAAAAGGTTGGTGAAACCTGCAAATCAAGCTGTTGTGGAGATAAAGAGAAGGCTAAGGAAACACG

TTTGTTGCTTGCTAGTGAGGATCCATCTTATCTGGAGAAGGAGAATCTGAAAAGTGAAAGTGGTGATGAT

TGCAAATCTCTTTGTTGTGGAACTGGTTTGAAGCAAGAAGGGTCTTCTAGTTTGGTCAATGTTGTGGTGG

AGAGTGGTGAATCCGGGTCAAGCTGTTGCAGCAAGGAGGGAGAGATAGTGAAAGTCTCTAGCCAAAGCTG

TTGCACAAGTCCAAGTGATGTGGTGTTATCTGACTTTCAAGCTAAGAAACTAGAGATTTGTTGCGAAGTG

AAGAAGACTCCAGAGGAGGTTTGTGGATCTAAATGTAAGGAAACAGAGAAGCCTCACCACGTTGGTAAAA

GCTGTTGCAGGAGTTATGCAAAAGAGTATTGCAGCCACAGGCATCACGACAACCATCATCACCACCATGT

TGGGGCTGCTTGACGGAGATAGTGATTGATTACCTTTAAACTCTCGACCCATCCATCTATTTGCATAACC

TTTCCTTCTTCAACCAATGTCGCCCAGAACAAAATAAAAACTTATTTAGTGTTTCCAGCAAAGGTGTGAT

TCGTAAAGACAATGCTGTTGATCGTTGTTTGTCTTTTATGTTTGCCAAAACCATAATGTATTTCTCCTTT

TCTTGTTTTTATTCTCTTCTTGAAGATGCCCAGAAGAAGTTTGAACTTCGATCCTAGAGTCTTAAAATCA

AATAGAACAAGCAGTTGAAACATAACTTAGCCTTGGAGTCTTTTTGTATGCTGTGTACTACATAAGCTTT

CTTGACTGACACGTTTCTTGTCAATTCTAGGGCATTACTTTATTAGGACAGAGAAGGTGTTGCAGTTCGT

GTCCTGGAGAGTTTAGGTGAAAAAAAAATAAAGAGCAAAAACTGACTGCTCGCACATCCATGTAATCAAG

AATCAGTAAAAATAAAAATTAATCAAAGGGTGACACAGCTCATGATCTTATATGAATCACCAACCATACT

CTTCTCACTATATAAACAAATGTGTCATTTCTTGAAAACAATCTGAAATATTCCGCAATCGCTGAAAGCA

TAGCATTAGAGGCAAAACCCTAGCTATTCTTTTGTTCTCCGTCTTTATTTCTTATCTTTTATTTCGTCAA

GCTTGTCGATGAGGTGAAAACCTTCTTAAAATATATTATTCGCTTCTTCAAGTTTAATAAGACATATACC

CTAAGTTCCACAAACTTTTGTATCTCGAGATAAAACTTGTGACACATCTGATTCAACACGAAACCTAATG

TTTTTAAGATCTACTAGGTAGGATACTTGCGCTTCGCCGCGGAAGACTTTTTTTGTATTTTGATATTTTA

TATTTTGATATTTTAATCCAGTTTTCCTTATATTCCATCTGTTCCATATTAAAATGTCGTTTAAGATTTT

TTCATACATATTAAGAAAATATTAAAATTTTTTATTTTACTCATTATTACTCAAAATTAATCTTCTCAAG

GAGAAATGTGTAAAATCTTTGATGAATTCTCTTAGAAGGAAAGGAAGAATCATGAGTTTGATCTCCATCA

ATTTCAGAGAAAACTGCTTCCTCTTCCTCCTCCAGGTTGTTCTTCTTCTTCCTCCATTCTCTTATGCTCC

GACGCGGAATCGGCTTAAGACTTTTAAAGAAACACAGTCTCTAAACGCTCGATTTACGTTTTCCGGTTAA

ATGAAGGAAATCAATAAATGAAAACCGGATAAAATTAAACCAAAAAAATTGCTAACTGATATTATTTATA

ATATTTATATTAGATGTTTGTTTTAAATAACGTGAAGTAGATTATCTTGAGAAGCACATGAGATTTTTCT

GTGCTGATTAACTACAATATCTTTACAAAAAAAAACAACAGCAATTCCTCGAATGACACACGTAAAAATT

AACTCCAAATAATGTAACTTCATTTTTACCCCAGAATTAATGGTTGATTCCACCAATTTCATTATAATAG

CATTACCATTTAGGTCTCTGGTGATGTTTATGTTTGTATCACCATTCATTAGTTATATAAAATATATAAT

GCTAATGTTGGGTGTTGATAGTATGTCCACTATATAACTTCTTCGTGTTTCTCACTGTGTACATATCATG

ATAAGAAACTTGTCTCTAGACCTTGTCCCCATTTTAATATTATACACTTATTCAAAATCTTTATATATAT

ATATATATATATATATATGGTTTATTTATATACTATTTCAAAAAAACAATTGTTACAGTTAAATGTTTTA

ATTTTTAACATGTTGGTTCCAGGAATTCAAAGTGACTAATTTGATGATCTATATATGTTGCTTGTAATTT

GCGGTTTCAAGTTTTCAACAATGTCTGAAGTGAGACAGAAGTGAGACAAAAGACTTTGAAAGATTTGATT

GGAATATAAAATCTCGCTATGCTTAAGTATCAACTAGACAACTTATAACAGTTGAGACAATAATAGTAAA

CTATATGTTGGCATAGAAAATGGCGTTTTCTATATGAATTGGCTGGTTGATTTATATATTTACAATCTCT

AGTTCAGTATTCAGATGTTGTCAAAAAATCGAAAATAAGGTTAAATTGTGGTTTCACGGTATAAAAGATT

AAATACCAAAGTGGGTATACATCCAAACTCCGGAATATATAACTTACGAAAATTTCAATATGAATTACCA

AAAACAGTTCTAAGAAATCTGAATACACGTCTTAACTCTTAAGTAGTATGTTAGAATTTTAGATGTACTT

AGTCGACATTTTTTCAGTTTTCGATGTGAATTTAGGACTGGTTATCACTCATTAGGACAAAAGTGGATCT

CACAGGTTACTACTTTGCATCAATTCTATCATATAACTCAATGGTCATAATAGGCTTGGGCATTTTTACC

CAGCTCGAAATACTAAACCGAATCTGACCCATAATAGATGGAATCGAACCGAACCGGAACACGAATATTC

GAATGGGTCCTAAATTCCTATACCGGAAAGAATAGGACTCGAACCAGAACTGAATCGAGAACCAAAAGAG

TACCCAAAATATTCAAAATATAATTATATACCAAAAAATATTAGTTATATTTAGACTTAAAATAACTAAA

ATATGTAAAATTACAATTCTAAACTTAATATACTACTTAAATTTAGAAAAAATAACCAAAATATTCAACA

AATCCAAAACCGACCTGAACCCGGACAGAACCGAATCGGATCTGACCCGAAAATAAAAAATATTTGAATG

GTTTTAAAATTTCTAGAACGAAAAAAACTAAACAAAAAACAACCCAAACCCGATCCGAAAAACAGAATGC

CCGGGCCTTGGTCAAAAACAGATCGTAGAACGATATGCTTTTCTTTGATAAAGATACGATTATGATAATA

TTTAACGAATTAAGTATACTATTAAGCTTGACGCCTTGACCACCTCACTGATAATTTTGTTGCATTGCGC

ATTTGCACTTTCATAACCATTTTTACAACTTTTCTCAAAATATTAGATATCGATAAATAAATTAAAGGTA

TACATTAGTAATTTTCTAAGTCGTATTTATGTTTATTTGAATGCATCGCGATAACATATCCAACAATATT

TTTCTCGAGATTCGTGTATTAGTTTATACAATATTTTTTTAAAAAAATTAGACCGATCAAGAGGCCTCAG

CCACACCTAGCTAGGCTTCTAGGAATCCATTGGTTATAGTCATATAGAATTAACAATTTCTCAATTGTAA

AATTATAATCTTGTTTATTCAGATACGTAAAGGTTAATGAGTCATGTGACATTAATAATTGTCTACTCAC

ATTATTTAGAAGATTCAACGACTCCAAACTATTCTTGATAGTACAGTTGTTAAATAATTGGAGTACATGT

TGGGTCTTTGGTACGACTCTTGCTTGCATTGAAATCGATCATAACCATAGATTAACGATTCATAAATGCG

AGGGTGACATTTTTCCTTTAAGCCGCCAAACATTCACTTTTTATTACAATAAATTAAACTGAACGGTTTA

GGTTGTTAAGGTGAAAGTGTTATAATTTCAGTTCAGTTTTACAATAATGCAATTACCGAAATACTTGATT

TTGAGATATTGACATGGATTTATACTATTAATTTAAAGAAAGACAAAATTTAGATGAGGCACTTCACCCC

TGCTTAGGCTTCACTACGACCACTAAAATCTTATAACGAAGTTTTAGAGATTTACTCCTTGCTTTATAAA

TATGGATTTCGAATCACATTTAGACAATCACTTAGATTGGTAATTTTTAACTGAACGGTTTGGTATCAAT

ACATTTTCAAAATTATTAATCAGAAATACTAGTGTTAAACGAAAATATTCATATTGATGGTTGATTGAGG

AGGAGAGGTGGGCTCCAGCATAAAATAGCTGTGCATTTTAGGATGGGAATCCCGTTTTTTTCTGTACCAA

CTCAAATTTTCCAAACTTTTTGAAAATGAGAAGTAGCAATTTTCTGTTGGATTTATTTATTTTATTTTAA

TTGGTAAGGAAAAAGTACATGATAATGAGAAAGAAAGAAATACCTGAATTATTCGTTTAAAAATTAAAAA

TAAAAACAAAATTATTCATATTTTCCTTAAATTTTTCGAATATTTTATGTATTTACCCAAAATTTATCGT

ATTTTTCCATAATTTTTCAAAATCTCATCAATTTTTCCGATTAATTAAGACTTTGGTCTTTGGATGTCTA

ATTTTTAGTCACAAACTTCTTATTTCCTTCTTTTTCCCGTTATTTTCCGTCCCATTATCATAAAAGAATA

CTTCGGATAATATATTTTGATTCCCGAATTTTTCATTATCGTTCAGTTCCCGTCAATACCGGTCACAAAA

CCCAAAATGCAGATGGTTATGAACAAGTGTACTTTCTAATTCTACCTATCCACTTGCGCCAAATCTAGTG

AATATGTCAAAATTGGCAAAATGAATATAATAAACTATATGCCAAAAAAGGTTCATAATTTTTTTTATCA

GCATATACTGGTTATCTTTGAATCGCATATCAATAAGAAGCATCTCGTTTATCTAATTACCAACGCTAAT

GAGAGACGACCATTCCCTCATGTTCTCTCATTCACCCGCAAAATAGTATTGAGTTCTCCGCCTATGATAA

TCAGTTTATTCACCAGCCGCAACTCATCATTTAGTTGATGCGTACGCGACGATGATATGTAACACCTCCG

CATCCTTTTTAATTCTCGCATGAATAAACTGGTCCGTCGAATTAAGGACCTCTACATCCCCTATCCCATG

TCTTCAAAATAACCACAATCCACCGCTCTGACTCACTGCATCCACTCTGAAAGAACTTTCAAACCCCAAG

CCTTGACAAATGCTTCCTGCTCTCTCTGCACCAAAATGTGTTTCAAAAATAGCCAAAACATCCGTAAGCC

ACTTCTTCACCATATACCGAATCAATCTTCTGAAATAGGGTTTATTCGCCTCCCGACAGTTCCATAAAAT

ACAATTCATCATAATTTCATAGCTTAACCATAGAAATATCGGGGCAACTTGTTATGCTCCAATCTCCTCC

ATCTGCTTCTCCGACGATGCGATGTGCGTAACCAACGTCCCAATCGAACTACGTATCCAATAAAACCTCG

ATGTCTGTTTCACCTAGATTTTGAGCCAAGATTCCATTTTCCCGTACTTCACTTCCCTCCGTCACCACAA

AACCCCCAGCCCGACCAAGGTCCCCCTGTTCAACTCTGAGGCGTTTACCATTTGCAGATAGAGCAGTCTT

CCCCTTGGTTGGGCCAAAAACAAATCCGCTAGTGGGCCTACTAGTTAAATTTTGTTTGGGCCGAGTAAGT

TCCAAACTCTTCCCATTCCTGAACTTTTTTCCTTTATGCGCAGCTCTAGTGTAATTTGGGCCTTGTCCCG

ATCCAGACCCAAAAACAATCCCCTTCCCGTGTTCCTTACTCTTTCGGTGCTTAGTTCCTAAGACCGGATA

GACATTTTCCTTATCTCTGTTGCAAACATTAATGTTCATTGATTCCACTATCTTGATTGCGCCCGTACTG

CCCTCTCGTCCATTAACTGTCGAGTCCCTATTCTCTTCGGTTTCCATATTCTCATCGAGGTTTCCACACT

TATTCCTCAACACAATATTCAATGAAACTCTTTCCTCTCTTGAGACTCGCGTCTCCTCTTCCATCCCTTC

TTGACCAACCACCTCCGTTGGCCGTCCCCTACCTCGTCGAACCTGTGTGAACTCCTCCTCAACCGAAACA

GTTTCCTTCCCATTATCCCTATCCTGCTGGACTTGCATGTATGGACCAAGTGTCCATAAATTCCAAACTG

AGAACAAATGTTGGTAAGACCTTCATAGGACAAGAAATATCTCCCTCCGTTAATCAACACCGTGCCCTTC

AATGGTTTCTTTAGATTAACCTCAACGGATACCCTTGCAAACTGTGCTATTTCAAAGTTTAAAGTTGTGA

CGTCAACCTTCACCGGTTTCCCAAGACCCTTTGCAATCCCCTTTATGATAGCCTTATGATAAAAGTTCAC

CGGAATGTTCGATAACCTAACCCAAACCGGCGTTGTCTCAATCTTAGGATCAAACTGCGGAGACCACGCT

TGCACCATAAGATAACTCCCAAACGCTTTATATGGACCTCCCGTCAATGCCGCCATGTACTCCTCTTCAT

TCTCAAAATGGATCATAAAGAATTGACGAGGTAGATCCATGACATACATCGCAATTCTAGGTTTCCACAT

CTCCCTTAACTTCTTATTCATCGCCGTGATTGACACAGTTCGACCTAACACTTTGACAATCATACATTTT

TTCCACAAATCGTTCATGGCCGCCAACACTTCCTCCCTAATCGTTATTACTGGTTCTCCATCCTCACCAT

TCGGAAATTCCAAACGAAGTCTTGCTTCTACAAAGGCCTCATCCACCACCGTCTCTGGTACCGGTCTCCC

ACCCATACTACTCCCCGTCATCCTCTGCGCCTATGAGCTTGTTAAATCTGGTGGTCGCCCCTTCTCCCCA

ACGTCATCCATGTGGGCATTGATGACCCTGCCACTCTCGACCCCTGTTTCCTCAATCGCCTCACTCACCG

CACTCATTAGGGTTGTTTTTATAAGTTTACTAGTATGTGCCACTTAGCAATAGACATACTACTAGACTTA

TTTAAGTAGTAATGTCTCAATTCTAGGCAAGATTTATACTTTTGTTTAAAACAGATTATTTATGCTTAAA

CATGATTTCGTAGATTGGTAGACCTCGGTAGGGAATTTGGCATTGATGAATTGATTGGGGATGACTCTAG

TAGTCTCGGTAAACATCTAGAGCTTTCCATTAGTTGCTAACATGTGGGTAATATGTACCAGTATCACATA

TTATATGATTTGTCACGTCTCGGACCATGATTGTTTTCAACAGGGGTCCAAAATAGGTCAAGGGTCAAAG

CGGGGAATCGAACTTGGGTCAGAGGTTTCAATTAGCATATTTTACCAATTTTCCTAGTGAGTTTTGCTAC

AATTTCTGTTTTTATAAAATGAATAGGGTGTCACTTAACATCGTATTTTTCTAAGTCGACACCACTGTCT

CGGACGCGGTACGGTTTGAATTAAACAAGTAAGATTCATTTTTAAGATAAGTTACAAAGAAAATATCAAA

CATAATTAAAAATTTTGGGAAGCCAACAGATTAAAGATAATTAATGCTATTCCAATGTTGAGTCGCAAAT

TTAAGTTCTAATTAAGGAGAGAATTCACTTTTTATAGAACTGCCGCAAATTTTTTTTTATCTTTCTACCT

GATTATTCAAGCACCGAGCATAAGTTATGATCTTGTGCAAACATGTTACTAATTTAATAATATGTATTAA

AGATATAGATACATCTTAGAAGAAAAGCTAAGAGACTAGACGACAAGTGCATTGCGTTAAAAAAATGTGT

AGTTTAATTTGAATTTTAGAAATAAACTAAGAAAATTGTACTAAAAACCAAATAAAGAAAGCAATTAGAT

GAGGAATCACACATGGATTCCATTTTGTGAATCCATTTTGTGACATTACACTATTGGTGTTTTCCACTAA

CATTTTACTATTTTAGTAACTTTGACTTCGTATCTCTCACTCACGAGATTAAATCCCTCTTTGATCAAAT

TTTCTGCTCAATTATTTCTTTAGATAACTAGCAAGAATCTATATTATTAAAAGAGAAACAAAACCTGAAA

GTTATAAGGATAACACTATATCCAAACAAGAAAAAAACAAAAAAAAAAAACTGACATAAACCGGGTTGAA

TCCTGTTAAAAGACCAAACAACCCATTATCTATACTATTAAAAGAGAAACAAAATCTGAAATTTACAAAT

TTATCACTATAACCAAACAAGAAAAAACTCAGTCACAACCAAAAATACCCGACTAGATCCGACCCGGCAA

CAATGAGAAGACAAAAAGCCCAAAGCAGTAACAAATAAATATTTTTCGGATACAATTTTAAGAGGGGTTT

TCTGGTCCGAAAATCTTCAAGAAATATAGTTGTTTGGCAACGGAGATACTCGATCCGACCCGAACGCGGC

GTAGAAACACGCGGATCTAACAAGAGGAGAGGTGACTTAAATTGAACCGACTAAATAAACCGAATATTGT

TCCAAAAACAAAAGAACCGGACGCTTACCAAAGCCCATAAATAAGTCCAATTAACCGGATAGCTCCTAAG

AATATGCAAAAAATTATCAAGAAAATTAGTTCTTTTTCGATATTTGAAACATAAATGTCATATATTTGAA

ACTTAAATACGATATCACAGCGTAAGAAAGATTTTGCGTACCCTGATTTAGCATAATAAATACTTCAGCT

TCGACGAATGAAAAATCTGCGTCATTTTCCATAAATATCGTTAATTAACTGTGCCAATCAAATACAAAAA

CAGAAATGTCATAAACATCAATAACAACTCGAATTTCTTTCCACGCAAAATCTAAGAAGTTTTCCACAAA

TTTCGCTAATTAACTAAAACGAATATACTTAATACCTAATCTAAAGCATGTACTAAGCACACAAAAGTGT

CGACATTCATGATAGTATTAAAACTGAATTTGAAAATCAGTTACCAAAAAGAAAAAATCAAAGATTCTTG

TGAAAATATCCCTACCAAAAATATACCGTGTGATAATTAATATTTTTAAATCATATTGATTAAAAACCTA

ATGACCCATCCTATGAAACTATAAATACATCGCTCACAGGGTAATTGTTACATACCTTGAAATTGCTAAT

GATTTTTTCCTTCTGTCTAATTTCATGTTTACTAATCACGATTTGTTTTATATGTAGGGGAATTCATGAA

TGGAAAAGCAACCAAAAGCTCTTTCTTAAATCACCAAGAAACTATGATACGATCAAAGGTTAATCCCCAT

CGATCGGGTCAAGAAAATAGATAGTTGCCCCTTATTGTTTCACATACCTTTATGATGGCAAGCCTTTCAA

AATAGGTTGGGGAATCCAAGTTAAGCTTCTTTTGGAAGCAATACACGTCCGCATCTGGACAAATGGAGCT

AATCCTAGCAAAAGAAAATGTAAGTCAACGTAATATAATAGACTATTAGAGATTTTGTACACGTTTTCGA

TTCTGAAATCTTAATCTCGATATGTCTTGCAAGGTATGAAAACACAGGCTTATGAATGTCACCATACCTT

TAATTCGATTACGCGTGATTTTCATCGAAGTTTACAGAACCAACTGATCCGACGGATTCACCAAATCGAT

TTCATATCAGAAGACTAGATATCAATTTACGATCTTCGAGTCTACGCTCACGGTTGTGCGACTTTTCGTT

TTTCGCAAGATGGTAAACAAAAAGAGTGGATCCGACAAAGGCCCACTTTTTAATGGATAAAAACGACGAG

AAATGGGCTTATCCGTTTTAGACTCATACCCGAAAAGAATTAGTTCGGCCCAAATTTTGACCACTTAATT

TATCACTTTTAATCCTACACTAAAATTAACACTAAAATTAACACTGAATAATTGTATAATTCATCTCATA

TCATATAATATATTTTTTACCTAATGTATTTTAACGTATCAAAAATTACAAAATCTCGTGCTTCTCAACA

ACATACATTTCTTCGTTATATATCAGAAATATATTATTCTTTACATAATTACAATATAAATACTTTAGGG

GGGTTTATTGGTAGATGAATTTGTAAGAATTCTTAAAATTTTCAGAAATCTTTGTTATTGATTTGTGAAT

TCTAACAATCTTATTAAAATCTGTTGTTATTGGTTTGGTGATTTATAAAGTCAATACAAAATCAGTTGTT

ATTCAAAAAGTTTGTGTTTTAATGATTTCATGAATCCATTAAAATCCTTGTTATTGGGACATGGATTTTA

AACATTTTAACTCATAGAACAAGATTTCCAAAATACTAGCTATAACCCTTAGATTTTCAAAATTCATTAT

AACAAAATATTTTGATTGATTTTATGAATATACAATCTCTCTCCAAATCTAACATAAACTCTTCATAAAT

TTAACAAATCTCTTAACTTTCAAAATTTATCAACTCTATAGAAATTCATCTCCCAATAACCCCCCTTAGT

TTTCTTATAAAAAATTACAACATCATGTGTCTAGTCATGATTATAATAATTCCAATTCTTAGTATGCAAT

ATTGCGAGGATCATGTGTCTAAACTAGCGACGTATCGGACAAGTTTTATCCTCGCCCCATATTCAAATTG

ATAATGTTTTATAATCTCACTTTTCTTTTGTAACCATTTTATATAAAGTGTTAATAGATATATACCATAT

TTTTATCCCAAAAACTTAAGAGTATGTAGTTGTTTTGATAAAACTCTAATTGATCATCTACTCCATAAAA

AGCTAATTTCGAAATTTATAAAACAAAGTCACATGCACAAACAACTTATCTTGTGATTAAGGATGTTTTT

ACTTATGACTCAGCTAGGTTCAAATCTCAAAAACATAGCCAATTCAAATTTTATGAAGTTCGGTACTACT

GTAAATTGAGCCACCAATCGTTTTTAAAAAGGAGTTCGATTAGACTAGTCTATAATCCATTATAGTGAAA

ATTGCTACACAAAATATCATACTTTTATATAGTGCTAATGTAATCGATTTTAAAATAAACTTATAGTTTT

ATATTCTTGGAAATTACTGAAAACAAAAAGAAACTACATTTTGATAGGAACTAGCCTGAAAATTCGGAAG

GAAAATAGGAATTCGAAACAAAGATTAAAATATCCTACGAAATTAACATGGTAAAAAAAACTAAACCAAA

TAAAAAAAGTTTAACGTAAAAAGAAAAGTTTTAATTCAAAAGAATCCAGACTAACCACAACTTTTGAAGC

ATAATCCATAAATGTTAGGTTTAGAACGACTTTTAAAGATTTAGTAACTATTTTCAAATCATTTAGTACG

TCTTTCTTTTTTTTTTTGGCTCAAACATAACAGATTTCATTAGAAATGATTTCATTAGTACGTCTTTCTT

AAACTTCTAAGAACCAAATTTGTGGTATATATTTGATATATTTGAGTTATTTTTTCTTTTGACTTTTATG

TAATTGAATTCTTTTGGGGATTTCGGAAAAGCAAAATATACATTTCGGTACTGTTAGAAGGAGAAAATTA

CATCAATGAGGAGGACTTTGTAACTATGGTACATTTTGCCTTAACAAAAATACATTGTTGCCCTTTTATT

TGTTTCACCTTTAGACAATTTAACTTTATATTTGGGAAAATCGCATTTTAAGCCGATAAATGCTAACATT

TTAAACTTTTAAAGTTTTTATTAGCACTTCAAACACTCAAACTATTTTTTTCATACTTTAAACGAAAGTA

ACAAATTTTTACGCGCCGCCGAAAAAATTAATGAAAAAAATTTAATATTCTAAATTCAAAGAATCAAAAA

TTAAACGAAAAAGGCTTAATAACTTGAACTCAATCATACAAATTTTATTTACCCTATTTCGACTTATAAA

CATTCAATTATCTCTTTGAAACTCTGCATTGTATAAAAAATTGTTAATTTTATGGTTTAAAATACGAGGA

TAAATAGTTTGAGTGTTGTCATTGGTCATTTTCATTGTTTTAAAATGCAATTTCTCCCTTTATATTTTAC

TATTTTTTCAATTTTTACTTTTTATCTCTCAACAAAGAGATATCTTCTCTCCCTCACGATAAATCCTTCT

CTCTCTTCTTTTCTTTCTCCACTTTATTCATCTCCACTTTCCTTCTCTCTTTGCTTCCTACAAATTCTGT

CTCTCATTTTCCACTAGGTTCTTCTTCCACCAATAAAGATTGGGACAAGGGTTGCGTAAAGACATTATTA

TGCCTTGAATAAGTGTTTGTGTATGCATGCCCTTTTTCAAAAAAAAAAAAGAATAAGTGTTTGTGAACAA

AATATCTTGGATAGAATTTTAGAATATATCACGTAGATCGTGGACAATAGGTGTGTGAACATATATATAT

ATATTCAGACATATAACAATAAAATATGTACACAAGCTTCGATTTTCACCTCTGATACAACAATTCATCC

ACATCTTTATTATGTCCAAACAAAAGTAATCCACGGCAATAAATCTACATAATTTGGCTCTCTTTGATTC

TTCCACTCTTACTTTCATCTTTTTATTTCCGTAATCACAAGCAACAATGTTTCATTTTCACTTCTCGTTA

TCATCATATATTACAATTTTTTACCCATGTTAACCCTATCAAAACACCAATTAGATACATGAAAACAATT

CAATCCCACACCAAACCAGAAATCGTTTTAATTTATAGCTTCAGTTTCAGAAATTAATATACGCCAAAAA

CGTTGGTTACTGTGAAACACACACCAAACCAAAAATCACCATCACAGAAATAGCCAAATTTATCCACAAT

TGTATAAGAAAAAGTCTTTGCGTAAGGAGAGTTATAAAAGTAAATTTCTCGTACACAAGTGTCACCAAGT

GAACATACATTAGTCAATGTTAGTATTTTAATATTCTCTTTAGCTATATATAAGGACCCATCTTTGTTGA

AAGAAGATGAAGTTCACAAAAAAACTTTTGCCTTCTCTCTCTATCGAATACTGTTATCCCACTTTCCTTC

CCTTTTCCTTCCCTCTCTCTTTTAATATCTCACCTTTATATATAATATTTTATAACATTAATAATTTTTT

AATAAATCCTAATAGGGGTATGATATATAATTATATATGGTCAAAGAAGGTGGAAGAAGCGTGAGGATTA

CTGTGGAGGAAAAAGAGACATTGGAGAAAGCAACGGTCAACATTACTGTCAGATGTCGAAGGAGAGAAAG

TGAGAGAGTGTGAGACTCTGAGAGAGAGAGAAGTCAAGAAGGAGAAGAAGACAAAAGCTAATTTAAAGCT

ACGAATAATTTAAAGCTACGAAGACGAGACGGGACATATATTCACCCTCGCTTTTCACATATATTTTCGG

TATTGCCACTCTCAAATTTTATTTTTTCCCTTTTTTCTTGTCTTTTTTGACCCGGCCCTGCTTATTTGGC

TATATAAGCAACTACCTTATCTAGATATCTTCACCTCGCAATCTTCCTCTCTACGTTCCAAAACCTCTCT

CACTCTCTGTCTTCACCTTTGTGGTAATACTTTAATCTCTGATCGAACCGCACCAAACCAGTCCGGTCTT

TCTTCTCGGCCTCGTCTTTTCTCCGGTATTCTTTCTCTTCTTAATTCACATAGATTTCATAACAAGTGAT

TTTTTCGTAATAATTAAAATCCGATCAAATTCACGATAGTGATATGATATATGCATATATGCATCCAACA

CGTTATATGCATCCCAGCATAACAGTTTTGCTTTCTTATTTTTTTTCCCTTAAAAGATTTGGAAAATTAG

CCATTAATCCCATAATAATCTCTTTTTGCGATGTGATTTGTTTTTTTCTGTTTTAGATTTCCGTTTCACA

GATTCGCCATTAATCCCATAATAATCTCGATTTGTTTTTTATTTTTAGATTTCCGTTTCACAGATTCGCC

ATTAATCCCATAATATTCTCTTTTTATAATGCGATTTGTTTTTTTCTTTTTAGATTTCCGTTTCACAGAT

TCGTTAATCATAAAAAACTTTGATACAGAAATGGCGTTACAGAAGGAGGACAAGAACAAAGAAGAAAATA

AAATGACAAAGAAGAAGTGGCAGAAGAGTTACTTCGACGTTTTAGGAATCTGTTGTACATCGGAGATTCC

TCTGATCGAGAATATTCTCAAGTCTCTCGACGGCATTAAGGACTATACCATCATCGTTCCGTCGAGAACC

GTGATCGTTGTCCACGACAGTCTCCTCATCTCCCCGTTCCAAATTGGTAAAGCATTAGCTAATCACTTTC

TTCGAATTTTTATTTTTACCTAATAAAAATAATTGAATCAAAAACCATAAAGTAATCTCACTTAACACGT

AAACAATCACTTTACTTTTCTTCTCTTTCTGTTTTCTTCAAAATTAATTAATGGTTTCGCGTCCTCGTTT

GATACGCAAAGCCTCAAATTAATTTTTTTTTGGGAACTAAAATTACTCTATCTATCAGATTTACCATAAA

AGCTTACTTTGACTTTACAAAACATTTATTAGCAAAATTCGTTTATCACCAACCTATTCAAGATTTAAGG

GAAAATAGTTATCCTCAAAACTAGGGAATTCAGATTTTTGAAGTTTTTAACGATTCTACTGAAAAACAAA

AGCCCTATTATTTGGGTTTCTTCTCGAGAAAAAATAGAATATTGTTGTTATGGATTTTTTTTCATTTTTA

TTAAAATTAAAAGAAAATTCAAAAGTTATTTATAAATCAAGTTTTTTAAAGCTATTTTGATGGATTGTTT

TAGGAAAATTGATCTAACCAACAATTGTAATTTTTTTTTTTTGTGTGTGTGATAAAGTCTACTTTTTCAA

CATTAAAAACTAGAAATTGAAATTTACGGCTTCTTTATACAATTTTGCTCGAGCCAGCATCTTTGTGTAT

AAAACTTTGCATAACTCATACATACCACATGTGACATGTCACGTGTGTACTGTGTAGCATAAACAATATC

TAACTGAGTATTCCAAAAACATTTGCAAAAGAAAAGTGTTCAGAAAAGCCTGTTGAGTTATTTACCAGAT

CTTTTTATAATTTTGCTAGAGCCAGCTTTTTTGTGTATAAAACTTTGCATAACTCACACATACCACATGT

GACATGTCACGTGTGAACTGTGTAGCATAAACATAATATCTAACTGAGTATTCCAAAAACATTTGTAAAA

GAAAAGTGTTCAAAAAAGCCTGTCGAGTTGTTTACCAGATCTTTTTATCAAAATATTTTATTGGTAGTGG

ATCATACTCGTTACTTAACTATATATTTATTTTTTATTTGACTGAAAACCCATTCCAGTAGTACTTTTTT

TCCACTCAAGAAAAGTATGAATTTGATGTTAAAAAAAAAAAAGTATTAATTTTTAAAACAAAATTTCTTA

CATATTGGTTGTTTAATCATTAACTTCCAAACAAAATTGCGGTGCAGCTAAGGCACTGAACCAAGCGAGG

TTAGAAGCAAACGTGAAAGTAGACGGAGAAACCAGCTTCAAGAATAAATTGCCAAGCCCTTTCGCGGTGT

TTTCCGGCATATTCCTCCTCCTCTCCTTCTTAAAATTTGTATACCCACCTCTTCGATGGCTAGCTGTCGT

GGGCGTCGCTACTGGTATTTATCCGATTCTTGCAAAATCCGTCGCTTCTATAAGAAGGCTTAGGGTCGAC

ATCAACATCCTAGTCATTATCACAGGTAATACCCACTTTTCACTTTTTATTTAATATTATTATTTTTATC

CACATCACTCATATTGCGTGTAACTACTGTATAATGATTTGTTAGTTTACTATGTAGTATTAGTTGAGAA

AGAAAATTGTGGTTATAGTAAAACTATTCAGGCCCTATTAATAGACCTATAATGTTCTTGGAAACTTGCG

AGTCTTTTACGCTGAATTTACCCCTTTATATGGTACTTCAGATTAGCTTACCTATATACTACTGCTTTCC

TGCAACACCTACCACTCCACGAAACCTTTTAGAAAGTTATCCTTTACTTTTTTCTTAATATTTTTTTAAA

GTATTACATATGGGAAAAATATCAAAACACATATTTATTAATTAATAGATGCGCAATTATTACTTTATAG

AAATTCAATTCTAGGAATGTAGCAATTTGATATTTATGTTGTATATGTTAATTGTATATTTGAGTTATAA

GTTGTGGAACTACATAAAACTACTTTATATTTTCTTTTTATGTAAAGTACATTTGAGTAATAGCCTAATA

GGATATAGAAAAATATCAAAATGTCAATGTTTTTAAAACCGGACCAGAAGGCGAACCGGATAATCATCCG

GGTCATGGTTCAATTTGGTTCGACCGGGTTGAATTCGGTTCATAATAATTTATGTTTATTTATTTTTAAA

TATAGAACTTTTATTTTTCAAAGTTCCCAAGTGTAAACACATACATAGAATAATTATTGTGATTTTACAT

AATTCTCTTATGGAAATATAATAATTCTTTTTTAACATGTAGTTTAAAAAGATAAATCTTTTACGTACAC

ACAACATAGATATATAGATTTTATATATAACTATCGAGGCAACTAGGAAAATGGAAGTTTCATGATCGAG

AGTTGTGTGGTTCTTTGGGAAAACTTAATTTTTTTGGTTATTTTATACGAAAGTAAAGGATTCGTTTGAT

TCTTGCTCAGTTTATTATTATTATTTTTTAAAAAAAAGCTGCAGTTACGTCCCATAGAAGAAAAAAAGGT

TAACTCGTATTTGATTGGCTTATCTTCTACGACTCAAAATGGGAAAAACTCAAAAAAGCAAAGCAAACTT

TTTAGTTTTAAGTTTTAACTCGTGAAAAGAAAATTAAAAAGAGCAACAAATAATTGAAAGAACAAAAGCA

TCAAAAGTAAAGAAATTAATTCATAATTCATAGACTGATAACGGAGTTACTTTTAGTTGAAATTTCGGTT

TAGGACACCAAGCTTGTGAATCCATAATATAAAATATTTTTTTAAAATCTTGATCTTTTGTTCGTTTGTA

TGATGTAATAGTCACTTCAACAAAACTATAACTCACTAATATTCCAATTTCATCAAACAGTGGCTGCAAC

ACTTGCAATGCAAGATTACATGGAGGCTGCAGCAGTTGTCTTCTTATTCACCATAGCTGACTGGCTGGAA

ACAAGAGCTAGCTACAAGGTATGTTAACTAGTAATCATCATATATTGTGTTAATCAAACTACTATGGATT

ATCTGAAGTTGAAATTGTAATGGATTATTGATTATGGCAATTGCAATCCCAGGCGAGCTCGGTGATGCAG

TCTCTGATGAGCTTAGCTCCACAAAAGGCAGTCATAGCAGAGACTGGAGAAGAAGTTGAAGTAGATGAGG

TTGAGCTCAACACAATCATAGCAGTTAAAGCCGGTGAAACCATACCTATTGATGGAATTGTAGTCGATGG

AAACTGTGAAGTAGACGAGAAAACCTTAACTGGTGAAGCATTTCCTGTGCCTAAACAGAGAGATTCTACG

GTTTGGGCTGGAACTATTAATCTAAATGGTAATGTAACCCTCTTACACAAGCTTCAATCTTAGAAAAGTT

TCAAGCTTTAACCTTTTTGTTTTGGCAGGTTATATAAGTGTGAACACAACTGCTTTAGCTAGTGATTGTG

TGGTTGCAAAGATGGCTAAGCTCGTAGAAGAAGCTCAGAGCAGTAAAACCAAATCTCAGAGACTAATAGA

CAAATATTCTCAGTACTATACTCCAGGTTTGCAAAAAAACATAAACCATAACTTGTTTTCTTTATGTTCT

TGATTCTTGTAATTTGAGACCTCTCTGTTTTTTGTTTGTTTCAGCAATCATCATAATATCGGCTGGCTTT

GCAATTGTCCCGGCTATAATGAAAGTTCGCAACCTCAACCATTGGTTTCATTTAGCACTGGTTGTGTTAG

TCAGTGCTTGTCCCTGTGGTCTTATCCTCTCTACACCAGTAGCTACATTCTGTGCACTTACTAAAGCGGC

AACTTCAGGGCTTCTGATCAAAAGTGCTGATTATCTTGACACTCTTTCAAAGATCAAGATCGCTGCTTTT

GACAAAACCGGAACTATCACTAGAGGAGAGTTCATTGTCATAGAATTCAAGTCACTCTCTAGAGACATAA

GCCTAAGCAGCTTGCTTTACTGGTAATAAAACAATATCTTGTTCTAACCAAAAACTAGTTTGATGGGATA

ACGTATGAATGACAATTTCTTGTTTGGTTCTCAGGTATCAAGTGTTGAAAGCAAATCAAGTCATCCAATG

GCAGCAACGATTGTGGACTATGCTAAATCTGTTTCTGTTGAGCCTAGGAGTGAAGAGGTTGAGGATTATC

AGAACTTTCCTGGTGAAGGAATCTATGGGAAGATTGATGGGAACAATGTTTACATTGGGAACAAAAGGAT

TGCTTCACGAGCTGGTTGTTCAACAGGTAAAGCTTCAAACTTTGGCCAAGAAAAAACTCAATGGAATGGT

TTTGTTGAGTCCTTAATCATTTTGAAACTGTTCTTCCTTGACAGTTCCAGAGATTGATGTTGATACCAAA

GAAGGAAAGACTGTCGGATACGTCTATGTAGATGAAAGATTAGCTGGAGTTTTCAATCTTTCTGATGCTT

GTAGATCCGGAGTAGCTCAAGCAATGAAGGAACTCAAAGATCTTGGAATCAAAACCGCAATGCTAACAGG

AGATAATAAAGATTCAGCAATGCATGCTCAAGAACAGGTATGAGACTGAAAAAACCAAGAATTTTTCATT

ACTCTCCTAACGTTAAGAGATTATATTAAAACTTTGACATGTTCTTATATGGAACAGCTAGGGAATGCTT

TGGATGTTGTTCATGGAGAGCTTCTTCCAGAAGACAAATCCAAAATCATACAAGAGTTTAAGAAAGAAGG

ACCAACTTGTATGGTAGGAGATGGTGTGAATGATGCACCAGCTTTAGCTAATGCTGATATTGGTATCTCC

ATGGGATTTCTGGCTCTGCGCTCGCGACGCAGTCTGGTCATATCATTCTCATGTCAAATGATATCAGAAG

GATACCAAAGCGATAAAGCTAGCAAGAAGAGCTCAGCGGAAAGTTCTTGAAAACGTGTTCATCTCCATCA

CTTTGAAAGTAGGGATACTGGTTTTAGCATTTGCTGGTCATCCTTTGATTTGGGCTGCGGTGCTTACTGA

TGTAGGGACTTGCCTGATTGTGATTTTTAACAGTATGTTGCTTCTGCGAGAGAAGGATAAATCTAAGAAC

AAGAATTGTTACAGGGCTTCTACATCTGTGTTGAATGGTAAGAAACTTGAAGGCGATGATGAAGAAGGTC

TTGACTTAGAAGCAGGGTTGGTATCAAAGAGTCAATGCAACTCAGGATGTTGTGGTGATAAGAAAAGCCA

AGAGAAGGTGATGTTGATGAGACCAGCTAGTAAAACCAGTACTGACCATCTTCACTCTGGTTGTTGTGGT

GAAAAGATCAAGAGAGTGTAAAGCTTGTGAAAGATAGCTGTTGCGGTGAGAAAAGTAAGAAACCAGAGGG

AGATATGGCTTCACTGAGCTCATGCAAGAACTCTAACAATGACCTGAAAATGAAAGGTGGTTCAAGTTGT

TGTGCTAGTAAAAATGAGAAGCTGAAGGAAGTAGTAGTAGCAAAGAGCTGCTGTGGAGAGAAGGAGAAAG

CAGAGGGAAATGTTGAGATGCAGATTCTAAATTTGGAGAAAGGGTCGCAGAAAAAGGTTGGTGAAACCTG

CAAATCAAGCTGTTGTGGAGATAAAGAGAAGGCTAAGGAAACACGTTTGTTGCTTGCTAGTGAGGATCCA

TCTTATCTGGAGAAGGAGAATCTGAAAAGTGAAAGTGGTGATGATTGCAAATCTCTTTGTTGTGGAACTG

GTTTGAAGCAAGAAGGGTCTTCTAGTTTGGTCAATGTTGTGGTGGAGAGTGGTGAATCCGGGTCAAGCTG

TTGCAGCAAGGAGGGAGAGATAGTGAAAGTCTCTAGCCAAAGCTGTTGCACAAGTCCAAGTGATGTGGTG

TTATCTGACTTTCAAGCTAAGAAACTAGAGATTTGTTGCGAAGTGAAGAAGACTCCAGAGGAGGTTTGTG

GATCTAAATGTAAGGAAACAGAGAAGCCTCACCACGTTGGTAAAAGCTGTTGCAGGAGTTATGCAAAAGA

GTATTGCAGCCACAGGCATCACGACAACCATCATCACCACCATGTTGGGGCTGCTTGACGGAGATAGTGA

TTGATTACCTTTAAACTCTCGACCCATCCATCTATTTGCATAACCTTTCCTTCTTCAACCAATGTCGCCC

AGAACAAAATAAAAACTTATTTAGTGTTTCCAGCAAAGGTGTGATTCGTAAAGACAATGCTGTTGATCGT

TGTTTGTCTTTTATGTTTGCCAAAACCATAATGTATTTCTCCTTTTCTTGTTTTTATTCTCTTCTTGAAG

ATGCCCAGAAGAAGTTTGAACTTCGATCCTAGAGTCTTAAAATCAAATAGAACAAGCAGTTGAAACATAA

CTTAGCCTTGGAGTCTTTTTGTATGCTGTGTACTACATAAGCTTTCTTGACTGACACGTTTCTTGTCAAT

TCTAGGGCATTACTTTATTAGGACAGAGAAGGTGTTGCAGTTCGTGTCCTGGAGAGTTTAGGTGAAAAAA

AAATAAAGAGCAAAAACTGACTGCTCGCACATCCATGTAATCAAGAATCAGTAAAAATAAAAATTAATCA

AAGGGTGACACAGCTCATGATCTTATATGAATCACCAACCATACTCTTCTCACTATATAAACAAATGTGT

CATTTCTTGAAAACAATCTGAAATATTCCGCAATCGCTGAAAGCATAGCATTAGAGGCAAAACCCTAGCT

ATTCTTTTGTTCTCCGTCTTTATTTCTTATCTTTTATTTCGTCAAGCTTGTCGATGAGGTGAAAACCTTC

TTAAAATATATTATTCGCTTCTTCAAGTTTAATAAGACATATACCCTAAGTTCCACAAACTTTTGTATCT

CGAGATAAAACTTGTGACACATCTGATTCAACACGAAACCTAATGTTTTTAAGATCTACTAGGTAGGATA

CTTGCGCTTCGCCGCGGAAGACTTTTTTTGTATTTTGATATTTTATATTTTGATATTTTAATCCAGTTTT

CCTTATATTCCATCTGTTCCATATTAAAATGTCGTTTAAGATTTTTTCATACATATTAAGAAAATATTAA

AATTTTTTATTTTACTCATTATTACTCAAAATTAATCTTCTCAAGGAGAAATGTGTAAAATCTTTGATGA

ATTCTCTTAGAAGGAAAGGAAGAATCATGAGTTTGATCTCCATCAATTTCAGAGAAAACTGCTTCCTCTT

CCTCCTCCAGGTTGTTCTTCTTCTTCCTCCATTCTCTTATGCTCCGACGCGGAATCGGCTTAAGACTTTT

AAAGAAACACAGTCTCTAAACGCTCGATTTACGTTTTCCGGTTAAATGAAGGAAATCAATAAATGAAAAC

CGGATAAAATTAAACCAAAAAAATTGCTGACTGATATTATTTATAATATTTATATTAGATGTTTGTTTTA

AATAACGTGAAGTAGATTATCTTGAGAAGCACATGAGATTTTTCTGTGCTGATTAACTACAATATCTTTA

CAAAAAAAAAACAACAGCAATTCCTCGAATGACACACGTAAAAATTAACTCCAAATAATGTAACTTCATT

TTTACCCCAGAATTAATGGTTGATTCCACCAATTTCATTATAATAGCATTACCATTTAGGTCTCTGGTGA

TGTTTATGTTTGTATCACCATTCATTAGTTATATAAAATATATAATGCTAATGTTGGGTGTTGATAGTAT

GTCCACTATATAACTTCTTCGTGTTTCTCACTGTGTACATATCATGATAAGAAACTTGTCTCTAGACCTT

GTCCCCATTTTAATATTATACACTTATTCAAAATCTTTATATATATATATATATATGGTTTATTTATATA

CTATTTCAAAAAAACAATTGTTACAGTTAAATGTTTTAATTTTTAACATGTTGGTTCCAGGAATTCAAAG

TGACTAATTTGATGATCTATATATGTTGCTTGTAATTTGCGGTTTCAAGTTTTCAACAATGTCTGAAGTG

AGACAGAAGTGAGACAAAAGACTTTGAAAGATTTGATTGGAATATAAAATCTCGCTATGCTTAAGTATCA

ACTAGACAACTTATAACAGTTGAGACAATAATAGTAAACTATATGTTGGCATAGAAAATGGCGTTTTCTA

TATGAATTGGCTGGTTGATTTATATATTTACAATCTCTAGTTCAGTATTCAGATGTTGTCAAAAAATCGA

AAATAAGGTTAAATTGTGGTTTCACGGTATAAAAGATTAAATACCAAAGTGGGTATACATCCAAACTCCG

GAATATATAACTTACGAAAATTTCAATATGAATTACCAAAAACAGTTCTAAGAAATCTGAATACACGTCT

TAACTCTTAAGTAGTATGTTAGAATTTTAGATGTACTTAGTCGACATTTTTTCAGTTTTCGATGTGAATT

TAGGACTGGTTATCACTCATTAGGACAAAAGTGGATCTCACAGGTTACTACTTTGCATCAATTCTATCAT

ATAACTCAATGGTCATAATAGGCTTGGGCATTTTTACCCAGCTCGAAATACTAAACCGAATCTGACCCAT

AATAGATGGATCGAACCGAACCGGAACACGAATATTCGAATGGGTCCTAAATTCCTATACCGGAAAGAAT

AGGACTCGAACCAGAACTGAATGAGAACCAAAAGAGTACCCAAAATATTCAAAATATAATTATATACCAA

AAAATATTAGTTATATTTAGACTTAAAATAACTAAAATATGTAAAATTACAATTCTAAACTTAATATACT

ACTTAAATTTAGAAAAAATAACCAAAATATTCAACAAATCCAAAACCGACCTGAACCCGGACAGAACCGA

ATCGGATCTGACCCGAAAATAAAAAATATTTGAATGGTTTTAAAATTTCTAGAACGAAAAAAACTAAACA

AAAAACAACCCAAACCCGATCCGAAAAACAGAATGCCCGGGCCTTGGTCAAAAACAATCGTAGAACGATA

TGCTTTTCTTTGATAAAGATACGATTATGATAATATTTAACGAATTAAGTATACTATTAAGCTTGACGCC

TTGACCACCTCACTGATAATTTTGTTGCATTGCGCATTTGCACTTTCATAACCATTTTTACAACTTTTCT

CAAAATATTAGATATCGATAAATAAATTAAAGGTATACATTAGTAATTTTCTAAGTCGTATTTATGTTTA

TTTGAATGCATCGCGATAACATATCCAACAATATTTTTCTCGAGATTCGTGTATTAGTTTATACAATATT

TTTTTAAAAAAATTAGACCGATCAAGAGGCCTCAGCCACACCTAGCTAGGCTTCTAGGAATCCATTGGTT

ATAGTCATATAGAATTAACAATTTCTCAATTGTAAAATTATAATCTTGTTTATTCAGATACGTAAAGGTT

AATGAGTCATGTGACATTAATAATTGTCTACTCACATTATTTAGAAGATTCAACGACTCCAAACTATTCT

TGATAGTACAGTTGTTAAATAATTGGAGTACATGTTGGGTCTTTGGTACGACTCTTGCTTGCATTGAAAT

CGATCATAACCATAGATTAACGATTCATAAATGCGAGGGTGACATTTTTCCTTTAAGCCGCCAAACATTC

ACTTTTTATTACAATAAATTAAACTGAACGGTTTAGGTTGTTAAGGTGAAAGTGTTATAATTTCAGTTCA

GTTTTACAATAATGCAATTACCGAAATACTTGATTTTGAGATATTGACATGGATTTATACTATTAATTTA

AAGAAAGACAAAATTTAGATGAGGCACTTCACCCCTGCTTAGGCTTCACTACGACCACTAAAATCTTATA

ACGAAGTTTTAGAGATTTACTCCTTGCTTTATAAATATGGATTTCGAATCACATTTAGACAATCACTTAG

ATTGGTAATTTTTAACTGAACGGTTTGGTATCAATACATTTTCAAAATTATTAATCAGAAATACTAGTGT

TAAACGAAAATATTCATATTGATGGTTGATTGAGGAGGAGAGGTGGGCTCCAGCATAAAATAGCTGTGCA

TTTTAGGATGGGAATCCCGTTTTTTTCTGTACCAACTCAAATTTTCCAAACTTTTTGAAAATGAGAAGTA

GCAATTTTCTGTTGGATTTATTTATTTTATTTTAATTGGTAAGGAAAAAGTACATGATAATGAGAAAGAA

AGAAATACCTGAATTATTCGTTTAAAAATTAAATAAAACAAAATTATTCATATTTTCCTTAAATTTTTCG

AATATTTTATGTATTTACCCAAAATTTATCGTATTTTTCCCATAATTTTTTCAAAATCTCATCAATTTTT

CCGATTAATTAAGACTTTGGTCTTTGGTATGTCTAATTTTTAGTTCACAAACTTCTTATTTCCTTCTTTT

CCCGTTATTTTCCGTTCCCATTATCATAAAAGAATACTTCGGATAATATATTTTGATTCCCGAATTTTTC

ATTATCGTTCAGTTCCCGTCAATACCGGTCACAAAACCCAAAATGCAGATGGTTATGAACAAGTGTACTT

TCTAATTCTACCTATCCACTTGCGCCAAATCTAGTGAATATGTCAAAATTGGCAAAATGAATATAATAAA

CTATATGCCAAAAAAGGTTCATAATTTTTTTTTATCAGCATATACTGGTTCTCTTTGAATCGCATATCAA

TAAGAAGCATCTCGTTTATCTAATTACCAACGCTAATGAGAGACGACCATTCCCTCATGTTCTCTCATTC

ACCCGCAAAATAGTATTGAGTTCTCCGCCTATGATAATCAGTTTATTCACCAGCCGCAACTCATCATTTA

GTTGATGCGTACGCGACGATGATATGTAACACCTCCGCATCCTTTTTAATTCTCGCATGAATAAACTGGT

CCGTCGAATTAAGGACCTCTACATCCCCTATCCCATGTCTTCAAAATAACCACAATCCACCGCTCTGACT

CACTGCATCCACTCTGAAAGAACTTTCAAACCCCAAGCCTTGACAAATGCTTCCTGCTCTCTCTGCACCA

AAATGTGTTTCAAAAATAGCCAAAACATCCGTAAGCCACTTCTTCACCATATACCGAATCAATCTTCTGA

AATAGGGTTTATTCGCCTCCCGAAGTTCCATAAAATACAATTCATCATAATTTCATAGCTTAACCATAGA

AATATCGGGGCAACTTGTTATGCTCCAATCTCCTCCATCTGCTTCTCCGACGATGCGATGTGCGTAACCA

ACGTCCCAATCGAACTACGTATCCAATAAAACCTCGATGTCTGTTTCACCTAGATTTTGAGCCAAGATTC

CATTTTCCCGTACTTCACTTCCCTCCGTCACCACAAAACCCCCAGCCCGACCAAGGTCCCCCTGTTCAAC

TCTGAGGCGTTTACCATTTGCAGATAGAGCAGTCTTCCCCTTGGTTGGGCCAAAAACAAATCCGCTAGTG

GGCCTACTAGTTAAATTTTGTTTGGGCCGAGTAAGTTCCAAACTCTTCCCATTCCTGAACTTTTTTCCTT

TATGCGCAGCTCTAGTGTAATTTGGGCCTTGTCCCGATCCAGACCCAAAAACAATCCCCTTCCCGTGTTC

CTTACTCTTTCGGTGCTTAGTTCCTAAGACCGGATAGACATTTTCCTTATCTCCGTTGCAAACATTAATG

TTCATTGATTCCACTATCTTGATTGCGCCCGTACTGCCCTCTCGTCCATTAACTGTCGAGTCCCTATTCT

CTTCGGTTTCCATATTCTCATCGAGGTTTCCACACTTATTCCTCAACACAATATTCAATGAAACTCTTTC

CTCTCTTGAGACTCGCGTCTCCTCTTCCATCCCTTCTTGACCAACCACCTCCGTTGGCCGTCCCCTACCT

CGTCGAACCTGTGTGAACTCCTCCTCAACCGAAACAGTTTCCTTCCCATTATCCCTATCCTGCTGGACTT

GCATGTATGGACCAAGTGTCCATAAATTCCAAACTGAGAACAAATGTTGGTAAGACCTTCATAGGACAAG

AAATATCTCCCTCCGTTAATCAACACCGTCCCTTCAATGGTTTCTTTAGATTAACCTCAACGGATACCCT

TGCAAACTGTGCTATTTCAAAGTTTAAAGTTGTGACGTCAACCTTCACCGGTTTCCCAAGACCCTTTGCA

ATCCCCTTTATGATAGCCTTATGATAAAAGTTCACCGGAATGTTCGATAACCTAACCCAAACCGGCGTTG

TCTCAATCTTAGGATCAAACTGCGGAGACCACGCTTGCACCATAAGATAACTCCCAAACGCTTTATATGG

ACCTCCCGTCAATGCCGCCATGTACTCCTCTTCATTCTCAAAATGGATCATAAAGAATTGACGAGGTAGA

TCCATGACATACATCGCAATTCTAGGTTTCCACATCTCCCTTAACTTCTTATTCATCGCCGTGATTGACA

CAGTTCGACCTAACACTTTGACAATCATACATTTTTTCCACAAATCGTTCATGGCCGCCAACACTTCCTC

CCTAATCGTTATTACTGGTTCTCCATCCTCACCATTCGGAAATTCCAAACGAAGTCTTGCTTCTACAAAG

GCTCATCCACCACCGTCTCTGGTACCGGTCTCCCACCCATACTACTCCCCGTCATCCTCTGCGCCTATGA

GCTTGTTAAATCTGGTGGTCGCCCCTTCTCCCCAACGTCATCCATGTGGGCATTGATGACCCTGCCACTC

TCGACCCCTGTTTCCTCAATCGCCTCACTCACCGCACTCATTAGGGTTGTTTTTATAAGTTTACTAGTAT

GTGCCACTTAGCAATAGACATACTACTAGACTTATTTAAGTAGTAATGTCTCAATTCTAGGCAAGATTTA

TACTTTTGTTTAAAACAGATTATTTATGCTTAAACATGATTTCGTAGATTGGTAGACCTCGGTAGGGAAT

TTTGGCATTGATGAATTGATTGGGGATGACTCTAGTAGTCTCGGTAAACATCTAGAGCTTTCCATTAGTT

GCTAACATGTGGGTAATATGTACCAGTATCACATACTATATGATTTGTCACGTCTCGGACCATGATTGTT

TTCAACAGGGGTCCAAAATAGGTCAAGGGTCAAAGCGGGGAATCGAACTTGGGTCAGAGGTTTCAATTAG

CATATTTTACCAATTTTCCTAGTGAGTTTTGCTACAATTTCTGTTTTTATAAAATGAATAGGGTGTCACT

TAACATCGTATTTTTCTAAGTCACACCACTGTCTCGGACGCGGTACGGTTTGAATTAAACAAGTAAGATT

CATTTTTAAGATAAGTTACAAAGAAAATATCAAACATAATTAAAAATTTTGGGAAGCCAACAGATTAAAG

ATAATTAATGCTATTCCAATGTTGAGTCGCAAATTTAAGTTCTAATTAAGGAGAGAATTCACTTTTTATA

GAACTGCCGCAAATTTTTTTTTATCTTTCTACCTGATTATTCAAGCACCGAGCATAAGTTATGATCTTGT

GCAAACATGTTACTAATTTAATAATATGTATTAAAGATATAGATACATCTTAGAAGAAAAGCTAAGAGAC

TAGACGACAAGTGCATTGCGTTAAAAAAATGTGTAGTTTAATTTGAATTTTAGAAATAAACTAAGAAAAT

TGTACTAAAAACCAAATAAAGAAAGCAATTAGATGAGGAATCACACATGGATTCCATTTTGTGAATCCAT

TTTGTGACATTACACTATTGGTGTTTTCCACTAACATTTTACTATTTTAGTAACTTTGACTTCGTATCTC

TCACTCACGAGATTAAATCCCTCTTTGATCAAATTTTCTGCTCAATTATTTCTTTAGATAACTAGCAAGA

ATCTATATTATTAAAAGAGAAACAAAACCTGAAAGTTATAAGGATAACACTATATCCAAACAAGAAAAAA

AAAAAAAAAAAAAACTGACATAAACCGGGTTGAATCCTGTTAAAAGACCAAACAACCCATTATCTATACT

ATTAAAAGAGAAACAAAATCTGAAATTTACAAGTTTATCACTATAATCAAACAAGAAAAAACTCAGTCAC

AACCAAAAATACCCGACTAGATCCGACCCGGCAACAATGAGAAGACAAAAAGCCCAAAGCAGTAACAAAT

AAATATTTTTCGGATACAATTTTAAGAGGGGTTTTCTGGTCCGAAAATCTTCAAGAAATATAGTTGTTTG

GCAACGGAGATACTCGATCCGACCCGAACGCGGCGTAGAAACACGCGGATCTAACAAGAGGAGAGGTGAC

TTAAATTGAACCGACTAAATAAACCGAATATTGTTCCAAAAACAAAAGAACCGGACGCTTACCAAAGCCC

ATAAATAAGTCCAATTAACCGGATAGCTCCTAAGAATATGCAAAAAATTATCAAGAAAATTAGTTCTTTT

TTCGATATTTGAAACATAAATGTCATATATTTGAAACTTAAATACGATATCACAGCGTAAGAAAGATTTT

GCGTACCCTGATTTAGCATAATAAATACTTCAGCTTCGACGAATGAAAAATCTGCGTCATTTTCCATAAA

TATCGTTAATTAACTGTGCCAATCAAATACAAAAACAGAAATGTCATAAACATCAATAACAACTCGAATT

TCTTTCCACGCAAAATCTAAGAAGTTTTCCACAAATTTCGCTAATTAACTAAAACGAATATACTTAATAC

CTAATCTAAAGCATGTACTAAGCACACAAAAGTGTCGACATTCATGATAGTATTAAAACTGAATTTGAAA

ATCAGTTACCAAAAAGAAAAAATCAAAGATTCTTGTGAAAATATCCCTACCAAAAATATACCGTGTGATA

ATTAATATTTTTAAATCATATTGATTAAAAACCTAATGACCCATCCTATGAAACTATAAATACATCGCTC

ACAGGGTAATTGTTACATACCTTGAAATTGCTAATGATTTTTTCCTTCTGTCTAATTTCATGTTTACTAA

TCACGATTTGTTTTATATGTAGGGGAATTCATGAATGGAAAAGCAACCAAAAGCTCTTTCTTAAATCACC

AAGAAACTATGATACGATCAAAGGTTAATCCCCATCGATCGGGTCAAGAAAATAGATAGTTGCCCCTTAT

TGTTTCACATACCTTTATGATGGCAAGCCTTTCAAAATAGGTTGGGGAATCCAAGTTAAGCTTCTTTTGG

AAGCAATACACGTCCGCATCTGGACAAATGGAGCTAATCCTAGCAAAAGAAAATGTAAGTCAACGTAATA

TAATAGACTATTAGAGATTTTGTACACGTTTTCGATTCTGAAATCTTAATCTCGATATGTCTTGCAAGGT

ATGAAAACACAGGCTTATGAATGTCACCATACCTTTAATTCGATTACGCGTGATTTTCATCGAAGTTTAC

AGAACCAACTGATCCGACGGATTCACCAAATCGATTTCATATCAGAAGACTAGATATCAATTTACGATCT

TCGAGTCTACGCTCACGGTTGTGCGACTTTTCGTTTTTCGCAAGGATGGTAAACAAAAAGAGTGGATCCG

ACAAAAGGCCCACTTTTTAATGGATAAAAACGACGAGAAAATGGGCTTATCCGTTTTTAGACTCATACCC

GAAAAGAATTTAGTTCGGCCCAAATTTTGACCACTTAATTTATCACTTTTAATCCTACACTAAAATTAAC

ACTAAAATTAACACTGAATAATTGTATAATTCATCTCATATCATATAATATATTTTTTACCTAATGTATT

TTAACGTATCAAAAATTACAAAATCTCGTGCTTCTCAACAACATACATTTCTTCGTTATATATCAGAAAT

ATATTATTCTTTACATAATTACAATATAAATACTTTAGGGGGGTTTATTGGTAGATGAATTTGTAAGAAT

TCTTAAAATTTTCAGAAATCTTTGTTATTGATTTGTGAATTCTAACAATCTTATTAAAATCTGTTGTTAT

TGGTTTGGTGATTTATAAAGTCAATACAAAATCAGTTGTTATTCAAAAAGTTTGTGTTTTAATGATTTCA

TGAATCCATTAAAATCCTTGTTATTGGGACATGGATTTTAAACATTTTAACTCATAGAACAAGATTTCCA

AAATACTAGCTATAACCCTTAGATTTTCAAAATTCATTATAACAAAATATTTTGATTGATTTTATGAATA

TACAATCTCTCTCCAAATCTAACATAAACTCTTCATAAATTTAACAAATCTCTTAACTTTCAAAATTTAT

CAACTCTATAGAAATTCATCTCCCAATAACCCCCCTTAGTTTTCTTATAAAAAATTACAACATCAGGTCT

AGTCATGATTATAATAATTCCAATTCTTAGTATGCAATATTGCGAGGATCATGTGTCTAAACTAGCGACG

TATCGGACAAGTTTTATCCTCGCCCCATATTCAAATTGATAATGTTTTATAATCTCACTTTTCTTTTGTA

ACCATTTTATATAAAGTGTTAATAGATATATACCATATTTTTATCCCAAAAACTTAAGAGTATGTAGTTG

TTTTGATAAAACTCTAATTGATCATCTACTCCATAAAAAGCTAATTTCGAAATTTATAAAACAAAGTCAC

ATGCACAAACAACTTATCTTGTGATTAAGGATGTTTTTACTTATGACTCAGCTAGGTTCAAATCTCAAAA

ACATAGCCAATTCAAATTTTATGAAGTTCGGTACTACTGTAAATTGAGCCACCAATCGTTTTTAAAAAGG

AGTTCGATTAGACTAGTCTATAATCCATTATAGTGAAAATTGCTACACAAAATATCATACTTTTATATAG

TGCTAATGTAATCGATTTTAAAATAAACTTATAGTTTTATATTCTTGGAAATTACTGAAAACAAAAAGAA

ACTACAATTTTGATAGGAACTAGCCTGAAAATTCGGAAGGTAAAATAGGTAATTCGAAACAAAGATTAAA

ATATACCTACGAAATTAACATGGTAAAAAAAACTAAACCAAATAAAAAAAGTTTAACGTAAAAAGAAAAG

TTTTAATTACAAAAGAATCCAGACTAACCACAACTTTTGTAAGCATAATCCATTAATGTTAGGTTTAGAA

CGACTTTTAAAGATTTAGTAACTATTTTACAAATCATTTAGTTACGTACTTTCTTTAAACTTCTAAGAAC

CAAATTTGTGGTATATATTTGATAATATTTAGAGTTATTTTTTCTTTTAGACTTTTATGTAATTGAATTC

TTTTGGGGATTTCGGAAAAGCAAAATATACATTTCGGTACTGTTAGAAGGAGAAAATTACATCAATGAGG

AGGACTTTGTAACTATGGTACATTTTGCCTTAATAAAAATATATTGTTGCCCTTTTATTTGTTTCACCTT

TAGACAATTTAACTTTATATTTGGGAAAATCGCATTTTAAGCCGATAAATGCTAACATTTTAAACTTTTA

AAGTTTTTATTAGCACTTCAAACACTCAAACTATTTTTTTCATACTTTAAACGAAAGTAACAAATTTTTA

CGCGCCGCCGAAAAAATTAATGAAAAAAATTTAATATTCTAAATTCAAAGAATCAAAAATTAAACGAAAA

AGGCTTAATAACTTGAACTCAATCATACAAATTTTATTTACCCTATTTCGACTTATAAACATTCAATTAT

CTCTTTGAAACTCTGCATTGTATAAAAAAATTGTTAATTTTATGGTTTAAAATACGAGGATAAATAGTTT

GAGTGTTGTCAGTGGTCATTTTCATTGTTTTAAAATGCAATTTCTCCCTTTATATTTTACTATTTTTTCA

ATTTTTACTTTTTATCTCTCAACAAAGAGATATCTTCTCTCCCTCACGATAAATCCTTCTCTCTCTTCTT

TTCTTTCTCCACTTTATTCATCTCCACTTTCCTTCTCTCTTTGCTTCCTACAAATTCTGTCTCTCATTTT

CCACTAGGTTCTTCTTCCACCAATAAAGATTGGGACAAGGGTTGCGTAAAGACATTATTATGCCTTGAAT

AAGTGTTTGTGTATGCATGCCCTTTTTACAAAAAAAAAAAAAGAATAAGTGTTTGTGAACAAAATATCTT

GGATAGAATTTTAGAATATATCACGTAGATCGTGGACAATAGGTGTGTGAACATATATATATATATTCAG

ACATATAACAATAAAATATGTACACAAGCTTCGATTTTCACCTCTGATACAACAATTCATCCACATCTTT

ATTATGTCCAAACAAAAGTAATCCACGGCAATAAATCTACATAATTTGGCTCTCTTTGATTCTTCCACTC

TTACTTTCATCTTTTTATTTCCGTAATCACAAGCAACAATGTTTCATTTTCACTTCTCGTTATCATCATA

TATTACAATTTTTTACCCATGTTAACCCTATCAAAACACCAATTAGATACATGAAAACAATTCAATCCCA

CACCAAACCAGAAATCGTTTTAATTTATAGCTTCAGTTTCAGAAATTAATATACGCCAAAAAACGTTGGT

TACTGTGAAACACACACCAAACCAAAAATCACCATCACAGAAATAGCCAAATTTATCCACAATTGTATAA

GAAAAAAAAAAAAAAAAGTCTTTGCGTAAGGAGAGTTATAAAAGTAAATTTCTCGTACACAAGTGTCACC

AAGTGAACATACATTAGTCAATGTTAGTATTTTAATATTCTCTTTTAGCTATATATAAGGGACCCATCTT

TGTTGAAAGAAGATGAAGTTCACAAAAAAACTTTTGCCTTCTCTCTCTATCGAATACTGTTATCCCACTT

TCCTTCCCTTTTCCTTCCCTCTCTCTTTTAATATCTCACCTTTATATATAATATTTTATAACATTAATAA

TTTTTTTAATAAATCCTAATAGGGGTATGATATATAATTATATATGGTCAAAGAAGGTGGAAGAAGCGTG

AGGATTACTGTGGAGGAAAAAGAGACATTGGAGAAAGCAACGGTCAACATTACTGTCAGATGTCGAAGGA

GAGAAAGTGAGAGAGTGTGAGACTCTGAGAGAGAGAGAAGTCAAGAAGGAGAAGAAGACAAAAGCTAATT

TAAAGCTACGAATAATTTAAAGCTACGAAGACGAGACGGGACATATATTCACCCTCGCTTTTCACATATA

TTTTCGGTATTGCCACTCTCAAATTTTATTTTTTCCCTTTTTTCTTGTCTTTTTTGACCCGGCCCTGCTT

ATTTGGCTATATAAGCAACTACCTTATCTAGATATCTTCACCTCGCAATCTTCCTCTCTACGTTCCAAAA

CCTCTCTCACTCTCTGTCTTCACCTTTGTGGTAATACTTTAATCTCTGATCGAACCGCACCAAACAAGTC

CGGTCTTTCTTCTCGGCCTCGTCTTTTCTCCGGTATTCTTTCTCTTCTTAATTCACATAGATTTCATAAC

AAGTGATTTTTTCGTAATAATTAAAATCCGATCAAATTCACGATAGTGATATGATATATGCATATATGCA

TCCAACACGTTATATGCATCCCAGCATAAAAGTTTTGCTTTCTTATTTTTTTTTCCCTTAAAAGATTTGG

AAAATTAGCCATTAATCCCATAATAATCTCTTTTTGCGATGTGATTTGTTTTTTTCTGTTTTAGATTTCC

GTTTCACAGATTCGCCATTAATCCCATAATAATCTCGATTTGTTTTTTATTTTTAGATTTCCGTTTCACA

GATTCGCCATTAATCCCATAATATTCTCTTTTTATAATGCGATTTGTTTTTTCTTTTTAGATTTCCGTTT

CACAGATTCGTTAATCATAAAAAACTTTGATACAGAAATGGCGTTACAGAAGGAGGACAAGAACAAAGAA

GAAAATAAAATGACAAAGAAGAAGTGGCAGAAGAGTTACTTCGACGTTTTAGGAATCTGTTGTACATCGG

AGATTCCTCTGATCGAGAATATTCTCAAGTCTCTCGACGGCATTAAGGACTATACCATCATCGTTCCGTC

GAGAACCGTGATCGTTGTCCACGACAGTCTCCTCATCTCCCCGTTCCAAATTGGTAAAGCATTAGCTAAT

TACTTTCTTCGAATTTTTATTTTTACCTAATAAAAATAATTGAATCAAAAACCATAAAGTAATCTCACTT

AACACGTAAACAATCACTTTACTTTTCTTCTCTTTCTGTTTTCTTCAAAATTAATTAATGGTTTCGCGTC

CTCGTTTGATACGCAAAGCCTCAAATTAATTTTTTTTTGGGAACTAAAATTACTCTATCTATCAGATTTA

CCATAAAAGCTTACTTTGACTTTACAAAACATTTATTAGCAAAATTCGTTTATCACCAACCTATTCAAGA

TTTAAGGGAAAATAGTTATCCTCAAAACTAGGGAATTCAGATTTTTGAAGTTTTTAACGATTCTACTGAA

AAACAAAAGCCCTATTATTTGGGTTTCTTCTCGAGAAAAAATAGAATATTAACCAAGTCGCCTTGGCCTA

GTGGTAATGGACTCACGGCTGTGAGCACCGCCCCCGGGTTCGAGTCGCCTTGGCCACCTAGCCGCGCCTT

TAACAGGGATAACGCGTAACCGGATGCAAGGCCTCGTGGGATTAGTCTTTAACCGGGATGCCCACGGTTA

TCAAAAAAAAAAAAAAAAAAAAAAAAAAATAGAATATTGTTGTTATGGATTTTTTTTCATTTTTATTAAA

ATTAAAAGAAAATTCAAAAGTTATTTATAAATCAAGTTTTTTAAAGCTATTTTGATGGATTGTTTTAGGA

AAATTGATCTAACCAACAATTGTAATTTTTTTTTTTTTTGTGTGTGATAAAGTCTACTTTTTCAACATTA

AAAACTAGAAATTGAAATTTACGGCTTCTTTATACAATTTTGCTCGAGCCAGCATCTTTGTGTATAAAAC

TTTGCATAACTCATACATACCACATGTGACATGTCACGTGTGTACTGTGTAGCATAAACAATATCTAACT

GAGTATTCCAAAAACATTTGCAAAAGAAAAGTGTTCAAGAAAGCCTGTTGAGTTATTTACCAGATCTTTT

TTATAATTTTGCTAGAGCCAGCTTTTTTGTGTATAAAACTTTGCATAACTCACACATACCACATGTGACA

TGTCACGTGTGAACTGTGTAGCATAAACATAATATCTAACTGAGTATTCCAAAAACATTTGTAAAAGAAA

AGTGTTCAAAAAAGCCTGTCGAGTTGTTTACCAGATCTTTTTATCAAAATATTTTATTGGTAGTGGATCA

TACTCGTTACTTAACTATATATTTATTTTTTATTTGACTGAAAACCCATTCCAGTAGTACTTTTTTTCCA

CTCAAGAAAAGTATGAATTTGATGTTAAAAAAAAAAAGTATTAATTTTTAAAACAAAATTTCTTACATAT

TGGTTGTTTAATCATTAACTTCCAAACAAAATTGCGGTGCAGCTAAGGCACTGAACCAAGCGAGGTTAGA

AGCAAACGTGAAAGTAGACGGAGAAACCAGCTTCAAGAATAAATTGCCAAGCCCTTTCGCGGTGTTTTCC

GGCATATTCCTCCTCCTCTCCTTCTTAAAATTTGTATACCCACCTCTTCGATGGCTAGCTGTCGTGGGCG

TCGCTACTGGTATTTATCCGATTCTTGCAAAATCCGTCGCTTCTATAAGAAGGCTTAGGGTCGACATCAA

CATCCTAGTCATTATCACAGGTAATACCCACTTTTCACTTTTTATTTAATATTATTATTTTTATCCACAT

CACTCATATTGCGTGTAACTACTGTATAATGATTTGTTAGTTTACTATGTAGTATTAGTTGAGAAAGAAA

ATTGTGGTTATAGTAAAACTATTCAGGCCCTATTAATAGACCTATAATGTTCTTGGAAACTTGCGAGTCT

TTTACGCTGAATTTACCCCTTTATATGGTACTTCAGATTAGCTTACCTATATACTACTGCTTTCCTGCAA

CACCTACCACTCCACGAAACCTTTTAGAAAGTTATCCTTTACTTTTTTCTTAATATTTTTTTAAAGTATT

ACATATGGGAAAAATATCAAAACACATATTTATTAATTAATAGATGCGCAATTATTACTTTATAGAAATT

CAATTCTAGGAATGTAGCAATTTGATATTTATGTTGTATATGTTAATTGTATATTTGAGTTATAAGTTGT

GGAACTACATAAAACTACTTTATATTTTCTTTTTATGTAAAGTACATTTGAGTAATAGCCTAATAGGATA

TAGAAAAATATCAAAATGTCAATGTTTTTAAAACCGGACCAGAAGGCGAACCGGATAATCATCCGGGTCA

TGGTTCAATTTGGTTCGACCGGGTTGAATTCGGTTCATAATAATTTATGTTTATTTATTTTTAAATATAG

AACTTTTATTTTTCAAAGTTCCCAAGTGTAAACACATACATAGAATAATTATTGTGATTTTACATAATTC

TCTTATGGAAATATAATAATTCTTTTTTAACATGTAGTTTAAAAAGATAAATCTTTTACGTACACACAAC

ATAGATATATAGATTTTATATATAACTATCGAGGCAACTAGGAAAATGGAAGTTTCATGATCGAGAGTTG

TGTGGTTCTTTGGGAAAACTTAATTTTTTTGGTTATTTTATACGAAAGTAAAGGATTCGTTTGATTCTTG

CTCAGTTTATTATTATTATTTTTTTTAAAAAAGCTGCAGTTACGTCCCATAGAAGAAAAAAAGGTTAACT

CGTATTTGATTGGCTTATCTTCTACGACTCAAAATGGGAAAAACTCAAAAAAGCAAAGCAAACTTTTTAG

TTTTAAGTTTTAACTCGTGAAAAGAAAATTAAAAAGAGCAACAAATAATTGAAAGAACAAAAGCATCAAA

AGTAAAGAAATTAATTCATAATTCATAGACTGATAACGGAGTTACTTTTAGTTGAAATTTCGGTTTAGGA

CACCAAGCTTGTGAATCCATAATATAAAATATTTTTTTAAAATCTTGATCTTTTGTTCGTTTGTATGATG

TAATAGTCACTTCAACAAAACTATAACTCACTAATATTCCAATTTCATCAAACAGTGGCTGCAACACTTG

CAATGCAAGATTACATGGAGGCTGCAGCAGTTGTCTTCTTATTCACCATAGCTGACTGGCTGGAAACAAG

AGCTAGCTACAAGGTATGTTAACTAGTAATCATCATATATTGTGTTAATCAAACTACTATGGATTATCTG

AAGTTGAAATTGTAATGGATTATTGATTATGGCAATTGCAATCCCAGGCGAGCTCGGTGATGCAGTCTCT

GATGAGCTTAGCTCCACAAAAGGCAGTCATAGCAGAGACTGGAGAAGAAGTTGAAGTAGATGAGGTTGAG

CTCAACACAATCATAGCAGTTAAAGCCGGTGAAACCATACCTATTGATGGAATTGTAGTCGATGGAAACT

GTGAAGTAGACGAGAAAACCTTAACTGGTGAAGCATTTCCTGTGCCTAAACAGAGAGATTCTACGGTTTG

GGCTGGAACTATTAATCTAAATGGTAATGTAACCCTCTTACACAAGCTTCAATCTTAGAAAAGTTTCAAG

CTTTAACCTTTTTGTTTTGGCAGGTTATATAAGTGTGAACACAACTGCTTTAGCTAGTGATTGTGTGGTT

GCAAAGATGGCTAAGCTCGTAGAAGAAGCTCAGAGCAGTAAAACCAAATCTCAGAGACTAATAGACAAAT

ATTCTCAGTACTATACTCCAGGTTTGCAAAAAAACATAAACCATAACTTGTTTTCTTTATGTTCTTGATT

CTTGTAATTTGAGACCTCTCTGTTTTTTGTTTGTTTCAGCAATCATCATAATATCGGCTGGCTTTGCAAT

TGTCCCGGCTATAATGAAAGTTCGCAACCTCAACCATTGGTTTCATTTAGCACTGGTTGTGTTAGTCAGT

GCTTGTCCCTGTGGTCTTATCCTCTCTACACCAGTAGCTACATTCTGTGCACTTACTAAAGCGGCAACTT

CAGGGCTTCTGATCAAAAGTGCTGATTATCTTGACACTCTTTCAAAGATCAAGATCGCTGCTTTTGACAA

AACCGGAACTATCACTAGAGGAGAGTTCATTGTCATAGAATTCAAGTCACTCTCTAGAGACATAAGCCTA

AGCAGCTTGCTTTACTGGTAATAAAAACAATATCTTGTTCTAACCAAAAACTAGTTTGATGGGATAACGT

ATGAATGACAATTTCTTGTTTGGTTCTCAGGGTATCAAGTGTTGAAAGCAAATCAAGTCATCCAATGGCA

GCAACGATTGTGGACTATGCTAAATCTGTTTCTGTTGAGCCTAGGAGTGAAGAGGTTGAGGATTATCAGA

ACTTTCCTGGTGAAGGAATCTATGGGAAGATTGATGGGAACAATGTTTACATTGGGAACAAAAGGATTGC

TTCACGAGCTGGTTGTTCAACAGGTAAAGCTTCAAACTTTGGCCAAGAAAAAACTCAATGGAATGGTTTT

GTTGAGCCTTTGATCATTTTGAAACTGTTCTTTCTTGACAGTTCCAGAGATTGATGTTGATACCAAAAAA

GGAAAGACTGTCGGATACGTCTATGTAGGTGAAAGATTAGCTGGAGTTTTCAATCTTTCCGATGCTTGTA

GATCCGGAGTAGCTCAAGCAATGAAGGAACTCAAAGATCTTGGAATCAAAACCGCAATGCTAACAGGAGA

TAATAAAGATTCAGCAATGCATGCTCAAGAACAGGTATGAGACTGAAAAAACCAAGAATTTTTCATTACT

CTCCTAACGTTAAGAGATTATATTAAAACTTTGACATGTTCTTATATGGAACAGCTAGGGAATGCTTTGG

ATGTTGTTCATGGAGAGCTTCTTCCTGAAGACAAATCCAAAATCATACAAGAGTTTAAGAAAGAAGGACC

AACTTGTATGGTAGGAGATGGTGTGAATGATGCACCAGCTTTAGCTAATGCTGATATTGGTATCTCCATG

GGGATTTCTGGCTCTGCGCTCGCGACGCAGTCTGGTCATATCATTCTCATGTCAAATGATATCAGAAGGA

TACCAAAAGCGATAAAGCTAGCAAGAAGAGCTCAGCGGAAAGTTCTTGAAAACGTGTTCATCTCCATCAC

TTTGAAAGTAGGGATACTGGTTTTAGCATTTGCTGGTCATCCTTTGATTTGGGCTGCGGTGCTTACTGAT

GTAGGGACTTGCCTGATTGTGATTTTTAACAGTATGTTGCTTCTGCGAGAGAAGGATAAATCTAAGAACA

AGAAGTGTTACAGGGCTTCTACATCTGTGTTGAATGGTAAGAAACTTGAAGGCGATGATGAAGAAGGTCT

TGACTTAGAAGCAGGGTTGGTATCAAAGAGTCAATGCAACTCAGGATGTTGTGGTGATAAGAAAAGCCAA

GAGAAGGTGATGTTGATGAGACCAGCTAGTAAAACCAGTACTGACCATCTTCACTCTGGTTGTTGTGGTG

AAAAGAAGCAAGAGAGTGTAAAGCTTGTGAAAGATAGCTGTTGCGGTGAGAAAAGTAGGAAACAAGAGGG

AGATATGGCTTCACTGAGCTCATGCAAGAAGTCTGACAATGACCTGAAAATGAAAGGTGGTTCAAGTTGT

TGTGCTAGTAAAAATGAGAAGCTGAAGGAAGTAGCAGTAGCAAAGACCTGCTGTGAAGACAAGGAGAAAG

CAGAGGGAAATGTTGAGATGCAGATTCTTGATTTGGAGAAAGGGTCGCAGAAAAAGGTTGGTGAAACCTG

CAAATCAAGCTGTTGTGGAGATAAAGAGAAGGCTAAGGAAACACGTTTGTTGCTGGCTAGTGAAGATCCA

TCTTATCTGGAGAAGGAAGAAAGGCAAACTACTGAAGCTAACATTGTGACAGTGAAACAGAGCTGCCATG

AGAAGGCAAGTCTGGACATTGAAACTGGAGTTACTTGTGATCTCAAGTTGGTCTGCTGTGGAGACATAGA

AGTGGGAGAGCAATCTGATCTTGAGAAAGGCATGAAGTTAAAGGGTGAAGGACAATGCAAGTCTGACTGC

TGCGGTGATGAAATACCTCTAACTTCTGAGGAAGACAGTGTGGATTGCTCCTCCGGATGCTGCGGAAACA

AGGAGGAATTAACACAAATCTGTCATGAGAAGGCATGTCTGGACATTGTAAGTTGTGATTCCAAGTTGGT

TTGTTGTGGAGAAACAGAAGTGGAAGTGAGAGAGCAATGTGATCTCAAGAAGGGTCTGCAGATAAAGAAT

GAAGGACAATGCAAGTCTGTTTGTTGCGGTGATGAAAAGAAAACAGAGGAGATAACTGAAGAGACGGACA

ATCTGAAAAGTGAAAGTGGTGATGATTGCAAATCTCTTTGTTGTGGAACTGGTTTGAAGCAAGAAGGGTC

TTCTAGTTTGGTCAATGTTGTGGTGGAGAGTGGTGAATCCGGGTCAAGCTGTTGCAGCAAGGAGGGAGAG

ATAGTGAAAGTCTCTAGCCAAAGCTGTTGCACAAGTCCAAGTGATGTGGTGTTATCTGACTTTCAAGCTA

AGAAACTAGAGATTTGTTGCAAAGTGAAGAAGACTCCAGAGGAGGTTTGTGGATCTAAATGTAAGGCAAC

AGAGAAGCCTCACCACGTCGGTAAAAGCTGTTGCAGGAGTTATGCAAAAGAGTATTGCAGCCACAGGCAT

CACGACAACCATCATCACCACCATGTTGGGGCTGCTTGACGGAGATAGTGATTGATTACCTTTAAACTCT

CGACCCATCCATCTATTTGCATAACCTTTCCTTCTTCAACCAATGTCGCCCAGAAAAAAAATAAAAACTT

ATTTAGTGTTTCCAGCAAAGGTGTGATTCGTAAAGACAATGCTAGTGATCGTTGTTAGTCTTTTATGTTT

GCCAAAACCCTAATGTATTTCTCCTTTTCTTGTTTTTATTCGCTTCTTGAAGATGCCCAGAAGAAGTTTG

AACTTTGATCCTAGAGTCTTAAAATCAAATAGAACAAGCAGTTGAAACATAACTTCAACTTAGGCTTGGA

GTCTTTTTGTATGCGGTGTACTACATAAGCTTTCTTGACTGACACGTTTCTTGTCAATTCTAGGGCATTA

CTATATTAGGACAGAGAAGGTGTTGCAGTTCGTGTCCTGGAGAGTTTAGGTGAAAAAAAATAAAGAGCAA

AAACCGACTGTATGAAAAAACACTCGCACATCCATGTAATCAAGAATCAGTAAAAATAAAAATTAATCAA

AGGGTGTCACAACTCACTATATAAACAAAGGTCGTCACTCTTGAAAACAATCTGAAATATTCCGCAATCG

CTGAAAGCAGAGCATTAGAGGCAAAACCCTAGCTATTCTTTTGTTCTCGTCTTTGTTTCTTATCTTTCAT

CTCGTCAAGCTTGTCGATGAGGTGAAAATTTAAAATCTATTGTTCACTTCTTCAAGTTTAATAAGACATA

TATCCTAAGTTCCACAAACTTTTGTATCTCAAGATAAAACTCGTGACACATCTGATTCAACACGAAACCT

AATATTTTTAAAGATCTACTAGGTAGGATACTTGCGCTTCGCCGCGGAAGACTTTTTTTGTATCTTTCCC

GTGTTATAAATGATTAAATTATACATTTTATATTTTGATATTTTAATCCAATTTTCCTTATATTTCCTCT

GTTTCATATTAAAATGTCGTTTAAGATTTTTTCACACATATTAAGAAAATATTAAAATTTTCTGTTTTAC

TCATAATTAATCTTCTCAAGGAGAAATGTGTAAAATCTTAGACGAATTCTCGTAGAAGGAAAGGAAGAAT

CAAGATACCGATTAGAGTCCATCATCATAGAAGAAGATGAACAGTCATGAGTTTGATCTCCATCAATTTC

AGAGAAAACTGCTTCCTCTTTCTCCTCCATGTTGTTCTTCTTCTTCCTCCATTCTCTTCTGCTCCGACGC

GGAATCGGTTTAAGACTTTTGAAGAAACACAGTTTCTAAACGCTCGATTTACGTTTTCCGGTTAAATGTT

TGACTAGTAAATGAAAACAGGATAAAATTAAAAACCGAAAACCAGGATAAATGTTAAATGTTTTCAGGAT

AAATGTTTGACTAGTAAATCAATAAATGAAAACAGGATAAAATTAAACCGAAAAAATTGCTAACTAGTAT

TAGATGTTTGTTTTAAATAACGTGTCAGCTTCAAAAAGGAGATTACCGTGAGAAGCACATGAGATTTTTC

TGTGTTGATTAAGTACAATATCTTTACAAAGAAAAAAAAAAAATTCCTCCAATGACACATGTAAAAATTA

ACTCCAAATAATGCAACTTCATTTTTACCCCAGAATTAATGGTTGATTCCACCAATTTCATTATAATAGC

ATTACCATTTAGGTCTCTGGTGATGTGTATGTTTGTACCACCATTCATTAGTTATATAACATATATAATG

CTAATGTTGGGTGTTGATAGTATGTCCACTATATAACTTCTTCGTGTTTTTCACTGTGTACATATCATGA

TAAGAAACTTGTCTCTAGACCTTGTCCCCATTTTAATATTATACACTTATTCAAAATCTTTATATATATA

GATTTTGAAAGATTTGCTTGGAATAGAAAATATCGCTATGCTTAAGTATCAACTAGACAACTTGTAACAG

TTGAGAATCGTAAACTATATGTTGGCATAGAAAATGGCGTTTTCTATGTGAGTTGGCTGGTTGATTTATA

TATTTACAATCTCTAGTTCAGTATTCAGATGATGTCAAAAAAACGAAAATAAGGTTAAATTATGGTTTCA

CGGTAGAAAAGAGTTATTGGGAGATGAATAAGTCAAAAGAATTGTTAAATTTATAATGAGTTTTTGTTAG

ATTTGAAAAGAGTATGTAAATTTAGGAAATCAATCAAAATATTTTATTCTAAAGGATTTTGAAAATCTAA

GGGTTATTGATAGTATTTTGAAAGTTTTGTTTCATGAGTTAATTTGTTTAAAATCCATCTCCCAATAACA

AGGATTTTAATGGATTCATGAAATCATTAAAACACAAACTTTTTGAATAACAATGGATTCTGTATGGAAT

TTAAAAATCATCAAACCAATAACAATGGATTTTAGTAAGATTATCAGAATTCATAAACCAATAACAACGG

ATTCTTAAAATTTTAAAAATTCTTTGAAATCCATCTCCCAATAACCCCTAAATACCAAAGTGGCTATACG

TCCAAACTCCGGAGTATATAACTTACGAAAATTTCAATATGAATTACAAAAACAGTTCTAAGAAATCTGA

ATACACGTCTTAAGCAGTATGTTAGAATTTTAGATGTACTTAGCCGACATTTTTTCAGTTTTCGATGTGA

ATTTAGGACTGGTTATCGCTCATCAGGACTAGACATGACCAAAATCAAACCGTAACCGTATTTCGGACCG

TAACCGGACCGTTTTGTAACCGTAACCAGACCGTAACCATATTAAACAGTTAAAACCGTAACCGTTAACC

GTTAAAATATATTAGTTATGGTTACAAAAATTATTAACCGTAATCATTTTTTTAACCGTAACTATATTAA

AACCGTTGGTTAACCGCAAGGTTAAACCGTATAATTTTAACCGTAACCGTTTTAAAAGTAATTTAAATAT

AACATTAATTAGTTATATACAAATAATTTGACATAGAGAAGTTATGTTGTACAATCAACTTATCAAATCC

AAACCTAAATTATTTAGAAGAATATATAACTATAAATTTAATGTGAATGTGCAAAAATCAACTGATATTT

TTCTAACAAATAAAACCTTTTTTTTGCCGTAACTAATTTTTAATAATAATAATAATAATTTTATAATAAA

TTTAGGTTAAAAAAAAACTAGATCGTGCTAGATACAATCATACAACTAAATAAAATTATAATTATATAAA

TTACAATCTCATCATCCAACTATATATCATTATCTAGTATTCTAATTTATAAATATGTATGTATAATTAT

ATGATATTTATATATCTGAAATAATTATATTATACGTACATGAAATATATTTCTTTTCATTTTATATAAA

AATATTTAATTACAAATCATTAATTAAATATAACCGTGTTTAACCATAACCGTGTTAACCGTAACCGTTC

TAACCGTAACCGTTTTAACCGTATATTAAATGGTTAAGGTTAAGGTTAAAAAAAAATTATAACCGTAACC

GGAGGTTAAATAACCTTAACCGTGATAACCGTAACCGTGGTCATGCCTAATCAGGACAAAGGTGGATCTC

ACAGGTTACTACTTTGCATCAATTCTATCATATAACTCAAAGGTCAGAATAGGTTTGGGCATTTTTACCC

GATCCGAAATACTGAACCGAACTGAAGCGGGACACAAATATTCGAATGGGTCATAAATTCTTATACCTAA

AAGAATAGGACTTGAACCAAAACTAAACCGATAACCGAAAGAGTACCTAAAATATTCAAAATATAATTAT

ATACCCAAAAATATTAGTTATATTATATTTAGACTTAAAATAATTAAAATATGTAAAATTACAATTCTAA

ACTTAATATAATAGTTAAATTTAGAAAAAATAACCAAAATATTCAATAAATCCAAAAATCTTTAGTTATA

TTTGTTAATATTTAATATTTTGTTGTTAAAACAACATTTCATTTAGAATTAAAGTTTATCCAATTTTTTA

TTTTTATTTTATTTTTTATAATTAAAGTTTGGATACACCGAACCGATCCGAATCCGGGCAGAACCGAATC

GGACCCGACCCAAAAATAAAAAAATATTTTAATGGTTCTAAAATTTCTAGAACGAAAGAATTAAAAAAAA

CTGACCCGAATCCGATCCGAAAAACCGAATGCCCAGGCCTAGGTCAGAATATATCATGCCCTCGATCGTA

GAACGATATGCTTTTCTTTGATAAAGATACGATTATGATAATATTTAACGAATTAAGTATACTATTAAGC

TTGACGCCTTGACCACCTCACTGATAATTTTGTTGCATTGCGCATTTGCACTTTCATAACCATTTTTACA

ACTTTTCTCAAAATATTGGATATCGATAAATAAATTAAAGGTATACATTAGAAATATTCTAAGTCGTGTT

TATGTTTATTTGAATGCATATCGCGATAACATATCCAACAATATTTTTCTCAAGATTCGTGTATTAGTTT

ATACAATAATTTTTTTAAATAAATTAGACCGATCAGGAGGCCTCAGCCACACCTAGCTCGGCTTCTAGGA

ATCCATTGGTTATAGTCTTATAGAATTAACAATTTCTCATTGTAAAATTATAATCTGGTTTATTGAGATA

TGTAAAGGTTAATGGTCAGGAGGCATTAATAATTGTCTACTCACATTATTTAGAAGATTCAACGACTCCA

AACTATTCTTGATAGTACAGTTGTTAAATAATTGGAGTACATGTTGGTCTTTGGTACGACTCTTGCTTGC

ATTGAAATCGGTCATAGCCATAGATTAACGAGTCATAAATGCGAGGGTGACATTTTTCCTTTAAGCCGCC

AAACATTCACTTTTTATTACAGCCAATTAAACTGAACGGTTCTGGTGGTTAAGGTGAAAGTGTTATAATT

TCAGTTCAGTTTTACAATAACGCAATTACCGAAATACTTGATTTTGAGATATTGACATGGATTTATATTA

TTAATTTAAAGAAAGACAAAATTTAGATGGGACACTTCACCCCTGCTTAAGCTTCACTACGACCACTAAA

ATCTTATAACGAAGTTTTAGAGATTTACTACTTGCTTTATAAATATGGATTTCGAATCACATTTAGACAA

TCACTTAGATTGGTAATTTTTAACTGAACGGTTTGGTATCAGTACATTTTCAAAATTATTAATCAGAAAT

GCCTATGTTCAAGATTTCGCTAATCGCTAACCAGGCGGTTGGTCACCGATTAGCGATTTTTCAAAAATCG

GTGATAAATCGAGGATTAATCGGGGTAGAATTTTTACTATATTTTAATATATTTTAAAAATTATATATAT

AAAACATTAATACCAAAATCATAAACTCATACAAAAGATTATATTAAGCATTTTTATCAATTCACATATA

ATAACAAAGGTAAAAATAGTCTTGATCATATAGAGAACATACAAAAAAATAACTAAAGGATGGTAATTTA

ATAATTTTTCATCTTTATATTCCTCAATGATCAAATTGTCCATAAATGTGGACTAAAAATGGTAATTTCC

TAATTGTTCATCTTATTTTGGGTCCGTTTCATTTTTTTTTAACACATGGCAAAGCAAAATAAAGTAAATG

GGCTTCAATTTTTTAATGGCCCCAAAAAATTTCTGATTAATCGGTCAATTTTTTGCAATTAATTGGTCAA

ACCAGTCAAACCACGGTTGACCGGTTTGTGATAACGATTAGGGGAAATCGACTCGGTCAGCACCCGATTA

GCGATTAAACGGCCGATTAATCGTTAAATCGGCCGGTTTTTTGAACAGAGAGAAATGCTAATGTTAAACG

AAAATATTCATAGTGATGGTTGACTGACGAGCAGAGGTGGGCTCCAGCATAAAATAGCTGTGCATTTTAG

CTTTTGGGAAGAGAATGCCGTTCCTTACGGTACCGGTCTCAAATTTTCCAAATTTTGTGAAAATGAGAAG

TAGCAATTTCCTGTTTGGGCTTATTTCCTTTATTTTAGCTAGTAAGGAAAGGGTACATCATAGGTATGGG

AAATAAAATCCAAATTACCCGTTTAAAAATTATAAATAAAAATAAAAATTATTAATATTAATATTTTTAA

AAAAAATTTTGAATATTTCTCATATTTATCAAATTTCCCATATTTTTCAATAATTTCTCAAAATCTTTTC

AATTTTTCCGATTAAGTAAGATTTTGATATTTGAGTATGTAATTTTTAGTCCCAAACTTCTCATTTCCTT

CATTTTCCCCTTATTTTCCATGCCATTATCATAAAAGAATACTTCAGATAATATATTTTGATTCCCGAAT

TTTTCATTGTCGTCCGATTCCCGTCAATACCTGTCACAAAACCCAAAATGTACAGGTTATGAACAAATGT

ACTTTCTAATTCTACCTAATCTAGTGAATATGTCAAAATTGGAAAAATGAATATAATAAACTATATGCCA

AAAAAGGTTCATAATTTTTTTTATTAGCATAAACATAATTAGGATCTTAAAATTTTTTTATCTTTAGTAA

ATTAGCTCTTCTGGTTAAAGATATCAAAAGGAGTTGTAAATATATGATTATTTTTAGAAATGTGCTATTT

TGTATGTTTAGAAATGGTTTGTACTCTGAACATGCATACTTTCGTAAAAAGTAACATAATAACACATAAT

AAACAGTAAAAAGTAACTTAAACTATTTTGGACTATACGAGGTGGATCCATAACATACATCGCACCTCTA

GATTTCTACATCTCCCTTAACTTCTTACTCATCACCGTGATCGACACAGTTCGACATAACACTTTGACAA

TTATACATTTTTTCCACAAACCGTTCATGGCCACCAACACTTCCTCCCCAATCGTTATTACTGGTTCTTC

ATCCTCACCATTCGGAAATTCCAAAGGAAGTCTCGCTTCTACAGAGGCCTCATCCACCACCGTCTCCGGT

ACCGGTCTCCCACCCATACTACTCCCCGTCACCCTCTGCGCCCATGAGCCTGTTAAATCTGGTGGTCGCC

CCTTCTCCCCAACGTCCTCCATGTGGGCATCGATGACCCTGCCAATCTCGACCCCCGTTTTCTCAATCGC

CTCACTCGCCGCACTCATTAGGTGTTTTTTTAAGAATTTTACTAGTGTGTGCCACTTAGCAATAAACATA

CTACTAGACTTATTTAAGTAGTAATGTCACAATTCTAGGCAAGATTTATACTTTTGTTTGAAATAGCTTA

TTTATCTTTAAACATGATTTCGTAGATTGGTAGACCTCGGTACGGAATTTGGCATTGATAAATTGATTGG

GGATGACTCTAGTAGTCTCGGTAAACATCTAAAGCTTTCCATTAGTTGCTAAAATGTGGGTAATATGTAC

CAGTATCACATAATATATAATTTTTCACGTCTCGGACGAGGATTGTTTTCAATAGGGGTCAAAAATAGGT

GAAGGGTCAAAGCGGGGAATCGAACTTGTGGGTCAGAGGTTTCAATTAGCATATTTTACCAATTTTCCAA

GTGAATCTTATGGCATTTTAGCTACAATTTCTGGTTTTATAAAATGAATAGGGTGTCACTTAACATCGTA

TTCTTCTAAGTCGACGCCACTGTCTCGGACGCGGTACGGTTTGAATTAAACAAGGTTAATTTTTAAGATA

AGTTACAAAGAAAAGATCAAACATAATTAAAAAATTTGGGAAGCCAACAGATTAAAGATAATTAATGCTA

TTCCAATGTTGAGTCGCAAATTTAAGTTCTAATTAAGGAGAGAATTCACTTTTTATAGAACTGCCGCAAT

TTTTTTTTTATCTTTCTACCTGATTATTCAAGCACCGAGCATAAGTTATGATCTTGTGCAAACATGTTAC

TAATTTAATAATATGTATTAAAGATATAGATACATCTTAGAAGAAAAGCTAAGAGAGTAGACGACAAGTG

CATTGCGTAAAAAAAATGTGTAGTTTAATTAGAATTTTAGAAATAAACTAAGAAAATTGTACTAAAAACC

AAATAAAGAAAGCGATTAGATGAGGAATCACACATGGATTCCATTTTGTGACATTACACTATTGGTGTTT

TCCACTAACATTTTATTATTTTAGTAACTTTGACTTCGTATCTCTCACTCACGAGATTAAATCCCTCTTT

GATCAAATTTTCTGCTCAATTCTTTCTTTAGAGAACTAGCAAGAATCATGATTATAATAATTCCAATTCT

TAGTATGCAATATTGCGAGGATCATGTGTCTAAACTAGCGACGTATCGGACAAGTTTTATCCTCGCCCCA

TATTCAAACTGATAATGTTTTATAATCTCACTTTTCTTTTGTAACCATTTTATATAAAGTGTTAATAGAT

ATATACCATATTTTTATCCCAAAAACTTAGAATATGTAGTTGTTTTGATAAAACTCTAATTGATCATCTA

CTCCATAAAAAGCTAATTTCGAAATTTATAAAACAAAGTCACATGCACAAACAACTTATCTTGTGATTAA

GGATGTTTTTACTTATGACTCAGCTAGGTTCAAATCTCAAAAACATAGCCAATTCAAATTTTATGAAGTT

CCGTACTACTGTAAAATGAGCCATCAATCGTTTTTAAAAAGGAGTTAGATTAGACTAGTCTATAATCCAT

TATAGTGAAAACTGCTACACAAAATATCATACTTTTATATAGTGCTAATGTAATCGATTTTAAAATAAAC

TTACAGTTTTATATTCTTGGAAATTACTGAAAACAATAAGAAATTACATCTTGATAGGAACTAGGTTGAA

AATTCGGAAGGAATATAGGAATTCGAAACAAAGATTAAAATATCCTACGAAATTAACATAGTAAAAAAAA

AACTAAACCAAATAAAAAAAGTTTAACGTAAAAAGAAAAGTTTTAATTCAAAGAATCCAAACTAACCACA

ACTTTTGAAACATAATCCATAAATGTTAGGTTTAGAACGGCTTTTACAGATTTAGTAACTATTCTCAAAT

CATTTAGTACGTCTTTCTTAAACTTCTAAGAACCAAATTTGTGGTATATATTTGATATATTTGAGTTATC

TTTTTCGTTCGACTTTGATGTAATTGAATTTTTTGGGGGATTTCGGGAAAGCAAAATATACATTTCGGTA

CTGTTAGAACGAGAAAATTACATCAATAAGGACTTTTTGACTTATCTTATTACAGCAAGAAGGACAGTGT

AACTATGGTGCACTTTGCCTTAACAAAAATACATTGTTGCTCTTTTTATTTGTTTTACCTTTACACAATT

TAACTTTATATTTTACCAGTTACACAATTTATCTCTCTCCACGATAAATCCTTCTCTCTCTTCTTTTCTT

TCTCCACTTTATTCATCTCCACTTTCCTTATCTCTTTGCTTCCTACAAATTCTCTGTCTTTCTCATTTTC

CACTAGGTTCTTCTTCCACCAATAAAGATTGGGACAAGGGTTGCGTAAAGACATTATTATGCCTTGAATA

AGTGTTTGTGTATGCATGCCCTTTTTACAAAAAAAAAAAAAAGAATAAGTGTTTGTGAACAAAATATCTT

GGATAGAATTTTAGAATATGATTGACAAAAAAAAAAAAGAATTTTAGAATATATCACGTAGATCGTGGAC

AATATGCCATGGACAATATGGGTGTGTGAACATATATATATTCAGACATATAACAATAAAATATGTACAC

AAGCTTCGATTTTCACCTCTGATACACAATTCATCCACATCTTTATTATGTCCAAACAAAAGTAATCCAC

GGCAATTAATCTACATAATTTGGCTTTCTCTTTGATTCTTCCACTCTTACTTTCATCTTTTTATTTCCGT

AATCACAAGCAACAATGTTTCATTTTCACTTCTCGTTATCATCATATATTACAATTTTCTACCCATGTTA

ACCCTATTAAAACACCAATTGGATACATGAAAACAATTCAATCCCACACCAAACCAGAAATCGTTTTAAT

TTCTAGCTTCAGTTTCAGAAACCAATTTACGCCAAAAACGTTGTTTACTGTGAAACACACACCAAACCAA

AAATCACCATCACAAAAATAGCCAAATTCATCCATAATTGTATAAGTAACCGTACAAACGTATATAACAA

AGCGTACACATGTTTTCAATTGTCCAAAAACACATTTTACTACAATTAAACGAAGCTATCAGATCCATGT

CCTCTCCCAGAAAAGAAAATAAACACTCGTCAAGATAACCATATTATTTATGAATAAGTAAAATTCATGC

GATGCAATTTCCTCCAGCCGTATCAGTCTCTACGTGGTGCGGTCGCCGGAGTTAAATTAAAAGACACCAA

CCAAAATAATCTGATTTTTCACCCCTTTTTCATTAAAGGTAAAATGGAACTCTCAACACACTATAAGCAA

AAAAGAAAAAAAGTCCACCACTTTCACAATTTCCCTCTTATAAAAGTCACTCTTAGTGTAAATGACAAAT

GACTCCTGTTAGAACTACCTAAAAGCAGGTTATGGAATTCGTTGGTCAAATGAATTAAAAAAAAAAAAAC

AAATTATAAATTGTTATGAAAGAAAATATCTCGTACACAAGTGTCACCAAGTATAAGGGACCCATCTTTG

TTGAAAGAAGATGAAGTTAACAAAAAAACTTTTGCCTTCTCTCTCTCTCCATCGAATACTACTATTATCC

CACTTTCCTTCCCTCTCTCTTTCGAAAATGTTAAAGAACAAAAACCAATCATCTAACATTCTGACTTATC

TACAGGGGCGGCTTATTCGAGTGGGGTCAATAGATGCTCTGCACTAGGTGACGTAGAAAAACAAAATTTT

AGTAGAAAAAAAGTTCACTTAAGCCGGCTATATAAAGCAACTACCATTTCTAGATATCTTCACCTCACAA

TCTTCCTCTCTACGTTCTAAAACCTCTCTCACTCTCAGTCTTCACCTTTGTGGTAATACTTTAATCTGAT

CGAACCGCACCAAACAAGTCCGGTCTTTCTTCTCGGCCTCGTCTTTTCTCCGGTATTCTTTCTCTTCTTA

ATTCACATAGATTTCATAACAAGTGATTTCTTCGTAATAATTAATATCCGATCAAATTCACGATAGTGAT

ATCTCCAACACGTTATATGCATGATGCATCCCAGCATAAAAGTTTTGCTTTCTTAATTTTTTTTCCCTTA

AAAGATTGGAAATGGCTGCCATTAATCCCATAATAATCTCTTTTTGCGATGTGATTTATTTTTTTCTTTT

TAGATTTCCGTTTCACAGATTCGTTAATCATAAAAAACTTTGATACAGAAATGGCGTTACAGAAGGAGAT

CAAGAACAAAGAAGAAGATAAAAAGACAAAGAAGAAGTGGCAGAAGAGTTACTTCGACGTTTTGGGAATC

TGTTGTACATCGGAGATTCCTGTGATCGAGAATATTCTCAAGTCTCTCGACGGCGTTAAGGAATATACCG

TCATCGTTCCGTCGAGAACCGTGATCGTTGTCCACGACAGTCTCCTCATCTCCCCGTTCCAAATTGGTAA

GCATTAGCTAATCACTTTATTCGAATTTTATATCATTTTTATTTTTACTTAATAAAAATAATAGAATAAA

AAAGCATAAAGTAATCTCACTTAACACGTAAACAATCACTTTACTTTTCTTCTCTTTCTGTTTTCTTTAA

AATTAATTAATGGTTTCGCGTCCTCGTTTGATACGCAAAGCCTCAAACGTTACTTTTTGGGAACTAAAAT

TACTCTATCTATCAGATTTACCATAAAAGCTTACTTTGACTTTACAAAACATTTATTAGCAAAATTCGTT

TATCACCAACCTATTCAAGATTTAAGGGAAAATAGTTATCCTCAAAACTAGGGAATTCAGATTTTTGAAG

TTTTTACCGATTCTAATGAAAAACAAAGCCCTATTATTTGGGTTTCTTCTCGAGATAAAATAGAATATTG

TTGTTATGGATTCTTTTTTTCATTTTTATTAAAATTAAAAGAAAATCTAAAAGTTATTTATAAATCAAGT

TTTTTAAAGCTATGTTGGTGGATTGTTTTAGGAAATTTGATCTAACCAACAATTGTAATTTTTTTTTTTG

TGAGTGTGATAAAGTCTACTTTTCAACATTAAAAACTAGAAAATTGAAATTTACGGCTTCTTTATACAAT

TTTGCTAGAGCCAGCATTTTTGTGTATAAAACTTTGCATGACTCATACATACCACATGTGACATGTCACG

TGTGAACTGTGTAGCATAAACATAATATCTAACTGAGTATTCCAAAAACATTTGTAGAAGAAAGTGTTCA

AAAAAGCCTGTCGAGTTATTTACCAGATCTTTTTATCAAAATATTTTATTGGTAGTGGATCATACTCGTT

ACTTAACTATATTTTTATTTTTTATTTGACAGAAAACCTACTCCAGTAGTATTTTTTTTCCACTCAAGAA

AAGTATTAATTTGATGTTAAAAAAAAAGTATTAATTTTTAAAACAAATTTTCTTACATATTGGTTGTTTA

ATCATTAACTTCCGAACAAACAAAATTGTGGTGCAGCCAAGGCACTGAACCAAGCGAGGTTAGAAGCAAA

CGTGAAAGTAAACGGAGAAACCAGCTTCAAGAATAAATGGCCAAGCCCTTTCGCGGTGGTTTCCGGCATA

TTCCTCCTCCTCTCCTTCTTAAAATTTGTATACCCACCTCTTCGATGGCTAGCTGTCGTGGGCGTCGCTG

CTGGTATTTATCCGATTCTTGCAAAATCCGTCGCTTCTATAAGAAGGCTTAGGGTCGACATCAACATCCT

AATCATTATCACAGGTAATACCACTTTTCACTTTTTATTTAATATTATTATTTTTATCCACATCACTCAT

ATTGACGTGTAACTACTGTATAATGATTTGTTAGTTTATGGTTCTCCGTAATCAATATACTATGTAGTAT

TAGTTGAAAAATAAATTAGAAAGAAAATTGTGGTTATAGTACAACTATTCAGGCCCTATTAACTAATGTT

CTTGGAAACTTGCGAGTCTTTTACTCTGAATTTAGCAACACCTAGCACTCCACAAAACCTTTTAGAAAAG

TTTTCCTTTACTTTTTTCTTAATATTTTTTAAAAGTATTACATATGGGAAAAATATCAAAACACATATTT

ATTAATTAATAGATGCGCAATTATTACTTTATAGAAATTCAATATTAGGAATGTAGCAATTTGATATTTC

TGTTGTATATGTTAATTGTATATTTGACTTATAAGTTGTGGAACTACATAAAACTACTTTATATTTTCGT

TTTATGTAAAGTACATTTGAGTAATAGCCTAATAGGATATAGAAAAATATCAAAATGTTCTTTGGATAGG

TAAAGTAAACATGTGATTAAACTCGGAAAGTTGGTATGGAAGATAATGCAAAATATAAAGGTGTATTCAA

AATATGGAAAAAAAGATACGCCTTTCTCTTTTCTCCTTGCAAAAATCTACATTTTCGTTCATTTATTTGA

TGATACAGTAACGAAGGTGTAAAACATTACTTTTAGATGTGACATACATAGAGTAATTATTTTCATTTTA

CATAATTCTTTTATCGAAAATTACAATATTTCTTTTTAAAAATGTAGTATAAAAAGAGAAATCTTTTACG

TACACACAACGTAGATTTATAGATTTTATATAAAACGATCGAGGCAACTAGGAAAATAGAAATTTCATGA

TCGATCGAGAGTTGTGTGGTTCTTTGGGAAAACTTAATTATTTTTTGGTTATTTTATACGAAAGTAAAGG

ATTCGTTTGATTCTTGCTCAGTTTTTTTATATTTTTTATAAAAGCTGCAGTTACGTCCCATAGAAGGAAA

AAAGGTTAAGTGGTTTTTGATTGGCTTATCTTCTACGACTCAAAATGGGAAAAAAGCAAACTTTTTAGTT

TTAAGTTTTAACTCGTGAAAAGAAAATTAAAAAGAGCAACAAATAATTGAAAGAACAAAATCATCAAAAG

TAAAGAAATTAATTCATAATTCATTGACTGATAACGGAATTACTTTTAGTTGAAATTTCGGTTTAGGACA

CCCTGTTAACAAAGAAAAATAATAGAGACACCAAGCTTGTGAATCCATAATATAAAATATTTTTTGTAAT

ATAAAATATAGTCACTTCAACAAAACTATAACTCACTAATATTCCAATTTCATCAAACAGTGGCTGCAAC

ACTTGCAATGCAAGATTACATGGAGGCTGCAGCAGTTGTCTTCTTATTCACCATCGCTGACTGGCTGGAA

ACAAGAGCTAGCTACAAGGTATGTTAACTAGTAATGATCATATATTGTGTTAATCAAACAGCTATGGATT

ATCTTGCTTTATTTATAGATGATCTGAAGTTGAAATTGTAATGGATTATTGATTATGGCAATTGCAATCT

CAGGCCAACTCGGTGATGCAGTCTCTGATGAGCTTAGCTCCACAAAAGGCAGTCATAGCAGAGACTGGAG

AAGAAGTTGAAGTAGATGAGGTTCAGCTCAACACAATCATAGCAGTTAAAGCCGGTGAAACCATACCTAT

TGATGGAATTGTAGTCGATGGAAACTGTGAAGTAGACGAGAAAACCTTAACCGGTGAAGCATTTCCTGTG

CCTAAACAGAGAGATTCTACGGTTTTGGCTGGAACTATTAATCTAAATGGTAATGTAACCCTCTTACACA

AGCTTCAATCTTAGAAAAGTTTCAAGCTTTAACCTTTTTGTTTTCGCAGGTTATATAAGTGTGAACACAA

CTGCTTTAGCTAGTGATTGTGTGGTTGCAAAGATGGCTAAGCTCGTAGAAGAAGCTCAGAGCAGTAAAAC

CAAATCTCAGAGACTAATAGACAAATGTTCTCAGTACTATACTCCAGGTTTGCAAAAAAACATAAACCAT

AACTTGTTTTCTTTATGTTCTTGATTCTTGTAATTTGAGACCTCTCTGTTTTTTGTTTGTTTCAGCAATC

ATCATAATATCGGCTGGCTTTGCGATTGTCCCGGCTATAATGAAAGTTCGCAACCTCAACCATTGGTTTC

ATTTAGCACTGGTTGTGTTAGTCAGTGCTTGTCCCTGTGGTCTTATCCTCTCTACACCAGTAGCTACATT

CTGTGCACTTACTAAAGCGGCAACTTCAGGGCTTCTGATCAAAAGTGCTGATTATCTTGACACTCTTTCA

AAGATCAAGATCGCTGCTTTTGACAAAACCGGAACTATCACTAGAGGAGAGTTCATTGTCATAGAATTCA

AGTCACTCTCTAGAGACATAAGCCTACGCAGCTTGCTTTACTGGTAATAAAAACAATATCTTGTTCTAAC

CAAAAACTAGTTTGATGAGATAACTTATGAATGACAATTTCTTGTTTGGTTCTCAGGGTATCAAGTGTTG

AAAGCAAATCAAGTCATCCAATGGCAGCAACGATTGTGGACTATGCTAAATCTGTTTCTGTTGAGCCTAG

GAGTGAAGAGGTTGAGGATTATCAAAACTTTCCAGGTGAAGGAATCTATGGGAAGATTGATGGGAACAAT

GTTTACATTGGGAACAAAAGGATTGCTTCACGAGCTGGTTGTTCAACAGGTAAATCTTGGACTTTGGTAA

AATCAAACTCAATGGAATGTTTTTGAGGTTTTGTTGAGTCTTTGATCATTTTGAAACTGTTCTTTCTTGA

CAGTTCCAGAGATTGATGTTGATACCAAAAAAGGAAAGACTGTCGGATACGTCTATGTAGGTGAAAGATT

AGCTGGAGTTTTCAATCTTTCCGATGCTTGTAGATCCGGAGTAGCTCAAGCAATGAAGGAACTCAAAGAT

CTTGGAATCAAAACCGCAATGCTAACAGGAGATAATCAAGATTCAGCAATGCAAGCTCAAGAACAGGTAT

GAGGACTAAAAAAATCCCAGACATTTCCATTATACTCTCTTAATTGTATCGATTATATATTAAAACCTTG

TTTTATATGAAAACAGCTAGGGAATGCTTTGGATGTTGTTCATGGAGAGCTTCTTCCAGAAGACAAATCC

AAAATCATACAAGAGTTTAAGAAAGAAGGACCAACTTGTATGGTAGGAGATGGTGTGAATGATGCACCAG

CTTTAGCTAATGCTGATATTGGTATCTCCATGGGGATTTCTGGCTCTGCGCTCGCGACGCAGACTGGTCA

TATCATTCTTATGTCTAATGATATCAGAAGGATACCACAAGCGATAAAGCTAGCAAGAAGAGCTCAGCGG

AAAGTTCTTCAAAACGTGATCATCTCCATCACTTTGAAAGTAGGGATACTGGTTTTAGCATTTGCTGGTC

ATCCTTTGATTTGGGCTGCGGTGCTTACTGATGTAGGGACTTGCCTGATTGTGATTCTCAACAGTATGTT

GCTTCTGCGAGAGAAGGATAAATCTAAGATCAAGAAGTGTTACAGGAAGAAACTTGAAGGCGTCGATGAC

CAAGGCCTTGACTTAGAAGCAGGGTTGTTATCAAAGAGTCAATGCAACTCAGGATGTTGTGGTGATAAGA

AAAGCCAAGAGAAGGTGATGTTGATGAGACCAGCTAGTAAAACCAGTTCTGACCATCTTCACTCTGGTTG

TTGTGGTGAAAAGAAGCAAGAGAGTGTAAAGCTTGTGAAAGATAGCTGTTGCGGTGAGAAAAGTAGGAAA

CCAGTGGGAGACATGGCTTCACTGAGCTCATGCAAGAAGTCTAACAATGACCTGAAAATGAAAGGTGGTT

CAAGTTGTTGTGCTAGTAAAAATGAGAAGCTGAAGGAAGTAGTAGTAGCAAAGAGCTGCTGTGAAGAGAA

GGAGAAAGCAGAGGGAAATGTTGAGATGCAGATTCTAAATTTGGAGAAAGGGTCGCAGAAAAAGGTTGGT

GAAACCTGCAAATCAAGCTGTTGTGGAGATAAAGAGAAGGCTAAGGAAACACGTTTGGTGCTTGCTAGTG

AGGATCCATCTTATCTGGAGAAGGAAGAAAGGCAAACTACTGAAGCTAACATTGTGACAGTGAAACAGAG

CTGCCATGAGAAGGCAAGTCTGGACATTGAAAATGGAGTTACTTGTGATCTCAAGTTGGTCTGCTGTGGA

AACATAGAAGTGGGAGAGCAATCTGATCTTGAGAAAGGCATGAAGTTAAAGGGTGAAGGACAATGCAAGT

CTGACTGCTGCGGTGATGAAATACCTCTAGCTTCTGAGGAAGACAGTGTGGATTGCTCCTCCGGATGCTG

CGGAAACAAGGAGGAATTGACACAAATCTGTCATGAGAAGGCATGTCTGGACATTGTAAGTTGTGATTCC

AAGTTGGTTTGTTGTGGAGAAACAGAAGTGGAAGTGAGAGAGCAATGTGATCTCAAGAAGGGTCTGCAGA

TAAAGAATGAAGGACAATGCGAGTCTGTTTGTTGTGGTGATGAAAAGAAAACAGAGGAGATAACTCTGGT

TTCTGATGAAGAGACGGACAATCTGAAAAGTGAAAGTGGTGGCGATAGCAAAGCTCTTTGTTGTGGAACT

GGTTTGAAGCAAGAAGGGTCTTCTAGTTTGGTCAATGTGGTGGTGGAGAGCGGTGAATCCGGGTCAAGCT

GTTGCAGCAAGGAGGGAGAGATAGTGAAAGTCTCTAGCCAAAGCCGTTGCACAAGTCCAAGTGATGTGGT

GTTATCTGACTTGCAAGCTAAGAAACTAGAGATTTGTTGCAAAGTGAAGAAGACTCTTGAGGAGGTTCGT

GGATCTAAATGTAAGGAAACAGAGAAGCCTCACCACGTTGGTAAAAGCTGTTGCAGGAGTTATGCAAAAG

AGTATTGCAGCCACAGGCATCACCACCACCACCACCACCACCATGTCGGGGCTGCTTGACGACGGATTGA

TTAGCTTTAAATTCTCGACGCATCCATCTATTTGCATAACCTTTCCGTCTTCAACCAATGTCGCCGAGAA

AAAATAAAAACTTCTTTAGTGTTTCCAGCAAAGGTTAAAGGTTTATCAACTGTGTGAATCGTAAAGACAA

TGCTAGTGATCGTTGTTAGTCTTTTATGTTTGCCAAAACCCTAATGTATATTTCTTCTTTTCTTGTTTTT

ATTCTCTTCTTGAAGATGCCGAGAAGAAGTTTGAACTTCGATCCTAGAGTCTTAAAATCAAATAGAACAA

GCAGTTGAAACATAACTTCAACTAGGCCTGGGCATTCGGGTCTTCGGGTCGGTTCTTGTCGGGTCCGGTT

CTTTCGGGTTTAGAAATTTTTAGACCCATATAGGAACCGATAGGATTTCGGTTCGGTTCGGGTCGGTTTT

AGGTCGGGTCCGGGTCGGTTCGGTTTAGAATTTTCAAAACCTGAAAAATAACCGGTTTTTGGCGGGTCTA

ATTCGGTTCGGTTCTTTTTCGGTTATTTCGTACTCATAATCTACTTTTTAACCGAAAATATTACCAAATA

ACCGAAAATTTTGCAAAATAACTGGGAAAAAAAACAAAATATCCAAACAAAAGTCAGATTAACGCTCCAT

TCCATCTCATCTCCGTCATCTACGTAAACCTCACATGTCACACAAAACACACATACTTAGTCTATGACTA

TATGACTCAATGCATAATTAGTAAATCATAATTCACAATTTCACAATGTATCAACTCTATGACTATTCGA

TTCTTTGTGTTTAGTGATTACCTTACTCTTAAGTTCCTTGAGACTTAAACTCTGGTTCAAACTCTAAACA

AAACACAAAGAAAAAAACATATCATAAATTAGAAAGATGAGTATCGAATTTCATACCCGTGATTGATTTC

GAGACGTCTCTGATGGTGGAGACGATTCACCTCGACGATTTCGGATTTCCAGTGTTTTTTTTTCTTGATT

GAACTGGGAATTTATTAAGTATTGAGGATTTGGGAGTCGGGAAAGTAGATCGGGGAGTGGGAGAGACGAC

ATAAGCTATTGGCGAAGTTGGCGACAAAAAAAATTCTATCGGGGAGTGGGAGAGGAGAAAAGCTAATGGG

GTTTAGTTTTGGGCTGGGTATATGTGGGCTTGGGAAAACTTAGGAAGGGTCTTAGGGTTTTTGGTTTTGG

GCTATAGGTACCCTTATCGGATATCGGGTAATTACCCGGACCCGAACCGAAAACCGTGGGTCTGCGAAAA

AAGGACCCAATAGGGTAAAATCCAATTACCCATATCCGATCCGAACCGATTTTTCGGGTCGGTTCCGGGT

CGAGTCCCTGGGTCCGGTTAAAAATGCCCAGGCCTAACTTCAACTTAGGCTTGGATTCTTTTCATTCGAG

GAACTCTACCTGTACATGTGTCATTTATCTTTTGGATTTGAATATTTTTGTTTTTACTCACTGATCGATG

TTTACTTACGTTTATGCCTTTGATTATACTAGATTTAGACCCGCGCTACGCCGCGGTATTTTTTTCTTTT

AATTTGTTATATTTTTATTTAATTTTGGTCATCATTTTATTATTTAATATATGTGAAATAAAATAGTTTG

GGCATGTAGCCTGTTTTAATTATCTCTGTGTGAGCTGTCTATTGACTGTGTGAATCTTCTTTTTAGGGAC

GACGTACCGTCTAGAACATCTTCGAGAAGAGCTTGCTCCTCCTCCACCTCTTCTTCTTCTTCATCATCAT

CTTTTTTGTTTCTTAATTTTTTGAGTGATTTTTCGACTTTGTTCTGTTTCTTTGGTTGAACGATAATCGT

TTGCTTCTTACTCTCTTTTGCATAAGTTGATGGAAATTTTCGGAGACTGGTTAAATGGCGGCGCCGAAAA

TCAAAAACGAAGACTCGTCATCGAACGGGAATACTGCTTATGCTAAAATCACGCACAATTGAGATTTTAC

GAAAAGACTGAATGGGTCGGAAACAATAAATCAAATCCATTTTCTCCCGCAACTAATTCTCGTTTAAACT

CGACACAGAAAAGAAGAAGAGATTGGCCTCTGAATTCGCGTCAAAATTATTTATGCGGATAAAATCAATT

TTACCGGTTCGGTGGAATAAAAAAGAACAAATTAAACCAATTTCTATTTCACTTAAATCAAACCGGTTAA

AAAGCTGATGTGTATTAATGAAATGCTGACTGATATTATTTGAAATGTTCCTATTGGCCAGACGTTTTTG

CTGAGGTGTCAGCTCATACCTTACTCTTAAGTTCCTTGAGACTTAAACTCTGGTTCAAACTCTAAACAAA

ACACAAAGAAAAAAACATATCATAAATTAGAAAGATGAGTATCGAATTTCATACCCGTGATTGATTTCGA

GACGTCTCTGATGGTGGAGACGATTCACCTCGACGATTTCGGATTTCCAGTGTTTTTTTTTCTTGATTGA

ACTGGGAATTTATTAAGTATTGAGGATTTGGGAGTCGGGAAAGTAGATCGGGGAGTGGGAGAGACGACAT

AAGCTATTGGCGAAGTTGGCGACAAAAAAAATTCTATCGGGGAGTGGGAGAGGAGAAAAGCTAATGGGGT

TTAGTTTTGGGCTGGGTATATGTGGGCTTGGGAAAACTTAGGAAGGGTCTTAGGGTTTTTGGTTTTGGGC

TATAGGTACCCTTATCGGATATCGGGTAATTACCCGGACCCGAACCGAAAACCGTGGGTCTGCGAAAAAA

GGACCCAATAGGGTAAAATCCAATTACCCATATCCGATCCGAACCGATTTTTCGGGTCGGTTCCGGGTCG

AGTCCCTGGGTCCGGTTAAAAATGCCCAGGCCTAACTTCAACTTAGGCTTGGATTCTTTTCATTCGAGGA

ACTCTACCTGTACATGTGTCATTTATCTTTTGGATTTGAATATTTTTGTTTTTACTCACTGATCGATGTT

TACTTACGTTTATGCCTTTGATTATACTAGATTTAGACCCGCGCTACGCCGCGGTATTTTTTTCTTTTAA

TTTGTTATATTTTTATTTAATTTTGGTCATCATTTTATTATTTAATATATGTGAAATAAAATAGTTTGGG

CATGTAGCCTGTTTTAATTATCTCTGTGTGAGCTGTCTATTGACTGTGTGAATCTTCTTTTTAGGGACGA

CGTACCGTCTAGAACATCTTCGAGAAGAGCTTGCTCCTCCTCCACCTCTTCTTCTTCTTCATCATCATCT

TTTTTGTTTCTTAATTTTTTGAGTGATTTTTCGACTTTGTTCTGTTTCTTTGGTTGAACGATAATCGTTT

GCTTCTTACTCTCTTTTGCATAAGTTGATGGAAATTTTCGGAGACTGGTTAAATGGCGGCGCCGAAAATC

AAAAACGAAGACTCGTCATCGAACGGGAATACTGCTTATGCTAAAATCACGCACAATTGAGATTTTACGA

AAAGACTGAATGGGTCGGAAACAATAAATCAAATCCATTTTCTCCCGCAACTAATTCTCGTTTAAACTCG

ACACAGAAAAGAAGAAGAGATTGGCCTCTGAATTCGCGTCAAAATTATTTATGCGGATAAAATCAATTTT

ACCGGTTCGGTGGAATAAAAAAGAACAAATTAAACCAATTTCTATTTCACTTAAATCAAACCGGTTAAAA

AGCTGATGTGTATTAATGAAATGCTGACTGATATTATTTGAAATGTTCCTATTGGCCAGACGTTTTTGCT

GAGGTGTCAGCTCATCCCTTATTGAAAAAGCTGATGTGTCATCACCTGGATTGACAATGAGGGTTTTGTG

TATTGAATTATGTTTTCATCGATCACATCCGCTATTAAGAGGCTTTGCATCTTATTAATTTTCCACTGAC

CTAGTGACGACCGTCGTCAGTTCTCTTTGCTTTTGGCTTTTTGGTGGCGATTGTCGCTACTCGCTTTCGA

AGGTTGACTTGTTCGGCAGTAATTATTATTACAATTTTCTCCTAGGAGACATTGTCTCTGTTTACTAATG

ATCGTCAATTTTCTTTTTTTTTCCGTTTTTAGCAAGCTTTTCATTCTAAAGAAGACTTGCTGTGTACGTA

AGTGTAACGATGAACGTCACTTCTCGTTTTCGAAGGTAAGTGATATGGTCTGTCACTTATCTTTTTCCCG

AGGCTTTGGCTTAGTTTTTGGTGCGTGACCATCGTAGTTTTTACTTCTAGGTGGTGCGTGACCGAACCTA

ACATAAACGTCCCATGTTCGACATTTTAATCTAGTACAACTTTTCTAATGATATGTTCTCTTTTTAGTTT

AGCGTCGATCGCGGACATTATTTTAGGCTTAGTTCTAAGCAACCATAGTCATTTTAGGGTTAGTGCCTTT

AGTTTGACAATGACAGTCGTTGCAGTCATCTATACCAACGGCATCGGCCGTGGTCAGAGCCAGTGGTCAC

TGCTTGTCACTCTTGTAAGAGGTGTTTCCTCTTATTTGATTGGCATTGACCATGGACGTTGCACGTTAAT

TAAGGCCTTAATTTCCTTTGATTTGTTTAGTAGTGGCCGTTTTCACACTCGTTTTGAAGTCGTACCTCTT

AATTGGCTAAATGTAACTGTTAACACAGATTTTTGTGGCAGTCACAACTTGGTCTCTAGAGGTCATGCCT

CTACTTTGTTTTGTGGTGATCCTTGTCCTTTCTGTTTTTTTTTTAATGCAATGCTCATAAATCAGTTTTG

TTGTGAGTATAGTAGCTGTTTTATTTTTTTCCTGACCACTATGTATGTGACAGTAGCCAAGTCAATTTTA

AGAGCTATATTGTCTCTTAAATATGTTTTCGTTCACTTAAGACAATACTCCAAGTCAATTTTTAAGCCTT

TCTAGAAAACAAAGATGCCATTTCAAATCGATTTAGGCAAAATATATCATTAGGAAGCAAAAAGAGGAGA

TTCAAAATCTAAAGAACATTATCACTACATCTTCTCAAGATGGTGCGCGTATGGAGTATCCGAGTGGCTT

AGTGGACGATCTGAAGCAAAAACTAATTGATGTGGGTTCGCTAGTATTTTAGCATAATTTCATTATTTCT

CTGGTCCACCAAACTAAAGTAAAACTATCAGTATTAGGTCAATTTATCTTTTATTATAAATAATTTGTGT

GGATGTGAAACATTCTGGGGGGGGGCATTATCAGGACCATGATTATGAAGATGAAAATGATGGATATCAC

GAAGCTGATGTAGAGAAAAGGGATGAGAAATATGATGAAGTCCCAAATTAGGCTATATTAGTTGTAGGGT

TTATGTAATAGACTAATAGTGACTCTAGTTGGTTCGAAGTGTCTTGAAAATGGGAATTATAATTTAAAAT

ATCTCCAATGTTTACATTCAATCAACGGTCCAAAATTCCTAATCATAAGTTCAAAAGCTCACCTGAATTC

TGGTTTAAAAGTCAAGAAAATAAGAAGCACCATCTACGTTAAGAGGAAAAGGAGGAGCAAGAGAAAAAGG

CAAGAAAATAAGAAGCACCATCTACGTACTACTGGTTTTGGTTCTATAGAGTATAGAATAGTCTTATCTC

TTGTGCAAGCGTTTATCAAACAGCGATCACTTGAGGTACGCTACACAGAGTTTGACCATTACAACAAATG

AGAAAGAAGCTACAATTTGTAGACGTAATTTCTTCAAACAAGAATCAAAACGTGGTATCATATAAACGTG

TTATACTTAGAAATCCTTCAAACAAGAAATGCATTGTCTTAGACCAGACCTTTCTTCTACTGTTCTACAT

GCCACAGAGAGTTCACAATAACTAAACCAAACATTTCTATATATTTGTTACCATTATAAAGGCTACACTG

CAGACACACCTTAGAGTTCCGAGCTGCTGTAGGAGTAGAGCTTCTTCCAGGCCAACCAAATAGATTTTAA

AAACCCACATCCATTTGGATTAGTTAACCCATTCCGCAAAAAGATTTGTACGAATCCTAATACCCTCTTA

ATCTTTTGTTATCTTCATATTTTAGCATAATCACAGCAAACAAGCTATCACTACATATGGATCTTCTTTC

CTTTTGTTTGTTGTTTGTAAAAAAATGTAGGTCTCATAAAGAAAAGTTATGAGAAAATGGATGTTTGCTT

AAAAGACCGAGAAAAACATTGTGGAAACAAGGAAGTCGACATTTTTGTTAATTATCTTCTCAATCTTGTC

AATAATTTTTTTCAACATTTCATTTTGTTAGCCACTTCATTTGATCGAGACCCATGTCGTGAAAGTAGCA

GCTTTGAAACAGTAGGAGTTTGTTTTTGCGATAGAGGATCCTGATGCTGGTAAAATTGGGGGCTTTATGT

TCAATTTCAATCCAGGACGCTTACAGCGTGATATTCCAACTGGGTCATACTGCTTCACTGTTGACTGTTG

AGTATGGATTTTGAATTTAGGTCTCGAAACTGTAGTAATATGTTTCTTTTTGCGATAGAGGACGGATGAT

CTTTCTGTTGGTAAAGTTTGTGGATTTTAGGCTCAGTTTCAATCCAGGATGCTTACAATATGATCTAATT

GGTACTTACTGCTTCACCAATGAGTATAAATTTACTCAACAGATAGAGCTTGAAACTGTCTGCGATTAAG

TTTGTAAATAGTAAACACTCTCTGTAAATGTTTTGAGTTTATGAGTCTCACTTTTAGATTTAGTTGTTGA

AACCGTGTCGATATATATATGTAAAGATTGCAAAGGGCTAAATCCTTTGAAATTTATTTTCTTTGATTGA

TACAGGATGCTAGCAGTTTGATAAAACAATCTGATAAATTCTCTGAATACGGCGAGATTCTTCTCATGAA

AGTTTTTGGAGTTCTTGCTGTATGTTTTTGGAGGTCTAGTGCAATTCTCTGTCAAGCAGTTTTAGTTTTT

GGCCTCTTGATTTGGTTACATTACATATATCTTATGTTTGCTTTCTTCGCAAACACCGCAGGTTACATAC

ATGTATTATACCGGAAGATTAGAAGTCTTCAACGAGAATTTTCCTGGTGTGAGTCACTAAGAGCTTTTCT

CGTTCCGTTAAAGTTCTTATCAGTTGAGATATTGATAGTAACATTTCCTATTCTACAATCTTGCAGGCTG

ACACAAAGCTATCATATGCCTTACAACATTGCAACCCCAGGAGAGACCGGAATATAAGGTATGATTGTGG

TTTACTCTCTCATTTACCTTACTCTTGCATTGCGTTGCTTAATGAGCTTATGTTGGATCAGGATGATATT

GAAGTATCTGATACCAGTAAAACTTTCGTTAGGAATCATACCAAAAGATGAGCTCTTGCAAAATTACAGT

CTTAATGAGGTAACAACTTTCCTTTTGCTCTATAATATTCTCTTCAATCTACGTCAAAACTTATTTCGCA

TCTGTGAGTGAGATTAGTATCCCTCTGAATCAAACTTCTCTATGAGTCCAGTACATAAAGATTGTGCAAG

CTCTGAGAAAGGGTGATCTCAGACTTCTCCGCCATGCTCTTCAAGAACATGAAGATCGGTATGTTTTACT

TGTTAGTCTAGCGCAAGAGCATTGTAATTAGCGAGGATTGTAGCTGTGTTGAATGCTTTCGTCAGTCAAG

TCTCTCTATGATGTGTAGTTTTTTGGTGAATTGAATAAAGGAAGTTTATCGATCTGGATTGAATCACAAA

AGAGCTCCAAGTCTACCAGAGACTCATGAAGAAAATGTAAGAAGCTTTTATATAAGGGCACACAAGCCAT

TTATACATTGAACAGTCTCAGATTTACAGTACTGTTGCGTAACTCTTTTATCAATGTTTCAGTTATACCA

TCCAGAAGCTGAGTGATCCAGCGAGAGCTCACCAACTAAAACTTGAAGTGATTGCCAAAGCACTTGGATG

GCTAGAAATGGACATGCCATACCATTTGTCGCTGTTGATTTACAAAACAAATTTCTGTAAAACTCACAGT

TCTTCTGATACTATTTTTCTTGCAGGTGGAATGTATAATGACGATCCTGATATACAAGAACCTTGTGAAA

GGCTATTTAGCACACAAGAACAAAGTGGTTGTCCTAAGCAAGCAAAAGATCCTTTCCCTAAGGAAGCCCG

TTGGCTCATAGATGTTACCTTCACTTGTTAGCCTGCGAATTGCATCAAGAAACCATGTTTAAGCACATGC

GTAATCTCAGTATTTGTTTTGACAACTTACTTTCCTCTGGTTACTCTAGTGCATGAGCTTAAGAAAGTTA

ATATCGATCTAATATCCTCTGATCAGGTTCTTGAGGTCGGTGTATTCGAAAAGCTAGAGCTCAAAAAATT

GAAAGCTAAATCTCAAACTATAAATTTAGATGCAAAATGAGTTCATAATAAAGCATATGGTTCAAAATAA

GTTCAGAAATAAAGCATATGGTTCAAAATGAGTTCAGAAATAAAGCTTAGGGTTCAAACTGAAATTAAAA

AGGACCAAATCGCAAAGAAAGAAACACATGAACTTCATAGGTTTTTCTTTCATTAGCCTGATTCTTTAGG

TAACTCTAAAAGATGATGTTGCTTTTATAATAAAGCAACTAGATCTTGACCGTGCTTGAGAAGCACGGGA

TGTTGGAATTTTTTTAAAGATTTAAGTTGTTTGAGTTACACATTTATTGAGTGTTAATTTTAATTTAGGG

TTAACTTAATTATTGAATCAGTAATGAAATAAAAAGTCATCATCTTAATTAATTGATAAATTTTTAGGCC

ACAATAATTCTTATCGGATATGGGTCTAGAACGGAGAAACCCATTAGCATGTTTTCTACTTCAGATTTGC

ATTTGCCTTTTTTGCATCCATACCGTTGGTTTAACATCTTGGGCGAAAGGAAATGTCGCGCAACTTGGGG

TCATGTGAGAAATCGAATCTCAAATCGATTTGATATATACTCTCCTGATATGAAATGGATTTGCCGACAT

CAGCTGTTTGGGATTTGATATAATATTCACTAATTAATTAATTAAATAGATACTTTCATTTGGGATTTTT

ATTTGCCTATATCTCAGATTTAACTAATGGATACAAATTTTAAAGCTGAATAGTTCAAAAATATTTTAGG

ATCTTCGTAAAACTAAACTTTAAAAAAATTCGAATATACGATGGAATCTGAAAACATGAGAGCAACACCC

TAGAAGTGGGATGAAGGTACTTTAATTGTGGCTAGGAGCAAGTATGTGTATGTCAATGTGGGCCGTTGAG

TTGACTTGACCTTGTATAGATCGTTGATTAGATAACTTATATACGTACGACTTTTCGATTAACGGTTTGT

ATTAGGAAATAAGGATATGATTTTGATTTAATATTTGTGGAAGTTCGTCTATACGAGAACATATAAGAAT

AATTTGGAAATTGACGCAGTCGTGGGAAGTATATGGGTGGCACGATCTTTCATACCCTTTAACCTTTACG

TCTCTCTTGCTTTAAGTTATGTCGAGCTTTTTTTTTTTTTTTTTTCGAACTATTTTTCATATAAGAAATG

AAATTGTAGGGCATATCGCAAGTTGGTCAAGGTGTTCTACGCAACTTTTGTTTTTCCTCTCTCTATCACA

TAGTTTTAGTAAAAAAAATAGTAAACCAATATTATTATATTTTACAATCGAATAATATATCTATCATATA

TGGATGATCTGATTTATAAAACACAGAATTTTGACAAATGATATATATAAAACATAGTTGAATCTACGAA

CTGCAATAGTTTACTTTTCAAATCTGAATCTAGACCTAATCTTATGAATATATAATAACATCAATTTGTT

CACATATAAACCAATTTATAACTACCAAGATGGCAAGATATATTCCCAATAGTTTGCTATTCATATTTGA

ATGTAGACCTAATCTTCAAAAAAATAAAATAAAATTGTTTGGTCCAACAATCTTCAAATAAGTTATTAAT

TATACGATAAAACGATTATACGAATTCACGAGTTATACCAAAGACAGAAAAATAAGTAGAGGTCAATGAC

CACTTGTTTGAGAAAATGGGATAATAAATAATTAGAGCCTGTAAACAACCCAACAAAAATTATAATATTA

CAATTTGTCTGGGATATGCTTTGGAACATAACTCGAATACAACGTAAAAAAATTATTTTCGGAGTGAAAA

AACACATGTTAATTATTTTCTTACAGAGTATTGGATGATGTAGATCTACGTATGAATCTAAATTAAGTTT

GATATCCTATTATAAAATACTATCGTGCATCTAGAGAAAAGATTTGTAAGCTTACAAAACAGTTGTATGC

GCCTGTAAGTGAAAGAACGTGCATTCCTTTGACTCGACACCAAATTGACCAACCAACCAACCAATCGACG

TTGACTTCTCCAGAGATGAACCTAGAAACCGAACCAAACCAAATCGAGTCGAACTAACAAAAACACTTGT

CATTTTATCAACGTAAGTTTGTTACGAATTTCATATCAACCCAAATGATATGATAATTGCACTTAGACCA

TAAGAGAACCAAATCCAAATTCATAAAACATGGATTTTACACAATTAATTTTTCTCCAAAACCCAATTGA

ATCTGAACCGGATTCAGATTGAACCGAACAGAGTCACAATTCTTTTCCAAAGCCAAATAAACTGGACGAA

CCGAACCAATAAACGTACAGTTTTCAAACCTACGCGGTATATAGCGCGTGGTTACTCAATACAATTTCAA

GTAGCCAATTATAAAATTACACGTCAGTGATTTAGAAAATTCTTAATTATTGTAAAATAAAATAAAAAAT

TCTTATTAATCTCTCTCTACCTCAAAATTTTACTTGTTTATGTTTTTGAACCGAAGAGACAGACTTCTTC

TTCTCCGTCGTCGTCGTCTTCTTTCTTCTTCTTTTGTGTACAGACATACCTTCATTTTATAGCGAGAACG

ACAAAGAGAAACATAGAGAGAGAGAGAGAGATCTTAAGAAGAAGAAGAAGAAACGAAATCGCGGACGAAC

AATAGAAGAAATTGAAATTACCCATAGATATCGATACGTATACTCCGGGAGATTTATACACGTTTCTTCG

AGTGTGTAGCTCTCAGTTGTTCGTTCGCTGAGAGTTAAGGATTTTTCTCGGGGTTTTGTTTTTCGTTAGA

TCTTTGGGGTGGAAACAAACAAGCTCGTGTAGACGGAAACTCTCGCCGTCTGTGGGATCCGCCTCCGTTT

TGAAAAGGTTCTATTTTTCCTCGGATTTTTTATTATTAGGCTGTTACAGTGTTCAATTTTTCGATTCTGG

ATTTTGTGCGGACGATGAATTTAGCTTTAGAAGAGATCCGATTTGGTTTGCGGGATGTGAAAAAAACTCT

AATTGAATATTTCGATTTTCATTTGATCTCCTTCTATTCTTAGATTGAGAGAAGCATTTGTTTGACTTAG

ATGTTTGCTTTGAAGCAATGCGCGTGAATGATTGTGTTGTGTTGTGCATTTTGAATTGGCTATGAGAAGC

TGTGACGATGCTTTGACTTTTCTTTGTTTGGACTTTTTTGCTGTGCTAATTTGAAATCGTCAATTTATTT

TCTTGTCCATGTTTGATTCTTCCATCTTACTGTTGTTATGCTTATAAGCTTGCTGGTAGGAAACTTGAAA

AATGGTGAAATCGTGTTGTTCTGTTCAAGAAACGAACTTTAGAGAAACTATATTTTTCAAGAATCAAGGT

TGTTTAGTTGAGTTTCACTATTATGTTTCGATTAATTTGTATGGCTTTCTCTTCTCCATTTCTGTGTTGT

TTCCTTGTGTGTGTATGGATTTCTGATATTCAAATATGGTTTTTTTAATATGTAACTGTTAGGTGCGAAG

TGGAATTGTCTCGTTTGAGCTGAAGAAACTTGTTCCTACAAGTCTGAGGTGTTGCAGTATGCAATAAAGG

TTTTGTATGAGATGAAGGCAATTAAAGGGTGGCGTCTAGGCAGAACAAACTATATGCAGTCTTTGCCTGG

GGCTCGCCACCGTTCTCTTACAAGGAAACCAGTATGGATCATCGCGGTGGTTTCGCTGATAACAATGTTT

GTGATCGGTGCTTGCATGTTCCCTCATCACAGCAAAGCGGCTTGTTATATGTTTTCATCTAGAGGATGCA

AGGGGATTACTGACTGGCTTCCACCCTCGCCGAGAGAGTTTTCGGATGACGAGATTGCAGCTCGTGTAGT

CATTAGGGAGATATTGAGCTCCCCTCGTGTTATTAAAAAGACTTCTAAAATTGCATTCATGTTCTTAACT

CCTGGTACATTGCCTTTTGAAAAGCTATGGGACCTCTTTTTCCAGGTAGATTTCTTCTCTTCCCAACTTT

ATTTTCCCCGCCAAAATTGTGTCACAGGTTTCGTTTTGGTTAAGAAGATACTGATGATAGGAAGTACAAT

GAAGTCTTTTTAGTTTAGGTTGGGTTTTTATGCTGTTTGGTACTTCTTGGGAAATGTAGACTTTGTCTAG

AAAATCGTTTAAGATCTTAGATGCAGCATATTCTCAGGCGGCTAAATACTTAGATATGCCTTGTGAACAC

TTGCAATATGTGTAGTATCTCTGCGTTTGTTTGATTCTTTTAACTTATTTCTTTCACTGATGGTTTGTTG

TCCTTTTATCCCTCCTCACAGGGTCATGAGGGGAAGTTCTCTGTTTATATCCATGCATCGAAGGATACGC

CAGTTCACACCAGTCGTTACTTTCTTAACCGTGAAATTCGAAGTGATGAGGTCTGTATTTTTTCTGTGTC

TAATATCTTTAGATATCATGTCATTTCTCTCTATGGTCATGACTCATGAGTATAGTTGATGGAATATCAT

GAATTTTTCTTGCTTCTGTAATAAACATGAATCATATTCGTTTTCTGAAGGTGGTCTGGGGTAGGATATC

AATGATTGACGCTGAGAGACGTTTACTGACCAGTGCTCTTAGAGATCCTGAAAACCAGCAATTTGTTTTA

CTCTCTGATAGGTAATCTCCCAGAAACTTCATTTCTCCTCGCACTGATTATTCTGAGGTTTCCTTAACCA

ATTTGAGTGTTTCTTGTTTTTTGCAGTTGTGTGCCACTGCGAAGTTTTGAATACATGTACAACTATATGC

TGTACAGCAATGTCAGCTATGTTGACTGGTAAAGTCATTTCTCCAAGTTGATGAGATTTCAGAATTGTAA

TTACACATATGTTGCCAGATTTCTTCCCGGATTCTCGATACTAATATATGGTTTTCTCCTACTCTTCAGC

TTTATCGATCCTGGTCCACATGGAACCGGCAGGCATATGGATCACATGTTGCCGGAAATTCCAAAGGAAG

ATTTTCGAAAGGGTGCACAGGTAAAACCTATTTCACTGATTCAGTAATATGCCTGCCTCATACTCTGAAC

ATACATGCGAAATCACTAAAATGTCTTTATATCCGTTCTGTGTCAATTATCCTTCTCTATATCCGCGCAG

TGGTTCTCCATGAAGCGTCAGCATGCTGTAGTAACAATGGCAGACAGTCTTTACTACTCTAAATTCCGGG

ACTACTGTGGGGTGAGTATGACCTATACCCTCAAAAATAGATATTTCTTACTTTCGTTCTTGATTTGATG

GAGGTGTTATTATCTACTTTATATGGATATAATGTAACCACCCATTAATAGGAAGCCTCTGTTTTCTCTT

CAGCCAGGTATAGAGAGCAACAAGAACTGCATTGCGGATGAACACTACCTGCCAACATTCTTCCATGTGA

GAGAATCTGTTCTGACCCTTATAACTAAAAATTCATTTTTTGTACTTTCTTCATCCGCTAATATACTTTT

GGTGTGATAAATCTCTCAGATGCTTGATCCTGGTGGCATTGCTAACTGGACTGTGACATCTGTTGATTGG

TCTGAGAAACAGTGGCATCCAAAGACATACATGCCCGAAGATGTCACTCTCGAGTTACTCAAGAACCTCA

CGGTATTATCACCAAACCAAACTGTTATGGGCTCATAAATTTTCAACCTTTGACTATTCCCTTTCTAGAG

ATAATTAACTGATTGGTATACCTTTTGATCCATTTGCAGTCCATTGACGCAGTCTCACGCGGAACAACTG

AGGGAACGGTACACGCTTTAAAACCAATTCATCTCCCACTTGTAACCAGTTGTGAAATTACCAAAGACTA

TAATTATCTTTTTTGTTTTGATGTCTGCAAACACAGGGTGAAGAAACATGGACACATTGCATGTGGAACG

GAATCAAAAGACCCTGCTATCTCTTTGGAAGGAAATTCCACGCAGACACTCTCGATAAACTCATCGAACT

CTTTTCAAACTACACAAGCATCGCATAAACCTTCATGAGATGTTTTGACTGATCGATACGGAAGCAAAGG

AGGAGGTATTCTTTTGACTCGGATTCTTTGACCAACGTCCCATTACATTTCCAAGGAACCAAATTAAGAA

CTTGAGGAACTATCATGGAATGCCTAGAAGATGTTACAACTCGCTGTAAAGTCCCTTTAAGAGCCGAGAA

AATCGTTGTGATCTTGAGAAGCTTAGGAAGAAGTGTTTGGGATTTGTTTATCATTTGGTTCGGGATGTTG

GTACATTTTTTTCACTCATCTGTTTTTACTACATTTCATTGTTATTATAAAAAAAATTTCAGAGTGCTTT

TTAAATCTCTCTTTTTGTTTGGAACTGGTAATGAATCGTTAAAAAAAAGACAAAAGTTTATACAGTTTGA

AGCTAAGCATTTTGATCATTTCTCACTGAAACTCCACTTTCTGGAGTTCTTTTATCAGACTAATTATACA

AACTGTTATTAAGCAATTTTCTCTTCGTTAAGACAGAGTTCTCGAGAACTACATTCTTATTACTGCATAT

TACAAAAAAATAATATTACAACACCATTTGTTCATCGCCGATGTCCCAAAGCCACCACCGGAATCCTCAC

CTTATGCCCTCGGCTACCTTTCAACTTCACCTCTCCAAAGCTGTATACTCCAGAAACTGATCTCACGGTC

AGTGTGACCGAGAAACTTCTAGCAGCGCCAGGTCTGAGAGTCATTGCCGGTGGGTTTACTTCAATGGCGA

TAGATGGCTGCATCCTAGCTGTTATGGTGTATGTTTCTTCTACCTCTGCCACGTTTGTCACTCTCCTTGT

TATGGTTTGAGTCCCCACAAGGTGAGAAATGGCGATTGATGGTGCATTGAAGTTTGAAGGATGTTTCATG

TTGAAGTTGCAGGGCGTGTTTGTGTAGTTTCTTATCTCGTGTGCATCGATCCCCGGGGTTGTGCACAAGA

ATCCTAGATAGTCCTCATAACCTACACAGACAAAATCGACCAGTCTTATTGACCAGAGATCAAAAGATCT

TTGATATATCATCATTGGGGGAAAACAGAGCAACAACTACCTGCATCAAAGATAAGACCAGGATCTAGAG

CAGCACTTGGATTGACATGACCACTTCCGTAATCAAAAGGAGTGGCTTTAACAAGCGTTACAGTTTCTGT

GTCTGAATATTGCTGTGCCTGAAGAGGCCTTCCTGCTCTATCTATGACCGTTGAGGTTGTCATCAACGCT

GATTTGATAGCAGCTGGACTCCATTGAGGATGCTTCTGCTTCACCAGCGCTGCTATCCCAGCTATGTGTG

GTGCAGCCATGCTGGTTCCTGATATCAGTGCAAATCCTTCACCTACTCAAAAGAGCCCAAAGTAAGAAGA

AACTTTGACTTCTGTTCAAAAGAATCTCCACATGTTAGCTTCAGTACTTTGAGATTTGAATGCTTACCAA

CATAGTTAGGCTCGTCTGTTCCATTTGGACACCAAGCAGCCCATATTAGATAACCAGGTGCAAGAATATC

CGGTTTAAGAAGATCAGCATCTTGAAAGCTGAAATCTTTGGTATTAGGTCCTCTAGCTGAGAACAATGCC

ACTTGAGGTGCTGATTTGTGAAGTACAGGTTCTAAACCATCTCCGATGCTTCCCTCGGCTTTAAAGCTTT

TTACTCGTCCTGTCCAATCCCTTAATGTACTGACGTTATAGTAATCAATCAAATCCTAACGATGCCAAGA

AAAAAACAGCATTTCAGATTGAGAAATTAGGCTGAGAGTAAGTGAAAACATTGAAACTCCCATTACCATT

GACTTCGACACATCAGTAATCAGGATTCCTGGAATGGCAGAAGGAACAGGATCGAATTTTGTTCCTGGAG

AAACATTTTCAACGACAAGAACAAAACCAGCAGCTCCGAGATGCTTTGCGGTTTCAACAACTTTCTTGAT

GGAAGCTGTACCAACTACAAAGTTGAAAGAATATCCACAGAGAAGAATCTTCCCTTCAACCAGTTTCTTG

TTCAGAACGTCTGGTCTCTGACAATCAGATGGATTATACTTGGAAACAGATGAATCCAGTAGAACATCAT

TTGCAGACACCAAAGTGTACAACCGATGAGGCCGAGTTGATGCTGCAGAAATTACAGAACCATTCATCAA

AAACATCAAATAGAAACTTGGTATAGAATATAGATCATGCCAAATGTAATATAACATTTTTGTGTGTTCT

TACGTGATAATCCCATCCCAGCTAGCATTTTTCCGTTTCCTAGGGTCAGATGGTTTTTGTATCTGCGGTC

ATCGATTGCAGCTGCGACAGTAGTTATCCAGGGGCTATACGAAACCAAAGTCTTCGGAAATGGGCCTCCG

TTTCCAGCAGCTTGAGCAACGAACACGCCAGCTTTAACGGCTCCAAGAAGTGTGGCATCGAATGGATTCA

AGAACGTTGTCTTGGTAGTTGTTGGAGGACTGTTTGGACCAACAGAGAGGCTAAGAATATCAACTCCATC

ATGAACAGCCTGCTCATGAAAATTAAGGTAATATTCATGGAAAGTTAGCAGTTAGAACAACAATACAACA

AGCATATGCAGCTGGAGAAACTTGTGATCAGTTCTTAGTACTCTTACCTGATCAATCGCAGCAACTACAT

CAGCAACAAAGCCTCCAAAAAGCCGGTAAAGAGCCTTGTAAACAGCAATCCTGAAAGAAACTTAGCCTTT

TAGCTTACGAAGGAGAAGAAAACAAAACTGTCAACAAAACTGCCATTTTTCATTATCTACATACCTTGCC

CGCGGAGCCATCCCACTTGCTTTTCCGAATTCATAACCGTGCATTCTCAACGGAATACCGTTATTCCCAG

CTGCAATGGCTGCTGTATGACTGAAGAATAAAAGAATCAGAATTGCTTAGTACAGTGAAATAAGAGCACA

ATGAAAGGGTCCTATATCACCTCCTAGATCCTGAGGACAATATTAAATTATACAGACAAACCTTCCATGT

CCATCGCCATCCATTGGCGATGCATAGTCAATATTAGGGTTAAATGCCCCAGCCGCTTTAGCAGCTTCAG

CAAAATGTTGGGCTCCAACGATCTTCCTGTTGCAGAAGCTCTTCTTGGTGTGAGGATCTTCTTCACATTT

CCCTCTGTAATGAGGAAGAGGGCCATAAGGTAGTCTATGGTGAGAGGCAAAACTCGGGTGATGCGGATAG

ATCCCCGAGTCCACAAACCCGATAACAATGTCTTCTCCTGCTCTATCAAAACCACCACCAGTAGGCCAAA

CATCTGTTGGTAGTCCAAGAAACTCTGGTGTATGTGTGGTGAGTCTCCTCACTTTCCAATCTTTGTTAAC

AGATTTCACACCGGGGGCGCGACGTAGTGTCTCTGCCTACCAAATATAGTGGATTAGTGTAAGGGAACAC

GGATAAAGAAACTCGTAGAAAATGATCTTAAATCAGTTTTCCACCTGCTCAGGAGAAACATGAGCTGCAA

ATCCATTTATAACGTGTTTATAGCTGTAAAGCTTTTTGTATGATCCTTCCTCAAAGAGCATCCCAAGAAT

CATATCGTGCTTCCTCTCTAAGTGACGAGCATAAGATGTCACCAACTCACTGGAAAAAAGTAACAGATTG

TTGATCTAGCAAGCAATGTATAGACAGTTCTGAAGAGGAGAAACAATGTATCTAAGTACCTTGATGTATC

AATCTTCTCATCAGATTCAACAGCAGTTGCTTCAAAACCATTTTCTCCACCTTTGTAACTTATGATTGGA

TCTCCTTCCATAGTCACAATGTAGACCTCTGCTATCACGGTAACAAGAAG
