# Supplementary material for: Tandem Quadruplication of HMA4 in the Zinc (Zn) and Cadmium (Cd) Hyperaccumulator Noccaea caerulescens
Source: PLoS One. 2011 Mar 10;6(3):e17814. doi: 10.1371/journal.pone.0017814 (PMC3053397; doi:10.1371/journal.pone.0017814)
Supplement: Data S7 — Sequence alignment of overlapping regions of fosmids P6P46 and H2P47. (DOC) [file pone.0017814.s015.doc]

**Data S7 Sequence Alignment of overlapping regions of fosmids P6P46 and H2P47.**

CLUSTAL 2.0.12 multiple sequence alignment

H2P47 TCTCGTACACAAGTGTCACCAAGTGAACATACATTAGTCAATGTTAGTATTTTAATATTC 60

P6P46 TCTCGTACACAAGTGTCACCAAGTGAACATACATTAGTCAATGTTAGTATTTTAATATTC 60

************************************************************

H2P47 TCTTTTAGCTATATATAAGGGACCCATCTTTGTTGAAAGAAGATGAAGTTCACAAAAAAA 120

P6P46 TCTTT-AGCTATATATAAGG-ACCCATCTTTGTTGAAAGAAGATGAAGTTCACAAAAAAA 118

***** ************** ***************************************

H2P47 ACTTTTGCCTTCTCTCTCTATCGAATACTGTTATCCCACTTTCCTTCCCTTTTCCTTCCC 180

P6P46 -CTTTTGCCTTCTCTCTCTATCGAATACTGTTATCCCACTTTCCTTCCCTTTTCCTTCCC 177

***********************************************************

H2P47 TCTCTCTTTTAATATCTTCACCTTTATATATAATATTTTTATAACATTAATAATTTTTTT 240

P6P46 TCTCTCTTTTAATATCT-CACCTTTATATATAATATTTT-ATAACATTAATAATTTTT-- 233

***************** ********************* ******************

H2P47 TAATAAATCCTAATAGGGGTATGATATATAATTATATATGGTCAAAGAAGGTGGAAGAAG 300

P6P46 TAATAAATCCTAATAGGGGTATGATATATAATTATATATGGTCAAAGAAGGTGGAAGAAG 293

************************************************************

H2P47 CGTGAGGATTACTGTGGAGGAAAAAAGAGACATTTGGAGAAAGCAACGGTCAACATTACT 360

P6P46 CGTGAGGATTACTGTGGAGGAAAAA-GAGACATT-GGAGAAAGCAACGGTCAACATTACT 351

************************* ******** *************************

H2P47 GTCAGATGTCGAAGGAGAGAAAGTGAGAGAGTGTGAGACTCTGAGAGAGAGAGAAGTCAA 420

P6P46 GTCAGATGTCGAAGGAGAGAAAGTGAGAGAGTGTGAGACTCTGAGAGAGAGAGAAGTCAA 411

************************************************************

H2P47 GAAGGAGAAGAAGACAAAAGCTAATTTAAAGCTACGAATAATTTAAAGCTACGAAGACGA 480

P6P46 GAAGGAGAAGAAGACAAAAGCTAATTTAAAGCTACGAATAATTTAAAGCTACGAAGACGA 471

************************************************************

H2P47 GACGGGACATATATTCACCCTCGCTTTTCACATATATTTTCGGTATTGCCACTCTCAAAT 540

P6P46 GACGGGACATATATTCACCCTCGCTTTTCACATATATTTTCGGTATTGCCACTCTCAAAT 531

************************************************************

H2P47 TTTATTTTTTCCCTTTTTTCTTGTCTTTTTTGTACCCGGCCCTGCTTATTTGGCTATATA 600

P6P46 TTTATTTTTTCCCTTTTTTCTTGTCTTTTTTG-ACCCGGCCCTGCTTATTTGGCTATATA 590

******************************** ***************************

H2P47 AGCAACTACCTTATCTAGATATCTTCACCTCGCAATCTTCCTCTCTACGTTCCAAAACCT 660

P6P46 AGCAACTACCTTATCTAGATATCTTCACCTCGCAATCTTCCTCTCTACGTTCCAAAACCT 650

************************************************************

H2P47 CTCTCACTCTCTGTCTTCACCTTTGTGGTAATACTTTAATCTCTGATCGAACCGCACCAA 720

P6P46 CTCTCACTCTCTGTCTTCACCTTTGTGGTAATACTTTAATCTCTGATCGAACCGCACCAA 710

************************************************************

H2P47 ACCAGTCCGGTCTTTCTTCTCGGCCTCGTCTTTTCTCCGGTATTCTTTCTCTTCTTAATT 780

P6P46 ACCAGTCCGGTCTTTCTTCTCGGCCTCGTCTTTTCTCCGGTATTCTTTCTCTTCTTAATT 770

************************************************************

H2P47 CACATAGATTTCATAACAAGTGATTTTTTCGTAATAATTAAAATCCGATCAAATTCACGA 840

P6P46 CACATAGATTTCATAACAAGTGATTTTTTCGTAATAATTAAAATCCGATCAAATTCACGA 830

************************************************************

H2P47 TAGTGATATGATATATGCATATATGCATCCAACACGTTATATGCATCCCAGCATAACAGT 900

P6P46 TAGTGATATGATATATGCATATATGCATCCAACACGTTATATGCATCCCAGCATAACAGT 890

************************************************************

H2P47 TTTGCTTTTCTTTATTTTTTTTTCCCTTTAAAAGATTTGGAAAATTAGCCATTAATCCCA 960

P6P46 TTTGCTTTC--TTATTTTTTTT--CCCTTAAAAGATTTGGAAAATTAGCCATTAATCCCA 946

******** *********** ** *********************************

H2P47 TAATAATCTCTTTTTGCGATGTGTATTTTGTTTTTTTTCTTGTTTTAGTATTTCCGTTTC 1020

P6P46 TAATAATCTCTTTTTGCGATGTG--ATTTGTTTTTTT--CTGTTTTAG-ATTTCCGTTTC 1001

*********************** *********** ******** ***********

H2P47 ACAGATTCGCCATTAATCCCATAATAATCTCGATTTGTTTTTTTTATTTTTTAGTATTTC 1080

P6P46 ACAGATTCGCCATTAATCCCATAATAATCTCGATTTGTTTTTTA---TTTTTAG-ATTTC 1057

******************************************* ******* *****

H2P47 CGTTTCACAGATTCGCCATTAATCCCATAATATTCTCTTTTTATAATGCGATTTGTTTTT 1140

P6P46 CGTTTCACAGATTCGCCATTAATCCCATAATATTCTCTTTTTATAATGCGATTTGTTTTT 1117

************************************************************

H2P47 TTCTTTTTAGATTTCCGTTTCACAGATTCGTTAATCATAAAAAACTTTGATACAGAAATG 1200

P6P46 TTCTTTTTAGATTTCCGTTTCACAGATTCGTTAATCATAAAAAACTTTGATACAGAAATG 1177

************************************************************

H2P47 GCGTTACAGAAGGAGGACAAGAACAAAGAAGAAAATAAAATGACAAAGAAGAAGTGGCAG 1260

P6P46 GCGTTACAGAAGGAGGACAAGAACAAAGAAGAAAATAAAATGACAAAGAAGAAGTGGCAG 1237

************************************************************

H2P47 AAGAGTTACTTCGACGTTTTTAGGAATCTGTTGTACATCGGAGATTCCTCTGATCGAGAA 1320

P6P46 AAGAGTTACTTCGACGTTTT-AGGAATCTGTTGTACATCGGAGATTCCTCTGATCGAGAA 1296

******************** ***************************************

H2P47 TATTCTCAAGTCTCTCGACGGCATTAAGGACTATACCATCATCGTTCCGTCGAGAACCGT 1380

P6P46 TATTCTCAAGTCTCTCGACGGCATTAAGGACTATACCATCATCGTTCCGTCGAGAACCGT 1356

************************************************************

H2P47 GATCGTTGTCCACGACAGTCTCCTCATCTCCCCGTTCCAAATTGGTAAAGCATTAGCTAA 1440

P6P46 GATCGTTGTCCACGACAGTCTCCTCATCTCCCCGTTCCAAATTGGTAAAGCATTAGCTAA 1416

************************************************************

H2P47 TCACTTTCTTCGAATTTTTATTTTTACCTAATAAAAATAATTGAATCAAAAACCATAAAG 1500

P6P46 TCACTTTCTTCGAATTTTTATTTTTACCTAATAAAAATAATTGAATCAAAAACCATAAAG 1476

************************************************************

H2P47 TAATCTCACTTAACACGTAAACAATCACTTTACTTTTCTTCTCTTTCTGTTTTCTTCAAA 1560

P6P46 TAATCTCACTTAACACGTAAACAATCACTTTACTTTTCTTCTCTTTCTGTTTTCTTCAAA 1536

************************************************************

H2P47 ATTAATTAATGGTTTCGCGTCCTCGTTTGATACGCAAAGCCTCAAATTAATTTTTTTTTG 1620

P6P46 ATTAATTAATGGTTTCGCGTCCTCGTTTGATACGCAAAGCCTCAAATTAATTTTTTTTTG 1596

************************************************************

H2P47 GGAACTAAAATTACTCTATCTATCAGATTTACCATAAAAGCTTACTTTGACTTTACAAAA 1680

P6P46 GGAACTAAAATTACTCTATCTATCAGATTTACCATAAAAGCTTACTTTGACTTTACAAAA 1656

************************************************************

H2P47 CATTTATTAGCAAAATTCGTTTATCACCAACCTATTCAAGATTTAAGGGAAAATAGTTAT 1740

P6P46 CATTTATTAGCAAAATTCGTTTATCACCAACCTATTCAAGATTTAAGGGAAAATAGTTAT 1716

************************************************************

H2P47 CCTCAAAACTAGGGAATTCAGATTTTTGAAGTTTTTAACGATTCTACTGAAAAACAAAAG 1800

P6P46 CCTCAAAACTAGGGAATTCAGATTTTTGAAGTTTTTAACGATTCTACTGAAAAACAAAAG 1776

************************************************************

H2P47 CCCTATTATTTGGGTTTCTTCTCGAGAAAAAATAGAATATTGTTGTTATGGATTTTTTTT 1860

P6P46 CCCTATTATTTGGGTTTCTTCTCGAGAAAAAATAGAATATTGTTGTTATGGATTTTTTTT 1836

************************************************************

H2P47 CATTTTTATTAAAATTAAAAGAAAATTCAAAAGTTATTTATAAATCAAGTTTTTTAAAGC 1920

P6P46 CATTTTTATTAAAATTAAAAGAAAATTCAAAAGTTATTTATAAATCAAGTTTTTTAAAGC 1896

************************************************************

H2P47 TATTTTGATGGATTGTTTTAGGAAAATTGATCTAACCAACAATTGTAATTTTTTTTTTTT 1980

P6P46 TATTTTGATGGATTGTTTTAGGAAAATTGATCTAACCAACAATTGTAATTTTTTTTTTTT 1956

************************************************************

H2P47 GTGTGTGTGATAAAGTCTACTTTTTTCAACATTAAAAAACTAGAAATTGAAATTTACGGC 2040

P6P46 GTGTGTGTGATAAAGTCTACTTTTT-CAACATTAAAAA-CTAGAAATTGAAATTTACGGC 2014

************************* ************ *********************

H2P47 TTCTTTATACAATTTTGCTCGAGCCAGCATCTTTGTGTATAAAACTTTGCATAACTCATA 2100

P6P46 TTCTTTATACAATTTTGCTCGAGCCAGCATCTTTGTGTATAAAACTTTGCATAACTCATA 2074

************************************************************

H2P47 CATACCACATGTGACATGTCACGTGTGTACTGTGTAGCATAAACAATATCTAACTGAGTA 2160

P6P46 CATACCACATGTGACATGTCACGTGTGTACTGTGTAGCATAAACAATATCTAACTGAGTA 2134

************************************************************

H2P47 TTCCAAAAACATTTGCAAAAGAAAAGTGTTCAGAAAAGCCTGTTGAGTTATTTACCAGAT 2220

P6P46 TTCCAAAAACATTTGCAAAAGAAAAGTGTTCAGAAAAGCCTGTTGAGTTATTTACCAGAT 2194

************************************************************

H2P47 CTTTTTATAATTTTGCTAGAGCCAGCTTTTTTGTGTATAAAACTTTGCATAACTCACACA 2280

P6P46 CTTTTTATAATTTTGCTAGAGCCAGCTTTTTTGTGTATAAAACTTTGCATAACTCACACA 2254

************************************************************

H2P47 TACCACATGTGACATGTCACGTGTGAACTGTGTAGCATAAACATAATATCTAACTGAGTA 2340

P6P46 TACCACATGTGACATGTCACGTGTGAACTGTGTAGCATAAACATAATATCTAACTGAGTA 2314

************************************************************

H2P47 TTCCAAAAACATTTGTAAAAGAAAAGTGTTCAAAAAAGCCTGTCGAGTTGTTTACCAGAT 2400

P6P46 TTCCAAAAACATTTGTAAAAGAAAAGTGTTCAAAAAAGCCTGTCGAGTTGTTTACCAGAT 2374

************************************************************

H2P47 CTTTTTATACAAAATTATTTTATTGGTAGTGGATCATACTCGTTACTTAACTATATATTT 2460

P6P46 CTTTTTAT-CAAAAT-ATTTTATTGGTAGTGGATCATACTCGTTACTTAACTATATATTT 2432

******** ****** ********************************************

H2P47 TATTTTTTATTTGACTACGAAAACCCATTCCAGTAGTTCTTTTTTTCCACTCAAAGAAAA 2520

P6P46 -ATTTTTTATTTGACT--GAAAACCCATTCCAGTAGTACTTTTTTTCCACTCAA-GAAAA 2488

*************** ******************* **************** *****

H2P47 GTATAGTAATTTGATAGTTAAAAAAAAAAAGTATTAATTTTTAAAACAAAATTTCTTACA 2580

P6P46 GTATG--AATTTGATGTTAAAAAAAAAAAAGTATTAATTTTTAAAACAAAATTTCTTACA 2546

**** ******** * *****************************************

H2P47 TATTGGTTGTTTAATCATTAACTTCCAAACAAAATTGCGGTGCAGCTAAGGCACTGAACC 2640

P6P46 TATTGGTTGTTTAATCATTAACTTCCAAACAAAATTGCGGTGCAGCTAAGGCACTGAACC 2606

************************************************************

H2P47 AAGCGAGGTTAGAAGCAAACGTGAAAGTAGACGGAGAAACCAGCTTCAAGAATAAATTGC 2700

P6P46 AAGCGAGGTTAGAAGCAAACGTGAAAGTAGACGGAGAAACCAGCTTCAAGAATAAATTGC 2666

************************************************************

H2P47 CAAGCCCTTTCGCGGTGTTTTCCGGCATATTCCTCCTCCTCTCCTTCTTAAAATTTGTAT 2760

P6P46 CAAGCCCTTTCGCGGTGTTTTCCGGCATATTCCTCCTCCTCTCCTTCTTAAAATTTGTAT 2726

************************************************************

H2P47 ACCCACCTCTTCGATGGCTAGCTGTCGTGGGCGTCGCTACTGGTATTTATCCGATTCTTG 2820

P6P46 ACCCACCTCTTCGATGGCTAGCTGTCGTGGGCGTCGCTACTGGTATTTATCCGATTCTTG 2786

************************************************************

H2P47 CAAAATCCGTCGCTTCTATAAGAAGGCTTAGGGTCGACATCAACATCCTAGTCATTATCA 2880

P6P46 CAAAATCCGTCGCTTCTATAAGAAGGCTTAGGGTCGACATCAACATCCTAGTCATTATCA 2846

************************************************************

H2P47 CAGGTAATACCCACTTTTCACTTTTTATTTAATATTATTATTTTTATCCACATCACTCAT 2940

P6P46 CAGGTAATACCCACTTTTCACTTTTTATTTAATATTATTATTTTTATCCACATCACTCAT 2906

************************************************************

H2P47 ATTGCGTGTAACTACTGTATAATGATTTGTTAGTTTACTATGTAGTATTAGTTGAGAAAG 3000

P6P46 ATTGCGTGTAACTACTGTATAATGATTTGTTAGTTTACTATGTAGTATTAGTTGAGAAAG 2966

************************************************************

H2P47 AAAAATTGTGGTTATAGTAAAACTATTCAGGCCCTATTAATAGACCTATAATGTTCTTGG 3060

P6P46 AAAA-TTGTGGTTATAGTAAAACTATTCAGGCCCTATTAATAGACCTATAATGTTCTTGG 3025

**** *******************************************************

H2P47 AAACTTGCGAGTCTTTTACGCTGAATTTACCCCTTTATATGGTACTTCAGATTAGCTTAC 3120

P6P46 AAACTTGCGAGTCTTTTACGCTGAATTTACCCCTTTATATGGTACTTCAGATTAGCTTAC 3085

************************************************************

H2P47 CTATATACTACTGCTTTCCTGCAACACCTACCACTCCACGAAACCTTTTAGAAAGTTATC 3180

P6P46 CTATATACTACTGCTTTCCTGCAACACCTACCACTCCACGAAACCTTTTAGAAAGTTATC 3145

************************************************************

H2P47 CTTTACTTTTTTCTTAATATTTTTTTAAAGTATTACATATGGGAAAAATATCAAAACACA 3240

P6P46 CTTTACTTTTTTCTTAATATTTTTTTAAAGTATTACATATGGGAAAAATATCAAAACACA 3205

************************************************************

H2P47 TATTTATTAATTAATAGATGCGCAATTATTACTTTATAGAAATTCAATTCTAGGAATGTA 3300

P6P46 TATTTATTAATTAATAGATGCGCAATTATTACTTTATAGAAATTCAATTCTAGGAATGTA 3265

************************************************************

H2P47 GCAATTTGATATTTCTGTTGTATATGTTAATTGTATATTTGAGTTATAAGTTGTGGAACT 3360

P6P46 GCAATTTGATATTTATGTTGTATATGTTAATTGTATATTTGAGTTATAAGTTGTGGAACT 3325

************** *********************************************

H2P47 ACATAAAACTACTTTATATTTTCTTTTTATGTAAAGTACATTTGAGTAATAGCCTAATAG 3420

P6P46 ACATAAAACTACTTTATATTTTCTTTTTATGTAAAGTACATTTGAGTAATAGCCTAATAG 3385

************************************************************

H2P47 GATATAGAAAAATATCAAAATGTCAATGTTTTTAAAACCGGACCAGAAGGCGAACCGGAT 3480

P6P46 GATATAGAAAAATATCAAAATGTCAATGTTTTTAAAACCGGACCAGAAGGCGAACCGGAT 3445

************************************************************

H2P47 AATCATCCGGGTCATGGTTCAATTTGGTTCGACCGGGTTGAATTCGGTTCATAATAATTT 3540

P6P46 AATCATCCGGGTCATGGTTCAATTTGGTTCGACCGGGTTGAATTCGGTTCATAATAATTT 3505

************************************************************

H2P47 ATGTTTATTTATTTTTAAATATAGAACTTTTATTTTTCAAAGTTCCCAAGTGTAAACACA 3600

P6P46 ATGTTTATTTATTTTTAAATATAGAACTTTTATTTTTCAAAGTTCCCAAGTGTAAACACA 3565

************************************************************

H2P47 TACATAGAATAATTATTGTGATTTTACATAATTCTCTTATGGAAATATAATAATTCTTTT 3660

P6P46 TACATAGAATAATTATTGTGATTTTACATAATTCTCTTATGGAAATATAATAATTCTTTT 3625

************************************************************

H2P47 TTAACATGTAGTTTAAAAAGATAAATCTTTTACGTACACACAACATAGATATATAGATTT 3720

P6P46 TTAACATGTAGTTTAAAAAGATAAATCTTTTACGTACACACAACATAGATATATAGATTT 3685

************************************************************

H2P47 TATATATAACTATCGAGGCAACTAGGAAAATGGAAGTTTCATGATCGAGAGTTGTGTGGT 3780

P6P46 TATATATAACTATCGAGGCAACTAGGAAAATGGAAGTTTCATGATCGAGAGTTGTGTGGT 3745

************************************************************

H2P47 TCTTTGGGAAAACTTAATTTTTTTGGTTATTTTATACGAAAGTAAAGGATTCGTTTGATT 3840

P6P46 TCTTTGGGAAAACTTAATTTTTTTGGTTATTTTATACGAAAGTAAAGGATTCGTTTGATT 3805

************************************************************

H2P47 CTTGCTCAGTTTATTATTATTATTTTTTTTAAAAAAAGTACTGCAGTTACGTCCCATAGA 3900

P6P46 CTTGCTCAGTTTATTATTATTATTTTTT---AAAAAAAAGCTGCAGTTACGTCCCATAGA 3862

**************************** ****** ********************

H2P47 AGAAAAAAAGGTTAACTCGTATTTGATTGGCTTATCTTCTACGACTCAAAATGGGAAAAA 3960

P6P46 AGAAAAAAAGGTTAACTCGTATTTGATTGGCTTATCTTCTACGACTCAAAATGGGAAAAA 3922

************************************************************

H2P47 CTACAAAAAAGCAAAGCAAACTTTTTAGTTTTAAGTTTTAACTCGTGAAAAGAAAATTAA 4020

P6P46 CT-CAAAAAAGCAAAGCAAACTTTTTAGTTTTAAGTTTTAACTCGTGAAAAGAAAATTAA 3981

** *********************************************************

H2P47 AAAGAGCAACAAATAATTGAAAGAACAAAAGCATCAAAAGTAAAGAAATTAATTCATAAT 4080

P6P46 AAAGAGCAACAAATAATTGAAAGAACAAAAGCATCAAAAGTAAAGAAATTAATTCATAAT 4041

************************************************************

H2P47 TCATAGACTGATAACGGAGTTACTTTTAGTTGAAATTTCGGTTTAGGACACCAAGCTTGT 4140

P6P46 TCATAGACTGATAACGGAGTTACTTTTAGTTGAAATTTCGGTTTAGGACACCAAGCTTGT 4101

************************************************************

H2P47 GAATCCATAATATAAAATATTTTTTTAAAATCTTGATCTTTTGTTCGTTTGTATGATGTA 4200

P6P46 GAATCCATAATATAAAATATTTTTTTAAAATCTTGATCTTTTGTTCGTTTGTATGATGTA 4161

************************************************************

H2P47 ATAGTCACTTCAACAAAACTATAACTCACTAATATTCCAATTTCATCAAACAGTGGCTGC 4260

P6P46 ATAGTCACTTCAACAAAACTATAACTCACTAATATTCCAATTTCATCAAACAGTGGCTGC 4221

************************************************************

H2P47 AACACTTGCAATGCAAGATTACATGGAGGCTGCAGCAGTTGTCTTCTTATTCACCATAGC 4320

P6P46 AACACTTGCAATGCAAGATTACATGGAGGCTGCAGCAGTTGTCTTCTTATTCACCATAGC 4281

************************************************************

H2P47 TGACTGGCTGGAAACAAGAGCTAGCTACAAGGTATGTTAACTAGTAATCATCATATATTG 4380

P6P46 TGACTGGCTGGAAACAAGAGCTAGCTACAAGGTATGTTAACTAGTAATCATCATATATTG 4341

************************************************************

H2P47 TGTTAATCAAACTACTATGGATTATCTGAAGTTGAAATTGTAATGGATTATTGATTATGG 4440

P6P46 TGTTAATCAAACTACTATGGATTATCTGAAGTTGAAATTGTAATGGATTATTGATTATGG 4401

************************************************************

H2P47 CAATTGCAATCCCAGGCGAGCTCGGTGATGCAGTCTCTGATGAGCTTAGCTCCACAAAAG 4500

P6P46 CAATTGCAATCCCAGGCGAGCTCGGTGATGCAGTCTCTGATGAGCTTAGCTCCACAAAAG 4461

************************************************************

H2P47 GCAGTCATAGCAGAGACTGGAGAAGAAGTTGAAGTAGATGAGGTTGAGCTCAACACAATC 4560

P6P46 GCAGTCATAGCAGAGACTGGAGAAGAAGTTGAAGTAGATGAGGTTGAGCTCAACACAATC 4521

************************************************************

H2P47 ATAGCAGTTAAAGCCGGTGAAACCATACCTATTGATGGAATTGTAGTCGATGGAAACTGT 4620

P6P46 ATAGCAGTTAAAGCCGGTGAAACCATACCTATTGATGGAATTGTAGTCGATGGAAACTGT 4581

************************************************************

H2P47 GAAGTAGACGAGAAAACCTTAACTGGTGAAGCATTTCCTGTGCCTAAACAGAGAGATTCT 4680

P6P46 GAAGTAGACGAGAAAACCTTAACTGGTGAAGCATTTCCTGTGCCTAAACAGAGAGATTCT 4641

************************************************************

H2P47 ACGGTTTGGGCTGGAACTATTAATCTAAATGGTAATGTAACCCTCTTACACAAGCTTCAA 4740

P6P46 ACGGTTTGGGCTGGAACTATTAATCTAAATGGTAATGTAACCCTCTTACACAAGCTTCAA 4701

************************************************************

H2P47 TCTTAGAAAAGTTTCAAGCTTTAACCTTTTTGTTTTGGCAGGTTATATAAGTGTGAACAC 4800

P6P46 TCTTAGAAAAGTTTCAAGCTTTAACCTTTTTGTTTTGGCAGGTTATATAAGTGTGAACAC 4761

************************************************************

H2P47 AACTGCTTTAGCTAGTGATTGTGTGGTTGCAAAGATGGCTAAGCTCGTAGAAGAAGCTCA 4860

P6P46 AACTGCTTTAGCTAGTGATTGTGTGGTTGCAAAGATGGCTAAGCTCGTAGAAGAAGCTCA 4821

************************************************************

H2P47 GAGCAGTAAAACCAAATCTCAGAGACTAATAGACAAATATTCTCAGTACTATACTCCAGG 4920

P6P46 GAGCAGTAAAACCAAATCTCAGAGACTAATAGACAAATATTCTCAGTACTATACTCCAGG 4881

************************************************************

H2P47 TTTGCAAAAAAACATAAACCATAACTTGTTTTCTTTATGTTCTTGATTCTTGTAATTTGA 4980

P6P46 TTTGCAAAAAAACATAAACCATAACTTGTTTTCTTTATGTTCTTGATTCTTGTAATTTGA 4941

************************************************************

H2P47 GACCTCTCTGTTTTTTGTTTGTTTCAGCAATCATCATAATATCGGCTGGCTTTGCAATTG 5040

P6P46 GACCTCTCTGTTTTTTGTTTGTTTCAGCAATCATCATAATATCGGCTGGCTTTGCAATTG 5001

************************************************************

H2P47 TCCCGGCTATAATGAAAGTTCGCAACCTCAACCATTGGTTTCATTTAGCACTGGTTGTGT 5100

P6P46 TCCCGGCTATAATGAAAGTTCGCAACCTCAACCATTGGTTTCATTTAGCACTGGTTGTGT 5061

************************************************************

H2P47 TAGTCAGTGCTTGTCCCTGTGGTCTTATCCTCTCTACACCAGTAGCTACATTCTGTGCAC 5160

P6P46 TAGTCAGTGCTTGTCCCTGTGGTCTTATCCTCTCTACACCAGTAGCTACATTCTGTGCAC 5121

************************************************************

H2P47 TTACTAAAGCGGCAACTTCAGGGCTTCTGATCAAAAGTGCTGATTATCTTGACACTCTTT 5220

P6P46 TTACTAAAGCGGCAACTTCAGGGCTTCTGATCAAAAGTGCTGATTATCTTGACACTCTTT 5181

************************************************************

H2P47 CAAAGATCAAGATCGCTGCTTTTGACAAAACCGGAACTATCACTAGAGGAGAGTTCATTG 5280

P6P46 CAAAGATCAAGATCGCTGCTTTTGACAAAACCGGAACTATCACTAGAGGAGAGTTCATTG 5241

************************************************************

H2P47 TCATAGAATTCAAGTCACTCTCTAGAGACATAAGCCTAAGCAGCTTGCTTTACTGGTAAT 5340

P6P46 TCATAGAATTCAAGTCACTCTCTAGAGACATAAGCCTAAGCAGCTTGCTTTACTGGTAAT 5301

************************************************************

H2P47 AAAAACAATATCTTGTTCTAACCAAAAACTAGTTTGATGGGATAACGTATGAATGACAAT 5400

P6P46 AAAA-CAATATCTTGTTCTAACCAAAAACTAGTTTGATGGGATAACGTATGAATGACAAT 5360

**** *******************************************************

H2P47 TTCTTGTTTGGTTCTCAGGGTATCAAGTGTTGAAAGCAAATCAAGTCATCCAATGGCAGC 5460

P6P46 TTCTTGTTTGGTTCTCAGG-TATCAAGTGTTGAAAGCAAATCAAGTCATCCAATGGCAGC 5419

******************* ****************************************

H2P47 AACGATTGTGGACTATGCTAAATCTGTTTCTGTTGAGCCTAGGAGTGAAGAGGTTGAGGA 5520

P6P46 AACGATTGTGGACTATGCTAAATCTGTTTCTGTTGAGCCTAGGAGTGAAGAGGTTGAGGA 5479

************************************************************

H2P47 TTATCAGAACTTTCCTGGTGAAGGAATCTATGGGAAGATTGATGGGAACAATGTTTACAT 5580

P6P46 TTATCAGAACTTTCCTGGTGAAGGAATCTATGGGAAGATTGATGGGAACAATGTTTACAT 5539

************************************************************

H2P47 TGGGAACAAAAGGATTGCTTCACGAGCTGGTTGTTCAACAGGTAAAGCTTCAAACTTTGG 5640

P6P46 TGGGAACAAAAGGATTGCTTCACGAGCTGGTTGTTCAACAGGTAAAGCTTCAAACTTTGG 5599

************************************************************

H2P47 CCAAGAAAAAACTCAATGGAATGGTTTTGTTGAGCCTTTGATCATTTTGAAACTGTTCTT 5700

P6P46 CCAAGAAAAAACTCAATGGAATGGTTTTGTTGAGTCCTTAATCATTTTGAAACTGTTCTT 5659

********************************** * ** ********************

H2P47 TCTTGACAGTTCCAGAGATTGATGTTGATACCAAAAAAGGAAAGACTGTCGGATACGTCT 5760

P6P46 CCTTGACAGTTCCAGAGATTGATGTTGATACCAAAGAAGGAAAGACTGTCGGATACGTCT 5719

********************************** ************************

H2P47 ATGTAGGTGAAAGATTAGCTGGAGTTTTCAATCTTTCCGATGCTTGTAGATCCGGAGTAG 5820

P6P46 ATGTAGATGAAAGATTAGCTGGAGTTTTCAATCTTTCTGATGCTTGTAGATCCGGAGTAG 5779

****** ****************************** **********************

H2P47 CTCAAGCAATGAAGGAACTCAAAGATCTTGGAATCAAAACCGCAATGCTAACAGGAGATA 5880

P6P46 CTCAAGCAATGAAGGAACTCAAAGATCTTGGAATCAAAACCGCAATGCTAACAGGAGATA 5839

************************************************************

H2P47 ATAAAGATTCAGCAATGCATGCTCAAGAACAGGTATGAGACTGAAAAAACCAAGAATTTT 5940

P6P46 ATAAAGATTCAGCAATGCATGCTCAAGAACAGGTATGAGACTGAAAAAACCAAGAATTTT 5899

************************************************************

H2P47 TCATTACTCTCCTAACGTTAAGAGATTATATTAAAACTTTGACATGTTCTTATATGGAAC 6000

P6P46 TCATTACTCTCCTAACGTTAAGAGATTATATTAAAACTTTGACATGTTCTTATATGGAAC 5959

************************************************************

H2P47 AGCTAGGGAATGCTTTGGATGTTGTTCATGGAGAGCTTCTTCCTGAAGACAAATCCAAAA 6060

P6P46 AGCTAGGGAATGCTTTGGATGTTGTTCATGGAGAGCTTCTTCCAGAAGACAAATCCAAAA 6019

******************************************* ****************

H2P47 TCATACAAGAGTTTAAGAAAGAAGGACCAACTTGTATGGTAGGAGATGGTGTGAATGATG 6120

P6P46 TCATACAAGAGTTTAAGAAAGAAGGACCAACTTGTATGGTAGGAGATGGTGTGAATGATG 6079

************************************************************

H2P47 CACCAGCTTTAGCTAATGCTGATATTGGTATCTCCATGGGGATTTCTGGCTCTGCGCTCG 6180

P6P46 CACCAGCTTTAGCTAATGCTGATATTGGTATCTCCATGGG-ATTTCTGGCTCTGCGCTCG 6138

**************************************** *******************

H2P47 CGACGCAGTCTGGTCATATCATTCTCATGTCAAATGATATCAGAAGGATACCAAAAGCGA 6240

P6P46 CGACGCAGTCTGGTCATATCATTCTCATGTCAAATGATATCAGAAGGATACCAAA-GCGA 6197

******************************************************* ****

H2P47 TAAAGCTAGCAAGAAGAGCTCAGCGGAAAGTTCTTGAAAACGTGTTCATCTCCATCACTT 6300

P6P46 TAAAGCTAGCAAGAAGAGCTCAGCGGAAAGTTCTTGAAAACGTGTTCATCTCCATCACTT 6257

************************************************************

H2P47 TGAAAGTAGGGATACTGGTTTTAGCATTTGCTGGTCATCCTTTGATTTGGGCTGCGGTGC 6360

P6P46 TGAAAGTAGGGATACTGGTTTTAGCATTTGCTGGTCATCCTTTGATTTGGGCTGCGGTGC 6317

************************************************************

H2P47 TTACTGATGTAGGGACTTGCCTGATTGTGATTTTTAACAGTATGTTGCTTCTGCGAGAGA 6420

P6P46 TTACTGATGTAGGGACTTGCCTGATTGTGATTTTTAACAGTATGTTGCTTCTGCGAGAGA 6377

************************************************************

H2P47 AGGATAAATCTAAGAACAAGAATTGTTACAGGGCTTCTACATCTGTGTTGAATGGTAAGA 6480

P6P46 AGGATAAATCTAAGAACAAGAATTGTTACAGGGCTTCTACATCTGTGTTGAATGGTAAGA 6437

************************************************************

H2P47 AACTTGAAGGCGGCGATGACCAAGGCCTTGACTTAGAAGCAGGGTTGTTCTCAAAGAGTC 6540

P6P46 AACTTGAAGGCGATGATGAAGAAGGTCTTGACTTAGAAGCAGGGTTGGTATCAAAGAGTC 6497

************ ***** **** ********************* * **********

H2P47 AATGCAACTCAGGATGTTGTGGTGATAAGAAAAGCCAAGAGAAGGTGATGTTGATGAGAC 6600

P6P46 AATGCAACTCAGGATGTTGTGGTGATAAGAAAAGCCAAGAGAAGGTGATGTTGATGAGAC 6557

************************************************************

H2P47 CAGCTAGTAAAACCAGTACTGACCATCTTCACTCTGGTTGTTGTGGTGAAAAGAATCAAG 6660

P6P46 CAGCTAGTAAAACCAGTACTGACCATCTTCACTCTGGTTGTTGTGGTGAAAAGA-TCAAG 6616

****************************************************** *****

H2P47 AGAGTGTAAAGCTTGTGAAAGATAGCTGTTGCGGTGAGAAAAGTAAGAAACCAGAGGGAG 6720

P6P46 AGAGTGTAAAGCTTGTGAAAGATAGCTGTTGCGGTGAGAAAAGTAAGAAACCAGAGGGAG 6676

************************************************************

H2P47 ATATGGCTTCACTGAGCTCATGCAAGAACTCTAACAATGACCTGAAAATGAAAGGTGGTT 6780

P6P46 ATATGGCTTCACTGAGCTCATGCAAGAACTCTAACAATGACCTGAAAATGAAAGGTGGTT 6736

************************************************************

H2P47 CAAGTTGTTGTGCTAGTAAAAATGAGAAGCTGAAGGAAGTAGTAGTAGCAAAGAGCTGCT 6840

P6P46 CAAGTTGTTGTGCTAGTAAAAATGAGAAGCTGAAGGAAGTAGTAGTAGCAAAGAGCTGCT 6796

************************************************************

H2P47 GTGGAGAGAAGGAGAAAGCAGAGGGAAATGTTGAGATGCAGATTCTAAATTTGGAGAAAG 6900

P6P46 GTGGAGAGAAGGAGAAAGCAGAGGGAAATGTTGAGATGCAGATTCTAAATTTGGAGAAAG 6856

************************************************************

H2P47 GGTCGCAGAAAAAGGTTGGTGAAACCTGCAAATCAAGCTGTTGTGGAGATAAAGAGAAGG 6960

P6P46 GGTCGCAGAAAAAGGTTGGTGAAACCTGCAAATCAAGCTGTTGTGGAGATAAAGAGAAGG 6916

************************************************************

H2P47 CTAAGGAAACACGTTTGTTGCTTGCTAGTGAGGATCCATCTTATCTGGAGAAGGAGAATC 7020

P6P46 CTAAGGAAACACGTTTGTTGCTTGCTAGTGAGGATCCATCTTATCTGGAGAAGGAGAATC 6976

************************************************************

H2P47 TGAAAAGTGAAAGTGGTGATGATTGCAAATCTCTTTGTTGTGGAACTGGTTTGAAGCAAG 7080

P6P46 TGAAAAGTGAAAGTGGTGATGATTGCAAATCTCTTTGTTGTGGAACTGGTTTGAAGCAAG 7036

************************************************************

H2P47 AAGGGTCTTCTAGTTTGGTCAATGTTGTGGTGGAGAGTGGTGAATCCGGGTCAAGCTGTT 7140

P6P46 AAGGGTCTTCTAGTTTGGTCAATGTTGTGGTGGAGAGTGGTGAATCCGGGTCAAGCTGTT 7096

************************************************************

H2P47 GCAGCAAGGAGGGAGAGATAGTGAAAGTCTCTAGCCAAAGCTGTTGCACAAGTCCAAGTG 7200

P6P46 GCAGCAAGGAGGGAGAGATAGTGAAAGTCTCTAGCCAAAGCTGTTGCACAAGTCCAAGTG 7156

************************************************************

H2P47 ATGTGGTGTTATCTGACTTTCAAGCTAAGAAACTAGAGATTTGTTGCGAAGTGAAGAAGA 7260

P6P46 ATGTGGTGTTATCTGACTTTCAAGCTAAGAAACTAGAGATTTGTTGCGAAGTGAAGAAGA 7216

************************************************************

H2P47 CTCCAGAGGAGGTTTGTGGATCTAAATGTAAGGAAACAGAGAAGCCTCACCACGTTGGTA 7320

P6P46 CTCCAGAGGAGGTTTGTGGATCTAAATGTAAGGAAACAGAGAAGCCTCACCACGTTGGTA 7276

************************************************************

H2P47 AAAGCTGTTGCAGGAGTTATGCAAAAGAGTATTGCAGCCACAGGCATCACGACAACCATC 7380

P6P46 AAAGCTGTTGCAGGAGTTATGCAAAAGAGTATTGCAGCCACAGGCATCACGACAACCATC 7336

************************************************************

H2P47 ATCACCACCATGTTGGGGCTGCTTGACGGAGATAGTGATTGATTACCTTTAAACTCTCGA 7440

P6P46 ATCACCACCATGTTGGGGCTGCTTGACGGAGATAGTGATTGATTACCTTTAAACTCTCGA 7396

************************************************************

H2P47 CCCATCCATCTATTTGCATAACCTTTCCTTCTTCAACCAATGTCGCCCAGAACAAAATAA 7500

P6P46 CCCATCCATCTATTTGCATAACCTTTCCTTCTTCAACCAATGTCGCCCAGAACAAAATAA 7456

************************************************************

H2P47 AAACTTATTTAGTGTTTCCAGCAAAGGTGTGATTCGTAAAGACAATGCTGTTGATCGTTG 7560

P6P46 AAACTTATTTAGTGTTTCCAGCAAAGGTGTGATTCGTAAAGACAATGCTGTTGATCGTTG 7516

************************************************************

H2P47 TTTGTCTTTTATGTTTGCCAAAACCATAATGTATTTCTCCTTTTCTTGTTTTTATTCTCT 7620

P6P46 TTTGTCTTTTATGTTTGCCAAAACCATAATGTATTTCTCCTTTTCTTGTTTTTATTCTCT 7576

************************************************************

H2P47 TCTTGAAGATGCCCAGAAGAAGTTTGAACTTCGATCCTAGAGTCTTAAAATCAAATAGAA 7680

P6P46 TCTTGAAGATGCCCAGAAGAAGTTTGAACTTCGATCCTAGAGTCTTAAAATCAAATAGAA 7636

************************************************************

H2P47 CAAGCAGTTGAAACATAACTTAGCCTTGGAGTCTTTTTGTATGCTGTGTACTACATAAGC 7740

P6P46 CAAGCAGTTGAAACATAACTTAGCCTTGGAGTCTTTTTGTATGCTGTGTACTACATAAGC 7696

************************************************************

H2P47 TTTCTTGACTGACACGTTTCTTGTCAATTCTAGGGCATTACTTTATTAGGACAGAGAAGG 7800

P6P46 TTTCTTGACTGACACGTTTCTTGTCAATTCTAGGGCATTACTTTATTAGGACAGAGAAGG 7756

************************************************************

H2P47 TGTTGCAGTTCGTGTCCTGGAGAGTTTAGGTGAAAAAAAAAATAAAGAGCAAAAACTGAC 7860

P6P46 TGTTGCAGTTCGTGTCCTGGAGAGTTTAGGTGAAAAAAAAA-TAAAGAGCAAAAACTGAC 7815

***************************************** ******************

H2P47 TGCTCGCACATCCATGTAATCAAGAATCAGTAAAAATAAAAATTAATCAAAGGGTGACAC 7920

P6P46 TGCTCGCACATCCATGTAATCAAGAATCAGTAAAAATAAAAATTAATCAAAGGGTGACAC 7875

************************************************************

H2P47 AGCTCATGATCTTATATGAATCACCAACCATACTCTTCTCACTATATAAACAAATGTGTC 7980

P6P46 AGCTCATGATCTTATATGAATCACCAACCATACTCTTCTCACTATATAAACAAATGTGTC 7935

************************************************************

H2P47 ATTTCTTGAAAACAATCTGAAATATTCCGCAATCGCTGAAAGCATAGCATTAGAGGCAAA 8040

P6P46 ATTTCTTGAAAACAATCTGAAATATTCCGCAATCGCTGAAAGCATAGCATTAGAGGCAAA 7995

************************************************************

H2P47 ACCCTAGCTATTCTTTTGTTCTCCGTCTTTATTTCTTATCTTTTATTTCGTCAAGCTTGT 8100

P6P46 ACCCTAGCTATTCTTTTGTTCTCCGTCTTTATTTCTTATCTTTTATTTCGTCAAGCTTGT 8055

************************************************************

H2P47 CGATGAGGTGAAAACCTTCTTAAAATATATTATTCGCTTCTTCAAGTTTAATAAGACATA 8160

P6P46 CGATGAGGTGAAAACCTTCTTAAAATATATTATTCGCTTCTTCAAGTTTAATAAGACATA 8115

************************************************************

H2P47 TACCCTAAGTTCCACAAACTTTTGTATCTCGAGATAAAACTTGTGACACATCTGATTCAA 8220

P6P46 TACCCTAAGTTCCACAAACTTTTGTATCTCGAGATAAAACTTGTGACACATCTGATTCAA 8175

************************************************************

H2P47 CACGAAACCTAATGTTTTTAAGATCTACTAGGTAGGATACTTGCGCTTCGCCGCGGAAGA 8280

P6P46 CACGAAACCTAATGTTTTTAAGATCTACTAGGTAGGATACTTGCGCTTCGCCGCGGAAGA 8235

************************************************************

H2P47 CTTTTTTTGTATTTTGATATTTTATATTTTGATATTTTAATCCAGTTTTCCTTATATTCC 8340

P6P46 CTTTTTTTGTATTTTGATATTTTATATTTTGATATTTTAATCCAGTTTTCCTTATATTCC 8295

************************************************************

H2P47 ATCTGTTCCATATTAAAATGTCGTTTAAGATTTTTTCATACATATTAAGAAAATATTAAA 8400

P6P46 ATCTGTTCCATATTAAAATGTCGTTTAAGATTTTTTCATACATATTAAGAAAATATTAAA 8355

************************************************************

H2P47 ATTTTTTATTTTACTCATTATTACTCAAAATTAATCTTCTCAAGGAGAAATGTGTAAAAT 8460

P6P46 ATTTTTTATTTTACTCATTATTACTCAAAATTAATCTTCTCAAGGAGAAATGTGTAAAAT 8415

************************************************************

H2P47 CTTTGATGAATTCTCTTAGAAGGAAAGGAAGAATCATGAGTTTGATCTCCATCAATTTCA 8520

P6P46 CTTTGATGAATTCTCTTAGAAGGAAAGGAAGAATCATGAGTTTGATCTCCATCAATTTCA 8475

************************************************************

H2P47 GAGAAAACTGCTTCCTCTTCCTCCTCCAGGTTGTTCTTCTTCTTCCTCCATTCTCTTATG 8580

P6P46 GAGAAAACTGCTTCCTCTTCCTCCTCCAGGTTGTTCTTCTTCTTCCTCCATTCTCTTATG 8535

************************************************************

H2P47 CTCCGACGCGGAATCGGCTTAAGACTTTTAAAGAAACACAGTCTCTAAACGCTCGATTTA 8640

P6P46 CTCCGACGCGGAATCGGCTTAAGACTTTTAAAGAAACACAGTCTCTAAACGCTCGATTTA 8595

************************************************************

H2P47 CGTTTTCCGGTTAAATGAAGGAAATCAATAAATGAAAACCGGATAAAATTAAACCAAAAA 8700

P6P46 CGTTTTCCGGTTAAATGAAGGAAATCAATAAATGAAAACCGGATAAAATTAAACCAAAAA 8655

************************************************************

H2P47 AATTGCTGACTGATATTATTTATAATATTTATATTAGATGTTTGTTTTAAATAACGTGAA 8760

P6P46 AATTGCTGACTGATATTATTTATAATATTTATATTAGATGTTTGTTTTAAATAACGTGAA 8715

************************************************************

H2P47 GTAGATTATCTTGAGAAGCACATGAGATTTTTCTGTGCTGATTAACTACAATATCTTTAC 8820

P6P46 GTAGATTATCTTGAGAAGCACATGAGATTTTTCTGTGCTGATTAACTACAATATCTTTAC 8775

************************************************************

H2P47 AAAAAAAAAACAACAGCAATTCCTCGAATGACACACGTAAAAATTAACTCCAAATAATGT 8880

P6P46 AAAAAAAAAACAACAGCAATTCCTCGAATGACACACGTAAAAATTAACTCCAAATAATGT 8835

************************************************************

H2P47 AACTTCATTTTTACCCCAGAATTAATGGTTGATTCCACCAATTTCATTATAATAGCATTA 8940

P6P46 AACTTCATTTTTACCCCAGAATTAATGGTTGATTCCACCAATTTCATTATAATAGCATTA 8895

************************************************************

H2P47 CCATTTAGGTCTCTGGTGATGTTTATGTTTGTATCACCATTCATTAGTTATATAAAATAT 9000

P6P46 CCATTTAGGTCTCTGGTGATGTTTATGTTTGTATCACCATTCATTAGTTATATAAAATAT 8955

************************************************************

H2P47 ATAATGCTAATGTT 9014

P6P46 ATAATGCTAATGTT 8969

**************

*Noccaea caerulescens* *HMA*4-4 genomic sequence 5’ to 3’ is highlighted in yellow.
