# Supplementary material for: Tandem Quadruplication of HMA4 in the Zinc (Zn) and Cadmium (Cd) Hyperaccumulator Noccaea caerulescens
Source: PLoS One. 2011 Mar 10;6(3):e17814. doi: 10.1371/journal.pone.0017814 (PMC3053397; doi:10.1371/journal.pone.0017814)
Supplement: Data S8 — Sequence alignment of overlapping regions of fosmids H2P47 and B3P40. (DOC) [file pone.0017814.s016.doc]

**Data S8 Sequence Alignment of overlapping regions of fosmids H2P47 and B3P40.**

CLUSTAL 2.0.12 multiple sequence alignment

H2P47 TTTTAGAATATATCACGTAGATCGTGGACAATAGGTGTGTGAACATATATATATATAT-- 58

B3P40 TTTTAGAATATATCACGTAGATCGTGGACAATAGGTGTGTGAACATATATATATATATAT 60

**********************************************************

H2P47 --TCAGACATATAACAATAAAATATGTACACAAGCTTCGATTTTCACCTCTGATACAACA 116

B3P40 ATTCAGACATATAACAATAAAATATGTACACAAGCTTCGATTTTCACCTCTGATACAACA 120

**********************************************************

H2P47 ATTCATCCACATCTTTATTATGTCCAAACAAAAGTAATCCACGGCAATAAATCTACATAA 176

B3P40 ATTCATCCACATCTTTATTATGTCCAAACAAAAGTAATCCACGGCAATAAATCTACATAA 180

************************************************************

H2P47 TTTGGCTCTCTTTGATTCTTCCACTCTTACTTTCATCTTTTTATTTCCGTAATCACAAGC 236

B3P40 TTTGGCTCTCTTTGATTCTTCCACTCTTACTTTCATCTTTTTATTTCCGTAATCACAAGC 240

************************************************************

H2P47 AACAATGTTTCATTTTCACTTCTCGTTATCATCATATATTACAATTTTTTACCCATGTTA 296

B3P40 AACAATGTTTCATTTTCACTTCTCGTTATCATCATATATTACAATTTTTTACCCATGTTA 300

************************************************************

H2P47 ACCCTATCAAAACACCAATTAGATACATGAAAACAATTCAATCCCACACCAAACCAGAAA 356

B3P40 ACCCTATCAAAACACCAATTAGATACATGAAAACAATTCAATCCCACACCAAACCAGAAA 360

************************************************************

H2P47 TCGTTTTAATTTATAGCTTCAGTTTCAGAAATTAATATACGCCAAAAAACGTTGGTTACT 416

B3P40 TCGTTTTAATTTATAGCTTCAGTTTCAGAAATTAATATACGCCAAAAA-CGTTGGTTACT 419

************************************************ ***********

H2P47 GTGAAACAC 425

B3P40 GTGAAACAC 428

*********
